# Substance P promotes immunotherapy efficacy for airway allergy^☆^

**DOI:** 10.1016/j.waojou.2022.100730

**Published:** 2022-12-15

**Authors:** Yongjin Wu, Yu Liu, Xinxin Wang, Huazhen Liu, Gaohui Wu, Liteng Yang, Li Guan, Qinmiao Huang, Xianhai Zeng, Pingchang Yang

**Affiliations:** a Department of Allergy, Longgang ENT Hospital, Shenzhen Key Laboratory of ENT & Shenzhen ENT Institute, Shenzhen, China; b Departments of Respirology and Allergy, Third Affiliated Hospital, Shenzhen University, Shenzhen, China; c Guangdong Provincial Key Laboratory of Regional Immunity and Diseases, Shenzhen, China; d Institute of Allergy & Immunology, Shenzhen University School of Medicine, Shenzhen, China; e State Key Laboratory of Respiratory Diseases Shenzhen University Division, Shenzhen, China; f Shenzhen Municipal Key Laboratory of Allergy & Immunology, Shenzhen, China; g Guangdong Provincial Center for Standardized Allergen Engineering, Shenzhen, China

**Keywords:** Airway allergy disorder, Dendritic cells, Interleukin-10, Semaphorin, Immunotherapy, AIt, allergen-specific immunotherapy, SP, Substance P, Tr1 cell, Type 1 regulatory T cell, DEG, Differentially expressed gene

## Abstract

**Background:**

Allergen-specific immunotherapy (AIT) has been employed in the treatment of allergic diseases for many years. However, the effectiveness of AIT requires improvement. Substance P (SP) can interact with immune cells, modulate immune cell activity, and regulate immune reaction. The purpose of this study is to use SP as an immune regulator to enhance the therapeutic efficacy of AIT.

**Methods:**

An established mouse model of the airway allergy disorder (AAD) was employed with ovalbumin as a specific antigen. The AAD response was evaluated through established procedures. AAD mice were treated with AIT employing SP as an immune regulator. Dendritic cells were isolated from the airway tissues by magnetic cell sorting, and were analyzed by RNA sequencing (RNAseq).

**Results:**

We observed that after sensitization with ovalbumin, mice exhibited AAD-like symptoms, serum specific IgE, and Th2 polarization. The presence of SP in the course of sensitization prevented the development of AAD. Treating mice with SP by nasal instillations induced IL-10, but not TGF-β, in dendritic cells of the airway tissues. The most differentially expressed genes (DEG) in the dendritic cells were those related to the IL-10 expression, including *Il10*, *Tac1r*, and *Mtor*. The gene ontology analysis showed that these DEGs mainly mapped to the tachykinin-PI3K-AKT-mTOR pathway. The addition of SP substantially enhanced the therapeutic efficacy of AIT for AAD by inducing antigen specific type 1 regulatory T cells (Tr1 cells).

**Conclusion:**

Acting as an immune regulator, SP promotes the therapeutic efficacy for AAD by inducing antigen specific Tr1 cells in the airway tissues.

## Introduction

Airway allergic disorders (AAD) are an aberrant immune response to innocent airborne antigens by the immune system in the airway tissues.^1^^,^^2^ The offending allergic mediators mainly include those released by sensitized mast cells and eosinophils. For example, histamine, proteases, serotonin, leukotriene, major basic protein, and eosinophil peroxidase are commonly detected in the allergic lesions. Antigen specific IgE binds the high affinity IgE receptor on the surface of mast cells to make mast cells sensitized.^3^^,^^4^ Mast cells release allergic mediators as they are re-exposed to specific antigens. Another characteristic of AAD is Th2 polarization, which is represented by abnormally aggregated Th2 cells in allergic lesion sites.^5^^,^^6^ Although AAD has been extensively investigated, its pathogenesis is still poorly understood.

There are many therapeutic tools available for treating AAD.^7^ For example, corticosteroid nasal spray, antihistamines, and leukotriene blockers, are widely used in allergy clinics. These medications are generally effective in controlling the allergic response. However, they only work temporarily.^1^^,^^8^^,^^9^ To date, only allergen-specific immunotherapy (AIT) is an etiologic therapy for allergic conditions.^10^^,^^11^ AIT is to introduce specific allergens into patients at low doses in a progressive manner. The goal of AIT is to induce blocking antibodies and induce immunoregulatory T and B lymphocytes in the body. They then suppress the allergic response.^10^ Although AIT has been used at allergy clinics for many years, the prevalence of allergic diseases is steadily increasing.^11^ The point is that the therapeutic effectiveness of AIT needs to be improved.

Published data indicate that neuropeptides, such as substance P (SP), vasoactive intestinal peptide, and calcitonin gene-related peptide, could be detected in nasal secretions. Therefore, it is proposed that neuropeptides are associated with AAD.^12^ Studies also show that SP can interact with immune cells in response to different stimuli at multiple levels.^13^^,^^14^ By releasing from the nervous endings, SP can recruit immune cells and promote the proliferation of immune cells.^13^^,^^15^ Induction of histamine release and eosinophil recruitment by SP have also been reported.^16^^,^^17^ On the other hand, SP can stimulate the production of immunoregulatory cytokine IL-10.^18^ This implicates an immune adjuvant effect of SP. Immune adjuvants can accelerate immune response. Therefore, we hypothesize that SP may have immune adjunct properties to favor AIT. To test the hypothesis, we employed an established murine AAD model. AAD mice were treated with AIT with or without SP. We found that the SP greatly improved the effectiveness of the AIT. The results suggest that SP can be a good candidate of immune regulator to be used in immunotherapy.

## Materials and methods

### Establishment of a murine AAD model

**Fig. 1:**
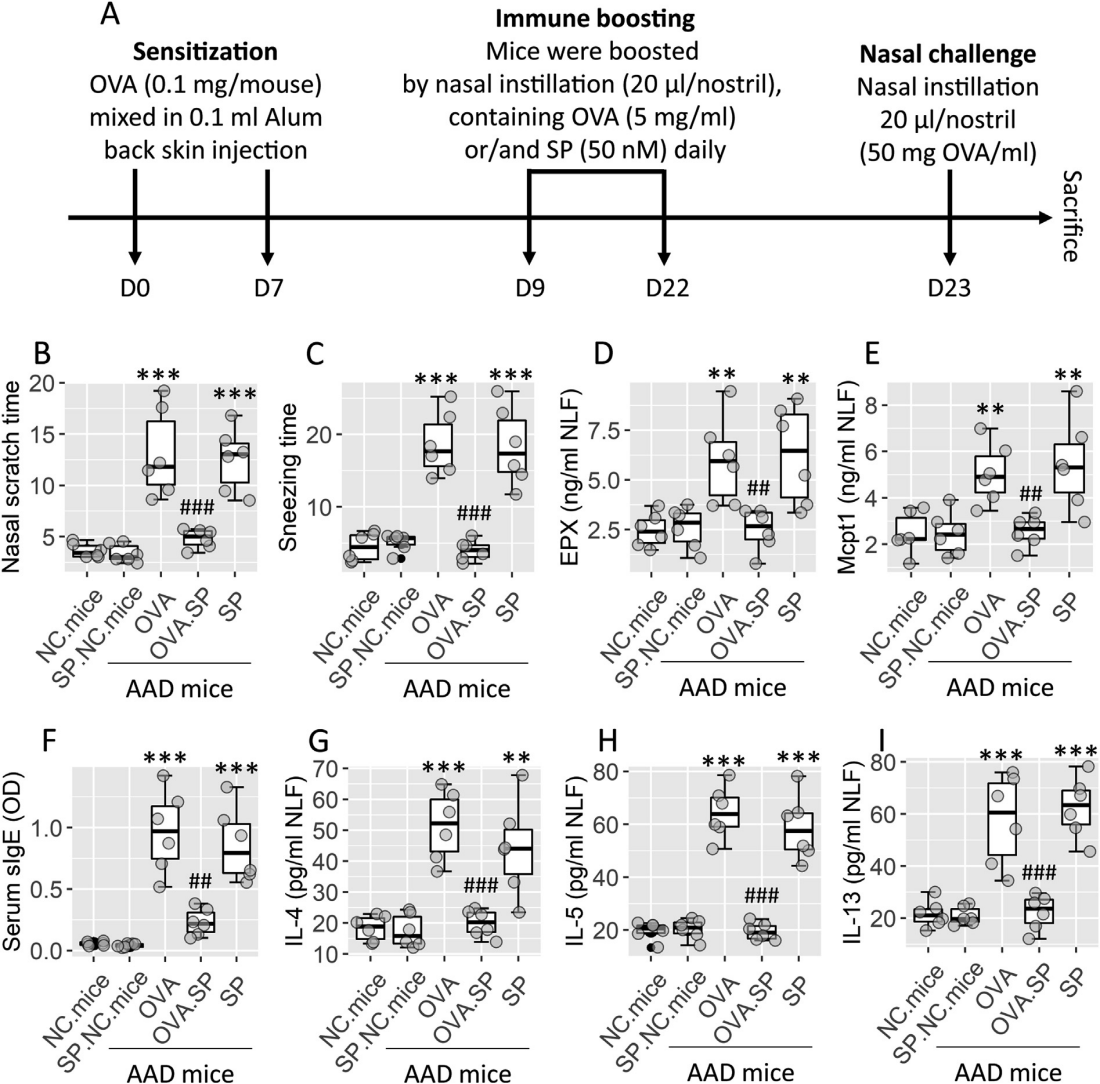
**SP prevents AAD development**. A, a protocol of AAD mouse model development. B–C, AAD clinical symptom (nasal itch and sneezing) records. D-E, allergic mediators (Mcpt1 and EPX) in NLF. F, serum sIgE levels. G-I, serum Th2 cytokine levels. Boxplots show median (IQR) of AAD parameters from 6 mice per group. Each dot presents data obtained from one sample. Statistics: ANOVA + Dunnett's test. ∗∗ (p < 0.01), ∗∗∗ (p < 0.001), compared with the NC mouse group. ## (p < 0.01), ### (p < 0.001), compared with the OVA group (sensitized group). **Abbreviations**: NC: Naïve control. OVA: Ovalbumin. SP: Substance P. AAD: Airway allergy disorder. Mcpt1: Mouse mast cell protease-1. EPX: Eosinophil peroxidase. NLF: Nasal lavage fluid. sIgE: Specific IgE.

Mice were randomly grouped into naïve control (NC) group and AAD group (the sensitized group). AAD group mice were further grouped into PBS group (treated with PBS), AIT group (treated with AIT), SP group (treated with SP), AIT/SP group (treated with both AIT and SP), and AIT/SP/KO group (*Tacr1*^ΔDC^ mice were treated with AIT and SP). Each group consisted of 6 mice. A mouse model of AR was employed following our established procedures^19^ that are depicted in Fig. 1A. Briefly, mice were sensitized by subcutaneously injected with ovalbumin (OVA, 0.1 mg/mouse mixed in 0.1 mL alum) on day 1 and day 7. Mice were boosted by nasal instillation (20 μL/nostril, containing 5 mg OVA/ml) daily from day 9 to day 21. Mice were challenged through the nasal instillation (20 μL/nostril, containing 50 mg OVA/ml). The AAD response was assessed and recorded following the challenge.

### Administration of AIT with SP as an immune regulator

One day after the completion of antigen-boosting, mice were treated with AIT with or without SP (50 nM, mixed with OVA) as an immune regulator by nasal instillation. Briefly, AAD mice were treated with nasal instillation [20 μL/nostril/day, containing OVA in escalating doses: 1 mg/mL (following the date-counts above: D22 and D23), 5 mg/mL (D24 and D25), 10 mg/ml (D26 and D27), 25 mg/mL (D28 and D29), and 50 mg/mL (D30-35)]. The control group of AAD mice was treated with PBS nasal instillation. One day after the last treatment as shown in Fig. 1A, the AAD response was assessed for each mouse.

### Assessment of AAD response

(1) AAD clinical symptoms (nasal scratch times and sneezing times) in AAD mice were observed during 30 min after antigen challenge. (2) Allergic mediator measurement: Nasal lavage fluid (NLF) was collected from each mouse. Briefly, after the sacrifice, the trachea was exposed; a syringe needle was inserted into the trachea (in the nose direction; the nostril was placed in the lower position). The nasal cavity was rinsed with 1 ml saline. The NLF was collected with an Eppendorf tube. The amounts of Mcpt1 and EPX in NLF were determined by ELISA. (3) serum sIgE and Th2 cytokine level measurement: Mice were sacrificed by decapitation. The trunked blood was collected with a test tube. Serum samples were prepared by centrifugation. Amounts of sIgE and Th2 cytokines were determined by ELISA.

### Isolation of airway mononuclear cells

Upon the sacrifice, the nasal mucosa and the lungs were excised, cut into small pieces, and incubated with collagenase IV (0.5 mg/mL) for 20 min with mild agitation. Single cells were passed through a cell strainer (70 μm first, then 40 μm). Airway mononuclear cells (AMCs) were isolated from the single cells by the Percoll gradient density centrifugation.

### Immune cell isolation

Dendritic cells (DCs), CD4^+^ T cells, and Tr1 cells were isolated from AMCs or spleen cells with commercial reagent kits, or stained with fluorescence-labeled Abs. The cells were then purified by magnetic cell sorting, or flow cytometry cell sorting. Isolated cells were post-checked by FCM. If purity did not reach 95%, the purification procedures were repeated.

### Assessment of immune suppressive capacity of Tr1 cells

Tr1 cells were purified from AMCs of AAD mice treated with SP/AIT or AIT alone by FCM cell sorting. DCs and CD4^+^ CD25¯ T cells (Teffs, labeled with CFSE) were purified from spleen cells of DO11.10 mice by magnetic cell sorting. Tr1 cells were cocultured with Teffs and DCs at a ratio of 1:5:1 in the presence of ovalbumin (OVA, 1 μg/mL; BSA was used as an irrelevant antigen) for 3 days. Cells were then analyzed by FCM.

### Statistics

The difference between the 2 groups was determined by Student's *t*-test. ANOVA + Dunnett's test or Bonferroni test was performed for multiple comparisons. *P* < 0.05 was set as a significant criterion.

Some experimental procedures are presented in (Supplementary material).

## Results

### SP prevents AAD development

A mouse AAD model was employed using ovalbumin as a specific antigen (Fig. 1A). As compared to naïve control (NC) mice, AAD mice showed the AAD response, including nasal itch (nasal scratches), sneezing (Fig. 1B–C), and increase in allergic mediators (Mcpt1 and EPX) in NLF (Fig. 1D–E), increase in sIgE and Th2 cytokines in the serum (Fig. 1F–I). Combination of SP and AIT during the boosting period (Fig. 1A) prevented the development of AAD. Exposure to SP alone did not impact the AAD parameters (allergic mediators and clinical symptoms) as shown in Fig. 1B–I.

#### Administration of SP increases immune tolerance factors in the airway tissues

**Fig. 2:**
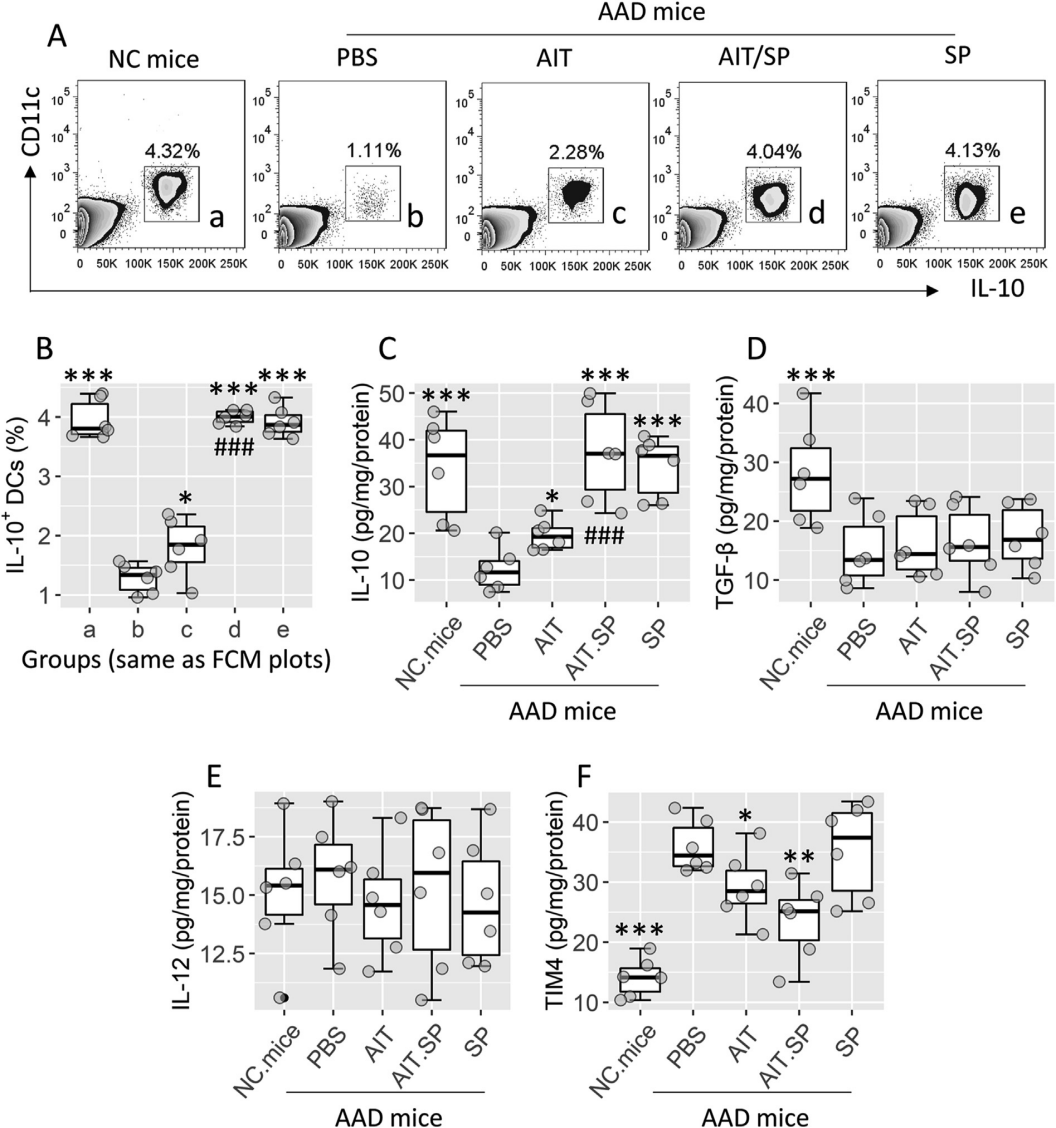
**Assessment of immune regulatory effects of SP in the airway tissues of AAD mice**. The airway tissues were collected from AAD mice treated with AIT or/and SP. A-B, airway mononuclear cells (AMCs) were prepared with the airway tissues, and analyzed by FCM. Gated FCM plots show IL-10^+^ DC counts. Boxplots show IL-10^+^ DC frequency in AMCs in panel A. C–F, protein extracts were prepared with the isolated DCs, and analyzed by ELISA. Boxplots show the levels of IL-10, TGF-β, IL-12, and TIM4 in the protein extract samples. Statistics: ANOVA + Dunnett's test. ∗ (p < 0.05), ∗∗∗ (p < 0.001), compared with the AAD. mice.PBS group. ### (p < 0.001), compared with the AAD. mice.AIT group. Data in panel A are from one experiment that represent 6 independent experiments. **Abbreviations**: NC: Naïve control. AAD: Airway allergy disorder. PBS: Phosphate-buffered saline. AIT: Allergen specific immunotherapy. SP: Substance P. DC: Dendritic cell. FCM: Flow cytometry.

Restoration of immune tolerance is critical for the therapeutic efficacy of AIT.^8^ Data of Fig. 1 suggests that concomitant administration of the AIT and SP can be particularly beneficial for immune tolerance factors in airway tissues. To test this, after completing the sensitization, the mice were treated with AIT or/and SP in a 2-week period. Since IL-10^+^ DCs play a vital role in the immune tolerance,^20^^,^^21^ we assessed IL-10^+^ DCs in the airway tissues by flow cytometry (FCM). The results showed that the AIT/SP therapy or SP alone markedly increased the frequency of IL-10^+^ DCs in the airway tissues of AAD mice. AIT alone slightly increased (*P* < 0.05) the frequency of IL-10^+^ DCs in airway tissues (Fig. 2A–B). Immune-regulatory mediators in the respiratory tissues of mice were then evaluated after AIT or/and SP therapy. Levels of IL-10 in the airway tissues were significantly higher in AAD mice treated with AIT/SP or SP alone than those treated with AIT alone. It is noteworthy that treated with SP alone also increased the IL-10 levels in airway DCs (Fig. 2C). AIT/SP therapy slightly increased TGF-β levels in respiratory tissues, but did not meet the significant criterion (Fig. 2D). Additionally, the treatment with AIT or/and SP did not alter the levels of IL-12 in airway DCs. TIM4 levels in airway DCs were also decreased by AIT, that were further decreased by AIT/SP (Fig. 2E–F). The results suggest that administration of SP facilitates immune tolerance associated with IL-10 in the tissues of the respiratory tract.

#### Lower NK1 receptor signaling is detected in AAD DC of airway tissues

**Fig. 3:**
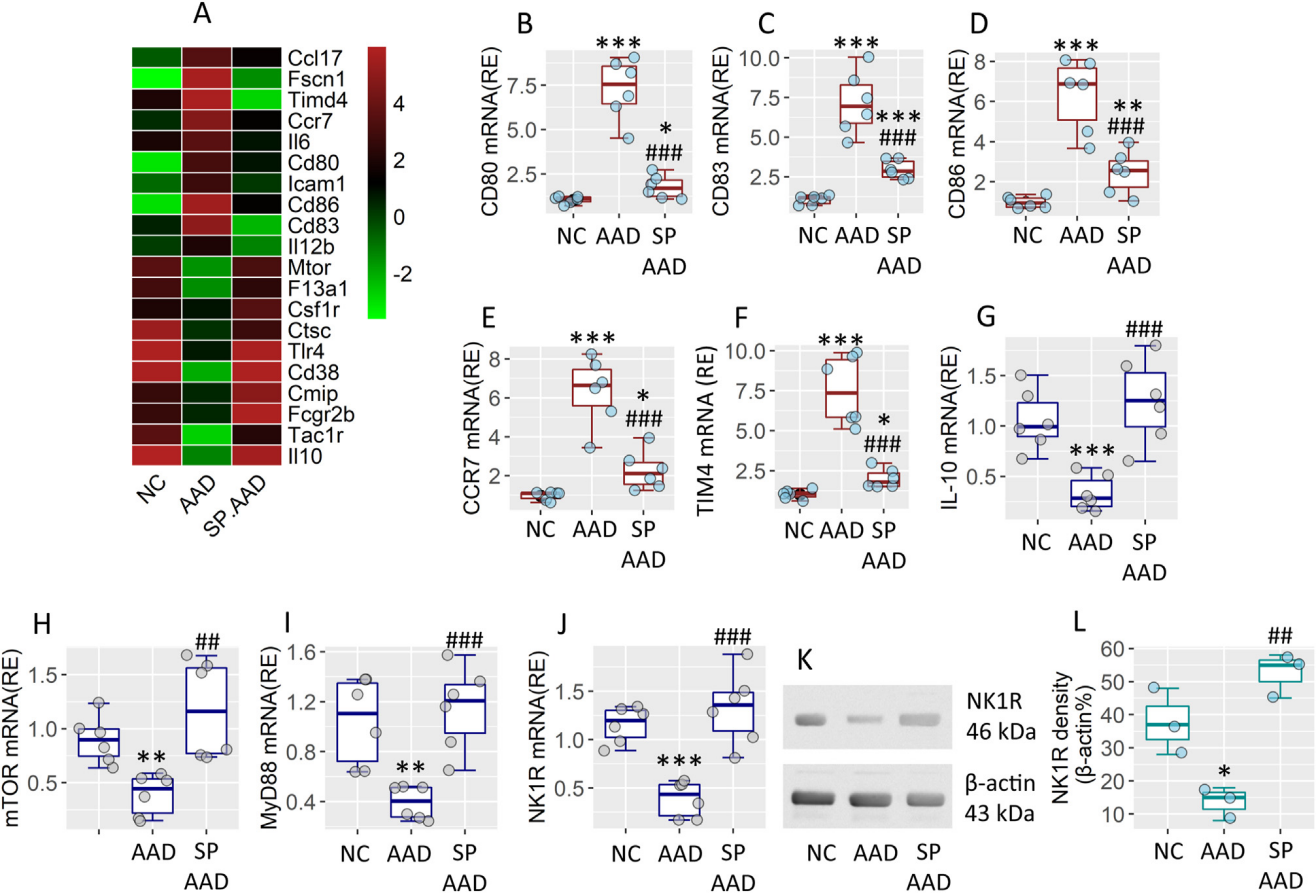
**Analysis of transcriptome of AAD DCs**. DCs were isolated from the airway tissues of NC mice, AAD mice, SP-treated AAD mice, and analyzed by RNAseq and RT-qPCR. A, heatmap show the top 20 differentially expressed genes in DCs. B-J, boxplots show median (IQR) of indicated gene expression levels from 6 mice per group. K-L, immunoblots show NK1R protein levels in DC protein extracts. Boxplots show integrated density of the NK1R immunoblots. The data of K are from one experiment that represent 3 independent experiments using pooled protein from 6 mice per group. Statistics: ANOVA + Dunnett's test. ∗ (p < 0.05), ∗∗ (p < 0.01), ∗∗∗ (p < 0.001), compared with the NC group. ## (p < 0.01, ### (p < 0.001), compared with the AAD group. **Abbreviations**: NC: Naïve control. AAD: Airway allergy disorder. SP: Substance P. DC: Dendritic cell. RNAseq: RNA sequencing. TIM4: T cell immunoglobulin domain molecule-4.

After the treatment described in Fig. 2, DCs were isolated from the airway tissues of AAD mouse and analyzed by RNA sequencing (RNAseq). As shown by the profile of differentially expressed gene (DEG), weaker activities of the IL-10 expression, mTOR and MyD88 signals were observed in the AAD DCs. On the contrary, the maturation parameters of AAD DCs (CD80, CD83 and CD86), DC trafficking chemokine receptor (*Ccr7*) and Th2 response-inducing tendency (*Timd4*) were at higher levels in AAD DCs. The AAD DEG profile was reversed after treatment with SP (Fig. 3A). The DEG profile of RNAseq in airway DCs was verified by conventional RT-qPCR (Fig. 3B–J). We specifically observed the expression of NK1R, the receptor of SP, in DCs. The levels of NK1R in airway DCs were lower in AAD DCs, that were significantly increased by the treatment with SP (Fig. 3K-L).

#### NK1R–PI3K-AKT-mTOR pathway mediates the effects of SP on regulating DC's properties

**Fig. 4:**
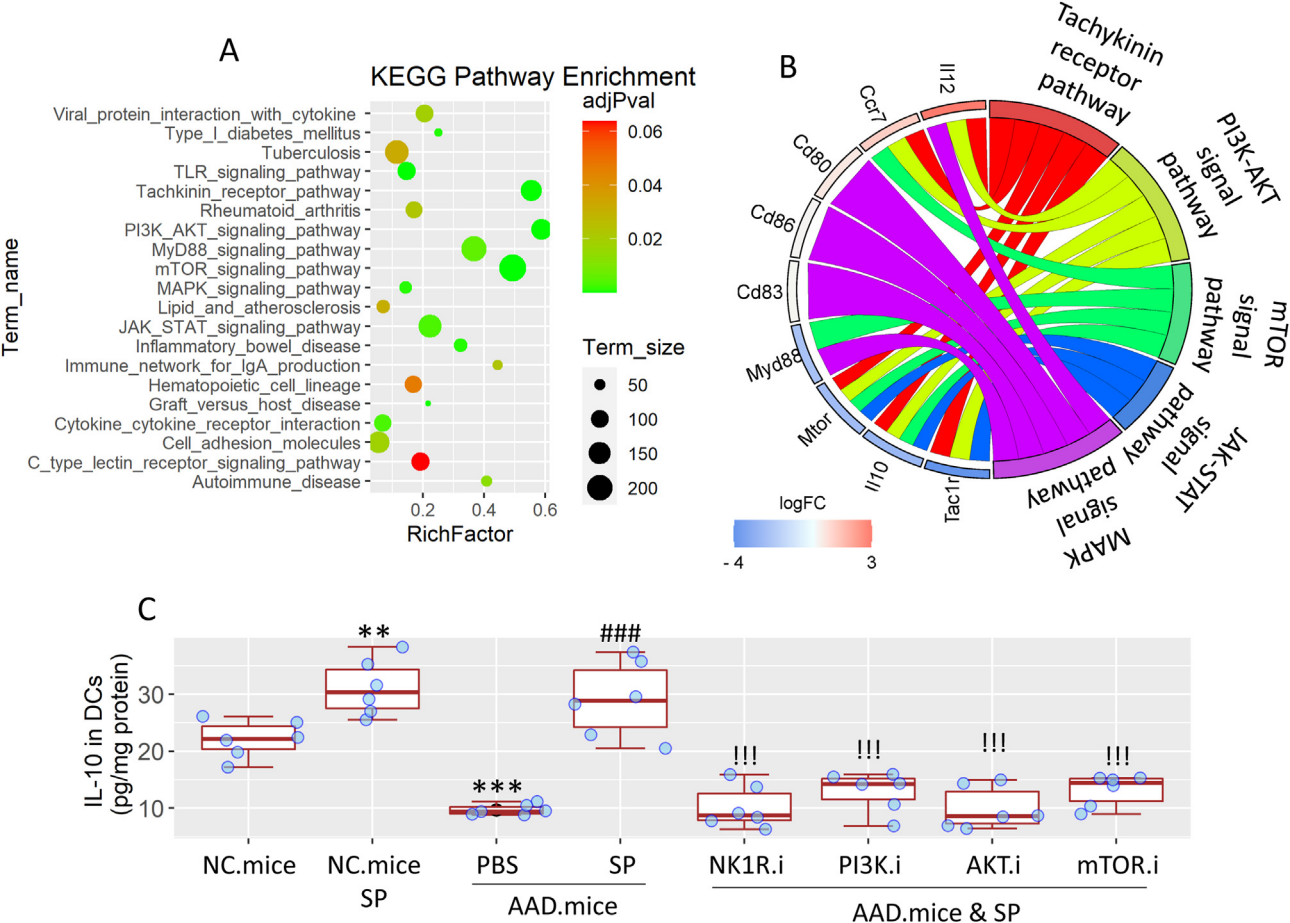
**Assessment of the role of the signal pathway SP-induced IL-10 expression in DCs**. A-B, DCs were isolated from the airway tissues of AAD mice. RNA was extracted from DCs and analyzed by RNAseq. A, signaling pathway enrichment with KEGG database. B, circus chart shows mapping between DEGs and 5 major signaling pathways. C, mice were treated with SP-containing nasal instillation (with or without inhibitors) daily for one week. DCs were isolated from the airway tissues. Proteins were extracted from DCs, and analyzed by ELISA. Boxplots show median (IQR) of IL-10 amounts in protein extracts of DCs from 6 mice per group. Statistics: ANOVA + Dunnett's test. ∗∗ (p < 0.01), ∗∗∗ (p < 0.001), compared with the naïve control (NC) group. ### (p < 0.001), compared with the AAD. mice.PBS group. !!! (p < 0.001), compared with the AAD. mice.SP group. The “.i” in group labels stands for “inhibitors” in nasal instillation: NK1R inhibitor (Rolapitant, 10 nM); PI3K inhibitor (Inavolisib, 5 nM); AKT inhibitor (Akt 1/2, 300 nM); mTOR inhibitor (BEZ23, 20 nM). **Abbreviations**: KEGG: Kyoto Encyclopedia of Genes and Genomes. PI3K: phosphatidylinositide 3-kinases. AKT: Protein kinase B. JAK: Janus kinase. MAPK: Mitogen-activated protein kinase. NK1R: Tachykinin receptor 1. mTOR: Mammalian target of rapamycin. AAD: Airway allergy disorder. SP: Substance P.

We then enriched the signal pathway of the DEGs of AAD DCs with the aid of KEGG database. The most enriched pathways in AAD DCs included the tachykinin receptor signal pathway, the PI3K-AKT signal pathway, and the mTOR signal pathway. The less enriched pathways included the JAK-STAT pathway and MAPK pathway (Fig. 4A). The lower expression of IL-10 may be associated with the lower signaling of JAK-STAT-MAPK pathway.^22^ Using a mapping approach, we found that the tachykinin receptor pathway matched most of the DEGs, including *Il10*, *Mtor*, *Myd88*, *Tac1r* and *Ccr7*. These DEGs were also mapped via the PI3K-AKT pathway. The JAK-STAT pathway weakly mapped to *Il10*, *Tac1r* and *Mtor*. mTOR is an important check point of the signaling pathway of the expression of IL-10. The weak signaling of mTOR linking to the JAK-STAT pathway implicate the expression of IL-10 is impaired.^23^ The MAPK pathway also mapped to *Mtor*, as well as *Ccr7* and *Myd88* (Fig. 4B). The data suggest that the NK1R–PI3K-AKT-mTOR pathway is important in the SP-induced IL-10 expression in DCs. To verify this, we treated naïve mice with nasal instillation (containing SP together with or without inhibitors of NK1R, or PI3K, or AKT, or mTOR daily, for one week. Mice were sacrificed one day after final treatment. DCs were isolated from the airway tissues. Protein extracts were prepared with isolated DCs, and analyzed by ELISA to determine the IL-10 contents. We found application of either of the inhibitors abolished the SP-induced IL-10 expression in airway DCs (Fig. 4C). The results demonstrate that SP can induce the IL-10 production in DCs through the NK1R–PI3K-AKT-mTOR pathway.

#### DCs isolated from AAD mice treated with SP-AIT induce antigen-specific Tr1 cells

**Fig. 5:**
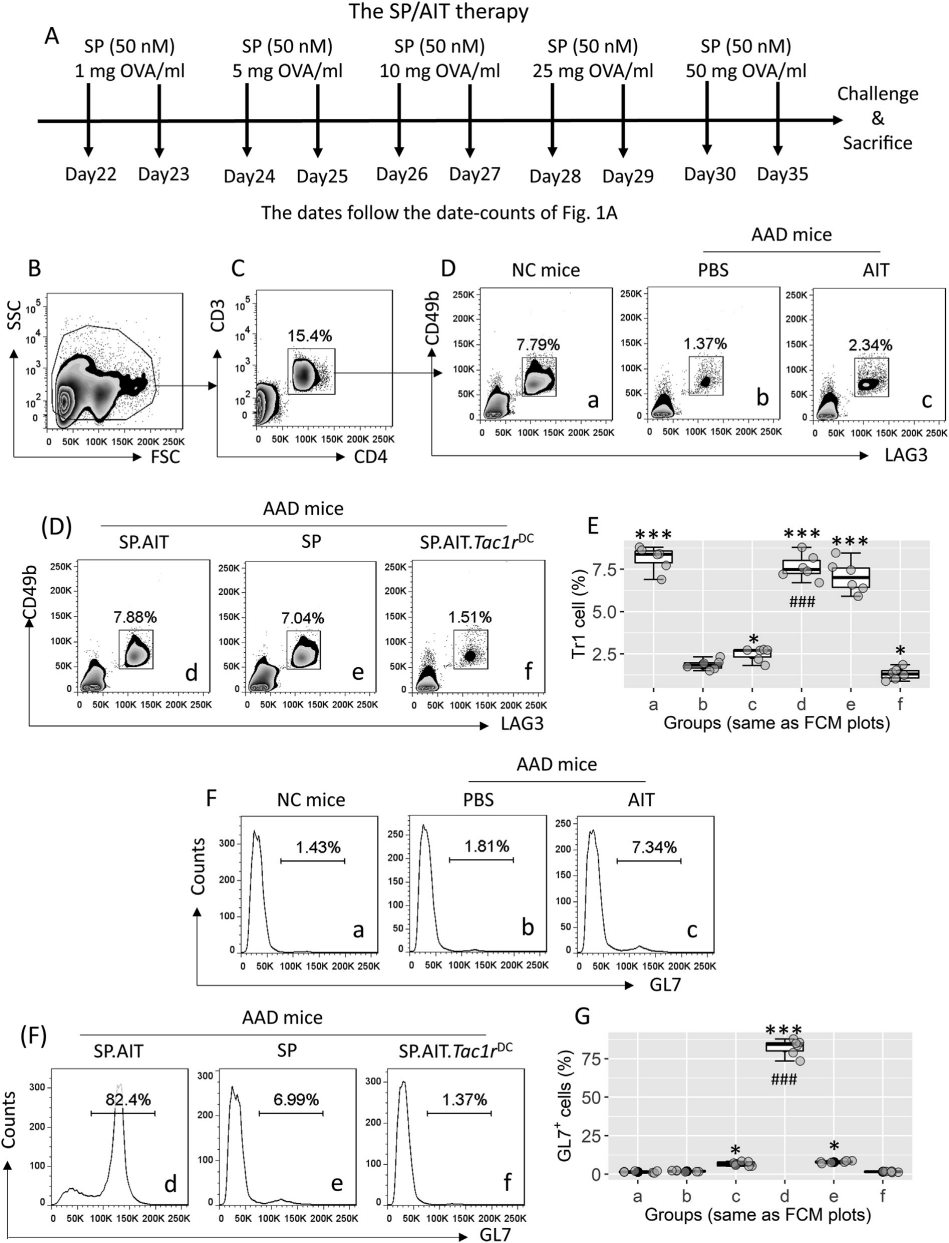
**SP. AIT therapy induces antigen specific Tr1 cells**. A, A schematic of the SP/AAD therapy. AAD mice were treated with AIT or/and SP. After the sacrifice, AMCs were prepared with the airway tissues upon the sacrifice, and analyzed by FCM. B, the FSC/SSC plots. C, CD3^+^ CD4^+^ T cells were gated. D, gated plots show Tr1 cell counts. E, median (IQR) of Tr1 cell frequency in AMCs of 6 mice per group. F, gated histograms show GL7^+^ cells in Tr1 cells of panel D. G, median (IQR) of GL7^+^ Tr1 cells in AMCs of 6 mice per group. Data in FCM plots are from one experiment that represent 6 independent experiments. Statistics: ANOVA + Dunnett's test. ∗ (p < 0.05), ∗∗∗ (p < 0.001), compared with group b. ### (p < 0.001), compared with group c. **Abbreviations**: SP: Substance P. AIT: Allergy specific immunotherapy. Tr1 cell: Type 1 regulatory T cell. AAD: Airway allergy disorder. AMC: Airway mononuclear cell. FCM: Flow cytometry. FSC: Forward scatter. SSC: Side scatter. IQR: Inter Quartile Range.

**Fig. 6:**
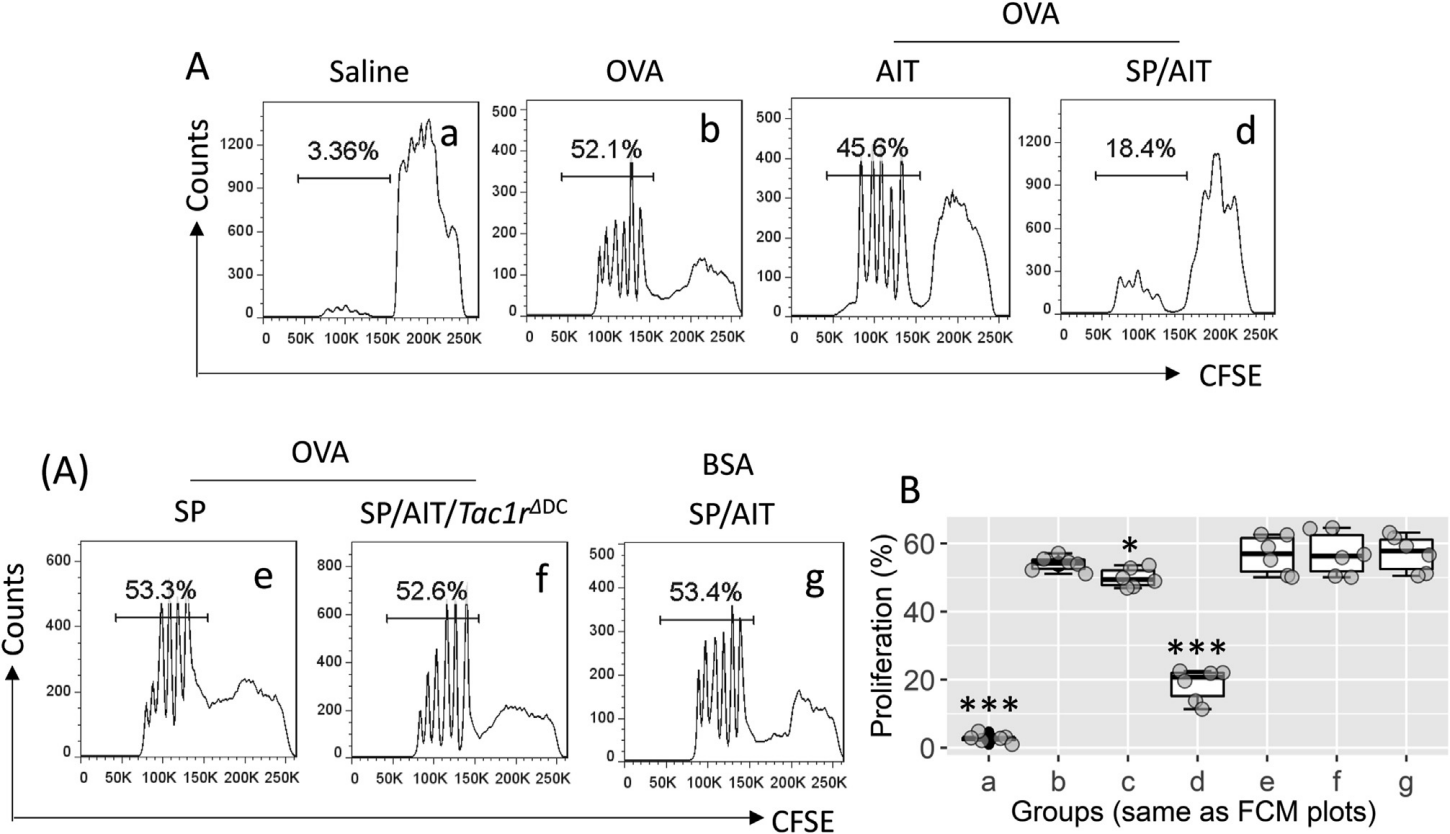
**Assessment of immune suppressive effects of Tr1 cells**. Tr1 cells were isolated from the airway tissues of mice treated with the procedures denoted above each FCM panel. CD4^+^ CD25¯ T cells (Teffs, labeled with CFSE) and DCs were isolated from the DO11.10 mouse spleen. Tr1 cells, Teffs, and DCs were cultured at a ratio of 1:5:1 in the presence of OVA (5 μg/ml; BSA was used as a control) for 3 days. Cells were analyzed by FCM. A, gated histograms show proliferating Teff counts. B, boxplots show median (IQR) of proliferating Teff frequency of 6 independent experiments per group. Statistics: ANOVA + Dunnett's test. ∗ (p < 0.05), ∗∗∗ (p < 0.001), compared with group b. **Abbreviations**: OVA: Ovalbumin. BSA: Bovine serum albumin. SP: Substance P. AIT: Allergen specific immunotherapy. *Tac1r*^ΔDC^: A mouse strain whose DCs are NK1R-deficient. DC: Dendritic cells. FCM: Flow cytometry. IQR: Inter Quartile Range.

It is known that IL-10-producing DCs induce Tr1 cells.^24^ We inferred that DCs isolated from AAD mice treated with the SP-AIT therapy might be able to induce antigen-specific Tr1 cells in AAD mice. To this end, we prepared AMCs from AAD mice treated with or without the SP-AIT therapy. The SP-AIT or SP therapy induced many more LAG-3^+^ CD49b^+^ Tr1 cells in the airway tissues of AAD mice than those treated with AIT alone (Fig. 5A–D). GL7 (a T and B lymphocyte activation marker^25^) was detected in more than 30% Tr1 cells in those treated with SP-AIT. Less than 10% Tr1 cells were detected in those treated with AIT alone. Although exposure to SP could induce Tr1 cells in the respiratory tissues of the AAD mouse, only about 2% of GL7 Tr1 cells were detected (Fig. 5E–F). Furthermore, LAG-3^+^ CD49b^+^ Tr1 cells isolated from the airway tissues of mice treated with the SP-AIT therapy exhibited effective immune suppressive effect on T cell proliferation. Mice treated with AIT alone exhibited much weaker (*P* < 0.001) suppressive effects as compared to those generated by the SP/AIT therapy. Tr1 cells isolated in mice treated only with SP, or from *Tac1r*^ΔDC^ mice treated with SP/AIT, did not show suppressive effects on proliferation of antigen-specific T cells (Fig. 6). The results demonstrate that the SP-AIT therapy can gain much better immune suppressive effect than the AIT alone.

#### SP promotes the therapeutic efficacy of AIT to mitigate AAD response

**Fig. 7:**
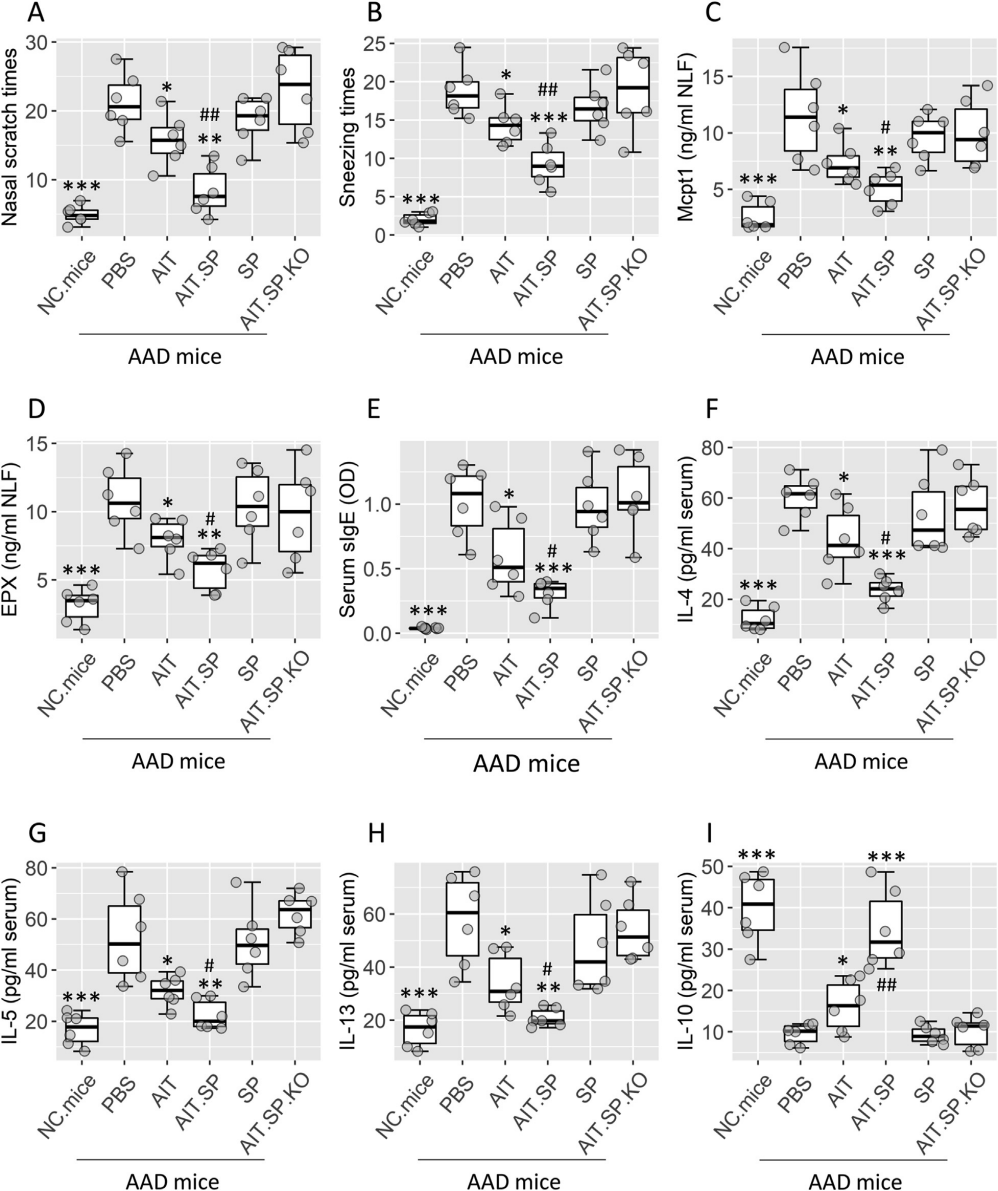
**SP promotes the therapeutic efficacy of AIT for AAD**. AAD mice were treated with AIT or/and SP. A-B, AAD clinical symptom (nasal itch and sneezing) records. C-D, allergic mediators (Mcpt1 and EPX) in NLF. E, serum sIgE levels. F–I, serum Th2 cytokine and IL-10 levels. Boxplots show median (IQR) of AAD parameters from 6 mice per group. Each dot presents data obtained from one sample. Statistics: ANOVA + Dunnett's test. ∗(p < 0.05), ∗∗ (p < 0.01), ∗∗∗ (p < 0.001), compared with the AAD. mice.PBS group (AAD mice were treated with PBS). # (p < 0.05), ## (p < 0.01), compared with the AAD. mice.AIT group. **Abbreviations**: SP: Substance P. AIT: Allergen specific immunotherapy. AAD: Airway allergy disorder. IQR: Inter Quartile Range. PBS: Phosphate-buffered saline. KO: KO mice (*Tac1r*^ΔDC^ mice: A mouse strain whose DCs are NK1R-deficient).

AAD mice were treated with AIT or/and SP as shown in Fig. 5A. In response to OVA (the specific antigen) challenge, AAD mice showed AAD response, including nasal itch (nasal scratches), sneezing (Fig. 7A–B), increased in allergic mediators (Mcpt1 and EPX) in NLF (Fig. 7C–D), increase in sIgE and Th2 cytokines in the serum (Fig. 7E–H) as well as a decrease in serum IL-10 levels (Fig. 7I). AIT therapy moderately (*P* < 0.05) attenuated the AAD response. The combination of AIT and SP achieved a higher therapeutic efficacy than the use of AIT alone. Mice treated with SP alone, or *Tac1r*^ΔDC^ mice treated with SP/AIT, only slightly (p > 0.05) altered the AAD response (Fig. 7).

## Discussion

This paper describes that we have identified a new immune regulator that is highly effective to favor AIT. We found that SP could substantially enhance the therapeutic efficacy of AIT. The combination of AIT and SP through nasal instillation obtained much better effect on mitigating experimental AAD than the treatment of AAD mice with AIT alone. The data also showed that SP increased the expression of IL-10 in DCs. This induces immune regulatory DCs, from which the antigen specific Tr1 cells were subsequently induced. In this experimental setting, the antigen specific Tr1 cells seems are the effective immune regulatory cells in suppressing experimental AAD, further experiments are warranted.

The data demonstrate that the addition of SP is beneficial to AIT. The main objectives of AIT are to induce regulatory immune cells, produce blocker antibodies, such as antigen-specific IgG, and suppress the allergic response.^8^ These effects of AIT were confirmed by the current data. Early studies revealed that SP could induce vasodilation.^26^ Later, it was found that SP had multiple functions in immune responses. However, the majority of observations about the effects of SP focus on its role in induction or facilitation of inflammation. For instance, SP induces epithelial cells to release IL-8; the latter chemoattracts the recruitment of neutrophils. By inducing MIP-1β expression, SP moves lymphocytes to the sites of inflammation to enhance the inflammatory reactions.^27^ By increasing the production of IL-2, SP also facilitate the proliferation of lymphocytes.^28^ Hanf et al observed that SP induced histamine release from nasal mucosa of AR patients,^16^ while Fajac et al did not find histamine release in AR patient nasal cavity after SP challenge, but increased eosinophil infiltration.^17^ SP can promote the efficacy of immunotherapy by enhancing the production of the immune regulatory cytokine IL-10.

We found that administration of SP induced the production of IL-10 in the DCs of AAD mice. DCs are the fraction of cells initiating immune responses. Current data also show that SP regulates DC properties in the first place. Upon exposing to SP, DCs showed an increase in the expression of IL-10. IL-10 is a cytokine that contributes to limiting tissue damage caused by infection and a variety of inflammations.^29^ In allergic reactions, cytokines in allergic lesions released by Th2 cells, mast cells and eosinophils are more than necessary. IL-10 released by SP-primed DCs is expected to suppress the pro-inflammatory activities.^30^ Thus, that SP-induced IL-10 production by DCs is an anti-inflammatory event.

The event of SP-inducing IL-10 expression in DCs was further analyzed by RNAseq. The most active DEGs are those associating the IL-10 expression. The results are verified by the data of signaling pathway enrichment with KEGG database. As shown by RNAseq results, the NK1R–PI3K-AKT signaling pathway was sorted to correspond to the SP-induced IL-10 expression in the DCs. Others also found that the microbial infection induced the IL-10 expression via the TLR/MyD88/NF-κB pathway.^31^ Lipopolysaccharide activating aryl hydrocarbon receptor followed by the activation of Src-STAT pathway in IL-10 expression was reported.^32^ The RNAseq data show different pathway results about the IL-10 expression in DCs, we observed the gene activities of MyD88 was strong. Whether the activities of MyD88 also involve the SP-induced IL-10 expression in DCs is to be further investigated. The above information indicates that the expression of IL-10 can be induced through multiple signal pathways. Our data demonstrate that SP can induce the IL-10 expression in DCs. This may be through the NK1R–PI3K-AKT pathway; further investigation is warranted.

The data show that airway DCs express the NK1R. SP binds NK1R to activate DCs, and thus, induces the IL-10 expression. It is known that T cells also express the NK1R. Thus, it is conceivable that SP can induce Tr1 cells directly. Our data show that the SP-containing nasal instillation does induce Tr1 cells in the airway tissues. However, these Tr1 cells were not activated by AIT. They could not suppress antigen specific T cell proliferation. Thus, it is necessary to combine SP and AIT to induce IL-10^+^ DCs first. These IL-10^+^ DCs, in turn, induce antigen specific Tr1 cells which play immune regulatory role in allergic lesion sites.

The main medical merit of the present study is that we have found that SP favors AIT. AIT is the only etiology-targeted therapy for allergic diseases currently. In allergy clinics, AIT has been employed in the treatment of allergic diseases for many years. It is the consensus that AIT can mitigate allergic diseases. However, the therapeutic effects of AIT were various or even exacerbated the clinical symptoms as reported by different studies.^8^^,^^33^ Although the various results of AIT may be because the heterogeneity in study design and reporting of outcomes,^8^^,^^33^ the fact suggests the therapeutic efficacy of AIT is needed. Our data provide a potential agent, SP, can be used to favor AIT. This needs to be further investigated.

DCs are the cell fraction to initiate immune response. Accumulation of DC in the allergic lesion sites is observed.^34^ Chemokine receptor CCR7 in DCs is associated with the pathogenesis of chronic inflammation, autoimmune diseases, and cancer.^35^ Our data show that the gene activities of CCR7 are higher in DCs of AAD mice. The significance of this event needs to be further investigated.

In summary, current data indicate that SP is a potent immune regulator for AIT. SP in synergy with specific antigen induces IL-10^+^ DCs with the tolerogenic properties. These DCs then induce antigen specific Tr1 cells to play immune regulatory roles in the sites of allergic lesions (see Table 1).

**Table 1:** Primers used in the present study

Genes	Forward	Reverse
*Il10*	Ataactgcacccacttccca	gggcatcacttctaccaggt
*Mtor*	Cgctactgtgtcttggcatc	ggttcatgctgcttagtcgg
*Tac1r*	Acatcttcttcctcctgccc	gctctgggtctggaggtatc
*Cd80*	Ttatcatcctgggcctggtc	gtgtctgcagatgggtttcc
*Cd83*	Gctctcctatgcagtgtcct	actctgtagcttccttgggg
*Cd86*	Gcacgtctaagcaaggtcac	catatgccacacaccatccg
*Ccr7*	Gggctggtgatactgacgta	acacaggtagacgccaaaga
*Il12*	Gacatgtggaatggcgtctc	ttattctgctgccgtgcttc

## Abbreviations

AIT; allergen-specific immunotherapy, SP; substance P, AAD; airway allergy disorder, RNAseq; RNA sequencing, DEG; differentially expressed genes, Tr1 cells; type 1 regulatory T cells, NC; naïve control, PBS; phosphate-buffered saline, BSA; bovine serum albumin, FCM; flow cytometry, OVA; ovalbumin, KO; knockout

## **Funding**

This study was supported by research grants of the 10.13039/501100001809National Natural Science Foundation of China (81870706, 32090052, 81970865), Guangdong Provincial Key Laboratory of Regional Immunity and Diseases (2019B030301009), The High-level University Development Project of 10.13039/501100009019Shenzhen University, 10.13039/501100004791Shenzhen Longgang District Medical & Health Scientific Technology Research Program (LGKCYLWS2020002, LGKCWWS2019000577) and 10.13039/501100004791Shenzhen Key Medical Discipline Construction Fund (No.SZXK039).

## Author contributions

YW, YL, XW, HL, GW, LY and LG performed experiments, analyzed data and reviewed manuscript. QH, XZ and PY supervised experiments. PY designed the project and write the manuscript.

## Consent for publication

All authors have agreed with this publication in the World Allergy Organization Journal.

## Ethics statement

The animal experimental protocol was reviewed and approved by the Animal Ethics Committee at our university (AE2021002).

## Data availability

All the data are included in this paper and the supplemental material.

## Conflict of interest

None to declare.
