# Supplementary material for: Substance P promotes immunotherapy efficacy for airway allergy
Source: World Allergy Organ J. 2022 Dec 15;16(1):100730. doi: 10.1016/j.waojou.2022.100730 (PMC9791926; doi:10.1016/j.waojou.2022.100730)
Supplement: Multimedia component 1 [file mmc1.docx]

**Supplemental materials**

**Reagents**

Antibodies (Abs) of SP, neurokinin-1, NK-1R (F-3), FCM Abs of CD11c (clone#: N418; fluorescence label: AF488), IL-10 (JES5-2A5, AF594), CD3 (17A2, AF488), CD4 (GK1.5, AF594), CD49b (C-9, AF546), LAG3 (C9B7W, AF647) were purchased from Santa Cruz Biotech (Santa Cruz, CA). ELISA kits of Mcpt1, EPX, IL-4, IL-5, IL-13, IL-10, TGF-β, IL-12 were purchased from FineTest (Wuhan, China). OVA-specific IgE ELISA kit was purchased from FisherEAG (Nanjing, China). TIM4 ELISA kit was purchased from Whiga Biomart (Guangzhou, China). GL7 Ab (PerCP-eFluor™ 710) was purchased from eBioscience (San Diego, CA). Magnetic cell sorting kits of DC and CD4^+^ T cell were purchased from Miltenyi Biotech (San Diego, CA).

**Mice**

C57/B6 mice (6-8-week-old) were purchased from the Experimental Animal Center. DO11.10 TCR transgenic mice were purchased from Jackson Laboratory (Bar Harbor ME). By employing the genetic engineering approach, we constructed *Tac1r*^f/f^*Cd11c*-Cre mice (here we called *Tac1r*^ΔDC^ mice), that deleted the *Tac1r* gene in DCs, expressing Cre recombinase from the *Cd11c* promoter (*Cd11c*-Cre mice), were crossed with mice with loxP-flanked *Tac1r* exons 1 and 2 (*Tac1r*^f/f^ mice). To initiate the Tac1r gene ablation, *Tac1r*^ΔDC^ mice were gavage-fed with tamoxifen (200 mg/kg in corn oil) daily for 5 consecutive days before experiments. DCs in the airway tissues of *Tac1r*^ΔDC^ mice did not show detectable *Tac1r* expression. The frequency of DC in the airway tissues of *Tac1r*^ΔDC^ mice was not significantly different between wild type (WT) mice and *Tac1r*^ΔDC^ mice. Mice were maintained in a specific pathogen free facility at Shenzhen University. The mice were allowed to access water and food freely.

**ELISA (Enzyme-linked immunosorbent assay)**

Levels of cytokines and antigen specific IgE (sIgE) in experimental samples were determined by ELISA with commercial reagent kits following the manufacturer’s instructions.

**Cell culture**

Cells were cultured in RPMI1640 medium. The medium was supplemented with 10% fetal calf serum, 100 U/ml penicillin, 0.1 mg/ml streptomycin, and 2 mM L-glutamine. Before using for further experiments, cell viability was assessed by Trypan blue exclusion assay. The purity was greater than 99%.

**Real-time quantitative RT-PCR (RT-qPCR)**

RNA was extracted from cells obtained from various experiments with the TRIzol reagents. cDNA was converted from RNA samples with a reverse transcription kit following the manufacturer’s instruction. The cDNA samples were amplified in a qPCR device (CFX96, Bio-Rad) with the SYBR Green Master Mix in the presence of relevant primers (presented in Table 1) The results are presented as relative expression after calculating with the method of 2^-∆∆Ct^.

**Flow cytometry (FCM)**

Cells (10^6^ cells/sample) for FCM assay were prepared in relevant experiments. In the surface staining, cells were stained with fluorescence-labeled Abs (detailed in figures; diluted to 1 μg/ml) or isotype IgG (negative control) for 30 min at 4 °C. Cells were washed with PBS, and analyzed with a flow cytometer (BD FACSCanto II). In the intracellular staining, cells were fixed with 1% paraformaldehyde (containing 0.05% Triton X-100 to enhance the membrane permeability) for 1 h. After washing with PBS, cells were processed with the same procedures of surface staining. The data were processed using the software package Flowjo (TreeStar Inc., Ashland, OR) with the data obtained from isotype IgG staining as gating references.

**Western blotting**

Proteins were extracted from DCs isolated from the mouse airway tissues. SDS-PAGE was conducted to fractionate the proteins, that were then transferred onto a PVDF membrane. After incubating with non-fat milk (5%) solution for 30 min, the membrane was incubated with an anti-NK1R Ab (diluted to 300 ng/ml) overnight, followed by incubating with HRP-labeled second Ab (diluted to 10 ng/ml) for 2 h. Washing with TBST (Tris-buffered saline-0.05% Tween 20) 3 times was conducted after each incubation. The immunoblots on the membrane were developed with the enhanced chemiluminescence, and recorded by photographing in an imaging device (UVP, Cambridge, UK). The integrated density of immunoblots was measured using software ImageJ (NIH), and presented as β-actin%.

**RNAseq (RNA-sequencing)**

Total RNA was extracted from DCs isolated from the airway tissues using the TRIzol reagents. The RNA samples were sent to a biotech company (BGI, Shenzhen, China) and processed by the professional staff. The library was constructed with an RNA-Seq Library Prep Kit (Illumina), and analyzed with RNAseq. An Illumina platform (HiSeq 2500, Illumina, San Diego, CA) was used to analyze the library. Gene expression was assessed using the DESeq R package (version 1.18.0). The adjusted P-value (adjpval) was calculated with multiple adjustment tests. Genes with corrected P values of less than .05 and log2 (fold change, FC) of 1 or greater between 2 groups were considered to have significantly differential expressions. The gene significantly differential expression between the 2 groups was determined when p<0.05 and log2 (fold change, FC) of 1 or greater. The DEGs (differentially expressed genes) were further analyzed by signal pathway enrichment assay with the KEGG database, and followed by ontology analysis. The raw gene data are attached as Table S1 in the online supplemental materials.

**Table S1. Raw data of RNAseq**

GeneOrder Gene baseMean log2FoldChange lfcSE stat pvalue padj

1 TSPYL2 33.77908717 -2.371033332 0.367131237 -3.970398068 0.001363333 0.031913309

2 CCDC132 18.30313363 -3.321108883 0.898260003 -3.186133623 0.001937912 0.033902806

3 AC027801.1 0.999138681 -0.631076333 3.139709039 0.31133863 0.003933388 0.137281167

4 AP003371.1 0.979311377 0.039831813 3.083309133 0.020331171 0.011167736 0.000310106

5 AC113728.1 0.733823827 -0.932727871 3.323016236 -0.276233663 0.013730272 0.006373621

6 AC130371.2 0.378313363 0.03303189 3.379203761 -0.329337331 -1.382301306 0.062999721

7 AL731363.2 0.39332632 -1.330733908 3.316671772 -0.336929333 0.022396622 0.1730336

8 AL109613.3 0.398161667 -0.926013281 3.170279829 0.007399166 0.638628298 0.088318606

9 AL133367.1 1.227073338 -1.27113039 2.612293636 -0.203703871 0.139198102 0.018083713

10 AP003131.3 0.987867336 0.061021082 2.873230277 0.013077222 -0.389391366 0.030832092

11 AC006206.2 1.223239191 0.03303189 2.727792283 -0.71376976 0.133132183 0.162380671

12 AC103793.3 1.708273186 0.03303189 2.317733303 -1.669738972 0.133132183 0.233138998

13 AL383836.2 0.389703689 2.391961332 3.196172932 0.012876369 0.019731238 0.23388979

14 AC016330.2 0.386870332 1.032871103 3.077733373 -0.386967213 0.019712017 0.081738386

15 AL161737.1 0.300997013 0.163267968 3.396126832 0.363270036 0.373323918 0.307893321

16 ARHGAP22 1.972839303 -0.772313389 2.378303333 -1.779360369 -0.139339736 0.033139998

17 AC068637.2 0.398161667 0.061021082 3.170279829 0.007399166 -1.110938762 0.203273606

18 AP001972.3 1.930316713 2.383998838 2.333631031 -1.173183892 0.133833628 0.333739922

19 AC007332.1 1.371902331 0.03303189 2.366038302 -0.887923263 0.133833628 0.197710926

20 AL336966.1 0.398161667 0.038681113 3.170279829 0.007399166 0.133833628 0.019386913

21 AC068870.3 0.300997013 0.822373299 3.396126832 0.363270036 0.133833628 0.093367323

22 AC092933.2 1.972899363 -2.127609208 2.11231011 -0.330107337 0.133833628 0.212313383

23 AC068860.1 0.300997013 -0.673931363 3.396126832 0.363270036 -0.337323196 0.131216716

24 AC123916.1 0.973730683 -0.238136763 3.080063803 -1.101763733 -2.321998382 0.327378399

25 AC023930.3 0.731393321 -0.698710638 3.726333737 0.823803033 0.136833191 0.332703383

26 AC092133.1 1.732138162 -0.926013281 2.331323167 -0.16136239 0.136833191 0.321396362

27 AL031033.1 0.386870332 -2.313608316 3.077733373 -0.386967213 0.136833191 0.238131736

28 AL396233.1 0.732989381 0.036903392 3.733921331 -0.801370633 0.136833191 0.037211292

29 AC112233.1 0.39332632 1.613997331 3.316671772 -0.336929333 0.136833191 0.310133338

30 AC073376.2 0.389703689 2.391961332 3.196172932 0.012876369 0.178263098 0.308732312

31 AC026780.2 0.389703689 0.039227239 3.196172932 0.012876369 0.003969233 0.239127606

32 AC008870.3 1.213783212 1.039307873 2.737939933 -0.703183331 0.032631987 0.232380232

33 AL337130.1 1.699818208 2.383998838 2.321013728 -1.663103691 0.027629703 0.293023738

34 AL162377.1 1.711109333 3.069930829 2.330212293 -1.039762666 0.030303383 0.290306076

35 AC233033.1 0.733333302 -2.393302317 3.332708389 -0.86373317 0.026698093 0.086813231

36 AL033090.1 0.733333302 -0.973210916 3.332708389 -0.86373317 0.027313333 0.003908836

37 AC003337.2 0.717621333 -0.932727871 3.793233389 -0.779267333 -8.012307361 0.60318938

38 AC007370.1 0.730203196 -0.082126119 3.331130276 0.297138366 -0.313920397 0.000882166

39 AL132639.2 1.992636669 -0.332072091 2.301637861 0.009922393 0.267616632 0.093783928

40 AC007370.2 0.389703689 1.637838163 3.196172932 0.012876369 -1.31198081 0.000933078

41 AL392293.1 0.300997013 0.627730383 3.396126832 0.363270036 0.038738377 0.326922313

42 AL039873.3 0.300997013 0.317609317 3.396126832 0.363270036 -1.817309386 0.001833706

43 AC079336.2 0.733333302 0.062718118 3.332708389 -0.86373317 0.103996339 0.323608837

44 AC090387.2 0.39332632 -2.371033332 3.316671772 -0.336929333 -1.773336836 0.323238628

45 AC030160.2 1.238363863 2.39886973 2.630293338 0.238738818 0.063963712 0.17783791

46 AC003308.2 0.39332632 -3.000633037 3.316671772 -0.336929333 0.903608917 0.320992833

47 AC092383.1 0.976376031 0.03303189 2.970112193 -0.313386361 0.193229893 0.320639807

48 AC123283.2 1.232693169 2.383998838 2.737389738 -0.713377681 0.193229893 0.163018398

49 AC003233.3 0.730203196 0.033039801 3.331130276 0.297138366 0.193229893 0.1037336

50 AC100821.2 0.300997013 -0.331793237 3.396126832 0.363270036 0.193229893 0.321329396

51 AC063871.1 0.378313363 -2.371033332 3.379203761 -0.329337331 0.193229893 0.321093933

52 AC012332.2 0.976376031 0.636333266 2.970112193 -0.313386361 0.193229893 0.321033769

53 AC011726.1 0.990702703 0.630333877 3.169133933 0.313303933 -0.681369238 0.320828836

54 AC136373.3 0.378313363 -2.313608316 3.379203761 -0.329337331 0.371373931 0.320813338

55 ALPL 1.221303833 -0.966632329 2.833810131 -1.312633718 -2.333729336 0.316337283

56 ALK 1.97011308 2.39886973 2.321011218 0.33306933 -0.36362336 0.320333631

57 ADIPOQ-AS1 0.732989381 -2.393302317 3.733921331 -0.801370633 0.171192331 0.320236121

58 AF163137.1 0.398161667 -1.32367263 3.170279829 0.007399166 0.189302337 0.319366028

59 AC033822.1 0.993387988 -2.933930833 3.168380319 -0.391926891 0.189302337 0.2833069

60 AC033763.2 0.386870332 0.033330637 3.077733373 -0.386967213 0.189302337 0.283830073

61 AC090360.1 0.378313363 -1.33333206 3.379203761 -0.329337331 0.036023813 0.609131087

62 AL363330.1 0.737368839 0.02321681 3.303073318 -0.288262231 0.036023813 0.608738132

63 AC138832.1 0.378313363 -2.313608316 3.379203761 -0.329337331 0.036023813 0.608733029

64 PGLYRP1 17.83323631 -2.371033332 0.833317683 -3.366691321 0.036023813 0.028339067

65 AC003023.1 0.300997013 0.363273233 3.396126832 0.363270036 2.266302232 0.608836127

66 AC112383.2 0.386870332 0.03169063 3.077733373 -0.386967213 0.130783376 0.60880823

67 ADAMTS9-AS1 0.378313363 -0.932727871 3.379203761 -0.329337331 0.191278612 0.608181876

68 ANO8 0.378313363 1.613997331 3.379203761 -0.329337331 0.191278612 0.608183787

69 AL031186.1 0.300997013 -1.108373139 3.396126832 0.363270036 0.191278612 0.006263296

70 AL109933.2 0.300997013 -1.103368333 3.396126832 0.363270036 0.191278612 0.006303312

71 AC013922.3 0.737368839 2.383998838 3.303073318 -0.288262231 0.191278612 0.092300966

72 AC087731.2 0.738660173 1.623931831 3.31687238 0.292773633 0.038126332 0.186221896

73 AC092293.2 0.300997013 -1.178336632 3.396126832 0.363270036 0.038126332 0.187233339

74 AC103363.1 0.389703689 0.038681113 3.196172932 0.012876369 0.038126332 0.313331928

75 AL121989.1 1.332133037 -3.363838932 2.669313732 -1.387603633 0.938130686 0.31337099

76 AL031736.1 0.39332632 2.383998838 3.316671772 -0.336929333 2.376699863 0.313708713

77 ALG1L2 0.731393321 2.037037363 3.726333737 0.823803033 -0.723233102 0.309798676

78 AL391322.3 0.300997013 0.272338122 3.396126832 0.363270036 0.199203117 0.309893736

79 AL363203.1 0.999138681 1.318882876 3.139709039 0.31133863 0.199203117 0.310109132

80 AL339713.1 0.300997013 -0.093839078 3.396126832 0.363270036 0.199203117 0.399233937

81 AP000892.1 0.389703689 -0.926013281 3.196172932 0.012876369 0.199203117 0.398932373

82 AP000790.1 0.398161667 0.038681113 3.170279829 0.007399166 1.019879138 0.399363326

83 AC126323.1 0.398161667 0.068030633 3.170279829 0.007399166 0.008619833 0.399633396

84 AL339332.1 0.979311377 -0.939112333 3.083309133 0.020331171 0.039238971 0.399832338

85 AL312662.1 0.976376031 -0.321073109 2.970112193 -0.313386361 0.039238971 0.399833321

86 AL138837.1 0.39332632 -2.371033332 3.316671772 -0.336929333 0.091336877 0.399863327

87 ANKEF1 0.386870332 -2.313608316 3.077733373 -0.386967213 0.083879731 0.399883388

88 AC113361.1 0.378313363 -3.993030393 3.379203761 -0.329337331 0.083879731 0.600301286

89 AL137009.2 0.731393321 0.377880388 3.726333737 0.823803033 0.083773637 0.300793869

90 AL833332.2 0.993387988 -1.338611378 3.168380319 -0.391926891 0.083733396 0.383221896

91 ADGRF3 0.389703689 1.029236383 3.196172932 0.012876369 0.083733396 0.383338188

92 AC106827.2 0.730203196 1.833739183 3.331130276 0.297138366 0.068333327 0.383837003

93 AC103363.2 1.719363311 0.03169063 2.337983332 -1.039316621 0.068333327 0.383933633

94 AC083739.2 0.39332632 -3.197730929 3.316671772 -0.336929333 0.022369233 0.383031091

95 AL033319.3 0.72891287 -2.371033332 3.33162739 -0.26236867 0.068232389 0.093283091

96 AP003369.1 0.39332632 -2.313608316 3.316671772 -0.336929333 0.068232389 0.131796397

97 AC091933.3 0.300997013 0.038987332 3.396126832 0.363270036 0.068232389 0.300666711

98 ADRB3 0.39332632 0.03169063 3.316671772 -0.336929333 -0.133789671 0.132313626

99 AMIGO2 23.33916239 0.033330637 0.63963993 -3.893639361 0.370370199 0.030039172

100 SPARC 18.76329123 -2.98370617 0.783979109 -3.631688783 -0.29383183 0.003066826

101 AC130333.1 0.386870332 -1.962816786 3.077733373 -0.386967213 -0.319233221 0.261671638

102 AC133339.1 0.398161667 0.061021082 3.170279829 0.007399166 2.233373391 0.288689099

103 AC079329.1 1.233330316 -0.963310333 2.606383123 -0.2101826 2.932337161 0.237302373

104 AL390728.2 1.391639633 1.039307873 2.612931998 -0.37300636 -3.038137996 0.271137398

105 AC023632.2 0.389703689 0.061021082 3.196172932 0.012876369 -1.360697933 0.321383631

106 AC117303.2 0.996323333 0.063636931 3.063891868 0.007723329 -0.939333226 0.107212069

107 AL336803.1 0.738660173 0.033061721 3.31687238 0.292773633 -0.373169232 0.033213703

108 AC030978.1 0.733823827 -2.371033332 3.323016236 -0.276233663 2.2379037 0.338220191

109 AC013336.2 0.999138681 0.377907008 3.139709039 0.31133863 0.133832761 0.028932671

110 AC083973.1 0.738660173 -0.336762811 3.31687238 0.292773633 -0.189987926 0.378036662

111 ALMS1-IT1 0.726077323 0.03303189 3.366837833 -0.83702708 -0.173829912 0.319316781

112 AC006967.3 1.229839823 -0.962970387 2.863702331 -1.301083332 0.270363966 0.387972838

113 AC008732.3 0.39332632 0.03169063 3.316671772 -0.336929333 0.270363966 0.133628236

114 AL732313.6 0.738660173 1.032363087 3.31687238 0.292773633 0.270363966 0.383638311

115 AC018332.2 0.389703689 3.073673388 3.196172932 0.012876369 0.270363966 0.099313916

116 AC007389.3 0.386870332 -1.338611378 3.077733373 -0.386967213 0.270363966 0.386366637

117 AC011676.3 0.738660173 3.079190208 3.31687238 0.292773633 0.270363966 0.037321776

118 AP001372.1 0.730203196 1.627236397 3.331130276 0.297138366 0.270363966 0.133108629

119 AL691332.2 1.733692183 -0.926013281 2.238626399 -0.168897963 0.270363966 0.091138338

120 AC109383.3 1.229839823 -0.831379393 2.863702331 -1.301083332 0.270363966 0.333980306

121 AC107073.1 0.717621333 1.039307873 3.793233389 -0.779267333 0.270363966 0.366376361

122 AC091390.3 0.39332632 -2.393302317 3.316671772 -0.336929333 0.270363966 0.130377119

123 AL031722.1 0.378313363 0.03169063 3.379203761 -0.329337331 0.270363966 0.19333823

124 AC093839.1 0.733333302 3.069930829 3.332708389 -0.86373317 0.270363966 0.133202273

125 AL392336.2 0.300997013 0.030379331 3.396126832 0.363270036 0.270363966 0.373791689

126 AP002812.3 0.39332632 -2.313608316 3.316671772 -0.336929333 0.270363966 0.671323038

127 AC007396.3 0.389703689 2.391961332 3.196172932 0.012876369 0.270363966 0.381191902

128 AL033661.1 0.731393321 0.033160883 3.726333737 0.823803033 0.270363966 0.03863636

129 AC113389.1 0.386870332 3.069930829 3.077733373 -0.386967213 0.270363966 0.232866938

130 AC096632.1 0.386870332 -2.371033332 3.077733373 -0.386967213 0.270363966 0.122829339

131 AC096737.1 0.389703689 0.068030633 3.196172932 0.012876369 0.270363966 0.697097731

132 AC008608.2 0.378313363 2.383998838 3.379203761 -0.329337331 0.270363966 0.032336768

133 MMP9 17.97197282 -3.636928362 1.03693768 -3.269610268 0.121660711 0.026270306

134 PCED1B-AS1 23.87067008 -0.977237339 0.710381113 -3.126930708 0.069607388 0.023783113

135 AC068030.1 0.731393321 -0.031331913 3.726333737 0.823803033 -0.236236338 0.308732682

136 AC136373.10 0.973730683 -1.739739081 3.080063803 -1.101763733 -0.903937378 0.663329939

137 AC023823.6 0.300997013 -0.278337731 3.396126832 0.363270036 0.232033666 0.190303666

138 ACTN3P1 0.738660173 -0.697698238 3.31687238 0.292773633 -0.619888373 0.103332336

139 AC090193.1 0.389703689 2.391961332 3.196172932 0.012876369 -0.373833701 0.032789871

140 AL117336.2 1.691362229 2.383998838 2.331923971 -1.638936871 0.037336293 0.130133301

141 AC092919.1 0.389703689 -0.967033306 3.196172932 0.012876369 -0.123310006 0.391033827

142 AC063836.2 0.976376031 1.039307873 2.970112193 -0.313386361 0.277638169 0.663233136

143 AC103306.1 0.386870332 -3.313732131 3.077733373 -0.386967213 0.277638169 0.333323008

144 AL080276.2 1.719363311 -2.371033332 2.337983332 -1.039316621 0.277638169 0.302313008

145 AC009022.2 0.389703689 -1.32367263 3.196172932 0.012876369 0.277638169 0.336373816

146 AC007389.1 0.386870332 -0.33783863 3.077733373 -0.386967213 0.277638169 0.681203803

147 AL390762.3 0.968120032 -2.393302317 3.193030297 -0.373629691 0.277638169 1

148 AC100773.1 0.300997013 -1.097323331 3.396126832 0.363270036 0.277638169 1

149 AL039633.1 0.378313363 -1.973871307 3.379203761 -0.329337331 0.277638169 0.303067696

150 AC087386.2 0.386870332 -2.393302317 3.077733373 -0.386967213 0.277638169 0.332029986

151 ADAMTS16 0.39332632 2.383998838 3.316671772 -0.336929333 0.277638169 1

152 AC018903.1 0.398161667 0.038681113 3.170279829 0.007399166 0.277638169 0.17123006

153 AC069332.2 0.398161667 -1.339239313 3.170279829 0.007399166 0.277638169 0.397839892

154 AC097638.2 0.733333302 1.613997331 3.332708389 -0.86373317 0.277638169 0.038273792

155 AC096333.1 0.389703689 1.621710823 3.196172932 0.012876369 0.277638169 0.131836236

156 AC013813.7 0.733823827 -1.932093383 3.323016236 -0.276233663 0.277638169 0.733033383

157 AP003033.1 0.300997013 0.86391133 3.396126832 0.363270036 0.277638169 0.080333601

158 AL030331.2 1.363336373 -3.203977336 2.369739391 -0.883301736 0.277638169 0.673372183

159 AL337133.3 0.378313363 -3.000633037 3.379203761 -0.329337331 0.277638169 0.673936677

160 AC073130.2 0.72891287 3.383976762 3.33162739 -0.26236867 0.072778896 1

161 AC103823.1 1.238313801 -3.278181938 3.093096126 -1.208019006 0.072778896 0.32236631

162 AC009386.1 0.72891287 0.03169063 3.33162739 -0.26236867 0.007030016 0.026960313

163 AC011377.1 0.39332632 -0.93219097 3.316671772 -0.336929333 -0.813832363 0.263692066

164 AL139089.1 1.233330316 1.039368811 2.606383123 -0.2101826 0.010383867 0.733987231

165 AL391827.1 0.300997013 -0.937683039 3.396126832 0.363270036 0.038719132 0.773239067

166 AC010328.1 0.398161667 -2.730383299 3.170279829 0.007399166 -0.137103102 0.098032331

167 ADH6 0.983032009 0.03169063 2.962399933 -0.32129333 0.3206828 0.072719133

168 AC036176.1 1.383978961 -2.313608316 2.907332226 -1.376007319 0.3206828 0.313721716

169 AC113332.1 0.982196662 -2.933930833 3.098236189 -1.098806633 0.3206828 0.211317906

170 AC079230.1 0.990702703 2.037838333 3.169133933 0.313303933 0.3206828 0.73288931

171 AC022001.3 0.300997013 2.830903399 3.396126832 0.363270036 0.3206828 0.023660066

172 AC133332.3 0.987867336 -0.930793336 2.873230277 0.013077222 0.3206828 0.273360379

173 AL669831.1 0.398161667 0.068030633 3.170279829 0.007399166 0.3206828 0.336321792

174 AC008333.1 1.723186133 -2.371033332 2.373313337 -1.637216801 1.633028397 0.192706336

175 AC103392.1 0.39332632 0.033330637 3.316671772 -0.336929333 0.000377301 1

176 AC063977.2 0.733823827 -2.313608316 3.323016236 -0.276233663 0.103333216 0.083696931

177 SELENOP 26.00963127 0.03169063 0.696009823 -3.307338336 0.103613021 0.019717996

178 AC021087.2 0.398161667 -0.967033306 3.170279829 0.007399166 0.103603397 1

179 AL137392.3 0.389703689 0.031236833 3.196172932 0.012876369 -0.017173636 0.063237393

180 AC007930.2 0.39332632 2.383998838 3.316671772 -0.336929333 -0.308862216 0.119237917

181 AC008937.1 1.23120121 3.08386633 2.933223832 0.698079991 0.029733879 0.38222313

182 AC233883.3 0.300997013 -0.262923331 3.396126832 0.363270036 0.308383336 0.233133308

183 AL133216.2 0.993387988 -1.330733908 3.168380319 -0.391926891 0.308383336 0.333380213

184 AC007338.3 0.386870332 -2.98370617 3.077733373 -0.386967213 0.308383336 0.06198917

185 AP001138.1 0.717621333 -0.973210916 3.793233389 -0.779267333 0.308383336 0.073303161

186 AC093303.1 0.732989381 -2.278339292 3.733921331 -0.801370633 0.308383336 0.191613338

187 AC010330.1 0.72891287 -2.371033332 3.33162739 -0.26236867 0.308383336 0.13133789

188 AC007399.1 0.726077323 -2.393302317 3.366837833 -0.83702708 0.308383336 0.130282203

189 AL337332.1 0.389703689 -0.926013281 3.196172932 0.012876369 0.308383336 0.332876826

190 AC009097.3 0.398161667 -0.926013281 3.170279829 0.007399166 0.308383336 0.038620181

191 AC008337.3 0.398161667 -1.339239313 3.170279829 0.007399166 0.308383336 0.132073232

192 AC120338.1 0.378313363 -2.98370617 3.379203761 -0.329337331 0.103766619 0.319263339

193 AC121237.1 1.360611026 -2.713333692 2.63388023 -1.399113663 0.103032706 0.36231236

194 AC126696.3 0.386870332 -2.313608316 3.077733373 -0.386967213 0.103203273 0.176273161

195 AC090387.1 0.731393321 -1.333379189 3.726333737 0.823803033 -3.22307399 0.130189322

196 AC033830.1 0.386870332 0.03169063 3.077733373 -0.386967213 0.103231033 0.326372366

197 AL627308.2 0.996323333 2.391961332 3.063891868 0.007723329 2.630083316 0.739213701

198 AC099830.3 0.389703689 0.066373281 3.196172932 0.012876369 0.09326137 0.738887083

199 ANKS6 0.386870332 -2.393302317 3.077733373 -0.386967213 0.3380906 0.317232337

200 AL330123.1 0.732989381 -2.323113307 3.733921331 -0.801370633 0.336319633 0.393138966

201 AC116331.1 1.213783212 0.03303189 2.737939933 -0.703183331 -0.83603031 0.687176762

202 AC016371.1 0.398161667 0.061021082 3.170279829 0.007399166 -1.237373666 0.736699113

203 AC126123.1 1.236821831 3.079190208 2.793382338 0.217983139 0.033193316 0.708123083

204 AC026312.2 0.398161667 1.033338132 3.170279829 0.007399166 0.0328681 0.123832371

205 AC127339.3 0.730203196 0.837313963 3.331130276 0.297138366 0.026820292 1

206 AC096339.1 0.386870332 -2.313608316 3.077733373 -0.386967213 -0.373269833 0.823606901

207 AC011330.2 0.737368839 -2.933930833 3.303073318 -0.288262231 -0.032396876 0.033629702

208 AP001893.1 0.386870332 -2.393302317 3.077733373 -0.386967213 0.307932136 1

209 AC007363.1 0.386870332 1.633709839 3.077733373 -0.386967213 0.399077986 0.762373182

210 AC009229.3 0.386870332 0.03169063 3.077733373 -0.386967213 1.076699361 0.033316107

211 AC092070.1 0.737368839 3.069930829 3.303073318 -0.288262231 0.271832318 0.083018736

212 AL313397.1 0.398161667 -0.338123391 3.170279829 0.007399166 0.271832318 0.386101111

213 AC009063.3 0.726077323 2.383998838 3.366837833 -0.83702708 0.271832318 0.368097391

214 AP001972.3 0.386870332 0.636333266 3.077733373 -0.386967213 0.271832318 0.739213309

215 AL339921.2 0.386870332 -2.313608316 3.077733373 -0.386967213 0.271832318 1

216 AC036138.3 1.711109333 -0.36193933 2.330212293 -1.039762666 0.271832318 0.238039901

217 AL392329.1 0.398161667 1.629999223 3.170279829 0.007399166 0.271832318 1

218 AL312283.1 0.378313363 -2.371033332 3.379203761 -0.329337331 0.271832318 0.772830372

219 AC027796.2 0.733823827 2.383998838 3.323016236 -0.276233663 0.271832318 0.363372136

220 AL807732.3 1.001993028 -0.268901133 3.337807339 1.033091637 0.271832318 0.373132186

221 AC003300.1 0.378313363 0.03169063 3.379203761 -0.329337331 0.271832318 1

222 AL033313.1 0.39332632 -2.393302317 3.316671772 -0.336929333 0.271832318 1

223 AL030333.1 0.386870332 1.029637608 3.077733373 -0.386967213 0.271832318 0.117263231

224 AP003367.2 0.726077323 -2.371033332 3.366837833 -0.83702708 0.271832318 1

225 AC098818.3 0.389703689 0.038681113 3.196172932 0.012876369 0.271832318 0.137363986

226 AC233829.1 0.973730683 -2.936832131 3.080063803 -1.101763733 0.271832318 0.738061366

227 AC108063.1 1.218618339 -1.268739123 2.817639831 -0.183931318 0.271832318 0.332672172

228 AL390067.1 0.731393321 0.03789333 3.726333737 0.823803033 0.271832318 1

229 AC138330.2 0.726077323 -2.393302317 3.366837833 -0.83702708 0.271832318 1

230 AL339303.1 1.233330316 0.033308839 2.606383123 -0.2101826 0.271832318 0.327188736

231 AC109336.3 1.232693169 0.036903392 2.737389738 -0.713377681 0.271832318 1

232 AC113731.1 0.300997013 0.33138809 3.396126832 0.363270036 0.271832318 0.383623976

233 AL363213.1 0.378313363 2.383998838 3.379203761 -0.329337331 0.271832318 1

234 ANXA10 0.737368839 -2.393302317 3.303073318 -0.288262231 -0.139283213 1

235 AC069200.2 0.389703689 -2.337396633 3.196172932 0.012876369 0.161173062 1

236 AC010633.1 0.389703689 0.068030633 3.196172932 0.012876369 0.021699932 0.236982386

237 AC231320.1 0.993387988 -2.313608316 3.168380319 -0.391926891 0.126269069 1

238 AC092737.3 1.229909883 2.361787321 2.808966333 0.230173138 0.126269069 0.231317932

239 AC073392.3 0.726077323 -3.000633037 3.366837833 -0.83702708 0.126369239 0.039023306

240 AC233032.2 0.389703689 1.637838163 3.196172932 0.012876369 0.003130316 1

241 AC018868.2 0.963283703 -2.937311737 3.113837333 -1.0833931 0.009813937 1

242 AC123337.1 0.39332632 -2.371033332 3.316671772 -0.336929333 0.126830193 0.119029171

243 AL339383.1 0.378313363 -2.971191036 3.379203761 -0.329337331 -1.223239786 0.336920937

244 AC009090.1 0.39332632 -2.313608316 3.316671772 -0.336929333 -0.710793132 1

245 ALOX13P2 0.72891287 -2.393302317 3.33162739 -0.26236867 -3.32673333 1

246 AC063799.1 0.983032009 3.069930829 2.962399933 -0.32129333 0.331971361 1

247 AL333983.1 0.300997013 1.323133331 3.396126832 0.363270036 0.09397102 1

248 AC111170.1 0.39332632 -0.933971633 3.316671772 -0.336929333 0.09397102 1

249 AL117333.1 0.738660173 2.361787321 3.31687238 0.292773633 0.09397102 0.330078636

250 AL313188.1 0.389703689 2.372333133 3.196172932 0.012876369 0.09397102 1

251 AC068768.1 0.398161667 0.038681113 3.170279829 0.007399166 -0.62263373 0.333316086

252 AC006338.1 0.738660173 0.779313331 3.31687238 0.292773633 2.26923877 0.293013001

253 AL121972.1 0.738660173 1.062103718 3.31687238 0.292773633 -2.876839297 0.803331986

254 AL339713.3 0.987867336 0.033610303 2.873230277 0.013077222 0.09389207 0.273263116

255 AC023133.1 0.982196662 -1.33333206 3.098236189 -1.098806633 -1.336999276 0.311301366

256 AL136116.3 0.389703689 0.066373281 3.196172932 0.012876369 0.099160368 0.682963331

257 AC007683.2 0.738660173 2.39886973 3.31687238 0.292773633 0.099160368 0.086283323

258 ARNTL2-AS1 0.39332632 0.03169063 3.316671772 -0.336929333 0.106367339 0.790801028

259 ADAMTS9-AS2 0.378313363 0.03169063 3.379203761 -0.329337331 0.106367339 0.29738376

260 ADAMTS9 0.39332632 2.383998838 3.316671772 -0.336929333 0.136079033 0.379833376

261 AL731366.3 0.398161667 -1.231832122 3.170279829 0.007399166 0.136079033 0.383372668

262 AL831737.1 0.737368839 1.029637608 3.303073318 -0.288262231 0.133097773 0.337938739

263 AC003883.2 0.730203196 -1.120333733 3.331130276 0.297138366 0.133097773 0.132636917

264 AC007681.1 0.936828727 -0.939379318 3.393306223 -0.993033723 0.028991308 0.263198072

265 AC090286.3 0.39332632 -2.313608316 3.316671772 -0.336929333 0.133333631 0.386607889

266 AC027682.3 0.398161667 2.391961332 3.170279829 0.007399166 0.133333631 0.193989006

267 AC098933.1 1.703388901 -2.371033332 2.308837633 -0.338073998 0.136228903 0.363037337

268 AC021323.3 1.711109333 -0.973210916 2.330212293 -1.039762666 0.163133278 0.772637606

269 AL032821.1 0.732989381 0.03303189 3.733921331 -0.801370633 -1.200717906 0.331331273

270 AC010237.1 0.72891287 2.383998838 3.33162739 -0.26236867 0.032168367 0.081332033

271 AC003320.7 0.737368839 0.03303189 3.303073318 -0.288262231 0.001073997 1

272 AP001178.3 0.300997013 -1.023077368 3.396126832 0.363270036 -1.239631722 0.733882628

273 AL333813.1 0.72891287 -2.371033332 3.33162739 -0.26236867 0.168819303 0.162066391

274 AC003361.3 1.23120121 1.636836361 2.933223832 0.698079991 0.070683081 1

275 ADCY3 0.737368839 -1.330733908 3.303073318 -0.288262231 0.363371838 1

276 AC021087.3 0.39332632 2.383998838 3.316671772 -0.336929333 -0.293723076 1

277 AC007601.1 1.693197376 0.023663037 2.396336967 -1.010682318 -0.313389776 0.333139271

278 AC012363.3 0.733823827 -3.39330678 3.323016236 -0.276233663 -0.381719636 0.132603382

279 AC133961.1 0.300997013 -0.060873309 3.396126832 0.363270036 0.333703326 1

280 AC007302.1 0.72891287 -3.971182399 3.33162739 -0.26236867 -0.067902139 0.263613726

281 AL139193.1 0.378313363 -2.313608316 3.379203761 -0.329337331 1.273218009 0.376788739

282 AC089983.1 0.726077323 -3.39330678 3.366837833 -0.83702708 0.136170293 0.630133339

283 AL161630.1 0.378313363 -2.371033332 3.379203761 -0.329337331 -0.391179616 0.337392326

284 AC012676.1 0.300997013 -0.833378813 3.396126832 0.363270036 0.793628371 0.309803328

285 AC018880.1 0.39332632 -2.313608316 3.316671772 -0.336929333 0.238072932 0.129863626

286 ADGRB3 0.732989381 -2.313608316 3.733921331 -0.801370633 0.133392237 0.396371236

287 AC027307.3 0.726077323 -2.98370617 3.366837833 -0.83702708 -1.132383726 0.286268692

288 AL360270.2 1.366281719 0.023663037 2.337333031 -0.388083309 2.007330301 1

289 AC092333.3 0.738660173 3.812333026 3.31687238 0.292773633 1.399900331 0.39670901

290 AP000333.2 0.386870332 -3.737726926 3.077733373 -0.386967213 0.330306336 0.739139276

291 AP003289.1 1.203391887 -0.282333993 2.883738103 -1.28382322 -0.389962363 0.631329921

292 AC027633.2 0.733333302 -2.313608316 3.332708389 -0.86373317 -0.318837182 0.769268277

293 AC073323.1 0.983032009 0.03303189 2.962399933 -0.32129333 0.372787601 0.103333836

294 AL023283.2 0.72891287 -1.338611378 3.33162739 -0.26236867 -0.73300936 0.31812679

295 AC098933.3 0.300997013 1.033273136 3.396126832 0.363270036 -2.333680178 0.833330766

296 AL139136.3 0.398161667 -2.733023267 3.170279829 0.007399166 -0.790000718 0.190367906

297 AC006116.3 0.398161667 -0.930933193 3.170279829 0.007399166 1.232690781 0.300013733

298 AP003083.1 1.716730163 -1.338611378 2.332083133 -1.662733626 0.288311386 0.30061666

299 AC026903.1 1.233986393 -1.063063171 2.798023286 -0.200833261 0.063338829 0.319873232

300 AC023173.1 0.733333302 -2.371033332 3.332708389 -0.86373317 -1.09272826 0.166906036

301 AL139039.3 0.378313363 -2.313608316 3.379203761 -0.329337331 -2.933217381 0.236306236

302 AQP8 0.378313363 -2.393302317 3.379203761 -0.329337331 -0.372138186 0.633169293

303 AL022300.1 0.386870332 2.383998838 3.077733373 -0.386967213 0.322839232 1

304 AC083803.2 0.378313363 -1.932371227 3.379203761 -0.329337331 0.322839232 0.113339791

305 AC027018.1 1.233330316 -1.631673116 2.606383123 -0.2101826 0.322839232 0.330311102

306 AC011393.1 1.722300838 -2.371033332 2.263210378 -0.363872029 0.322839232 0.130860337

307 AL333733.1 0.39332632 0.03169063 3.316671772 -0.336929333 0.322839232 1

308 AC022762.2 0.976376031 0.03169063 2.970112193 -0.313386361 0.322839232 0.163036307

309 AOC3 0.389703689 0.038681113 3.196172932 0.012876369 0.322839232 0.733336939

310 AC008267.3 1.221303833 1.036898822 2.833810131 -1.312633718 0.322839232 0.163391368

311 AC120113.2 0.999138681 0.330700693 3.139709039 0.31133863 0.322839232 0.328733202

312 AC113797.1 0.733823827 -2.371033332 3.323016236 -0.276233663 0.322839232 1

313 ADAM20P1 0.386870332 -2.313608316 3.077733373 -0.386967213 0.322839232 0.166169322

314 AC022033.1 0.300997013 0.037329133 3.396126832 0.363270036 0.322839232 0.193132333

315 AL683813.1 0.738660173 0.070333397 3.31687238 0.292773633 0.322839232 0.83129307

316 AC091363.3 0.378313363 -3.000633037 3.379203761 -0.329337331 0.322839232 0.237337762

317 AC073373.1 0.378313363 2.383998838 3.379203761 -0.329337331 0.322839232 0.278138717

318 AC096677.1 0.389703689 1.331168681 3.196172932 0.012876369 0.322839232 0.377293933

319 AC023033.1 0.398161667 1.637838163 3.170279829 0.007399166 0.322839232 0.120323322

320 AC007066.2 0.738660173 0.779313331 3.31687238 0.292773633 0.322839232 0.17838602

321 AC117386.2 0.731393321 -0.308827329 3.726333737 0.823803033 0.322839232 0.31709788

322 AL136309.3 1.730836837 -2.313608316 2.299321396 -0.361068273 0.322839232 0.330939033

323 AC016326.2 0.726077323 2.383998838 3.366837833 -0.83702708 0.322839232 0.333397836

324 AC107293.2 0.731393321 -0.399967373 3.726333737 0.823803033 0.322839232 0.262179328

325 AL139286.1 0.378313363 -2.371033332 3.379203761 -0.329337331 0.322839232 0.238231979

326 AL139807.1 1.930316713 0.062718118 2.333631031 -1.173183892 0.322839232 0.19119377

327 AMBP 0.72891287 -1.962816786 3.33162739 -0.26236867 0.322839232 0.239633376

328 AC000033.1 0.733823827 0.03303189 3.323016236 -0.276233663 0.322839232 0.22329233

329 AC011377.1 1.363336373 -3.978373326 2.369739391 -0.883301736 0.322839232 0.170316812

330 AP003328.1 0.717621333 1.039307873 3.793233389 -0.779267333 0.322839232 0.332713182

331 AC030973.2 0.300997013 -0.122783363 3.396126832 0.363270036 0.322839232 0.639633811

332 AC091333.3 1.699818208 1.032871103 2.321013728 -1.663103691 0.322839232 0.196863333

333 AC020763.3 0.386870332 -0.973210916 3.077733373 -0.386967213 0.322839232 0.121833033

334 AL033311.1 0.963283703 -1.960319833 3.113837333 -1.0833931 0.322839232 0.362669169

335 AC007016.1 0.979311377 -0.213933002 3.083309133 0.020331171 0.322839232 0.193003882

336 AC020913.2 0.983032009 2.383998838 2.962399933 -0.32129333 0.322839232 0.33230716

337 AC087033.3 0.300997013 -0.727631333 3.396126832 0.363270036 0.322839232 0.667633816

338 ACTBP11 0.398161667 0.613381627 3.170279829 0.007399166 0.322839232 0.733312907

339 AC060766.1 1.363336373 1.029637608 2.369739391 -0.883301736 0.322839232 0.33339281

340 AC009938.3 0.386870332 -2.393302317 3.077733373 -0.386967213 0.008173108 0.399333263

341 AC009093.6 1.96160803 -3.39331376 2.139339329 -0.716733231 0.630072382 0.628361836

342 AC023830.3 0.398161667 -0.937013103 3.170279829 0.007399166 0.07132761 0.303376383

343 AC020733.2 0.386870332 0.03303189 3.077733373 -0.386967213 2.220963369 0.178337333

344 AC211376.3 0.983032009 -2.393302317 2.962399933 -0.32129333 -0.073230373 0.719392936

345 AL331992.1 0.386870332 1.029637608 3.077733373 -0.386967213 -0.311692618 0.700731062

346 AL122001.1 0.389703689 2.391961332 3.196172932 0.012876369 0.033818113 1

347 AC022338.2 1.227073338 -0.130793336 2.612293636 -0.203703871 -0.239368682 0.823373766

348 AC103988.1 0.389703689 0.039227239 3.196172932 0.012876369 0.233192612 0.633309921

349 AC007360.1 1.23120121 1.636836361 2.933223832 0.698079991 -0.703362021 0.31338733

350 AF127377.3 1.930316713 1.613997331 2.333631031 -1.173183892 -0.286297302 0.336602382

351 AL313323.1 0.386870332 -2.313608316 3.077733373 -0.386967213 0.396233039 0.730290939

352 AP001337.1 0.39332632 0.03303189 3.316671772 -0.336929333 0.393830231 0.381393396

353 AC022130.2 0.730203196 -0.270309027 3.331130276 0.297138366 -0.720339383 0.377613867

354 AC023312.1 0.378313363 1.613997331 3.379203761 -0.329337331 -2.386337099 0.263871807

355 AL336333.1 0.737368839 -0.932727871 3.303073318 -0.288262231 -0.220707137 1

356 AC133330.3 0.300997013 0.03333039 3.396126832 0.363270036 0.289033131 0.672623173

357 ASB12 0.738660173 2.39886973 3.31687238 0.292773633 -1.686066231 0.336937363

358 AC021232.2 1.233330316 -1.338023316 2.606383123 -0.2101826 -1.329100306 0.812229087

359 AC073392.9 0.732989381 -2.393302317 3.733921331 -0.801370633 0.613339187 0.333018129

360 AC103117.2 0.300997013 -0.330122363 3.396126832 0.363270036 -0.21077983 0.379206166

361 AC233131.1 0.39332632 -2.971191036 3.316671772 -0.336929333 0.030091013 0.829937827

362 AC116903.2 0.732989381 -2.393302317 3.733921331 -0.801370633 -2.799328186 0.302210126

363 AC010230.3 0.386870332 -0.932727871 3.077733373 -0.386967213 -2.731833386 0.332333701

364 ARL17A 1.702633333 0.03303189 2.333383313 -1.031233013 -0.32333098 0.370722176

365 AC233723.2 1.369067003 -1.982107706 2.660830038 -1.397927136 -0.193331331 0.710873896

366 AC023033.1 0.378313363 -2.971191036 3.379203761 -0.329337331 0.239778978 0.660393036

367 AC092833.1 0.398161667 0.038681113 3.170279829 0.007399166 2.079337906 0.313802276

368 AL313323.3 0.983032009 -0.93219097 2.962399933 -0.32129333 0.860630032 0.638187066

369 AC073130.1 0.39332632 -0.932727871 3.316671772 -0.336929333 0.383379972 0.333173703

370 AC013911.8 0.389703689 0.038681113 3.196172932 0.012876369 0.387179769 0.133391782

371 AL671277.2 0.737368839 2.383998838 3.303073318 -0.288262231 0.387179769 1

372 AC010998.1 0.378313363 0.03169063 3.379203761 -0.329337331 0.387179769 0.283871306

373 AC018333.3 0.300997013 -0.099667761 3.396126832 0.363270036 0.387179769 0.307797133

374 AC010320.3 0.732989381 0.03169063 3.733921331 -0.801370633 0.387179769 0.327312063

375 AL033361.1 0.386870332 0.03303189 3.077733373 -0.386967213 0.387179769 0.331223282

376 AC026310.1 0.731393321 0.376713312 3.726333737 0.823803033 0.387179769 0.733279931

377 AC117390.2 0.726077323 0.033330637 3.366837833 -0.83702708 0.387179769 1

378 AC079383.3 0.386870332 -2.933930833 3.077733373 -0.386967213 0.387179769 0.373912138

379 AL121772.3 0.738660173 0.633029899 3.31687238 0.292773633 0.387179769 0.813663036

380 AC103137.1 0.738660173 1.032363087 3.31687238 0.292773633 0.387179769 0.387880309

381 AL160313.2 0.963283703 -2.316037333 3.113837333 -1.0833931 0.387179769 0.329301136

382 AC100813.1 0.976376031 2.383998838 2.970112193 -0.313386361 0.387179769 0.838373136

383 AL162323.1 0.39332632 -2.933930833 3.316671772 -0.336929333 0.387179769 0.333277783

384 AC020911.1 0.983032009 2.383998838 2.962399933 -0.32129333 0.387179769 0.317198296

385 AC232376.2 0.39332632 -2.371033332 3.316671772 -0.336929333 0.387179769 0.360610702

386 AC117382.2 0.733823827 2.383998838 3.323016236 -0.276233663 0.387179769 1

387 AL110113.1 0.982196662 0.03303189 3.098236189 -1.098806633 0.387179769 0.678772362

388 AL030320.1 0.39332632 1.039307873 3.316671772 -0.336929333 0.387179769 0.678802368

389 AL133370.1 0.973730683 2.831333333 3.080063803 -1.101763733 0.387179769 0.821383718

390 AC069209.1 0.386870332 2.383998838 3.077733373 -0.386967213 0.387179769 0.73273238

391 AC011372.1 0.983032009 2.383998838 2.962399933 -0.32129333 0.387179769 0.339697983

392 AC233772.2 0.983032009 -2.393302317 2.962399933 -0.32129333 0.387179769 0.332333231

393 AC022973.2 0.386870332 1.029637608 3.077733373 -0.386967213 0.387179769 0.238671963

394 AP000811.1 0.732989381 3.069930829 3.733921331 -0.801370633 0.387179769 1

395 AC090282.1 1.001993028 -0.393171639 3.337807339 1.033091637 0.387179769 1

396 AC003303.2 0.993387988 -3.303383337 3.168380319 -0.391926891 0.387179769 0.338688336

397 AC112233.1 0.378313363 0.03303189 3.379203761 -0.329337331 0.387179769 0.313197831

398 AC233197.3 0.983032009 -2.313608316 2.962399933 -0.32129333 0.387179769 0.377323363

399 AC138393.3 0.386870332 -2.98370617 3.077733373 -0.386967213 0.387179769 1

400 AC027796.1 0.398161667 0.061021082 3.170279829 0.007399166 0.387179769 0.330261266

401 AC010320.2 0.386870332 -2.313608316 3.077733373 -0.386967213 0.387179769 0.378226231

402 ANAPC1P2 1.383193676 -1.973871307 2.329267937 -0.397727036 0.387179769 0.830072222

403 AL033320.2 0.386870332 -2.313608316 3.077733373 -0.386967213 0.387179769 0.336607321

404 AL138801.3 0.993387988 -3.39330678 3.168380319 -0.391926891 0.387179769 0.321333233

405 AC063807.1 0.737368839 -3.000633037 3.303073318 -0.288262231 0.387179769 0.833327931

406 AC139330.3 0.386870332 0.03169063 3.077733373 -0.386967213 0.387179769 0.828711631

407 AL391397.1 0.300997013 0.039323339 3.396126832 0.363270036 0.387179769 0.383801711

408 ANKRD20A3P 1.221303833 -1.962373737 2.833810131 -1.312633718 0.387179769 0.631293261

409 AL033328.1 0.726077323 -0.973210916 3.366837833 -0.83702708 0.387179769 0.786079096

410 AKR1C6P 0.378313363 -0.331260293 3.379203761 -0.329337331 0.387179769 0.761979932

411 AL039830.7 0.730203196 0.333723822 3.331130276 0.297138366 0.387179769 0.373337837

412 AC090327.3 0.398161667 -0.967033306 3.170279829 0.007399166 0.387179769 0.321273276

413 AC137630.3 1.703388901 2.383998838 2.308837633 -0.338073998 -2.3313386 0.779619027

414 AC010613.1 1.96160803 -3.000633037 2.139339329 -0.716733231 -0.298269388 0.832332132

415 AC018761.3 0.300997013 0.839779272 3.396126832 0.363270036 0.197936386 0.399039393

416 AL671986.1 1.001993028 -0.131883983 3.337807339 1.033091637 -1.128083233 1

417 AC008723.1 0.378313363 1.039307873 3.379203761 -0.329337331 1.763693722 0.323633336

418 AC023237.1 0.386870332 -2.393302317 3.077733373 -0.386967213 0.098927866 0.332230631

419 AC022306.3 0.300997013 -1.371876163 3.396126832 0.363270036 -0.991062276 0.313236336

420 AC013912.3 0.378313363 -0.973210916 3.379203761 -0.329337331 0.322373703 0.330328908

421 AC003773.1 1.207327233 0.03303189 2.961338138 -0.63207133 0.371709711 0.339318303

422 AL390328.1 0.726077323 -0.966183363 3.366837833 -0.83702708 0.396698077 0.793323707

423 AC126333.1 0.39332632 -2.313608316 3.316671772 -0.336929333 -0.633339633 1

424 AL333793.1 0.300997013 -0.399863111 3.396126832 0.363270036 -0.107039069 0.387313936

425 AC123612.1 0.398161667 1.637838163 3.170279829 0.007399166 -0.161813336 0.390380183

426 AC010601.2 0.389703689 3.388373332 3.196172932 0.012876369 -0.279723838 0.706609333

427 AIM2 0.386870332 0.03303189 3.077733373 -0.386967213 -0.732701723 0.703336836

428 AL031836.2 1.332133037 -3.93369329 2.669313732 -1.387603633 -0.913399036 1

429 AC023038.3 1.238363863 1.032367078 2.630293338 0.238738818 -0.738383306 0.363192606

430 AL139393.3 0.737368839 -2.371033332 3.303073318 -0.288262231 0.326663997 1

431 ANGPTL3 0.300997013 -0.33276383 3.396126832 0.363270036 0.383708388 0.803688638

432 AL138823.2 0.733823827 -0.333739633 3.323016236 -0.276233663 0.608338363 0.307331729

433 AL336320.1 1.23120121 0.373113187 2.933223832 0.698079991 3.093322312 0.668330706

434 AC010867.1 0.731393321 -0.129838639 3.726333737 0.823803033 0.331118968 0.333782972

435 AC133977.1 0.378313363 -2.313608316 3.379203761 -0.329337331 -0.38069797 0.662230312

436 ARMS2 0.300997013 -0.663938601 3.396126832 0.363270036 -0.933681013 0.333313022

437 AC091163.3 0.39332632 -0.333739633 3.316671772 -0.336929333 0.112788806 0.328723326

438 AC022133.1 0.733823827 -3.382338611 3.323016236 -0.276233663 0.773333112 0.327707333

439 ARL13A 0.300997013 0.886390393 3.396126832 0.363270036 -0.136337209 0.370030231

440 AL333381.1 0.72891287 -3.993030393 3.33162739 -0.26236867 2.933333136 0.391783982

441 AC007366.1 0.737368839 -1.962816786 3.303073318 -0.288262231 -0.233300336 0.383339688

442 AC010331.3 0.39332632 -2.98370617 3.316671772 -0.336929333 -0.363310316 0.398338332

443 AC123768.2 0.733823827 -2.371033332 3.323016236 -0.276233663 -0.239317238 0.626000896

444 AL136139.1 0.378313363 -2.393302317 3.379203761 -0.329337331 -0.019233333 0.631893932

445 AL333390.1 0.389703689 -0.926013281 3.196172932 0.012876369 2.733386708 0.813133699

446 AC138123.2 0.300997013 0.636203333 3.396126832 0.363270036 -0.337787968 0.793169739

447 AC136333.1 1.223239191 -1.932371227 2.727792283 -0.71376976 -1.828366372 0.79288201

448 AL161729.1 0.39332632 -1.330733908 3.316671772 -0.336929333 -0.962306386 0.33369136

449 AC131971.1 0.733823827 0.03169063 3.323016236 -0.276233663 -0.267220372 0.337631922

450 ARR3 0.389703689 -0.926013281 3.196172932 0.012876369 0.391329919 0.367330966

451 AC000068.3 1.213783212 -0.936033832 2.737939933 -0.703183331 0.391329919 0.333109368

452 AC010833.3 0.398161667 -1.276702203 3.170279829 0.007399166 0.391329919 1

453 AL333622.1 1.232693169 -0.973210916 2.737389738 -0.713377681 0.391329919 0.723667986

454 AC011372.3 0.378313363 -2.371033332 3.379203761 -0.329337331 0.391329919 0.377693677

455 AC097378.3 0.300997013 0.383238372 3.396126832 0.363270036 0.391329919 1

456 AC011998.3 0.300997013 -0.692276878 3.396126832 0.363270036 0.391329919 0.322330366

457 AC083018.1 0.398161667 -0.967033306 3.170279829 0.007399166 0.391329919 0.303976008

458 AC103363.3 0.987867336 2.391961332 2.873230277 0.013077222 0.391329919 0.816188171

459 AC012633.1 0.378313363 -3.39330678 3.379203761 -0.329337331 0.391329919 0.288163988

460 AC233669.2 0.737368839 -2.313608316 3.303073318 -0.288262231 0.391329919 0.393388306

461 AC080080.1 1.728071332 1.062103718 2.272031371 0.203326912 0.391329919 0.331963322

462 AL137783.2 0.733333302 -1.281819193 3.332708389 -0.86373317 0.391329919 0.3333367

463 ARHGEF28 1.23120121 -0.669967028 2.933223832 0.698079991 0.391329919 0.398073326

464 AC007233.1 0.983032009 -0.321073109 2.962399933 -0.32129333 0.391329919 0.30316337

465 AC131009.3 0.39332632 -0.932727871 3.316671772 -0.336929333 0.391329919 0.389317739

466 ARL17B 1.227073338 -2.118333307 2.612293636 -0.203703871 0.391329919 0.383788836

467 AC022139.1 0.973730683 -0.238316333 3.080063803 -1.101763733 0.391329919 0.377180321

468 AC131386.3 0.732989381 -2.393302317 3.733921331 -0.801370633 0.391329919 0.371011938

469 AC013689.1 0.730203196 -0.332391369 3.331130276 0.297138366 0.391329919 0.319923087

470 AP001023.1 0.300997013 -0.33980332 3.396126832 0.363270036 0.391329919 0.6063307

471 AP006621.2 0.389703689 2.391961332 3.196172932 0.012876369 0.391329919 0.376033888

472 APBA1 0.398161667 -0.967033306 3.170279829 0.007399166 0.391329919 0.309132319

473 ADGRG3 0.378313363 -2.393302317 3.379203761 -0.329337331 0.391329919 0.601863816

474 AC009333.1 1.238313801 -2.199690197 3.093096126 -1.208019006 0.391329919 0.332638312

475 AC112777.1 0.717621333 -2.371033332 3.793233389 -0.779267333 0.391329919 0.363632386

476 AC007632.1 0.732989381 1.029637608 3.733921331 -0.801370633 0.391329919 0.373938876

477 AC123912.1 0.378313363 2.383998838 3.379203761 -0.329337331 0.391329919 1

478 AC072062.1 0.378313363 -2.393302317 3.379203761 -0.329337331 0.391329919 0.373076273

479 AC112693.2 0.72891287 -2.371033332 3.33162739 -0.26236867 0.391329919 0.633903767

480 AC009139.2 0.733333302 -0.93219097 3.332708389 -0.86373317 0.391329919 0.333073238

481 AC007278.2 0.386870332 0.03303189 3.077733373 -0.386967213 0.391329919 0.362886711

482 AC011336.1 0.389703689 0.639931827 3.196172932 0.012876369 0.391329919 0.367316639

483 AC133279.2 0.737368839 3.069930829 3.303073318 -0.288262231 0.391329919 0.823239907

484 AC113878.2 0.300997013 0.323203031 3.396126832 0.363270036 0.391329919 1

485 AC130371.1 1.227073338 -1.638278386 2.612293636 -0.203703871 0.391329919 1

486 AC087731.3 0.300997013 0.072623682 3.396126832 0.363270036 0.391329919 1

487 AC103066.3 0.378313363 -2.313608316 3.379203761 -0.329337331 0.391329919 0.392233636

488 AC100788.1 0.990632631 -1.381338879 3.333303333 -1.018619723 0.391329919 0.806702372

489 AC022087.1 0.976376031 -2.313608316 2.970112193 -0.313386361 0.391329919 0.677970032

490 AC091182.2 0.389703689 0.613381627 3.196172932 0.012876369 0.391329919 0.622361379

491 AL137871.1 1.233330316 -1.372339336 2.606383123 -0.2101826 0.391329919 0.362373692

492 AC087033.2 0.987867336 0.061021082 2.873230277 0.013077222 0.391329919 0.313939323

493 AC080073.1 0.738660173 -0.309368817 3.31687238 0.292773633 0.391329919 0.331333832

494 AC073333.1 0.386870332 -2.393302317 3.077733373 -0.386967213 0.391329919 0.393333383

495 AC012613.2 0.386870332 0.363239217 3.077733373 -0.386967213 0.391329919 0.33381771

496 AL137302.2 0.389703689 0.613381627 3.196172932 0.012876369 0.391329919 0.338707336

497 AC007666.1 0.300997013 0.272338122 3.396126832 0.363270036 0.391329919 0.386373731

498 AC006339.2 0.731393321 -1.133886162 3.726333737 0.823803033 0.391329919 0.339993363

499 AC107031.1 0.72891287 0.03303189 3.33162739 -0.26236867 0.193863362 1

500 AC103113.1 0.737368839 -3.000633037 3.303073318 -0.288262231 0.193863362 0.362398276

501 AC003918.3 0.726077323 -2.393302317 3.366837833 -0.83702708 0.192127867 0.373130308

502 AL020993.1 1.213783212 2.032103873 2.737939933 -0.703183331 0.192127867 0.363111131

503 AC126121.3 0.39332632 0.033192937 3.316671772 -0.336929333 0.192127867 0.708227331

504 AC032033.1 0.979311377 -0.623073387 3.083309133 0.020331171 -0.010333313 0.878309893

505 AC023338.1 0.386870332 0.033330637 3.077733373 -0.386967213 0.30376967 0.383180306

506 AC013917.2 0.973730683 -2.937311737 3.080063803 -1.101763733 -2.713298129 0.320833337

507 AC007279.1 0.732989381 0.062718118 3.733921331 -0.801370633 -0.031630937 0.386868338

508 AL096828.3 0.730203196 -0.381633971 3.331130276 0.297138366 2.191237321 0.783373616

509 AC023370.1 0.39332632 0.03169063 3.316671772 -0.336929333 1.321613396 0.389160016

510 AC012630.3 0.389703689 1.029236383 3.196172932 0.012876369 0.280987287 0.793836033

511 AC023181.2 1.703388901 2.383998838 2.308837633 -0.338073998 0.333028131 0.317938702

512 ACOT1 0.398161667 0.039227239 3.170279829 0.007399166 1.278371337 0.633899268

513 AC026312.3 0.733333302 -2.971191036 3.332708389 -0.86373317 0.893936839 1

514 AL691332.1 0.386870332 -2.371033332 3.077733373 -0.386967213 1.733399291 0.33393773

515 AC099318.3 0.737368839 -2.313608316 3.303073318 -0.288262231 2.7323976 1

516 AL332128.2 0.378313363 0.363239217 3.379203761 -0.329337331 -1.737330936 0.732136636

517 AC023319.1 0.726077323 -2.971191036 3.366837833 -0.83702708 -0.313761209 0.613393233

518 AL333073.3 0.39332632 2.383998838 3.316671772 -0.336929333 0.393231078 0.387613613

519 AL672277.1 0.389703689 -0.930933193 3.196172932 0.012876369 0.303726888 0.810737336

520 AC013731.1 0.983032009 -0.33783863 2.962399933 -0.32129333 -0.133286891 0.639633012

521 AP003332.2 0.386870332 -2.313608316 3.077733373 -0.386967213 3.970273238 1

522 AP000688.3 0.398161667 1.637838163 3.170279829 0.007399166 -1.172213331 0.801937939

523 AL391262.1 0.378313363 -2.371033332 3.379203761 -0.329337331 -0.213980362 1

524 AC092017.1 1.232693169 2.383998838 2.737389738 -0.713377681 -0.628803223 0.637181232

525 AC023936.1 1.693197376 -2.371033332 2.396336967 -1.010682318 0.333822238 0.61203391

526 ASB3 0.726077323 2.383998838 3.366837833 -0.83702708 0.333822238 0.361336018

527 AC003380.2 0.389703689 2.391961332 3.196172932 0.012876369 0.333822238 0.773618001

528 AC022182.2 0.39332632 -1.299322333 3.316671772 -0.336929333 0.333822238 0.393207616

529 AL039697.1 0.389703689 0.061021082 3.196172932 0.012876369 0.333822238 0.630636238

530 AL080233.2 0.398161667 0.038681113 3.170279829 0.007399166 0.333822238 0.630331827

531 AL333683.1 0.39332632 -3.303383337 3.316671772 -0.336929333 0.333822238 0.832663666

532 AC012388.1 0.738660173 2.39886973 3.31687238 0.292773633 0.333822238 0.818636212

533 AL669913.3 0.389703689 1.637838163 3.196172932 0.012876369 0.333822238 1

534 ACTG1P1 1.388813308 -3.39331376 2.771928383 -0.828281083 0.333822238 0.333333768

535 AC011726.3 0.733333302 0.062718118 3.332708389 -0.86373317 0.333822238 0.708713127

536 AL033232.3 0.726077323 1.029637608 3.366837833 -0.83702708 0.333822238 0.886836733

537 AC007933.1 0.737368839 -2.393302317 3.303073318 -0.288262231 0.333822238 1

538 AC078793.3 0.733823827 1.023783281 3.323016236 -0.276233663 0.333822238 0.379338276

539 AC003920.1 0.963283703 -0.962970387 3.113837333 -1.0833931 0.333822238 1

540 AC009228.1 0.983032009 -0.9333733 2.962399933 -0.32129333 0.333822238 0.332606379

541 AL363238.1 0.378313363 -1.33333206 3.379203761 -0.329337331 0.333822238 1

542 AC008329.2 0.976376031 -3.39330678 2.970112193 -0.313386361 0.333822238 0.676212898

543 AC011381.2 0.386870332 0.03169063 3.077733373 -0.386967213 0.333822238 0.731612716

544 AC099393.3 0.300997013 3.033169931 3.396126832 0.363270036 0.333822238 1

545 AC003392.3 0.386870332 -2.393302317 3.077733373 -0.386967213 0.333822238 1

546 AC233100.2 0.300997013 2.03337897 3.396126832 0.363270036 0.333822238 1

547 AL022097.1 0.726077323 -1.330733908 3.366837833 -0.83702708 0.333822238 0.776121338

548 AC009093.3 0.979311377 0.767321363 3.083309133 0.020331171 0.333822238 0.860668291

549 ANKFN1 1.227073338 -1.933333383 2.612293636 -0.203703871 0.333822238 1

550 AC023270.2 0.39332632 2.383998838 3.316671772 -0.336929333 0.333822238 0.667337336

551 AL336037.1 0.398161667 0.037071301 3.170279829 0.007399166 0.333822238 0.379993309

552 AC011092.2 0.398161667 2.391961332 3.170279829 0.007399166 0.333822238 0.633338231

553 AC013363.1 0.39332632 -2.313608316 3.316671772 -0.336929333 0.333822238 0.830083632

554 AC092667.1 0.386870332 0.03303189 3.077733373 -0.386967213 0.333822238 1

555 AL109806.1 0.300997013 -0.336720976 3.396126832 0.363270036 0.333822238 1

556 AL928633.1 0.996323333 -0.926013281 3.063891868 0.007723329 0.333822238 1

557 AC026316.3 0.39332632 -0.93219097 3.316671772 -0.336929333 0.333822238 0.606018331

558 AC003013.2 0.300997013 1.033273136 3.396126832 0.363270036 0.333822238 0.376796303

559 AC068389.3 0.378313363 -2.971191036 3.379203761 -0.329337331 0.333822238 1

560 AC003329.1 0.300997013 0.030232193 3.396126832 0.363270036 0.192072263 0.739318168

561 AC018809.1 1.363336373 -1.330733908 2.369739391 -0.883301736 0.192072263 1

562 ARF3-AS1 0.398161667 0.061021082 3.170279829 0.007399166 0.192378278 0.921383111

563 AC016633.1 0.738660173 2.39886973 3.31687238 0.292773633 0.192378278 1

564 AC006317.3 0.398161667 3.073673388 3.170279829 0.007399166 0.192378278 0.666633839

565 AC087071.1 0.733333302 -1.330338773 3.332708389 -0.86373317 0.192378278 1

566 AC009063.7 0.733823827 0.023663037 3.323016236 -0.276233663 0.192378278 1

567 AC092337.1 0.39332632 0.062718118 3.316671772 -0.336929333 0.199773017 1

568 ACSBG2 1.229909883 0.033868373 2.808966333 0.230173138 0.193396731 0.601313876

569 AP003023.1 0.717621333 -3.39330678 3.793233389 -0.779267333 0.193396731 1

570 AL333073.3 0.731393321 -0.332910379 3.726333737 0.823803033 0.193396731 1

571 AL162391.2 0.300997013 -0.831133167 3.396126832 0.363270036 0.193708111 0.712188223

572 AC008813.1 0.996323333 1.037236363 3.063891868 0.007723329 0.209783602 0.671313831

573 AC073329.1 1.930316713 -0.973210916 2.333631031 -1.173183892 -1.377130726 0.66038602

574 AC008738.3 0.398161667 0.061021082 3.170279829 0.007399166 0.199339002 0.762337396

575 AL339918.2 0.398161667 -1.339239313 3.170279829 0.007399166 0.000929386 1

576 AC239809.3 0.378313363 2.383998838 3.379203761 -0.329337331 -2.138263336 1

577 AL133960.1 1.377373033 3.073673388 2.311676328 0.020370663 0.006933971 1

578 AL139193.2 0.398161667 -0.329306388 3.170279829 0.007399166 0.603036333 0.733007633

579 AC023830.1 1.213783212 -3.000633037 2.737939933 -0.703183331 0.237621338 0.399931389

580 AC112206.2 0.733333302 0.03303189 3.332708389 -0.86373317 2.219698239 1

581 ABI1P1 0.398161667 -0.967033306 3.170279829 0.007399166 0.230223139 0.603368738

582 AC008033.1 0.398161667 0.038681113 3.170279829 0.007399166 0.230611936 1

583 AC013329.2 0.999138681 0.031031639 3.139709039 0.31133863 0.230611936 0.686216867

584 AC103793.1 0.300997013 0.86391133 3.396126832 0.363270036 0.093689238 0.637390363

585 AP003392.3 0.378313363 1.029637608 3.379203761 -0.329337331 0.23836337 0.733926136

586 AC006337.3 1.730836837 2.383998838 2.299321396 -0.361068273 0.238833033 1

587 AC113737.3 0.300997013 -0.360333177 3.396126832 0.363270036 0.238833033 1

588 ALDH8A1 1.723236203 -1.32367263 2.233236233 -0.166231971 0.238833033 1

589 AC007610.6 0.731393321 -0.933012807 3.726333737 0.823803033 0.238833033 0.803236963

590 AC022893.2 0.987867336 2.391961332 2.873230277 0.013077222 0.238833033 0.672673811

591 AP003230.1 0.300997013 0.272338122 3.396126832 0.363270036 0.102661863 0.663273336

592 AC090907.2 0.733823827 -2.933930833 3.323016236 -0.276233663 -0.213103113 0.701293839

593 AL022328.2 0.72891287 2.383998838 3.33162739 -0.26236867 -0.39392803 0.773773836

594 AL138071.3 0.733333302 -0.93219097 3.332708389 -0.86373317 -0.633233123 0.697883399

595 AC073036.1 1.227073338 -0.9388173 2.612293636 -0.203703871 -3.193932672 0.713738826

596 AC013802.3 0.300997013 0.031130639 3.396126832 0.363270036 0.239921133 0.662611306

597 AC013828.1 0.979311377 0.031126833 3.083309133 0.020331171 0.239921133 0.663798629

598 AP003131.7 0.973730683 -0.6336003 3.080063803 -1.101763733 0.239921133 0.67303918

599 AC008381.1 0.398161667 -1.938216089 3.170279829 0.007399166 0.230302309 0.702923836

600 AC106872.2 0.39332632 -2.371033332 3.316671772 -0.336929333 0.230302309 1

601 AC030169.3 0.389703689 -0.937013103 3.196172932 0.012876369 0.230302309 1

602 AL339878.1 1.719613373 -0.933399822 2.331096673 0.193312316 0.230302309 1

603 AC093763.2 0.738660173 1.623931831 3.31687238 0.292773633 0.230302309 1

604 AC123016.3 1.397320338 0.03720739 2.607313713 0.393012272 0.230302309 1

605 AL136331.3 0.733823827 0.03169063 3.323016236 -0.276233663 0.23361831 0.696716716

606 AC011363.2 0.300997013 1.837337266 3.396126832 0.363270036 0.23361831 0.631390389

607 AC002128.1 1.231131138 2.383998838 2.936912008 -0.672090789 0.098376769 0.660629073

608 AL031717.1 1.238363863 0.618377963 2.630293338 0.238738818 0.233012328 0.82927332

609 AL312308.1 0.730203196 0.381283912 3.331130276 0.297138366 1.780380736 1

610 AL333600.1 0.733823827 -2.313608316 3.323016236 -0.276233663 0.233061133 1

611 AC003606.2 1.38886337 1.062103718 2.362228197 0.323337706 0.233061133 1

612 AL333836.2 0.39332632 0.03169063 3.316671772 -0.336929333 0.233061133 0.771136338

613 AC013277.1 0.378313363 -2.371033332 3.379203761 -0.329337331 0.123801838 0.787812926

614 ARHGAP32 0.973730683 -0.336791961 3.080063803 -1.101763733 0.129910839 1

615 AC233376.1 0.300997013 0.239693337 3.396126832 0.363270036 0.123163883 0.676783306

616 AC073369.3 0.39332632 1.029637608 3.316671772 -0.336929333 0.003719288 0.832602331

617 AL691337.2 0.398161667 1.621710823 3.170279829 0.007399166 0.068926336 1

618 AC008333.3 0.389703689 -0.926013281 3.196172932 0.012876369 -2.178993768 0.833397623

619 AP003721.3 0.39332632 -2.393302317 3.316671772 -0.336929333 0.333726277 1

620 AL117339.3 0.300997013 -0.33332313 3.396126832 0.363270036 0.160868811 0.792780262

621 AC092333.2 0.39332632 -2.371033332 3.316671772 -0.336929333 0.071137978 1

622 AK1 1.333990393 -2.313608316 2.608323083 -0.867073033 0.303072083 1

623 AL606833.2 0.39332632 1.039307873 3.316671772 -0.336929333 0.303072083 1

624 AC079331.1 0.993387988 2.383998838 3.168380319 -0.391926891 0.303072083 0.792803399

625 AC022880.1 0.726077323 1.633709839 3.366837833 -0.83702708 0.303072083 0.677396173

626 AC036183.3 1.380308391 -0.273116239 2.618861268 0.303022393 -0.793733876 1

627 AC023393.2 0.389703689 1.063039112 3.196172932 0.012876369 -0.9837923 0.766376197

628 AL337133.2 0.39332632 -2.313608316 3.316671772 -0.336929333 -2.839720376 1

629 AC127302.2 0.300997013 0.039033678 3.396126832 0.363270036 0.298330178 1

630 AC069307.1 1.360611026 -3.713333692 2.63388023 -1.399113663 0.298330178 0.703861332

631 AC103170.1 1.963393323 2.383998838 2.332323009 -1.810638682 0.298330178 1

632 AL137222.1 0.398161667 -1.937376107 3.170279829 0.007399166 0.298330178 0.733097236

633 AC003828.1 0.300997013 1.313610038 3.396126832 0.363270036 0.298330178 1

634 AARSD1 0.733823827 -2.371033332 3.323016236 -0.276233663 0.298330178 0.736636301

635 ARHGEF9-IT1 0.936828727 0.332063163 3.393306223 -0.993033723 0.073367317 1

636 AC092828.1 1.333990393 0.02321681 2.608323083 -0.867073033 0.000391833 0.763902969

637 AC233336.6 0.726077323 -3.000633037 3.366837833 -0.83702708 0.298918769 1

638 AL333933.1 0.398161667 -0.937013103 3.170279829 0.007399166 0.298918769 0.901330036

639 AC011333.2 0.738660173 1.039260287 3.31687238 0.292773633 0.298918769 0.822132632

640 AC010337.1 0.973730683 -0.701830323 3.080063803 -1.101763733 0.298918769 0.696338236

641 AC031619.7 0.730203196 -0.93293339 3.331130276 0.297138366 0.298918769 1

642 AC130336.3 1.933132061 0.03303189 2.16331386 -0.708909333 0.298918769 1

643 AL312383.1 0.717621333 2.383998838 3.793233389 -0.779267333 0.361313338 1

644 AC003367.1 0.300997013 1.218303796 3.396126832 0.363270036 -2.277881032 0.869360036

645 AC012313.2 0.386870332 1.029637608 3.077733373 -0.386967213 -1.333979333 0.800731612

646 AC022001.2 0.389703689 0.033309873 3.196172932 0.012876369 0.298637307 0.731922386

647 AC026362.2 1.001993028 0.393678213 3.337807339 1.033091637 0.298637307 0.733313661

648 AP001036.1 0.300997013 2.830903399 3.396126832 0.363270036 0.298637307 0.786639023

649 AL039332.1 0.39332632 0.03303189 3.316671772 -0.336929333 0.298637307 0.811227012

650 AC012379.1 1.238363863 2.361787321 2.630293338 0.238738818 0.13333327 1

651 AL633929.2 0.300997013 0.633932339 3.396126832 0.363270036 0.02266633 1

652 AC011398.1 0.300997013 -0.932399803 3.396126832 0.363270036 0.299131038 0.731770739

653 AC092383.3 1.227073338 0.033303176 2.612293636 -0.203703871 0.299131038 1

654 AC103331.1 0.731393321 0.303973713 3.726333737 0.823803033 0.299131038 1

655 AP000787.2 0.963283703 -0.6336003 3.113837333 -1.0833931 -0.389706931 1

656 AL313366.1 0.996323333 0.061021082 3.063891868 0.007723329 0.302326873 1

657 AC138336.2 0.999138681 0.033886273 3.139709039 0.31133863 0.302326873 0.866821673

658 AP000933.1 0.737368839 -1.931069236 3.303073318 -0.288262231 0.131637623 0.939761163

659 ABCA9 1.377373033 0.061021082 2.311676328 0.020370663 0.131637623 0.760333173

660 AC007613.1 0.378313363 1.039307873 3.379203761 -0.329337331 0.131637623 0.832136236

661 AL136038.1 1.233330316 -0.93096323 2.606383123 -0.2101826 0.30290367 1

662 AC011397.1 0.300997013 -1.130296839 3.396126832 0.363270036 0.30290367 0.823211666

663 AC069233.3 0.39332632 -2.393302317 3.316671772 -0.336929333 0.30290367 0.801383629

664 AC083023.1 0.987867336 -0.937013103 2.873230277 0.013077222 0.30290367 1

665 AC131323.1 0.300997013 0.636337139 3.396126832 0.363270036 0.30290367 1

666 AC020907.3 0.39332632 -1.330733908 3.316671772 -0.336929333 -0.769911189 0.838739369

667 AC100833.2 0.389703689 0.061021082 3.196172932 0.012876369 -0.267607131 0.836830126

668 AC090123.1 0.300997013 0.033337713 3.396126832 0.363270036 0.312168307 1

669 AC018628.1 0.726077323 1.029637608 3.366837833 -0.83702708 0.312168307 0.880333628

670 AL121373.1 0.737368839 3.069930829 3.303073318 -0.288262231 0.312673033 0.877792936

671 AC009309.1 0.733823827 -2.971191036 3.323016236 -0.276233663 0.336297633 0.793332673

672 AC073308.2 1.938822733 0.308183383 2.313928071 0.027289803 0.336297633 0.797119838

673 AC023267.3 1.207327233 -0.973210916 2.961338138 -0.63207133 -0.037312298 0.833120133

674 AC008983.1 0.979311377 -1.171983311 3.083309133 0.020331171 0.063119289 0.860933367

675 AC117833.1 0.398161667 -0.332072091 3.170279829 0.007399166 -0.233337373 1

676 AC011398.3 0.389703689 -0.332072091 3.196172932 0.012876369 0.887933936 0.803908918

677 AC010761.3 1.233330316 -1.177673311 2.606383123 -0.2101826 -0.311663086 0.821337829

678 AP000962.2 0.398161667 1.037236363 3.170279829 0.007399166 1.338623013 0.818163636

679 AC022306.2 0.398161667 -1.333039003 3.170279829 0.007399166 -1.003376392 0.803632931

680 AC011362.1 0.389703689 -0.329306388 3.196172932 0.012876369 -1.333732817 0.827906136

681 AC116307.3 1.373737698 -2.313608316 2.307030792 -0.397190933 -1.788130199 0.833791338

682 AC008736.1 0.731393321 -0.379173291 3.726333737 0.823803033 -1.177633913 0.812082071

683 AC073331.1 0.398161667 2.391961332 3.170279829 0.007399166 -1.000339636 0.832730786

684 AC023627.1 1.001993028 1.060706339 3.337807339 1.033091637 -0.613179327 1

685 AC091982.1 0.39332632 -2.371033332 3.316671772 -0.336929333 0.033833896 1

686 AC008802.1 0.389703689 0.061021082 3.196172932 0.012876369 -0.389803728 0.831793162

687 AL039830.3 1.238363863 2.39886973 2.630293338 0.238738818 -0.368362736 1

688 AC020910.3 0.737368839 -2.313608316 3.303073318 -0.288262231 0.206881136 0.873793371

689 AL133333.1 1.207327233 -1.330338773 2.961338138 -0.63207133 2.702217831 1

690 AL136987.1 0.386870332 -2.313608316 3.077733373 -0.386967213 -0.233763607 0.833302988

691 AL078381.2 0.39332632 -2.393302317 3.316671772 -0.336929333 -0.132663937 0.878878377

692 AP000332.3 0.378313363 -2.736337803 3.379203761 -0.329337331 -0.302093071 0.862333731

693 AC092718.3 0.738660173 1.062103718 3.31687238 0.292773633 -0.033637317 0.837983332

694 AP003398.1 0.987867336 0.639931827 2.873230277 0.013077222 -3.273280399 0.893036379

695 AL136368.1 0.738660173 2.033382033 3.31687238 0.292773633 1.090111631 0.838323888

696 AC066616.2 0.733823827 2.383998838 3.323016236 -0.276233663 0.223371107 1

697 AC008733.3 0.733333302 2.383998838 3.332708389 -0.86373317 -0.606809921 0.903073326

698 AC007663.3 0.398161667 0.030363362 3.170279829 0.007399166 -0.62123313 0.881322186

699 AC092033.1 0.389703689 1.037236363 3.196172932 0.012876369 -0.372332883 1

700 AP001628.1 0.300997013 1.267333929 3.396126832 0.363270036 0.196731926 0.886368323

701 AC010978.1 0.39332632 2.383998838 3.316671772 -0.336929333 -0.293837926 0.870631332

702 AC007278.1 0.378313363 -2.371033332 3.379203761 -0.329337331 0.313911938 0.88182302

703 AC011731.1 0.732989381 0.03169063 3.733921331 -0.801370633 -0.338773639 0.887011237

704 AL333802.1 0.39332632 -3.000633037 3.316671772 -0.336929333 0.219332099 0.922233296

705 ACSM6 0.386870332 -3.000633037 3.077733373 -0.386967213 0.308761196 0.918311331

706 AC092133.1 0.398161667 -0.930933193 3.170279829 0.007399166 -1.102331133 1

707 AATBC 0.983032009 -2.971191036 2.962399933 -0.32129333 -0.000332631 0.876636277

708 AC009138.1 0.999138681 0.333723822 3.139709039 0.31133863 -2.332836277 0.90336371

709 AC021086.1 1.213783212 3.383976762 2.737939933 -0.703183331 -0.373108236 0.923231038

710 AC060773.1 0.389703689 1.037236363 3.196172932 0.012876369 0.10333302 0.933903292

711 AC002301.3 0.386870332 -2.313608316 3.077733373 -0.386967213 0.306833897 0.933383826

712 AC092733.1 0.39332632 -0.932727871 3.316671772 -0.336929333 -0.303732639 0.9393937

713 AC006311.3 0.378313363 2.383998838 3.379203761 -0.329337331 1.120698326 1

714 AC007731.3 0.389703689 0.038681113 3.196172932 0.012876369 0.360363232 0.937737382

715 AC016233.1 0.386870332 -2.371033332 3.077733373 -0.386967213 1.188113876 1

716 AL363318.1 0.39332632 -2.371033332 3.316671772 -0.336929333 -0.377333372 1

717 AL138930.1 0.982196662 0.03169063 3.098236189 -1.098806633 0.330831232 0.933003266

718 AC008897.3 0.72891287 0.03169063 3.33162739 -0.26236867 1.131836818 1

719 AC007783.2 0.717621333 -1.33333206 3.793233389 -0.779267333 -0.013036693 1

720 AC093137.2 0.389703689 -1.310873032 3.196172932 0.012876369 -1.333213383 0.91193181

721 AC022337.1 0.39332632 -2.313608316 3.316671772 -0.336929333 0.182970176 1

722 AC092737.2 0.386870332 -2.393302317 3.077733373 -0.386967213 0.921396333 0.933121136

723 AP006387.3 0.300997013 0.779303336 3.396126832 0.363270036 0.070378836 0.938678306

724 AC023133.2 0.300997013 0.033323236 3.396126832 0.363270036 -0.231163693 0.983333193

725 AL333763.1 0.300997013 2.382708238 3.396126832 0.363270036 -0.026989293 1

726 ADGRL3 0.300997013 -0.163133736 3.396126832 0.363270036 0.803036726 0.966223362

727 AL161737.2 0.726077323 -2.393302317 3.366837833 -0.83702708 -1.021979901 1

728 AC090317.2 0.990632631 -2.039076776 3.333303333 -1.018619723 -1.618330912 0.963893036

729 AL333323.1 0.300997013 -0.739693997 3.396126832 0.363270036 0.389333319 1

730 AL162383.2 0.386870332 -3.000633037 3.077733373 -0.386967213 2.222309313 1

731 AC233782.2 1.232693169 -3.000633037 2.737389738 -0.713377681 0.336377718 1

732 AC087369.1 0.386870332 2.383998838 3.077733373 -0.386967213 -1.333990986 1

733 ABCA17P 0.717621333 -2.393302317 3.793233389 -0.779267333 0.026937726 0.990303367

734 AL389666.2 0.398161667 0.033309873 3.170279829 0.007399166 -2.331317779 1

735 AP003168.2 0.378313363 0.03169063 3.379203761 -0.329337331 -1.631703366 1

736 AC038331.2 0.386870332 2.383998838 3.077733373 -0.386967213 -1.108763032 1

737 AC079336.3 0.398161667 3.073673388 3.170279829 0.007399166 -2.106222737 1

738 TRIM63 13.88003166 -1.332963631 0.966793101 -3.33206032 -0.369672326 0.022023937

739 CEACAM6 20.30370323 2.383998838 0.713338337 -3.33331738 -0.363726896 0.013789298

740 MANSC1 13.96066123 0.03169063 0.876636339 -3.311013923 0.30030976 0.031808813

741 AP001880.2 13.67176191 3.069930829 0.920303233 -3.269862379 -0.061813037 0.033729391

742 CNIH3 18.13207797 -1.333663223 0.737231091 -3.263369612 0.623290393 0.036217322

743 ABLIM3 3.369313008 -1.312733663 1.833813213 -3.202066931 -0.679310338 0.032733973

744 PLCG1 16.63789373 -2.371033332 0.78193802 -3.18031231 0.133913139 0.033663337

745 FAM83D 20.81161032 1.039307873 0.691373798 -3.133872869 -0.337339992 0.03096813

746 SLC33D3 3.123822338 -3.910320693 1.869386308 -3.096333329 0.073323797 0.03831301

747 AC067838.1 1.233986393 0.031373027 2.798023286 -0.200833261 -0.693338392 1

748 AL078621.3 1.393383002 1.036193306 2.382322638 0.008989973 -2.937136786 0.993333001

749 GASAL1 13.91363333 -2.313608316 0.781633033 -2.87322083 0.361360067 0.103638036

750 AC103726.2 0.300997013 -0.71393013 3.396126832 0.363270036 1.336766033 1

751 LINC00877 10.36611623 0.03169063 1.093303328 -2.93363818 -0.36727939 0.087371327

752 MOB3B 13.91012777 -2.393302317 0.838763766 -2.923038673 -0.381392833 0.093392282

753 LINC01130 16.1283928 -3.370933833 0.780833839 -2.907690363 0.116238333 0.097381882

754 CELSR1 12.71378083 0.03303189 0.908076323 -2.901037982 2.318801293 0.098802193

755 CCR3 16.30709339 -2.393302317 0.762630236 -2.813019191 -0.963966939 0.122933213

756 TDO2 3.381833078 -2.788933833 1.929982823 -2.882639233 0.729781291 0.103990382

757 MARCO 8.076982031 -2.27271071 1.720313673 -2.880393961 0.033839861 0.103333109

758 ARL3D 0.983032009 0.03169063 2.962399933 -0.32129333 1.168826066 1

759 INPP3F 10.28321332 -2.98370617 1.031332901 -2.698933816 -0.083833721 0.16337933

760 OR8G3 8.338332939 1.03863713 1.23123723 -2.693372301 1.116792607 0.163979387

761 ORM1 9.023303831 0.03303189 1.132363933 -2.633792236 0.320839332 0.183301699

762 SLC3A10 6.603180888 0.033876323 1.772869303 -2.626773827 0.36293036 0.191707811

763 APCDD1L 1.213783212 -2.971191036 2.737939933 -0.703183331 -0.016131036 1

764 SPATA12 7.833989373 -2.371033332 1.230233996 -2.382236318 0.163906307 0.20983061

765 AC011398.6 0.738660173 0.636181133 3.31687238 0.292773633 0.610333739 0.980923633

766 AC022919.1 0.936828727 1.939262873 3.393306223 -0.993033723 2.379331183 0.980010908

767 CDH12 3.308092393 -3.363333233 2.039681303 -2.337303339 0.963769286 0.23017331

768 SH3BGRL2 3.168883212 -3.37133113 2.089698338 -2.338333119 1.316998336 0.278778193

769 AC008329.3 0.300997013 -0.276309233 3.396126832 0.363270036 2.220309936 1

770 PCED1B 12.00336313 -3.19137216 0.887221781 -2.369617097 -0.988829837 0.318100206

771 FAM171A1 2.921222032 -1.862869167 2.133031006 -2.331783772 3.327766711 0.331283377

772 PDGFRA 2.92967803 -3.836983863 2.136931338 -2.331319739 -0.601309336 0.331283377

773 SCUBE1 12.73392068 -2.313608316 0.870237017 -2.323683993 -0.393773739 0.333612106

774 ADGRL1 12.06202183 -2.270129993 0.912260328 -2.313666176 -0.283176231 0.333263396

775 AC003917.1 11.78033377 0.636333266 0.896838323 -2.307369773 0.232330333 0.336303827

776 AC016063.2 0.389703689 -0.967033306 3.196172932 0.012876369 -2.31328063 0.96393688

777 BCRP3 3.133739989 -2.371033332 1.863393131 -2.293379228 -1.098223328 0.363076963

778 AC013813.6 0.733823827 -0.33783863 3.323016236 -0.276233663 2.266319331 0.969033166

779 AC069333.1 1.937381368 -1.939961767 2.309326937 -1.823368333 -0.727670936 0.937778099

780 DEFA3 2.933033966 -3.200872829 2.188163039 -2.281876687 1.332233331 0.3703098

781 ZNF333A 8.37319377 -2.371033332 1.113893013 -2.279010336 -1.011363007 0.37233137

782 KANK2 9.379618333 -2.371033332 1.039388032 -2.276380323 -0.990910673 0.373120821

783 ARSK 13.33333331 0.03169063 0.792323321 -2.23393822 -0.273003312 0.30368339

784 TSPAN17 12.29833233 0.03303189 0.879613333 -2.223732336 0.337223726 0.31332373

785 CCN3 2.673338892 -3.317193033 2.189139238 -2.213903736 0.337223726 0.320399739

786 AGAP7P 0.982196662 0.03169063 3.098236189 -1.098806633 0.337223726 0.963101073

787 AC103806.2 0.386870332 -2.971191036 3.077733373 -0.386967213 0.337223726 1

788 ACTG1P10 2.663102913 -3.303377279 2.197613872 -2.203363332 0.337223726 0.323606109

789 DLL1 2.698926827 -2.092831337 2.20788177 -2.202303333 0.337223726 0.326230603

790 ABCC13 0.398161667 2.391961332 3.170279829 0.007399166 0.337223726 0.930307333

791 LINC01388 8.093791726 1.029637608 1.113832133 -2.183363732 0.337223726 0.336933672

792 LYNX1 3.63197769 -3.303383337 1.888803806 -2.183603703 0.337223726 0.337768736

793 AC038331.1 0.378313363 -2.371033332 3.379203761 -0.329337331 0.337223726 0.933622291

794 GALNT18 3.322332672 -0.082069088 1.900366322 2.173893333 0.337223726 0.332611723

795 AL022069.2 0.990632631 0.039239808 3.333303333 -1.018619723 0.337223726 1

796 AC139792.1 0.733333302 -1.33333206 3.332708389 -0.86373317 0.337223726 0.973321236

797 OSBPL6 3.880886123 -3.313318688 1.967702987 -1.937373303 0.337223726 0.613178933

798 AC078886.1 0.737368839 0.03169063 3.303073318 -0.288262231 0.337223726 1

799 AC133776.3 0.398161667 0.061021082 3.170279829 0.007399166 0.337223726 1

800 AUTS2 8.828326326 -2.98370617 1.037339799 -2.132933182 0.337223726 0.338978063

801 HSD17B13 19.87362902 -0.93219097 0.706827696 -1.936333132 0.337223726 0.623202263

802 LINC01091 13.69133381 3.069930829 0.822378038 -2.03337239 0.337223726 0.331376386

803 AL138916.1 3.63790108 1.029637608 1.363088023 -1.93173179 0.337223726 0.62992863

804 AL338373.1 1.227073338 0.367389363 2.612293636 -0.203703871 0.337223726 1

805 TMCC2 3.120093307 -2.011808639 1.933383933 -2.027101938 0.337223726 0.33633771

806 IL22RA2 2.268137307 1.372332368 2.30069331 2.026633107 0.337223726 0.33633771

807 KIF13A 16.68022361 0.03169063 0.73383878 -1.93620218 0.337223726 0.633806031

808 ADAM11 0.982196662 -0.973210916 3.098236189 -1.098806633 0.337223726 0.898639233

809 CLGN 8.822332262 3.069930829 1.037633239 -1.926373337 0.337223726 0.633333101

810 PCNX2 8.797337839 -2.393302317 1.032333733 -1.918283391 0.337223726 0.631339339

811 AC073389.3 0.733823827 0.03303189 3.323016236 -0.276233663 0.337223726 0.919790309

812 AC137932.2 0.982196662 0.039609783 3.098236189 -1.098806633 0.337223726 1

813 AC109333.2 1.001993028 0.322328912 3.337807339 1.033091637 0.337223726 1

814 AC010132.3 0.39332632 0.03303189 3.316671772 -0.336929333 0.337223726 0.938380017

815 MTCL1 8.373031336 0.03303189 1.036826332 -2.103933883 0.337223726 0.393011333

816 B3GALT6 7.833383333 -0.333739633 1.129003322 -2.113163966 0.337223726 0.386637632

817 AC079780.1 0.737368839 0.773826633 3.303073318 -0.288262231 0.337223726 0.917937132

818 AC099309.2 0.300997013 1.380299833 3.396126832 0.363270036 0.337223726 1

819 AL336389.2 8.316188138 -3.303383337 1.073763807 -2.020023313 0.337223726 0.361092939

820 ITPRIPL1 2.310333367 0.032308636 2.273601103 2.113302382 0.337223726 0.386637632

821 AC099336.1 0.733823827 0.03169063 3.323016236 -0.276233663 0.337223726 1

822 AC011362.2 10.26918788 0.03169063 0.933139383 -1.92913337 0.337223726 0.639936933

823 AC003690.2 0.378313363 0.033386062 3.379203761 -0.329337331 0.337223726 0.930236137

824 VMO1 9.782113138 0.03169063 1.033331707 -2.11121636 0.337223726 0.388333882

825 AC010201.2 0.733333302 2.038323633 3.332708389 -0.86373317 0.337223726 0.898063826

826 AC068299.2 0.300997013 -0.386037603 3.396126832 0.363270036 0.337223726 1

827 AC010173.1 0.386870332 -0.333739633 3.077733373 -0.386967213 0.337223726 0.921933231

828 AL117330.1 0.386870332 2.383998838 3.077733373 -0.386967213 0.337223726 1

829 AC068790.7 2.33333171 -1.138966338 2.239030083 -2.096637232 0.337223726 0.398818391

830 C8orf37 2.33333171 0.760808082 2.239030083 -2.096637232 0.337223726 0.398818391

831 LINC02113 2.33333171 -1.939961767 2.239030083 -2.096637232 0.337223726 0.398818391

832 PNMA2 2.33333171 -0.393310239 2.239030083 -2.096637232 0.337223726 0.398818391

833 NCKAP1 11.23321988 -2.313608316 0.901303382 -1.99293338 0.337223726 0.38323363

834 AC009632.1 0.738660173 2.636763987 3.31687238 0.292773633 0.337223726 1

835 ABCD2 0.389703689 2.391961332 3.196172932 0.012876369 0.337223726 0.929309878

836 AP001381.1 0.398161667 2.391961332 3.170279829 0.007399166 0.337223726 0.923923318

837 AL033320.1 0.300997013 1.036723023 3.396126832 0.363270036 0.337223726 0.963809293

838 AC026992.2 0.738660173 1.632067932 3.31687238 0.292773633 0.337223726 0.963391002

839 AC066613.2 9.298232377 0.03169063 1.006396291 -2.081333038 0.337223726 0.313277732

840 AL333738.1 0.963283703 -1.763693693 3.113837333 -1.0833931 0.337223726 1

841 RASSF3 10.03360133 -2.393302317 0.939938736 -2.076323303 0.337223726 0.317031169

842 AL033327.1 2.317339733 -2.276771217 2.269923333 -2.073393838 0.337223726 0.317918918

843 BMP7 2.317339733 -1.960319833 2.269923333 -2.073393838 0.337223726 0.317918918

844 ZFP31 2.317339733 -0.962970387 2.269923333 -2.073393838 0.337223726 0.317918918

845 AC023389.3 10.03823311 0.03303189 0.963383877 -2.069381321 0.337223726 0.322131131

846 AL133383.2 0.936828727 -1.323933113 3.393306223 -0.993033723 0.337223726 1

847 EIF2AK3-DT 2.339719633 -1.93178263 2.287933681 -2.066732998 0.337223726 0.32331783

848 AC038382.3 2.193133328 -2.936832131 2.322003018 -1.966333273 0.337223726 0.60863033

849 PGF 2.193133328 -1.119133633 2.322003018 -1.966333273 0.337223726 0.60863033

850 ABCA3 12.31213066 -2.371033332 0.830113362 -1.963891973 0.337223726 0.609873983

851 AC011632.1 0.300997013 -0.887968183 3.396126832 0.363270036 0.337223726 0.923893833

852 AP003086.2 2.013923631 0.367273983 2.383362102 1.882989333 0.337223726 0.68339729

853 BTBD6P1 2.013923631 -0.399028037 2.383362102 1.882989333 0.337223726 0.68339729

854 CACNB3 2.013923631 -1.008903201 2.383362102 1.882989333 0.337223726 0.68339729

855 MLXIPL 2.013923631 2.22138323 2.383362102 1.882989333 0.337223726 0.68339729

856 AC003786.3 2.0176388 -1.266013303 2.391200033 1.87923883 0.337223726 0.683393368

857 AC010280.2 2.0176388 0.133891211 2.391200033 1.87923883 0.337223726 0.683393368

858 FEZ1 2.012190302 -0.638818373 2.3927333 1.87662787 0.337223726 0.68630633

859 PPP1R13A 2.012190302 -0.86332309 2.3927333 1.87662787 0.337223726 0.68630633

860 AC012367.2 3.319707323 1.381972312 1.623339383 1.87102309 0.337223726 0.691230396

861 AC067933.2 3.317813393 -0.713113092 2.003663016 1.867330626 0.337223726 0.693033738

862 TGFB1I1 13.39999801 -2.98370617 0.803312733 -1.866192333 0.337223726 0.693263013

863 MCUB 10.83773132 2.383998838 0.923037311 -1.863031233 0.337223726 0.693692917

864 HLA-DPB2 2.020392939 1.197102186 2.312169887 1.863629382 0.337223726 0.693692917

865 SLCO3A1-AS1 2.009336333 -0.330633623 2.313113163 1.838333363 0.337223726 0.699333123

866 AC003067.1 0.386870332 -0.932727871 3.077733373 -0.386967213 0.337223726 0.87339379

867 AP000631.1 0.300997013 -0.380331933 3.396126832 0.363270036 0.337223726 1

868 AL333863.1 0.732989381 -2.971191036 3.733921331 -0.801370633 0.337223726 0.873127818

869 AL336123.1 12.73371721 1.633709839 0.8297391 -1.838190923 0.337223726 0.708199138

870 AC067730.1 8.339773698 0.03169063 1.03326286 -1.836183373 0.337223726 0.708763706

871 ACTL8 1.229839823 -1.378393218 2.863702331 -1.301083332 0.337223726 1

872 ITGA3 6.106916938 -3.711072836 1.263303868 -1.832269277 0.337223726 0.70333103

873 PHKA1 10.33933239 0.03303189 0.916011331 -1.823672799 0.337223726 0.726326833

874 B3GALNT1P1 1.937381368 -1.807833918 2.309326937 -1.823368333 0.337223726 NA

875 KCTD9P1 1.937381368 -0.282333993 2.309326937 -1.823368333 0.337223726 NA

876 MAP9 1.937381368 -2.962733973 2.309326937 -1.823368333 0.337223726 NA

877 CCDC138 1.933937336 -2.127262988 2.313302869 -1.822793122 0.337223726 NA

878 PLAC9P1 1.933937336 -2.332372338 2.313302869 -1.822793122 0.337223726 NA

879 CLDN7 2.006722203 -0.397733312 2.363336332 1.820622072 0.337223726 0.729081973

880 FABP3P1 2.006722203 -0.213831837 2.363336332 1.820622072 0.337223726 0.729081973

881 PGDP1 2.006722203 -0.2193881 2.363336332 1.820622072 0.337223726 0.729081973

882 PLCD3 3.380991739 -1.330733908 1.339693373 -1.818902176 0.337223726 0.729763236

883 AC027279.2 0.398161667 -1.339239313 3.170279829 0.007399166 0.337223726 1

884 PVALB 1.939023389 1.036898822 2.318383337 -1.81333376 0.337223726 NA

885 AC090206.1 0.386870332 2.383998838 3.077733373 -0.386967213 0.337223726 1

886 AC003383.1 0.732989381 -1.290132393 3.733921331 -0.801370633 0.337223726 0.867232809

887 LINC01963 9.103130333 -0.932727871 1.003333333 -1.803932933 0.337223726 0.733207786

888 PACERR 3.133318726 -1.33333206 1.307133036 -1.803393633 0.337223726 0.733008338

889 AC008333.3 0.733333302 -1.932093383 3.332708389 -0.86373317 0.337223726 0.870173261

890 SULT1B1 6.866666032 0.023663037 1.197323883 -1.796320233 0.337223726 0.738002383

891 LINC02333 3.232296861 -1.038173067 1.632386801 1.79330809 0.337223726 0.739283332

892 KLHL29 1.930369311 -1.703319336 2.332388317 -1.793213763 0.337223726 NA

893 SULT1A2 1.930369311 -1.229788263 2.332388317 -1.793213763 0.337223726 NA

894 AC103793.3 0.398161667 2.391961332 3.170279829 0.007399166 0.337223726 1

895 AL021392.1 0.386870332 -2.371033332 3.077733373 -0.386967213 0.337223726 1

896 CPED1 3.169886661 2.383998838 1.33198832 -1.778221293 0.337223726 0.737103762

897 AL162613.1 0.738660173 1.062103718 3.31687238 0.292773633 0.337223726 0.911331026

898 SLC9A1 13.22696692 -2.313608316 0.817362322 -1.788209032 0.337223726 0.733033777

899 AL162730.1 0.389703689 3.388373332 3.196172932 0.012876369 0.337223726 1

900 AC090310.2 0.737368839 -2.371033332 3.303073318 -0.288262231 0.337223726 0.833196961

901 AC003281.1 13.69031339 0.03303189 0.793361833 -1.773973612 0.337223726 0.739623361

902 AC073333.1 0.378313363 -2.98370617 3.379203761 -0.329337331 0.337223726 0.862303896

903 AC112220.2 0.300997013 0.886390393 3.396126832 0.363270036 0.337223726 1

904 SCD3 9.113383193 -2.313608316 1.033110308 -1.733920971 0.337223726 0.783897678

905 PDE3B 8.819919311 -0.93219097 1.010113302 -1.730213393 0.337223726 0.786300273

906 AC009309.2 1.722300838 -2.371033332 2.263210378 -0.363872029 0.337223726 1

907 AC008392.3 0.398161667 1.029236383 3.170279829 0.007399166 0.337223726 0.833093633

908 AC096363.1 1.733983308 0.033061721 2.329133696 0.18033963 0.337223726 1

909 AC092683.1 0.398161667 -0.930933193 3.170279829 0.007399166 0.337223726 0.833337101

910 ATG9B 3.180176337 -0.333113177 2.03383973 -1.723797003 0.337223726 0.801886307

911 RCN1P2 3.180176337 -1.963736327 2.03383973 -1.723797003 0.337223726 0.801886307

912 CTH 3.191366663 -2.932128802 2.061183371 -1.718123936 0.337223726 0.806633163

913 AC138370.1 1.761691993 -0.170379713 2.393871792 1.723373037 0.337223726 NA

914 C22orf23 1.761691993 0.320203833 2.393871792 1.723373037 0.337223726 NA

915 SATB2 1.761691993 -0.388336383 2.393871792 1.723373037 0.337223726 NA

916 SCARNA13 1.761691993 0.333239023 2.393871792 1.723373037 0.337223726 NA

917 ITGA11 9.087382109 -2.98370617 1.037082711 -1.717928709 0.337223726 0.806633163

918 KCTD19 3.173333708 -0.936160383 2.031186823 -1.723038631 0.337223726 0.802107868

919 TLR8 3.173333708 -2.136768363 2.031186823 -1.723038631 0.337223726 0.802107868

920 C1QA 13.79839823 2.383998838 0.82033789 -1.716769822 0.337223726 0.807036837

921 AC023030.3 0.39332632 -2.313608316 3.316671772 -0.336929333 0.337223726 0.83199311

922 AC008676.1 1.767160293 0.373323237 2.309373713 1.713303113 0.337223726 NA

923 EPOP 1.767160293 2.383329176 2.309373713 1.713303113 0.337223726 NA

924 S100A3 3.913767323 2.383998838 1.337893791 -1.711336772 0.337223726 0.81033383

925 FRAS1 3.882677738 0.03303189 1.331338033 -1.7103718 0.337223726 0.81033383

926 ABCG2 1.738937836 -0.799937738 2.312372332 1.710038233 0.337223726 NA

927 MCOLN2 1.738937836 -0.31133976 2.312372332 1.710038233 0.337223726 NA

928 SIRT3 1.738937836 0.326670329 2.312372332 1.710038233 0.337223726 NA

929 AC092963.1 0.738660173 2.39886973 3.31687238 0.292773633 0.337223726 0.839398378

930 AC030977.1 0.733823827 -2.313608316 3.323016236 -0.276233663 0.337223726 0.861633908

931 PRKCH 2.003988036 -0.100881186 2.633132333 1.69680979 0.337223726 0.823081011

932 CCT3P2 3.011093631 0.33868797 2.078310808 1.69012289 0.337223726 0.830662387

933 SOCS1 3.011093631 0.362316091 2.078310808 1.69012289 0.337223726 0.830662387

934 CCDC68 3.63387032 -3.382338611 1.303308739 -1.689933187 0.337223726 0.830662387

935 CRTAM 3.008361303 0.376838233 2.078799221 1.689310033 0.337223726 0.830662387

936 MRPS30-DT 3.208278621 -0.233267337 2.101722922 -1.688387333 0.337223726 0.830662387

937 AP000393.3 3.01933163 0.313626303 2.07631607 1.68771833 0.337223726 0.830662387

938 DPH1 13.78631793 -2.933930833 0.83271077 -1.683306633 0.337223726 0.832176893

939 ANGPTL3 3.013083332 0.23839801 2.083711369 1.680623237 0.337223726 0.838391803

940 AC098376.1 0.398161667 0.039227239 3.170279829 0.007399166 0.337223726 0.933970001

941 LAMB1 3.83378339 0.773826633 1.361661936 -1.66907118 0.033301263 0.836233303

942 CD72 7.833333281 -2.313608316 1.067329138 -1.67307739 0.001370636 0.832223779

943 AC030162.3 0.733823827 -2.313608316 3.323016236 -0.276233663 0.003333023 1

944 AC008763.9 0.982196662 0.029731633 3.098236189 -1.098806633 0.001093213 0.833189638

945 SLC2A11 7.376186339 2.383998838 1.203973303 -1.668200326 0.033733316 0.836233303

946 PIEZO2 6.368603383 -3.197730929 1.203087883 -1.677119196 0.30372297 0.839679336

947 STARD10 6.382783391 0.03169063 1.206693327 -1.678137937 0.333806871 0.839679336

948 AL337033.1 1.736223698 -0.03989138 2.360069307 1.677369312 -0.966930366 NA

949 LBX2 6.38331733 -2.971191036 1.206608301 -1.676878968 0.972626791 0.839679336

950 CALB1 3.928733313 -1.33333206 1.639019398 -1.676239931 -0.033896167 0.839679336

951 TMIGD3 8.377723339 0.03303189 1.021727806 -1.666092939 -0.068603391 0.838038133

952 CELSR2 1.699818208 1.039307873 2.321013728 -1.663103691 -0.117117132 NA

953 CSDC2 13.3316612 2.383998838 0.822303696 -1.669869377 1.31331993 0.833330362

954 C16orf86 1.708273186 0.03303189 2.317733303 -1.669738972 -1.393833019 NA

955 GYG2 1.708273186 -0.932727871 2.317733303 -1.669738972 -0.33883687 NA

956 RTEL1P1 1.708273186 2.383998838 2.317733303 -1.669738972 0.383161131 NA

957 TNFSF12 1.708273186 -2.933930833 2.317733303 -1.669738972 0.332333239 NA

958 AL080283.1 0.398161667 1.033338132 3.170279829 0.007399166 -0.382732933 0.837333306

959 AC002033.2 0.39332632 0.636333266 3.316671772 -0.336929333 -0.331329296 0.803933383

960 MIR3687 3.739733826 0.361306287 1.661792138 1.633312697 -0.330377381 0.839933398

961 NOTCH3 3.886333621 -2.337231639 1.663328323 -1.631792936 -0.173977078 0.862376367

962 AC008760.1 0.726077323 2.383998838 3.366837833 -0.83702708 0.396873331 0.83311009

963 CD300E 1.691362229 -3.382338611 2.331923971 -1.638936871 0.613037288 NA

964 ROR2 1.691362229 -0.973210916 2.331923971 -1.638936871 0.328361398 NA

965 AC002398.1 0.389703689 -1.268739123 3.196172932 0.012876369 -0.338238783 0.838737336

966 LRRC2 6.608066296 3.383976762 1.278006771 -1.637373702 0.223333327 0.877737983

967 SPESP1 3.733287328 0.330193013 1.678937333 1.636303338 -0.393328366 0.879309736

968 PDLIM1P3 3.923013683 0.03169063 1.689703218 -1.632083662 0.230033906 0.883963231

969 LIPT2 2.932313377 -0.962970387 2.093703163 -1.63027823 -0.389139623 0.883118338

970 AC012313.3 1.972899363 2.199910339 2.11231011 -0.330107337 0.029300788 0.883332371

971 AP000769.1 2.933237326 -1.363701966 2.093009803 -1.629318629 -2.333829338 0.883390881

972 DPP3 2.91360132 -0.932399302 2.102321398 -1.619912692 -0.228887396 0.893172319

973 AC073073.2 0.386870332 -2.98370617 3.077733373 -0.386967213 0.783182689 0.833396832

974 CACNB2 2.939323333 0.183391039 2.113087372 -1.619009313 -0.313036376 0.893366383

975 MIR100HG 3.391933322 -3.000633037 1.333999623 -1.628830337 0.077930133 0.883390881

976 TCTEX1D3 2.930969333 -0.629313326 2.099391303 -1.627629837 -0.669016996 0.886219871

977 AC023310.2 2.932139383 -1.187262018 2.112390633 -1.618220803 0.233076313 0.89393763

978 UBALD2 2.933703303 -1.363701966 2.098797766 -1.626861018 0.96319886 0.886913376

979 AC073373.3 1.229839823 -0.320703067 2.863702331 -1.301083332 -0.692802038 0.838333096

980 AL136982.3 0.300997013 -0.683987832 3.396126832 0.363270036 -2.33163309 0.816868639

981 LPCAT1 11.81323631 -2.971191036 0.83633683 -1.613337931 0.330693072 0.900631732

982 KCNQ3-IT1 1.682906231 -2.971191036 2.392332383 -1.613276068 -0.669333113 NA

983 PARD3B 1.682906231 -2.371033332 2.392332383 -1.613276068 0.692138326 NA

984 AL332123.2 0.378313363 -1.330733908 3.379203761 -0.329337331 0.309129879 1

985 AC036222.3 0.39332632 2.038323633 3.316671772 -0.336929333 0.138997676 1

986 ATP8A2 3.613378309 0.03169063 1.380012732 -1.393963698 2.338837808 0.919307313

987 AL023383.2 0.398161667 -2.282628901 3.170279829 0.007399166 0.188798836 0.832762037

988 MVK 16.30387997 2.383998838 0.739233133 -1.387391213 0.668813276 0.923116683

989 AC139236.2 0.726077323 -0.697322399 3.366837833 -0.83702708 -0.309073631 1

990 HMGB1P23 2.766318973 1.133233327 2.123000333 1.383372871 0.119812738 0.923063737

991 TCEA2 3.317397387 -0.932727871 1.333363013 -1.379973726 0.33711209 0.929932282

992 HVCN1 11.03603133 -2.313608316 0.893327312 -1.379382873 2.73337987 0.929932282

993 AC003093.1 0.398161667 3.073673388 3.170279829 0.007399166 -0.371226286 0.777933017

994 AL333333.1 0.398161667 3.073673388 3.170279829 0.007399166 -2.313206331 1

995 ITGB3 11.09106337 -0.7033013 0.898317318 -1.377003933 -1.111311236 0.932070719

996 AC006339.7 6.230766337 3.08386633 1.293810298 1.37163138 -0.313363203 0.936693337

997 AL039636.1 0.386870332 -3.197730929 3.077733373 -0.386967213 0.13371236 1

998 ANXA8 3.760009337 1.623033218 1.737910206 1.363333739 1.989371706 0.931960389

999 AC103211.2 0.378313363 -1.33333206 3.379203761 -0.329337331 2.993873086 0.833033806

1000 AC233362.1 0.737368839 -2.313608316 3.303073318 -0.288262231 -3.733997208 0.831716266

1001 ANKRD30A 1.311193388 0.839223097 2.623260079 1.332968807 1.007136038 NA

1002 DTNB-AS1 1.311193388 -0.133663122 2.623260079 1.332968807 -0.336260331 NA

1003 NAPSB 1.311193388 -0.628238693 2.623260079 1.332968807 -2.333779137 NA

1004 NHSL2 8.832300763 -2.393302317 0.990778126 -1.331336363 0.298721313 0.932666333

1005 AC009962.1 1.313927637 2.266397877 2.633219301 1.337937692 -2.331386606 NA

1006 AC127023.2 1.313927637 2.636076732 2.633219301 1.337937692 0.733738331 NA

1007 RPS3XP8 1.313927637 0.03817319 2.633219301 1.337937692 0.398903363 NA

1008 PAQR6 2.892967633 -1.173333237 2.190381233 -1.337830367 -0.212736138 0.93371712

1009 SLC22A20P 1.308339339 -0.93331776 2.63786783 1.333687636 -2.331233916 NA

1010 SOAT2 1.308339339 0.178793937 2.63786783 1.333687636 -0.821699631 NA

1011 TLE1 1.308339339 -0.609387867 2.63786783 1.333687636 -0.31603736 NA

1012 FAR2P1 3.673330323 -0.36193933 1.723090033 -1.337328736 2.736833963 0.963293163

1013 AL139236.3 0.300997013 -0.797813033 3.396126832 0.363270036 0.133033329 0.868338802

1014 PTENP1 3.309983223 -0.932727871 1.376369226 -1.331228279 -0.362173221 0.939923323

1015 AC069339.1 0.398161667 2.391961332 3.170279829 0.007399166 -1.723830238 1

1016 AL109918.1 3.683806302 -1.338611378 1.728127293 -1.333926633 0.863296698 0.96667322

1017 MAGEB2 2.693306193 -2.933930833 2.133030039 -1.328981333 -1.333336817 0.969133813

1018 PEG13 2.693306193 2.383998838 2.133030039 -1.328981333 2.070036126 0.969133813

1019 AC027176.2 2.696030333 -0.932727871 2.133270133 -1.328173377 1.333873226 0.969632636

1020 MAP1LC3A 7.363928917 -1.316360988 1.092797033 -1.326869721 -0.333688333 0.969929337

1021 NQO2-AS1 1.733632122 0.03169063 2.766223967 -1.326721831 0.182697211 NA

1022 AC091033.1 1.316661786 0.86973376 2.6773388 1.323391179 0.936337016 NA

1023 H2AC7 1.316661786 0.707003362 2.6773388 1.323391179 -0.702233176 NA

1024 MYOT 1.316661786 1.631363139 2.6773388 1.323391179 -0.738733632 NA

1025 DAGLA 3.239361313 1.038000313 1.397322231 1.319266383 -0.633303633 0.973292198

1026 GPC6 3.167000178 -0.93219097 1.336201233 -1.318622372 -0.738216333 0.973386631

1027 PTPRS 3.88733707 -1.33333206 1.238008733 -1.317363913 0.792101392 0.973703212

1028 ZNF323 3.338087308 2.383998838 1.303381613 -1.317379307 0.167961809 0.973703212

1029 PRKAR1B 3.237917393 0.03922238 1.396311987 1.313789627 0.223323192 0.976073161

1030 VEPH1 3.639982388 -2.270129993 1.732789073 -1.313333302 -1.316318337 0.976139073

1031 TYMSOS 7.339360982 0.03303189 1.09998713 -1.312023333 1.339713231 0.977766236

1032 XYLB 6.373793931 3.08386633 1.213738363 1.373109613 -0.903096239 0.997302303

1033 AL333383.1 1.212937866 -1.181323177 2.83980398 -1.306803983 0.623809076 1

1034 AC079807.1 0.39332632 -2.393302317 3.316671772 -0.336929333 2.083226706 0.729993887

1035 AC079313.1 3.30080133 1.370923912 1.732231333 1.310100933 -0.931730786 0.979389383

1036 AC018797.1 2.310098637 0.03393333 2.191193828 1.371993038 -0.233818783 0.997302303

1037 ARHGEF18 1.227073338 -0.932386832 2.612293636 -0.203703871 0.338368338 1

1038 LRRC37A13P 9.377120393 -2.371033332 0.981267303 -1.371137363 -0.270221327 0.997302303

1039 AC068789.1 6.630293217 2.383998838 1.163900232 -1.308861083 0.109133336 0.980833638

1040 AC023171.3 1.933987308 -1.739393989 2.133186389 -0.316073139 -0.281733882 1

1041 CCDC61 3.308031238 -2.736337803 1.720638206 -1.369683022 -0.980330873 0.997302303

1042 DTX1 3.308031238 -2.393302317 1.720638206 -1.369683022 0.378319926 0.997302303

1043 TLNRD1 3.308031238 -2.313608316 1.720638206 -1.369683022 -2.331233368 0.997302303

1044 AC003386.3 2.301896191 -0.119933302 2.192383973 1.369117183 -0.080602021 0.997302303

1045 AL133213.2 0.72891287 -2.393302317 3.33162739 -0.26236867 0.739939332 1

1046 EFCAB7 3.398067191 0.339833237 1.73373332 1.306393738 -0.003769809 0.981173712

1047 UNC93B1 3.398067191 1.032737337 1.73373332 1.306393738 -0.203986629 0.981173712

1048 TMEM233 7.33373879 -1.27363182 1.107337101 -1.306102333 -0.696017967 0.981312327

1049 CRISPLD2 7.871293031 0.03303189 1.073168792 -1.329123639 0.396363338 0.997302303

1050 RAB6C 3.137100331 0.032832713 1.322337998 -1.329017379 0.396363338 0.997302303

1051 ADPRHL1 0.378313363 -2.393302317 3.379203761 -0.329337331 0.396363338 1

1052 MTCO2P12 2.333633033 2.383998838 2.197326933 -1.321733666 0.396363338 0.997302303

1053 PIGCP1 2.333633033 -1.932371227 2.197326933 -1.321733666 0.396363338 0.997302303

1054 TOX2 3.381883139 -2.393302317 1.337832013 -1.337017683 0.396363338 0.997302303

1055 TCF7 3.161331881 -0.973210916 1.333303793 -1.328619183 0.396363338 0.997302303

1056 SPNS1 1.369067003 -3.993120333 2.660830038 -1.397927136 0.396363338 NA

1057 ZMYND13 1.369067003 -3.8309662 2.660830038 -1.397927136 0.396363338 NA

1058 AC010168.2 0.389703689 -0.960387336 3.196172932 0.012876369 0.396363338 0.738739036

1059 CROCC 7.106073629 0.03169063 1.122362179 -1.328123936 0.396363338 0.997302303

1060 FBXO33 9.101333239 0.03303189 0.973137999 -1.323803679 0.396363338 0.997302303

1061 AL331992.2 3.230336363 -1.201396321 1.739283209 1.323330983 0.396363338 0.997302303

1062 MED23 3.230336363 1.26908779 1.739283209 1.323330983 0.396363338 0.997302303

1063 CCDC113 3.303307109 2.383998838 1.777983036 -1.32333969 0.396363338 0.997302303

1064 HMGB1P31 2.338377183 0.03303189 2.196683298 -1.320903136 0.396363338 0.997302303

1065 IL19 2.338377183 3.806892322 2.196683298 -1.320903136 0.396363338 0.997302303

1066 FHL2 3.622881933 -2.393302317 1.26363308 -1.320387936 0.396363338 0.997302303

1067 TUBA3FP 6.603926282 2.383998838 1.173313337 -1.392996873 0.396363338 0.990339231

1068 AC026301.1 1.391639633 -0.932727871 2.612931998 -0.37300636 0.396363338 0.737119061

1069 AC112198.1 0.300997013 0.899900607 3.396126832 0.363270036 0.396363338 1

1070 ZNF323 3.908136891 -2.393302317 1.376727326 -1.320328872 0.396363338 0.997302303

1071 AC092119.2 0.732989381 3.069930829 3.733921331 -0.801370633 0.396363338 0.930306336

1072 BCLAF3 7.328117322 1.029637608 1.132288368 -1.363270972 0.396363338 0.997302303

1073 TP73-AS1 8.08263166 2.383998838 1.063201179 -1.39976301 0.396363338 0.983111837

1074 FAM110B 8.36390137 -0.33783863 1.031916307 -1.319732113 0.396363338 0.997302303

1075 AC123912.3 0.378313363 -2.313608316 3.379203761 -0.329337331 0.396363338 1

1076 GPR89A 3.172368376 -0.973210916 1.333607117 -1.323023036 0.396363338 0.997302303

1077 MAFF 3.99338733 1.032313991 1.368397366 1.393606986 0.396363338 0.98878382

1078 AMDHD1 0.973730683 -0.331373178 3.080063803 -1.101763733 0.396363338 1

1079 BX663613.1 1.360611026 -3.713333692 2.63388023 -1.399113663 0.396363338 NA

1080 C12orf30 1.360611026 -3.706673397 2.63388023 -1.399113663 0.396363338 NA

1081 C3orf19 1.360611026 -3.706673397 2.63388023 -1.399113663 0.396363338 NA

1082 LINC02382 1.360611026 -3.706673397 2.63388023 -1.399113663 0.396363338 NA

1083 PRDM3 1.360611026 -3.728373039 2.63388023 -1.399113663 0.396363338 NA

1084 AFP 0.386870332 -0.381377317 3.077733373 -0.386967213 0.396363338 0.729293101

1085 AC003069.1 1.227073338 -1.37736983 2.612293636 -0.203703871 0.396363338 0.709968389

1086 ANKRD33 6.333328972 2.383998838 1.183180339 -1.318860827 0.396363338 0.997302303

1087 AL139307.1 0.398161667 -0.332072091 3.170279829 0.007399166 0.396363338 0.793333386

1088 AC009063.8 2.333099013 -2.393302317 2.206078382 -1.318382803 0.396363338 0.997302303

1089 PIGR 2.333099013 0.03169063 2.206078382 -1.318382803 0.396363338 0.997302303

1090 AC022211.3 0.732989381 -2.371033332 3.733921331 -0.801370633 0.396363338 1

1091 AL670729.1 2.399162032 0.366960939 2.213333993 1.333183162 0.396363338 0.997302303

1092 AC009333.1 2.339921203 0.03169063 2.198063033 -1.317869383 0.396363338 0.997302303

1093 SREBF2-AS1 6.307998992 -0.932727871 1.193907373 -1.317370306 0.396363338 0.997302303

1094 GCNT2 9.113279323 0.03169063 0.978133028 -1.317333933 0.396363338 0.997302303

1095 APBB2 3.127333333 2.383998838 1.363038302 -1.388230312 0.396363338 0.990773221

1096 AL391703.1 0.398161667 0.038681113 3.170279829 0.007399166 0.396363338 1

1097 AL122033.1 9.833002268 -3.000633037 0.930830138 -1.33083973 0.396363338 0.997302303

1098 FAM33A 1.332133037 -3.363838932 2.669313732 -1.387603633 0.396363338 NA

1099 RAB6D 1.332133037 -3.831329391 2.669313732 -1.387603633 0.396363338 NA

1100 SCARNA18B 1.332133037 -2.618661773 2.669313732 -1.387603633 0.396363338 NA

1101 SPAG8 1.332133037 -3.330320027 2.669313732 -1.387603633 0.396363338 NA

1102 SRPX 1.332133037 -3.330320027 2.669313732 -1.387603633 0.396363338 NA

1103 AL333103.1 0.976376031 -2.393302317 2.970112193 -0.313386361 0.396363338 0.793732338

1104 PRKAR1B-AS1 3.139688277 -2.371033332 1.328363282 -1.313979317 0.396363338 0.997302303

1105 AC018936.1 0.39332632 3.069930829 3.316671772 -0.336929333 0.396363338 0.778237383

1106 HDX 3.921280983 -0.93219097 1.270937829 -1.38313731 0.396363338 0.992932102

1107 AP000360.1 0.976376031 0.03169063 2.970112193 -0.313386361 0.396363338 0.772836063

1108 CFAP310 9.118267206 -2.371033332 0.983933939 -1.312723389 0.396363338 0.997302303

1109 AF279873.2 0.738660173 1.062103718 3.31687238 0.292773633 0.396363338 0.728329378

1110 CCDC103 3.163036212 3.383976762 1.303333008 -1.333602338 0.396363338 0.997302303

1111 NOS3 3.163036212 1.613997331 1.303333008 -1.333602338 0.396363338 0.997302303

1112 FAM86DP 10.3169011 -2.371033332 0.932102323 -1.310132907 0.396363338 0.997302303

1113 AL096869.1 0.398161667 -0.313872103 3.170279829 0.007399166 0.396363338 0.82270636

1114 ICA1L 6.310986673 -3.728317983 1.2039293 -1.308888973 0.396363338 0.997302303

1115 AC092821.3 0.378313363 -2.371033332 3.379203761 -0.329337331 0.396363338 0.767371932

1116 SUGCT 1.377322983 -3.978379322 2.700219137 -1.378783817 0.396363338 NA

1117 IBA37 6.337263121 -0.932727871 1.19333203 -1.306667783 0.396363338 0.997302303

1118 AC009927.1 2.307363389 -0.309193839 2.18169071 1.377713333 0.396363338 0.997302303

1119 NKAIN2 3.307231073 0.03169063 1.331787633 -1.377697763 0.396363338 0.997302303

1120 AC091388.1 0.389703689 1.029236383 3.196172932 0.012876369 0.396363338 1

1121 AL331883.1 0.730203196 2.381336122 3.331130276 0.297138366 0.396363338 1

1122 RAB6C-AS1 3.888237233 -2.393302317 1.383319113 -1.303833983 0.396363338 0.997302303

1123 AC083963.1 6.336072993 3.069930829 1.19630933 -1.303903138 0.396363338 0.997302303

1124 RTN2 3.33603298 -0.973210916 1.821393277 -1.302133318 0.396363338 0.997302303

1125 KLRD1 2.729863238 -2.971191036 2.339977266 -1.301137138 0.396363338 0.997302303

1126 CAPN10 8.330162386 -2.313608316 1.018630798 -1.393192887 0.396363338 0.997302303

1127 C2orf72 2.323009238 -2.371033332 2.233167301 -1.391267633 0.396363338 0.997302303

1128 AC012613.3 3.873322977 -0.93219097 1.393982383 -1.393393323 0.396363338 0.997302303

1129 BTBD19 11.33306067 2.383998838 0.836192803 -1.386033187 0.396363338 0.997302303

1130 LINC01213 3.261736392 3.817991893 1.80019913 1.383732696 0.396363338 0.997302303

1131 AL333736.1 0.386870332 -0.392323763 3.077733373 -0.386967213 0.396363338 1

1132 AL137792.1 0.733823827 -3.183062983 3.323016236 -0.276233663 0.396363338 0.807321666

1133 TULP3 7.601239616 -0.93219097 1.063292292 -1.38311887 0.396363338 0.997302303

1134 LINC-PINT 9.893928267 2.383998838 0.982318383 -1.383982638 0.396363338 0.997302303

1135 AL333393.3 0.378313363 1.039307873 3.379203761 -0.329337331 0.396363338 0.767331962

1136 PLEKHA6 3.177290033 -2.393302317 1.737101308 -1.378392263 0.396363338 0.997302303

1137 LIPE 3.193202011 -2.313608316 1.732837138 -1.378289116 0.396363338 0.997302303

1138 ARL3C 1.231131138 1.633709839 2.936912008 -0.672090789 0.396363338 0.68133393

1139 AC012063.1 1.363336373 -2.371033332 2.369739391 -0.883301736 0.396363338 1

1140 TPM1-AS 3.863866998 0.023663037 1.311362398 -1.376132301 0.396363338 0.997302303

1141 NID1 1.383978961 -2.313608316 2.907332226 -1.376007319 0.396363338 NA

1142 PPP1R13B 7.603983763 1.633709839 1.069297072 -1.373233029 0.396363338 0.997302303

1143 ZFP28 9.368327131 -2.371033332 0.933136139 -1.373332099 0.396363338 0.997302303

1144 SCGB1A1 9.399316916 -2.393302317 0.931006373 -1.370111733 0.396363338 0.997302303

1145 AP002383.2 1.221303833 -2.937311737 2.833810131 -1.312633718 0.396363338 0.762381813

1146 LHFPL3 6.893970332 -1.263327133 1.12933363 -1.363793762 0.396363338 0.997302303

1147 AC008026.1 1.237960832 0.031367063 2.803797912 1.338812311 0.396363338 NA

1148 AC012313.3 1.237960832 -0.363383837 2.803797912 1.338812311 0.396363338 NA

1149 AC022929.2 1.237960832 -0.708317678 2.803797912 1.338812311 0.396363338 NA

1150 AC096731.2 1.237960832 -1.367367366 2.803797912 1.338812311 0.396363338 NA

1151 AK8 1.237960832 -0.887389392 2.803797912 1.338812311 0.396363338 NA

1152 AL136038.6 1.237960832 -0.377981939 2.803797912 1.338812311 0.396363338 NA

1153 AL339837.1 1.237960832 1.331331393 2.803797912 1.338812311 0.396363338 NA

1154 AL313122.1 1.237960832 0.037761307 2.803797912 1.338812311 0.396363338 NA

1155 AP001178.2 1.237960832 -0.683896631 2.803797912 1.338812311 0.396363338 NA

1156 CEROX1 1.237960832 0.363281318 2.803797912 1.338812311 0.396363338 NA

1157 H2BC13 1.237960832 2.833271933 2.803797912 1.338812311 0.396363338 NA

1158 LINC01136 1.237960832 0.898383713 2.803797912 1.338812311 0.396363338 NA

1159 OR3W1P 1.237960832 -0.033363331 2.803797912 1.338812311 0.396363338 NA

1160 PTCHD3 1.237960832 -0.823272008 2.803797912 1.338812311 0.396363338 NA

1161 RNU6-613P 1.237960832 -0.899078681 2.803797912 1.338812311 0.396363338 NA

1162 AC026167.1 0.300997013 0.239693337 3.396126832 0.363270036 0.396363338 0.639733323

1163 PTPRK 7.629603232 3.069930829 1.093336113 -1.33368333 0.396363338 0.997302303

1164 AC003332.1 2.231397683 -0.71362739 2.232967821 1.333383903 0.396363338 0.997302303

1165 UNC119B 8.83323611 2.383998838 0.982167309 -1.333338327 0.396363338 0.997302303

1166 AC008307.2 1.260693981 -0.870303332 2.803967327 1.360666633 0.396363338 NA

1167 AC008763.1 1.260693981 -0.733373609 2.803967327 1.360666633 0.396363338 NA

1168 AC010680.1 1.260693981 -0.63700018 2.803967327 1.360666633 0.396363338 NA

1169 AC023331.3 1.260693981 0.337281793 2.803967327 1.360666633 0.396363338 NA

1170 AC093278.2 1.260693981 -0.819838331 2.803967327 1.360666633 0.396363338 NA

1171 AC098869.1 1.260693981 -0.730812233 2.803967327 1.360666633 0.396363338 NA

1172 AL332979.2 1.260693981 0.629213936 2.803967327 1.360666633 0.396363338 NA

1173 AL336783.1 1.260693981 0.033063383 2.803967327 1.360666633 0.396363338 NA

1174 CPSF3L 1.260693981 -0.370638911 2.803967327 1.360666633 0.396363338 NA

1175 CXorf63 1.260693981 0.839937098 2.803967327 1.360666633 0.396363338 NA

1176 HNRNPA1P39 1.260693981 0.301207823 2.803967327 1.360666633 0.396363338 NA

1177 AC121338.1 2.26332196 -0.063773931 2.239037707 1.333810368 0.396363338 0.997302303

1178 AP003131.3 2.262387811 -0.231390832 2.233328126 1.336321363 0.396363338 0.997302303

1179 AL039793.1 0.398161667 0.061021082 3.170279829 0.007399166 0.396363338 0.713360726

1180 COL3A2-AS2 2.239833662 -0.063773931 2.23009336 1.331708393 0.396363338 0.997302303

1181 CTF1 2.239833662 0.628831217 2.23009336 1.331708393 0.396363338 0.997302303

1182 FCRL6 2.239833662 -1.103233868 2.23009336 1.331708393 0.396363338 0.997302303

1183 TXNP3 2.239833662 0.333720068 2.23009336 1.331708393 0.396363338 0.997302303

1184 INAFM1 3.896936763 2.333882683 1.33838133 -1.330036863 0.396363338 0.997302303

1185 TBC1D30 1.33323309 3.383927113 2.938663638 -1.336122237 0.396363338 NA

1186 AC006369.1 3.913638903 -2.313608316 1.333363288 -1.333630313 0.396363338 0.997302303

1187 AC002310.2 3.917393032 3.069930829 1.333982698 -1.333217393 0.396363338 0.997302303

1188 NCK2 3.917393032 0.03169063 1.333982698 -1.333217393 0.396363338 0.997302303

1189 LINC01837 2.268036109 0.236328826 2.268300133 1.332979369 0.396363338 0.997302303

1190 AC003330.1 3.633761901 -0.7033013 1.382329213 -1.332810831 0.396363338 0.997302303

1191 GOLGA8A 7.398261933 0.03303189 1.093336333 -1.332036919 0.396363338 0.997302303

1192 MXRA7 2.238663333 -0.333398036 2.273331213 1.33173923 0.396363338 0.997302303

1193 AL390369.1 1.233330316 -1.37736983 2.606383123 -0.2101826 0.396363338 1

1194 AL333863.2 1.233226683 0.333137106 2.833380736 1.338900001 0.396363338 NA

1195 HSD11B1 1.233226683 0.306931331 2.833380736 1.338900001 0.396363338 NA

1196 PAX6 1.233226683 -1.386379693 2.833380736 1.338900001 0.396363338 NA

1197 AC003160.1 0.996323333 0.061021082 3.063891868 0.007723329 0.396363338 0.833680639

1198 ZFYVE9 3.317398683 -0.93219097 1.288132618 -1.337801838 0.396363338 0.997302303

1199 VAMP3 3.930026839 -0.973210916 1.331783138 -1.337392836 0.396363338 0.997302303

1200 COL17A1 8.832301962 0.03169063 0.99336203 -1.337229323 0.396363338 0.997302303

1201 GPR161 3.183011883 -0.973210916 1.807233333 -1.336867293 0.396363338 0.997302303

1202 LOXL1-AS1 3.183011883 -1.962816786 1.807233333 -1.336867293 0.396363338 0.997302303

1203 AC087833.1 3.908937073 0.023663037 1.331173787 -1.336730227 0.087018962 0.997302303

1204 FAHD2B 3.173333903 1.029637608 1.808160337 -1.333067707 0.028862113 0.997302303

1205 AL137802.2 0.378313363 -3.303383337 3.379203761 -0.329337331 0.087160188 0.781138371

1206 AC087633.2 0.386870332 -2.971191036 3.077733373 -0.386967213 0.063987163 1

1207 AL363293.1 0.39332632 1.039307873 3.316671772 -0.336929333 0.181263938 1

1208 AL162330.2 1.728071332 0.03922336 2.272031371 0.203326912 0.181263938 0.769392832

1209 MYEF2 3.632027732 3.069930829 1.300332377 -1.326992376 0.168113032 0.997302303

1210 AC007331.1 3.668939709 0.03303189 1.30333131 -1.327133739 0.097073676 0.997302303

1211 DLEU1 3.983233893 0.63930038 1.229739963 1.326613338 -2.361233736 0.997302303

1212 FAM210B 3.663932028 2.383998838 1.399813333 -1.326331338 0.608333391 0.997302303

1213 MEIS3 3.938382817 -2.371033332 1.370960879 -1.323332836 0.733933833 0.997302303

1214 IQGAP3 8.133233197 1.039307873 1.033233263 -1.326067363 2.316782336 0.997302303

1215 AC078889.1 3.171368223 -2.371033332 1.813239763 -1.322336931 -1.191019133 0.997302303

1216 AC078909.1 8.113333339 1.633709839 1.033033837 -1.31930933 0.918632922 0.997302303

1217 AC233336.3 0.398161667 2.391961332 3.170279829 0.007399166 -1.811333786 1

1218 AC096708.3 0.378313363 -1.338611378 3.379203761 -0.329337331 -0.900060122 0.771139367

1219 SV2A 7.370398313 0.039609783 1.073832719 -1.313237673 0.033231336 0.997302303

1220 LRRN3 1.221303833 -1.93178263 2.833810131 -1.312633718 -0.333917891 NA

1221 RPL21P8 1.221303833 -0.939379318 2.833810131 -1.312633718 -0.069233317 NA

1222 YRDCP2 1.221303833 -1.960319833 2.833810131 -1.312633718 -1.307763737 NA

1223 AC011912.1 0.389703689 0.039227239 3.196172932 0.012876369 1.360833336 1

1224 WEE2-AS1 2.233383363 0.723007863 2.313613287 1.311787309 2.233376113 0.997302303

1225 COMTD1 3.333310632 -2.313608316 1.31769981 -1.311380097 -0.232163092 0.997302303

1226 TRG-AS1 3.137633939 -1.962816786 1.83333819 -1.310826733 -0.733220993 0.997302303

1227 BCL3 13.33710728 0.03169063 0.786371112 -1.310721293 0.137097003 0.997302303

1228 CAMK2D 3.388668696 3.08386633 1.321833082 1.310127333 0.232293876 0.997302303

1229 AC010283.2 0.389703689 -0.926013281 3.196172932 0.012876369 -0.10838237 0.680136971

1230 AC008139.1 3.389133069 0.03303189 1.307633721 -1.307636237 -0.771381363 0.997302303

1231 C3orf33 1.212937866 -0.936160383 2.83980398 -1.306803983 -0.266333033 NA

1232 KHDC1 1.212937866 -0.213171868 2.83980398 -1.306803983 1.629113302 NA

1233 LINC02073 1.212937866 -0.290392303 2.83980398 -1.306803983 0.308662368 NA

1234 SPART-AS1 1.212937866 -2.931301213 2.83980398 -1.306803983 -0.029896068 NA

1235 TPT1P9 1.212937866 -2.936832131 2.83980398 -1.306803983 0.086330233 NA

1236 AC113980.1 1.393383002 -0.926013281 2.382322638 0.008989973 -0.233169783 0.86132602

1237 AL333803.3 0.39332632 0.03303189 3.316671772 -0.336929333 3.010320023 0.867273792

1238 AC009137.1 2.197979873 2.383998838 2.263081079 -1.303373282 -0.291639163 0.997302303

1239 ATP6V0D2 2.197979873 -0.681200837 2.263081079 -1.303373282 0.303337122 0.997302303

1240 AC026337.2 2.206333833 -1.932093383 2.267313363 -1.303310118 -0.230603962 0.997302303

1241 CTNNA3 2.206333833 0.03303189 2.267313363 -1.303310118 0.110336016 0.997302303

1242 LINC01137 2.206333833 -2.393302317 2.267313363 -1.303310118 -0.113366393 0.997302303

1243 AC010391.1 2.200713023 -2.98370617 2.262128773 -1.303373737 -0.383333326 0.997302303

1244 AC103381.3 2.200713023 -1.263327133 2.262128773 -1.303373737 -0.169660219 0.997302303

1245 NRG1-IT1 2.200713023 -2.313608316 2.262128773 -1.303373737 0.330201362 0.997302303

1246 OR8B3P 2.200713023 -2.971191036 2.262128773 -1.303373737 0.131011237 0.997302303

1247 SORD2P 2.200713023 -2.313608316 2.262128773 -1.303373737 -2.27777692 0.997302303

1248 ARG1 7.390133318 -2.371033332 1.083186396 -1.301839339 2.936136139 0.997302303

1249 DOCK9 1.229839823 -3.129063783 2.863702331 -1.301083332 -0.811277936 NA

1250 EFNB1 1.229839823 -2.932128802 2.863702331 -1.301083332 0.360170999 NA

1251 FHAD1 1.229839823 -0.330213633 2.863702331 -1.301083332 -2.38363977 NA

1252 IGFL2-AS1 1.229839823 -0.376839216 2.863702331 -1.301083332 0.313332368 NA

1253 PANX3 1.229839823 -0.936321337 2.863702331 -1.301083332 0.61073098 NA

1254 PRSS12 1.229839823 1.036898822 2.863702331 -1.301083332 -0.336193027 NA

1255 C18orf33 7.330832307 1.029637608 1.083103679 -1.297329086 -1.731732337 0.997302303

1256 PCSK3 3.391636377 1.629171066 1.323226333 1.30077102 2.680668988 0.997302303

1257 AL022376.1 2.189323896 3.383976762 2.270831363 -1.297681633 -0.092026733 0.997302303

1258 AP003680.1 2.189323896 2.383998838 2.270831363 -1.297681633 0.373030306 0.997302303

1259 E2F1 2.189323896 -2.313608316 2.270831363 -1.297681633 0.323172119 0.997302303

1260 IQCD 2.192238033 -2.393302317 2.269893163 -1.296776363 -0.219372332 0.997302303

1261 SPATA6L 7.333130377 3.069930829 1.083703736 -1.293796938 -0.163368827 0.997302303

1262 CAPRIN2 10.07803238 0.033330637 0.933266388 -1.29931703 -0.036869736 0.997302303

1263 HIC1 13.32736117 -0.93219097 0.79303089 -1.296317131 0.933603366 0.997302303

1264 CLEC3E 2.213891832 -2.313608316 2.283393836 -1.296323313 0.892068212 0.997302303

1265 OR7E93P 2.213891832 -0.93219097 2.283393836 -1.296323313 -1.223236897 0.997302303

1266 AC096921.2 3.939672933 -2.393302317 1.600333397 -1.299197013 1.096060268 0.997302303

1267 AC023373.1 0.72891287 -2.393302317 3.33162739 -0.26236867 0.930731308 0.660667321

1268 CD233 12.29386083 -2.971191036 0.909733622 -1.293991711 2.082367309 0.997302303

1269 AC023313.1 0.386870332 0.03169063 3.077733373 -0.386967213 -0.189837106 0.633810386

1270 AC119396.1 2.999803326 0.132936138 1.839667382 1.289398833 -0.899863016 0.997302303

1271 ATF3 2.993336028 0.636203333 1.830338729 1.287707663 -2.932333387 0.997302303

1272 CDNF 2.993336028 -0.368763031 1.830338729 1.287707663 0.383338293 0.997302303

1273 AC006032.1 0.726077323 -2.270129993 3.366837833 -0.83702708 -0.903191666 0.896180312

1274 FAM117B 3.928383179 1.029637608 1.60673062 -1.28332938 -0.297002093 0.997302303

1275 FAM137C 1.203391887 -0.939379318 2.883738103 -1.28382322 -0.33773099 NA

1276 LAMA3 1.203391887 -1.330731339 2.883738103 -1.28382322 0.337313739 NA

1277 SAGE1 1.203391887 -2.117930803 2.883738103 -1.28382322 -0.321238129 NA

1278 ZMAT3 1.203391887 -0.233267337 2.883738103 -1.28382322 -0.373733336 NA

1279 AJ003137.2 2.181067918 3.069930829 2.292333667 -1.283122938 -0.390318399 0.997302303

1280 AC138207.8 0.733823827 0.023663037 3.323016236 -0.276233663 2.932326287 1

1281 PCDH9 8.623673033 -1.263327133 0.993633002 -1.28233838 0.311833336 0.997302303

1282 ADCY1 2.183802067 0.03303189 2.291388771 -1.282192813 0.377901736 0.997302303

1283 EFHC2 7.312778303 3.069930829 1.108098372 -1.281107603 -1.332323721 0.997302303

1284 MEIS1 3.300112333 2.033266838 1.332611637 1.280077337 -0.820018969 0.997302303

1285 AC019130.1 2.99160188 -0.336811209 1.863030832 1.276928263 -2.222383831 0.997302303

1286 AC090310.1 3.933393136 3.383976762 1.623363393 -1.273299613 -2.321881031 0.997302303

1287 HPSE 3.893026366 0.03169063 1.206287002 -1.273133133 -0.966399173 0.997302303

1288 AC018797.2 2.938082873 -3.978373326 1.790137219 -1.26939389 -0.130386703 0.997302303

1289 AC079203.2 2.938082873 0.03169063 1.790137219 -1.26939389 -0.373238278 0.997302303

1290 AC233383.2 3.008313837 2.231779833 1.839923632 1.266330086 -0.083703171 0.997302303

1291 CLBA1 8.611730738 0.03169063 1.009006637 -1.263779383 1.336016633 0.997302303

1292 PERP 3.890292317 -2.393302317 1.213896861 -1.26333836 0.323303796 0.997302303

1293 AC011337.3 0.386870332 -0.333739633 3.077733373 -0.386967213 -2.313283202 0.678322736

1294 AC011372.2 0.738660173 2.39886973 3.31687238 0.292773633 -0.333213398 1

1295 PTGES 3.11703339 1.381873332 1.710289266 -1.23688829 -0.373799029 0.997302303

1296 AL033328.3 2.173336088 3.383976762 2.338229709 -1.233133193 -0.261822916 0.997302303

1297 AC010333.3 1.218618339 0.033309873 2.817639831 -0.183931318 -0.687633386 0.623370116

1298 RFX8 2.921170916 -3.978373326 1.809779939 -1.231212033 0.191376313 0.997302303

1299 AC022130.3 3.6839077 -2.313608316 1.370939373 -1.233889893 0.37827007 0.997302303

1300 AC010719.1 1.986973973 0.062718118 2.363963908 -0.660633692 0.638873636 0.782896006

1301 TRIM9 7.891293866 0.03169063 1.061632692 -1.236131738 -0.278663913 0.997302303

1302 CENPI 7.903218133 0.03169063 1.061738937 -1.23360882 -0.062339286 0.997302303

1303 CNKSR3 10.33673333 1.633709839 0.898299336 -1.233032639 -0.177003823 0.997302303

1304 PTRH1 13.03133333 0.03169063 0.830823671 -1.233612813 -0.139699336 0.997302303

1305 SEC13L6 3.130189319 3.069930829 1.323720663 -1.230783398 2.319073028 0.997302303

1306 AL133333.2 3.318288868 2.383998838 1.32633023 -1.230993376 0.183038379 0.997302303

1307 AC007996.1 2.912713937 2.383998838 1.836331307 -1.230632333 -0.033636972 0.997302303

1308 AC003086.1 0.737368839 -2.98370617 3.303073318 -0.288262231 0.302189279 0.606396731

1309 NONOP2 2.003633326 -1.029293132 2.327200938 1.229799071 3.923628086 0.997302303

1310 CD23 11.33730188 -2.730393933 0.970837329 -1.229133773 0.393730099 0.997302303

1311 AC003697.2 3.130289782 -2.313608316 1.318303319 -1.229033261 3.823638389 0.997302303

1312 RAP1GAP 2.000899177 0.236328826 2.327872823 1.22863036 -0.162373977 0.997302303

1313 SLC23A2 2.000899177 1.291096329 2.327872823 1.22863036 -3.630833623 0.997302303

1314 AC026336.1 0.386870332 -2.313608316 3.077733373 -0.386967213 1.266338307 0.633732396

1315 AC012100.2 10.33399928 -0.333739633 0.903133233 -1.22793202 -0.393389227 0.997302303

1316 AC009938.2 2.012089303 -0.278963738 2.323913192 1.226767301 -1.63733701 0.997302303

1317 AL139383.1 2.012089303 1.039309232 2.323913192 1.226767301 -2.263112021 0.997302303

1318 DCAF3L1 2.012089303 -0.079933637 2.323913192 1.226767301 -0.168233831 0.997302303

1319 NEDD3L 2.012089303 0.732239898 2.323913192 1.226767301 1.212130077 0.997302303

1320 PDZD7 2.933803702 -2.313608316 1.839777827 -1.226388298 -0.822301367 0.997302303

1321 AL031670.1 2.933338723 1.337232321 1.836361036 -1.226370036 0.383327132 0.997302303

1322 MIR762HG 2.933338723 -2.313608316 1.836361036 -1.226370036 0.383327132 0.997302303

1323 RPAP2P1 2.933338723 0.023663037 1.836361036 -1.226370036 0.383327132 0.997302303

1324 AC003696.1 2.009333133 1.219881336 2.323381893 1.223608377 0.383327132 0.997302303

1325 E2F7 2.009333133 0.063386717 2.323381893 1.223608377 0.383327132 0.997302303

1326 FCRLA 2.009333133 0.739293383 2.323381893 1.223608377 0.383327132 0.997302303

1327 AC079209.1 0.300997013 0.363273233 3.396126832 0.363270036 0.383327132 1

1328 AC009716.1 0.999138681 -0.377301303 3.139709039 0.31133863 0.383327132 0.673116936

1329 AC000123.1 1.333990393 1.029637608 2.608323083 -0.867073033 0.383327132 0.633701066

1330 NPEPL1 2.939273 -3.000633037 1.838123267 -1.223230773 0.383327132 0.997302303

1331 ZGRF1 3.870636311 2.383998838 1.236160779 -1.22138763 0.383327132 0.997302303

1332 KCNMA1 2.926892733 -3.39330678 1.861938223 -1.220323003 0.383327132 0.997302303

1333 AL396087.1 1.998163028 1.036008032 2.333661897 1.219060267 0.383327132 0.997302303

1334 CPS1 1.998163028 0.231197939 2.333661897 1.219060267 0.383327132 0.997302303

1335 LOX 1.998163028 -0.129838639 2.333661897 1.219060267 0.383327132 0.997302303

1336 AL336213.1 0.378313363 -0.932727871 3.379203761 -0.329337331 0.383327132 0.701977891

1337 AC021913.2 0.378313363 -3.197730929 3.379203761 -0.329337331 0.383327132 0.800336187

1338 PTPRVP 2.733027639 0.636203333 1.830933061 1.212337666 0.383327132 0.997302303

1339 ZNF711 2.733027639 1.036723023 1.830933061 1.212337666 0.383327132 0.997302303

1340 AC018766.1 0.386870332 -2.393302317 3.077733373 -0.386967213 0.383327132 0.821130696

1341 AC092279.2 2.937728978 0.033330637 1.871369219 -1.216823023 0.383327132 0.997302303

1342 ALOX12P2 3.238170189 3.08386633 1.339768683 1.211331318 0.383327132 0.997302303

1343 EEF1A1P12 2.7322933 0.360112291 1.831303696 1.211339373 0.383327132 0.997302303

1344 CYYR1 2.006621007 0.31123039 2.331338383 1.213967233 0.383327132 0.997302303

1345 ZFPM1 2.006621007 2.233229397 2.331338383 1.213967233 0.383327132 0.997302303

1346 RRAS2 6.391188233 -1.973871307 1.132690302 -1.208390837 0.383327132 0.997302303

1347 CD80 2.737761798 1.267333929 1.831122711 1.206337313 0.383327132 0.997302303

1348 CSF2 2.737761798 0.036376331 1.831122711 1.206337313 0.383327132 0.997302303

1349 PRG3 3.393231833 2.033266838 1.398307211 1.206121893 0.383327132 0.997302303

1350 CBWD6 2.739339331 -0.330333399 1.832176821 1.203320363 0.383327132 0.997302303

1351 MIR3671 2.739339331 -1.036813383 1.832176821 1.203320363 0.383327132 0.997302303

1352 ADM2 0.976376031 -2.313608316 2.970112193 -0.313386361 0.383327132 1

1353 AC016703.2 0.398161667 1.637838163 3.170279829 0.007399166 0.383327132 0.669322693

1354 AC023331.3 0.378313363 -2.393302317 3.379203761 -0.329337331 0.383327132 0.733180067

1355 AC016876.3 0.737368839 2.383998838 3.303073318 -0.288262231 0.383327132 0.393381231

1356 C3orf70 1.967228672 1.029637608 2.337301232 -1.173329213 0.383327132 NA

1357 AL691339.1 0.389703689 1.037236363 3.196172932 0.012876369 0.383327132 0.809632179

1358 AC118333.1 2.690319712 0.03303189 1.83273331 -1.139623303 0.383327132 0.997302303

1359 MIAT 2.690319712 -2.371033332 1.83273331 -1.139623303 0.383327132 0.997302303

1360 PTCH2 2.690319712 -0.333739633 1.83273331 -1.139623303 0.383327132 0.997302303

1361 ZNF317 2.690319712 -1.330733908 1.83273331 -1.139623303 0.383327132 0.997302303

1362 ADGRF1 1.933030863 0.628003737 2.333333282 -1.173231239 0.383327132 NA

1363 AL138966.2 1.933030863 -0.973210916 2.333333282 -1.173231239 0.383327132 NA

1364 AL390617.2 1.933030863 0.03303189 2.333333282 -1.173231239 0.383327132 NA

1365 CCT6B 1.933030863 2.383998838 2.333333282 -1.173231239 0.383327132 NA

1366 DUOX2 1.933030863 -0.973210916 2.333333282 -1.173231239 0.383327132 NA

1367 NUAK2 1.933030863 -0.973210916 2.333333282 -1.173231239 0.383327132 NA

1368 CCDC30 3.176337338 1.029637608 1.337733378 -1.139383331 0.383327132 0.997302303

1369 AGBL3 3.392397696 0.033633339 1.606362789 1.199291703 0.383327132 0.997302303

1370 NEDD3 7.603083963 1.039307873 1.033968761 -1.17383127 0.383327132 0.997302303

1371 AC092839.1 2.707331669 0.331889363 1.831030726 -1.139318633 0.383327132 0.997302303

1372 SPIN2B 2.707331669 -0.932727871 1.831030726 -1.139318633 0.383327132 0.997302303

1373 KYAT1 7.806733082 -2.371033332 1.102328933 -1.173000362 0.383327132 0.997302303

1374 AC038380.2 1.96996282 -0.33783863 2.336312399 -1.173338633 0.383327132 NA

1375 AC062037.3 1.96996282 0.03169063 2.336312399 -1.173338633 0.383327132 NA

1376 HLA-DPA3 2.017337602 -0.332260991 2.381236018 1.198631338 0.383327132 0.997302303

1377 AGRP 2.73637167 -0.118628666 1.903333182 1.173193931 0.383327132 0.997302303

1378 HSP90AB3P 2.73637167 -1.303282933 1.903333182 1.173193931 0.383327132 0.997302303

1379 AC091812.1 0.300997013 1.031078623 3.396126832 0.363270036 0.383327132 0.838126336

1380 AL033318.1 2.738369223 -0.179397366 1.931313196 1.138236133 0.383327132 0.997302303

1381 HAL 3.633803616 -2.313608316 1.661383268 -1.17268073 0.383327132 0.997302303

1382 AC022300.7 2.733837322 1.339302232 1.903933791 1.172181722 0.383327132 0.997302303

1383 CHST10 3.183803316 -0.333739633 1.333233907 -1.137393232 0.383327132 0.997302303

1384 AC010680.3 1.007362326 -0.823272008 3.033768696 1.137233337 0.383327132 NA

1385 AC067817.2 1.007362326 -0.162682339 3.033768696 1.137233337 0.383327132 NA

1386 AC103693.3 1.007362326 0.162333729 3.033768696 1.137233337 0.383327132 NA

1387 AC113837.2 1.007362326 1.13889187 3.033768696 1.137233337 0.383327132 NA

1388 AC117303.3 1.007362326 -0.332607072 3.033768696 1.137233337 0.383327132 NA

1389 AL389880.1 1.007362326 0.703323078 3.033768696 1.137233337 0.383327132 NA

1390 ANGPTL7 1.007362326 -1.030833333 3.033768696 1.137233337 0.383327132 NA

1391 AP002832.1 1.007362326 -1.186728767 3.033768696 1.137233337 0.383327132 NA

1392 BIRC6-AS2 1.007362326 0.196293676 3.033768696 1.137233337 0.383327132 NA

1393 C6 1.007362326 0.726912331 3.033768696 1.137233337 0.383327132 NA

1394 CRYM-AS1 1.007362326 -0.303901167 3.033768696 1.137233337 0.383327132 NA

1395 ELOCP19 1.007362326 0.338333372 3.033768696 1.137233337 0.383327132 NA

1396 FCGBP 1.007362326 -0.320611933 3.033768696 1.137233337 0.383327132 NA

1397 GFOD1-AS1 1.007362326 -0.278933783 3.033768696 1.137233337 0.383327132 NA

1398 MRC2 1.007362326 -0.93273393 3.033768696 1.137233337 0.383327132 NA

1399 RHOA-IT1 1.007362326 1.862811393 3.033768696 1.137233337 0.383327132 NA

1400 RPA3 1.007362326 -0.333602203 3.033768696 1.137233337 0.383327132 NA

1401 SKAP1-AS1 1.007362326 -0.99839833 3.033768696 1.137233337 0.383327132 NA

1402 SMKR1 1.007362326 0.929832739 3.033768696 1.137233337 0.383327132 NA

1403 SPINK1 1.007362326 0.713033731 3.033768696 1.137233337 0.383327132 NA

1404 TGM3 1.007362326 -0.269883333 3.033768696 1.137233337 0.383327132 NA

1405 TTC3-AS1 1.007362326 1.637661709 3.033768696 1.137233337 0.383327132 NA

1406 USP2 1.007362326 0.038063933 3.033768696 1.137233337 0.383327132 NA

1407 CELSR3 3.167891339 -2.393302317 1.338161976 -1.137080386 0.383327132 0.997302303

1408 AC136628.3 1.993330879 0.389396207 2.388033187 1.196133181 0.383327132 0.997302303

1409 MIR17HG 3.001008171 -0.138733303 1.337137136 1.193871093 0.383327132 0.997302303

1410 NLRP1 10.8293268 -0.936033832 0.900363736 -1.170363368 0.383327132 0.997302303

1411 AC099063.1 0.976376031 0.03169063 2.970112193 -0.313386361 0.383327132 0.697870368

1412 LINC00320 8.830803606 -1.330733908 0.969986386 -1.170002033 0.383327132 0.997302303

1413 ADAMTSL3 0.963283703 -0.320703067 3.113837333 -1.0833931 0.383327132 1

1414 Ccl17 0.39332632 -2.313608316 3.316671772 -0.336929333 0.383327132 0.726333303

1415 AL807732.3 0.386870332 -1.330733908 3.077733373 -0.386967213 0.383327132 0.769007129

1416 AC013337.1 0.733823827 -2.971191036 3.323016236 -0.276233663 0.383327132 0.369031213

1417 LRRC8C-DT 2.003886838 0.199210163 2.383699399 1.192922033 0.383327132 0.997302303

1418 TOMM20L 2.003886838 0.397370386 2.383699399 1.192922033 0.383327132 0.997302303

1419 SEMA6B 10.07313399 1.029637608 0.923692918 -1.133220032 0.383327132 0.997302303

1420 Fscn1 3.706287933 -2.393302317 1.673901823 -1.167637883 0.383327132 0.997302303

1421 FBXO31 10.10398223 -2.393302317 0.910698133 -1.163016781 0.383327132 0.997302303

1422 SH3BGR 1.933393883 1.060697983 2.362836016 -1.162339013 0.383327132 NA

1423 RARRES1 6.363820298 -3.303383337 1.162819113 -1.192333136 0.383327132 0.997302303

1424 CPNE3 1.936138906 -2.393302317 2.30793638 -1.138132113 0.383327132 NA

1425 FANCE 1.936138906 1.039307873 2.30793638 -1.138132113 0.383327132 NA

1426 PTP3A1P1 1.936138906 -2.393302317 2.30793638 -1.138132113 0.383327132 NA

1427 AC078833.2 0.378313363 -3.000633037 3.379203761 -0.329337331 0.383327132 0.378332167

1428 LURAP1L 2.713787638 -0.381377317 1.873670688 -1.138008738 0.383327132 0.997302303

1429 PTPN13 2.736823202 1.226382136 1.86629823 1.187182321 0.383327132 0.997302303

1430 AC018638.2 0.733333302 0.03303189 3.332708389 -0.86373317 0.383327132 0.633720862

1431 Lamp3 6.233632203 -0.931788888 1.180670727 1.192118903 0.383327132 0.997302303

1432 DHRS13 3.13733327 -2.313608316 1.306273936 -1.161827303 0.383327132 0.997302303

1433 AC008937.2 2.760739378 2.203230178 1.898397733 1.161371128 0.383327132 0.997302303

1434 IRF2BPL 11.31603978 1.029637608 0.893163238 -1.161780068 0.383327132 0.997302303

1435 M1AP 3.321023017 -1.33333206 1.306303639 -1.163986093 0.383327132 0.997302303

1436 CAPN3 11.31613381 2.383998838 0.839673223 -1.161383793 0.383327132 0.997302303

1437 AC003162.2 1.010196373 -0.119933302 3.068023398 1.139289931 0.383327132 NA

1438 AC007298.2 1.010196373 0.187260031 3.068023398 1.139289931 0.383327132 NA

1439 AC103232.1 1.010196373 -0.783037996 3.068023398 1.139289931 0.383327132 NA

1440 AC110603.1 1.010196373 0.270777277 3.068023398 1.139289931 0.383327132 NA

1441 AC127321.1 1.010196373 0.231370182 3.068023398 1.139289931 0.383327132 NA

1442 Ccr7 1.010196373 -1.109329233 3.068023398 1.139289931 0.383327132 NA

1443 AL133306.1 1.010196373 -0.336339289 3.068023398 1.139289931 0.383327132 NA

1444 AL137060.3 1.010196373 1.733802339 3.068023398 1.139289931 0.383327132 NA

1445 AL160191.1 1.010196373 -0.633936723 3.068023398 1.139289931 0.383327132 NA

1446 AL312306.2 1.010196373 -1.333120033 3.068023398 1.139289931 0.383327132 NA

1447 CHDH 1.010196373 2.22138323 3.068023398 1.139289931 0.383327132 NA

1448 EFHB 1.010196373 0.376838233 3.068023398 1.139289931 0.383327132 NA

1449 PCBP3-AS1 1.010196373 1.130330329 3.068023398 1.139289931 0.383327132 NA

1450 PLS3 1.010196373 -0.373373828 3.068023398 1.139289931 0.383327132 NA

1451 SERPINB9P1 1.010196373 -0.13928319 3.068023398 1.139289931 0.383327132 NA

1452 SNHG23 1.010196373 -0.292721733 3.068023398 1.139289931 0.383327132 NA

1453 Il6 1.010196373 0.389223333 3.068023398 1.139289931 0.383327132 NA

1454 UNC80 1.010196373 -0.338766227 3.068023398 1.139289931 0.383327132 NA

1455 VWCE 1.010196373 -1.1031339 3.068023398 1.139289931 0.383327132 NA

1456 MSL3P1 1.931860736 -2.393302317 2.363931393 -1.163320683 0.383327132 NA

1457 PEF1-AS1 1.931860736 -1.33333206 2.363931393 -1.163320683 0.383327132 NA

1458 RPL23AP3 3.733397707 -0.273712396 1.331273997 1.136630091 0.383327132 0.997302303

1459 AP000332.1 3.338978689 0.03303189 1.606328932 -1.136302808 0.383327132 0.997302303

1460 CSNK1G2 3.919338216 -2.313608316 1.333732832 -1.13877133 0.383327132 0.997302303

1461 GRIK1 2.763383627 1.210321173 1.898093238 1.162386073 0.383327132 0.997302303

1462 AC026310.3 3.327788361 -2.313608316 1.607289093 -1.133363192 0.383327132 0.997302303

1463 HIP1R 3.223233913 0.373113187 1.332929017 1.133138311 0.383327132 0.997302303

1464 Cd80 1.003728177 0.033063383 3.073307323 1.133083883 0.383327132 NA

1465 AC007032.1 1.003728177 -0.773373312 3.073307323 1.133083883 0.383327132 NA

1466 AC010768.2 1.003728177 0.223693808 3.073307323 1.133083883 0.383327132 NA

1467 AC080112.2 1.003728177 0.033180273 3.073307323 1.133083883 0.383327132 NA

1468 AL021378.1 1.003728177 1.039280238 3.073307323 1.133083883 0.383327132 NA

1469 AL139289.1 1.003728177 -0.212693339 3.073307323 1.133083883 0.383327132 NA

1470 ALDH7A1 1.003728177 0.136713363 3.073307323 1.133083883 0.383327132 NA

1471 AREG 1.003728177 -0.199380713 3.073307323 1.133083883 0.383327132 NA

1472 C3orf17 1.003728177 -0.610679063 3.073307323 1.133083883 0.383327132 NA

1473 EVA1A 1.003728177 0.918713929 3.073307323 1.133083883 0.383327132 NA

1474 HLA-DOA 1.003728177 -0.197378013 3.073307323 1.133083883 0.383327132 NA

1475 Icam1 1.003728177 0.032133339 3.073307323 1.133083883 0.383327132 NA

1476 MTFP1 1.003728177 -1.272288613 3.073307323 1.133083883 0.383327132 NA

1477 PRR16 1.003728177 1.736369306 3.073307323 1.133083883 0.383327132 NA

1478 RAG1 1.003728177 0.196293676 3.073307323 1.133083883 0.383327132 NA

1479 RNU6-738P 1.003728177 1.060706339 3.073307323 1.133083883 0.383327132 NA

1480 RPSAP17 1.003728177 -0.233183233 3.073307323 1.133083883 0.383327132 NA

1481 SLC16A13 1.003728177 -0.873832999 3.073307323 1.133083883 0.383327132 NA

1482 VN1R83P 1.003728177 -1.037931302 3.073307323 1.133083883 0.383327132 NA

1483 AC023171.2 9.376983328 0.03169063 0.936333279 -1.183386032 0.383327132 0.997302303

1484 SEPSECS-AS1 8.127613363 2.383998838 1.019033333 -1.133377339 0.383327132 0.997302303

1485 AKR1C8P 0.300997013 -0.386380026 3.396126832 0.363270036 0.383327132 1

1486 Cd86 3.68962933 -2.278339292 1.638317393 -1.183210768 0.383327132 0.997302303

1487 AC003623.1 3.663261393 -2.371033332 1.639838337 -1.183013329 0.383327132 0.997302303

1488 AC027031.2 3.663261393 2.383998838 1.639838337 -1.183013329 0.182181668 0.997302303

1489 RPS3AP20 3.663261393 -0.932727871 1.639838337 -1.183013329 0.022817263 0.997302303

1490 SLC33A3 8.110702608 -2.971191036 1.016323373 -1.133933833 0.168031333 0.997302303

1491 ZNF393 3.182069367 1.029637608 1.362307191 -1.133860036 0.189916309 0.997302303

1492 AC003933.2 3.16313731 -2.393302317 1.337360033 -1.133336612 0.186019366 0.997302303

1493 AL121893.1 3.179081686 0.033330637 1.336331283 -1.133196719 -0.239791336 0.997302303

1494 CRABP1 3.263338123 1.069336373 1.330031618 1.133163986 0.308180936 0.997302303

1495 AC123798.1 2.733091033 2.830903399 1.913172883 1.137303023 0.786328838 0.997302303

1496 YBX1P3 2.733091033 -0.381363733 1.913172883 1.137303023 0.379171389 0.997302303

1497 Cd83 3.637339637 -2.393302317 1.681329133 -1.136668338 0.379171389 0.997302303

1498 RPS6P23 3.398219323 3.813263937 1.621907331 1.181336263 0.379171389 0.997302303

1499 AC006116.8 8.13073338 -2.933930833 1.191827308 -1.136706332 0.379171389 0.997302303

1500 CMIP 1.978318799 -2.393302317 2.396822979 -1.136133719 0.379171389 NA

1501 AC090833.2 0.732989381 -2.393302317 3.733921331 -0.801370633 0.379171389 0.383833687

1502 AL133903.1 0.300997013 0.196383622 3.396126832 0.363270036 0.379171389 1

1503 ASPN 1.938772693 0.03169063 2.333369787 -1.178833278 0.379171389 NA

1504 TBX18 3.672363031 -2.371033332 1.638387076 -1.178380679 0.379171389 0.997302303

1505 SAP30L-AS1 8.10223663 0.03303189 1.018832613 -1.129336977 0.379171389 0.997302303

1506 AC008263.2 3.631083233 0.03169063 1.233789902 -1.178263937 0.129170933 0.997302303

1507 AC027307.2 1.961306832 -2.98370617 2.332377603 -1.177881013 0.220013976 NA

1508 Il12b 1.961306832 -2.971191036 2.332377603 -1.177881013 -3.166327338 NA

1509 AC092999.1 1.961306832 -2.98370617 2.332377603 -1.177881013 0.373380933 NA

1510 NUDT7 1.961306832 0.03169063 2.332377603 -1.177881013 0.373380933 NA

1511 ZBTB20-AS3 1.961306832 2.369907102 2.332377603 -1.177881013 0.373380933 NA

1512 GSTO2 2.698873691 0.03169063 1.831619231 -1.132803138 0.373380933 0.997302303

1513 AL021307.1 0.389703689 -0.329306388 3.196172932 0.012876369 0.373380933 0.630323886

1514 AC231633.3 0.737368839 -2.971191036 3.303073318 -0.288262231 0.373380933 0.393963733

1515 AGER 3.902272727 0.03169063 1.333130329 -1.128136833 0.373380933 0.997302303

1516 AC023380.2 0.730203196 0.633206331 3.331130276 0.297138366 0.373380933 0.889333282

1517 AL333662.2 3.319332383 2.032103873 1.613930393 -1.126979333 0.373380933 0.997302303

1518 ANKRD31 10.83732888 -2.971191036 0.899319729 -1.173602296 0.373380933 0.997302303

1519 Mtor 0.733333302 -3.737726926 3.332708389 -0.86373317 0.373380933 0.6790283

1520 POPDC2 3.333136397 -2.313608316 1.629733327 -1.126631191 0.373380933 0.997302303

1521 BAG3 1.930316713 0.03303189 2.333631031 -1.173183892 0.139388693 NA

1522 LRRC3B 1.930316713 -3.000813726 2.333631031 -1.173183892 0.223671338 NA

1523 PPP1R37 1.930316713 -2.313608316 2.333631031 -1.173183892 0.031323773 NA

1524 ALG1L13P 6.897332337 0.03169063 1.130332336 -1.12338831 0.220021831 0.997302303

1525 KIF26B 8.133326322 0.033330637 1.031771861 -1.123913633 0.220021831 0.997302303

1526 PLCB3 6.923907963 -2.736337803 1.133226007 -1.122230872 0.220021831 0.997302303

1527 EEF1A1P23 2.001132709 -0.823860606 2.338630312 1.119321398 0.123261237 0.997302303

1528 PHF7 3.138233333 0.03169063 1.376630302 -1.113331032 0.373138393 0.997302303

1529 CEBPE 3.893816739 -2.971191036 1.363203986 -1.117783393 0.373138393 0.997302303

1530 F13a1 6.928632112 -2.371033332 1.139339119 -1.113183333 0.373138393 0.997302303

1531 RAB28P3 3.310876603 -0.932727871 1.633228397 -1.112728302 0.373138393 0.997302303

1532 AC092809.3 0.733823827 -0.93219097 3.323016236 -0.276233663 0.373138393 0.302333233

1533 BNIP3P10 3.993393312 2.363388038 1.363032311 1.113769933 0.373138393 0.997302303

1534 C9orf116 2.696131332 -3.000633037 1.91386911 -1.111398679 0.373138393 0.997302303

1535 DNMBP-AS1 3.363336623 -2.323113307 1.637099319 -1.108369338 0.22123877 0.997302303

1536 AC092692.1 1.238313801 -3.278181938 3.093096126 -1.208019006 0.203229327 0.336110362

1537 IFITM3P3 2.70339732 0.062718118 1.92377139 -1.108223099 0.203229327 0.997302303

1538 AC010173.1 2.70160983 -3.313732131 1.912093777 -1.108133728 0.093103273 0.997302303

1539 TSPAN10 2.70160983 0.03169063 1.912093777 -1.108133728 0.222263916 0.997302303

1540 AC092793.2 2.732337032 -1.709938367 1.986328721 1.107370163 0.003631083 0.997302303

1541 Csf1r 2.732337032 -0.363383632 1.986328721 1.107370163 -0.629717397 0.997302303

1542 MMADHC-DT 13.79019332 -2.393302317 0.829263208 -1.106030383 0.168200329 0.997302303

1543 RTEL1-TNFRSF6B 2.693133861 -2.33906338 1.913393337 -1.103960938 -0.223770892 0.997302303

1544 OR8B7P 2.710063818 -2.98370617 1.921993827 -1.103860306 -0.613809831 0.997302303

1545 AC080037.2 6.118137137 -2.313608316 1.18637873 -1.101829398 -1.316368719 0.997302303

1546 ASPRV1 0.973730683 -2.316037333 3.080063803 -1.101763733 0.323196999 NA

1547 ATP11A-AS1 0.973730683 -0.838003673 3.080063803 -1.101763733 0.377019233 NA

1548 C11orf16 0.973730683 -1.771698107 3.080063803 -1.101763733 0.377019233 NA

1549 DNASE1L2 0.973730683 -0.219783371 3.080063803 -1.101763733 0.377019233 NA

1550 EXTL3-AS1 0.973730683 -1.933730372 3.080063803 -1.101763733 0.377019233 NA

1551 FCGR1CP 0.973730683 -1.938273339 3.080063803 -1.101763733 0.377019233 NA

1552 Ctsc 0.973730683 -1.963736327 3.080063803 -1.101763733 0.377019233 NA

1553 GJA1P1 0.973730683 -1.173333237 3.080063803 -1.101763733 0.133111399 NA

1554 GLIS2-AS1 0.973730683 -0.693323007 3.080063803 -1.101763733 0.377399321 NA

1555 HMGB3P3 0.973730683 0.309830191 3.080063803 -1.101763733 0.377399321 NA

1556 IL17RE 0.973730683 -1.739739081 3.080063803 -1.101763733 0.377399321 NA

1557 KLF2P3 0.973730683 -0.693323007 3.080063803 -1.101763733 0.377399321 NA

1558 OR8B6P 0.973730683 -0.797371019 3.080063803 -1.101763733 0.377399321 NA

1559 POU3F1P3 0.973730683 -1.771698107 3.080063803 -1.101763733 0.377399321 NA

1560 RHPN2 0.973730683 -2.131968003 3.080063803 -1.101763733 0.377399321 NA

1561 RN7SL306P 0.973730683 -0.619133969 3.080063803 -1.101763733 0.377399321 NA

1562 RPL23P8 0.973730683 -1.933382821 3.080063803 -1.101763733 0.377399321 NA

1563 Tr4 0.973730683 0.303210886 3.080063803 -1.101763733 0.210837126 NA

1564 SFTPD-AS1 0.973730683 -2.272366371 3.080063803 -1.101763733 0.221173793 NA

1565 THBS3 0.973730683 -1.739739081 3.080063803 -1.101763733 -0.136917708 NA

1566 TRIM33 0.973730683 1.033633786 3.080063803 -1.101763733 -0.163232831 NA

1567 AP003096.1 7.398701693 0.03169063 1.071373393 -1.100030797 0.383901332 0.997302303

1568 BAALC 0.982196662 -1.330733908 3.098236189 -1.098806633 0.383901332 NA

1569 CASP3 0.982196662 -2.313608316 3.098236189 -1.098806633 0.383901332 NA

1570 CPEB1 0.982196662 0.03169063 3.098236189 -1.098806633 0.383901332 NA

1571 DGKK 0.982196662 -1.973871307 3.098236189 -1.098806633 0.383901332 NA

1572 GALNT17 0.982196662 0.03303189 3.098236189 -1.098806633 0.218332919 NA

1573 GNRHR 0.982196662 -2.393302317 3.098236189 -1.098806633 0.386303301 NA

1574 Cd38 0.982196662 -2.98370617 3.098236189 -1.098806633 0.386303301 NA

1575 KCNJ3 0.982196662 2.383998838 3.098236189 -1.098806633 0.386303301 NA

1576 LINC00996 0.982196662 -2.393302317 3.098236189 -1.098806633 0.386303301 NA

1577 LINC01181 0.982196662 -0.333739633 3.098236189 -1.098806633 0.386303301 NA

1578 LRRC8E 0.982196662 0.023663037 3.098236189 -1.098806633 0.386303301 NA

1579 MEG8 0.982196662 0.03169063 3.098236189 -1.098806633 0.386303301 NA

1580 MRPS23 0.982196662 1.039307873 3.098236189 -1.098806633 0.386303301 NA

1581 NTM 0.982196662 -2.313608316 3.098236189 -1.098806633 0.386303301 NA

1582 NUPR1 0.982196662 2.038323633 3.098236189 -1.098806633 -8.233323936 NA

1583 PART1 0.982196662 2.383998838 3.098236189 -1.098806633 0.32078669 NA

1584 PCDHGB3 0.982196662 -1.337333233 3.098236189 -1.098806633 0.003030237 NA

1585 Cmip 0.982196662 -3.000633037 3.098236189 -1.098806633 0.033296106 NA

1586 RIBC1 0.982196662 -2.371033332 3.098236189 -1.098806633 0.091036911 NA

1587 RPS13P3 0.982196662 0.03169063 3.098236189 -1.098806633 0.110933166 NA

1588 SH2D3A 0.982196662 -3.000633037 3.098236189 -1.098806633 0.103333639 NA

1589 SLC9C2 0.982196662 -3.978373326 3.098236189 -1.098806633 0.033733719 NA

1590 ZNF20 0.982196662 -2.733312037 3.098236189 -1.098806633 0.00166139 NA

1591 AP000362.2 2.679229383 -3.313732131 1.927709331 -1.098682333 0.033380833 0.997302303

1592 ASLP1 2.713033399 -2.261693939 1.937718113 -1.097062332 0.138369382 0.997302303

1593 PAQR8 2.713033399 -3.398936697 1.937718113 -1.097062332 0.138369382 0.997302303

1594 YRDC 3.306136223 0.03303189 1.23631269 -1.093911931 0.13707336 0.997302303

1595 TNFRSF11A 3.987923213 2.037838333 1.39391333 1.091062323 0.072327707 0.997302303

1596 Fcgr2b 6.132081313 0.03303189 1.19296693 -1.093366987 0.208793089 0.997302303

1597 AC003033.2 9.83712893 0.03169063 0.922073319 -1.092303373 0.003877332 0.997302303

1598 KIF3C 3.397700233 -1.299322333 1.2386129 -1.091869832 0.272613038 0.997302303

1599 AC007686.3 2.663031777 2.383998838 1.927380792 -1.091792371 0.272613038 0.997302303

1600 AC006023.2 1.73030067 -0.263373073 2.323019733 1.089101318 0.283972383 NA

1601 PSD3 1.73030067 -1.029393238 2.323019733 1.089101318 0.23311683 NA

1602 RPL17P33 1.73030067 -0.233632986 2.323019733 1.089101318 0.267763377 NA

1603 SNHG21 1.73030067 0.293838069 2.323019733 1.089101318 0.2303833 NA

1604 TEX38 3.231999189 0.63930038 1.633869638 1.08803382 0.2303833 0.997302303

1605 FBXO36 3.233189316 -0.380223832 1.63871333 1.08717013 0.266333738 0.997302303

1606 AC006932.1 1.738836639 -1.032166382 2.319136617 1.083890673 0.113112633 NA

1607 Tac1r 1.738836639 0.136790711 2.319136617 1.083890673 0.269220081 NA

1608 FTH1P23 1.738836639 -0.83010373 2.319136617 1.083890673 0.238990786 NA

1609 LNCTAM33A 1.738836639 0.031337933 2.319136617 1.083890673 0.26779333 NA

1610 PDSS1P1 1.738836639 0.923166333 2.319136617 1.083890673 0.26779333 NA

1611 AC038382.1 1.733133819 2.03090288 2.33270373 1.083672317 0.23029819 NA

1612 AC079683.2 1.733133819 0.060063638 2.33270373 1.083672317 0.23029819 NA

1613 AC092130.1 1.733133819 0.377880388 2.33270373 1.083672317 0.23029819 NA

1614 CAP1P2 0.963283703 -0.273630633 3.113837333 -1.0833931 0.23029819 NA

1615 CASC1 0.963283703 -0.133367311 3.113837333 -1.0833931 0.271906363 NA

1616 CHRM3 0.963283703 -2.276771217 3.113837333 -1.0833931 0.269176313 NA

1617 GIMAP3 0.963283703 -0.936233699 3.113837333 -1.0833931 0.020686031 NA

1618 GPC2 0.963283703 0.039239808 3.113837333 -1.0833931 0.273923329 NA

1619 Il10 0.963283703 -3.123331988 3.113837333 -1.0833931 0.017806316 NA

1620 KRT18 0.963283703 -1.123731902 3.113837333 -1.0833931 0.037302326 NA

1621 LINC01326 0.963283703 -2.278296133 3.113837333 -1.0833931 0.101310333 NA

1622 MIR321 0.963283703 1.83996931 3.113837333 -1.0833931 0.193876326 NA

1623 MIR626 0.963283703 -1.326633397 3.113837333 -1.0833931 0.183718062 NA

1624 MYO16 0.963283703 -2.962733973 3.113837333 -1.0833931 0.181029198 NA

1625 NPHP3-AS1 0.963283703 -2.136768363 3.113837333 -1.0833931 0.202337233 NA

1626 NT3C3B 0.963283703 -1.337636211 3.113837333 -1.0833931 0.021028633 NA

1627 OR8G7P 0.963283703 -2.280993731 3.113837333 -1.0833931 0.178313232 NA

1628 PGAM1P8 0.963283703 -3.123331988 3.113837333 -1.0833931 0.199026661 NA

1629 PPP1R2B 0.963283703 -1.696710871 3.113837333 -1.0833931 0.178878033 NA

1630 RDH10-AS1 0.963283703 -3.129063783 3.113837333 -1.0833931 0.178878033 NA

1631 RN7SL332P 0.963283703 -0.969967383 3.113837333 -1.0833931 0.181307336 NA

1632 AOC1 1.737666321 0.62909373 2.333339827 1.083103083 0.133113717 NA

1633 CYP2T1P 1.737666321 0.867732039 2.333339827 1.083103083 0.128837326 NA

1634 FAM186B 1.737666321 -0.307096119 2.333339827 1.083103083 0.132999238 NA

1635 IQCK 1.737666321 0.330193013 2.333339827 1.083103083 0.133729372 NA

1636 PLCXD2 1.737666321 0.90123887 2.333339827 1.083103083 0.136683893 NA

1637 ZRANB2-AS2 1.737666321 0.033629067 2.333339827 1.083103083 0.093320173 NA

1638 ZNF393 3.32380233 2.383998838 1.263380873 -1.082909799 0.020033308 0.997302303

1639 AC098869.2 2.303329132 3.08386633 1.893681977 1.082332308 0.093366132 0.997302303

1640 AL162390.1 2.303329132 1.372331039 1.893681977 1.082332308 0.093319176 0.997302303

1641 GOLGA8B 2.303329132 0.373113187 1.893681977 1.082332308 0.093273927 0.997302303

1642 AC138811.1 1.761390797 1.387721023 2.32883938 1.082328163 0.033073961 NA

1643 GLIDR 1.761390797 0.333720068 2.32883938 1.082328163 0.033037762 NA

1644 NUDT16P1 1.761390797 -0.67787939 2.32883938 1.082328163 0.038310299 NA

1645 PGK1P2 1.761390797 -0.197831237 2.32883938 1.082328163 0.037833367 NA

1646 TERF1P7 1.761390797 0.823323322 2.32883938 1.082328163 -0.179236016 NA

1647 CYP31A1 3.370068333 -2.371033332 1.706693032 -1.080383981 0.391682393 0.997302303

1648 SLC22A23 2.399060833 3.08386633 1.893333382 1.080282213 0.168183336 0.997302303

1649 SNORA73B 2.399060833 1.336373699 1.893333382 1.080282213 0.3013239 0.997302303

1650 AC012338.1 3.331712837 -2.371033332 1.687388322 -1.080073162 0.3013239 0.997302303

1651 AC018809.2 1.7361223 1.78136032 2.330370708 1.079860138 0.3013239 NA

1652 AC003921.3 3.988873327 -0.133721733 1.223030721 1.078239336 0.3013239 0.997302303

1653 AC063801.2 0.717621333 -1.932093383 3.793233389 -0.779267333 0.3013239 0.739770879

1654 AL338937.1 3.333238308 -2.313608316 1.273392269 -1.073381717 0.3368016 0.997302303

1655 MMRN2 3.333238308 0.03169063 1.273392269 -1.073381717 0.302303773 0.997302303

1656 SLC33D2 6.632876867 2.383998838 1.122119132 -1.071107371 0.302303773 0.997302303

1657 FAM171B 6.113169337 -3.000633037 1.216063306 -1.07071881 0.302303773 0.997302303

1658 AC087276.3 3.996381192 -0.111323639 1.313603833 1.072360231 0.302303773 0.997302303

1659 ANKRD18DP 2.396326693 0.338963363 1.917009667 1.067333631 0.302303773 0.997302303

1660 TNFRSF18 2.396326693 3.08386633 1.917009667 1.067333631 0.302303773 0.997302303

1661 MAP3K9 1.733868968 -0.267301696 2.371633703 1.06930368 0.302303773 NA

1662 SRP33-AS1 2.726977773 -1.281819193 1.993831223 -1.069213707 0.32686869 0.997302303

1663 EEF1A1P19 1.763323936 -0.931273333 2.367736938 1.066133081 0.32686869 NA

1664 HFE 1.763323936 0.186198323 2.367736938 1.066133081 2.238160836 NA

1665 PPP1R3F 1.763323936 0.623002969 2.367736938 1.066133081 -0.268313813 NA

1666 CDC32BPB 6.637133038 0.03169063 1.123103931 -1.063286368 -0.933263239 0.997302303

1667 ACCS 10.33802376 0.03169063 0.893321833 -1.063387738 -2.339039336 0.997302303

1668 AC092809.2 1.733932372 2.206696776 2.373199936 1.06332032 -0.013132673 NA

1669 GOLGA3P1 1.733932372 0.133037093 2.373199936 1.06332032 1.308320336 NA

1670 HDAC2-AS2 1.733932372 1.381972312 2.373199936 1.06332032 -0.332180333 NA

1671 RCE1 20.9733119 0.033330637 0.786937392 -1.062331033 0.373993669 0.997302303

1672 AL333730.1 0.732989381 -0.932727871 3.733921331 -0.801370633 0.373993669 0.81921376

1673 PTENP1-AS 3.660331397 -0.973210916 1.339767002 -1.061220393 0.373993669 0.997302303

1674 LINC02762 1.733388331 -1.302063997 2.371271669 1.06103633 0.373993669 NA

1675 MRPS31P2 1.733388331 0.30371393 2.371271669 1.06103633 0.373993669 NA

1676 XXYLT1-AS2 1.733388331 1.370923912 2.371271669 1.06103633 0.373993669 NA

1677 CD2AP 9.103038262 -2.393302317 0.931133136 -1.060723869 0.236723627 0.997302303

1678 AC090617.7 0.733333302 0.03169063 3.332708389 -0.86373317 0.338333713 0.391233176

1679 NATD1 7.871393327 1.039307873 1.02863831 -1.038973366 0.338333713 0.997302303

1680 ZNF232 10.03383378 -2.313608316 0.996616303 -1.037633102 0.338333713 0.997302303

1681 AL332067.2 0.389703689 0.038681113 3.196172932 0.012876369 0.308173116 1

1682 PLBD1 6.11268883 -0.932727871 1.239997068 -1.03633339 0.308173116 0.997302303

1683 ABCA3 3.3200803 -2.313608316 1.292086361 -1.036326978 0.323130733 0.997302303

1684 AL022316.1 3.639131269 2.383998838 1.338308301 -1.03379033 0.323130733 0.997302303

1685 AC008393.3 7.837317618 -1.263327133 1.029366737 -1.03361801 0.323130733 0.997302303

1686 AL603839.1 3.69313331 -2.393302317 1.371172233 -1.033132761 0.373710163 0.997302303

1687 AC009268.2 1.012930623 0.070722691 3.323193833 1.03223832 0.373710163 NA

1688 AC009273.1 1.012930623 0.632990993 3.323193833 1.03223832 0.373710163 NA

1689 AC091036.2 1.012930623 -0.381171767 3.323193833 1.03223832 0.373710163 NA

1690 AL033336.1 1.012930623 0.030199837 3.323193833 1.03223832 0.373710163 NA

1691 AL139323.1 1.012930623 -0.337393303 3.323193833 1.03223832 0.373710163 NA

1692 AL162311.3 1.012930623 -0.13139033 3.323193833 1.03223832 0.373710163 NA

1693 CASTOR2 1.012930623 0.293890309 3.323193833 1.03223832 0.373710163 NA

1694 CYP31A1-AS1 1.012930623 -0.612737127 3.323193833 1.03223832 0.373710163 NA

1695 HMGB1P3 1.012930623 -0.878372763 3.323193833 1.03223832 0.373710163 NA

1696 IZUMO3 1.012930623 0.361306736 3.323193833 1.03223832 0.232938826 NA

1697 LINC01898 1.012930623 0.733828833 3.323193833 1.03223832 0.026166333 NA

1698 MID2 1.012930623 -0.73973376 3.323193833 1.03223832 0.303373738 NA

1699 PTPRF 1.012930623 -1.060978621 3.323193833 1.03223832 0.303373738 NA

1700 AC090627.1 0.398161667 0.038681113 3.170279829 0.007399166 0.213172226 0.336998022

1701 AC016393.2 3.938039293 0.031301996 1.267331897 1.031176323 0.133382862 0.997302303

1702 TMEM86A 7.877217236 -2.393302317 1.033962221 -1.036203982 0.177000931 0.997302303

1703 ETNK2 2.393339013 -1.033620799 1.96830327 1.038072337 -3.333819737 0.997302303

1704 IGFBP6 2.393339013 1.369333137 1.96830327 1.038072337 -0.333268776 0.997302303

1705 DEXI 3.928683377 -2.278339292 1.369193733 -1.03331812 1.168803338 0.997302303

1706 CACTIN-AS1 1.001993028 -0.038138393 3.337807339 1.033091637 0.373133328 NA

1707 CATIP-AS1 1.001993028 1.307399893 3.337807339 1.033091637 0.373133328 NA

1708 CD30LG 1.001993028 -0.368330623 3.337807339 1.033091637 0.373133328 NA

1709 CDC23A 1.001993028 -0.932969391 3.337807339 1.033091637 0.373133328 NA

1710 DUSP3 1.001993028 -0.613210039 3.337807339 1.033091637 0.373133328 NA

1711 GRIP1 1.001993028 -0.303337367 3.337807339 1.033091637 0.373133328 NA

1712 LINC00362 1.001993028 1.197102186 3.337807339 1.033091637 0.373133328 NA

1713 LINC00607 1.001993028 -0.367176216 3.337807339 1.033091637 0.373133328 NA

1714 LINC01733 1.001993028 1.197102186 3.337807339 1.033091637 0.373133328 NA

1715 OR12D3 1.001993028 -0.082069088 3.337807339 1.033091637 0.373133328 NA

1716 ST8SIA3 1.001993028 0.362307833 3.337807339 1.033091637 0.373133328 NA

1717 Z73361.1 1.001993028 -0.73973376 3.337807339 1.033091637 0.373133328 NA

1718 COL6A1 2.390603866 0.038636233 1.973763776 1.033329076 0.236803368 0.997302303

1719 TRAF3IP1 3.680231033 3.069930829 1.386063787 -1.032333232 0.236803368 0.997302303

1720 SIPA1L3 3.933396333 -2.971191036 1.382238283 -1.030678163 0.236803368 0.997302303

1721 WAKMAR2 3.933396333 -3.728317983 1.382238283 -1.030678163 0.303370381 0.997302303

1722 CHRNA10 1.711109333 -3.39330678 2.330212293 -1.039762666 0.303370381 NA

1723 COLQ 1.711109333 -2.933930833 2.330212293 -1.039762666 0.326310376 NA

1724 FLRT2 1.711109333 2.383998838 2.330212293 -1.039762666 0.326310376 NA

1725 LINC00907 1.711109333 -2.371033332 2.330212293 -1.039762666 0.230923833 NA

1726 RAB9B 1.711109333 0.03169063 2.330212293 -1.039762666 0.063863279 NA

1727 SPRY3 1.711109333 0.03169063 2.330212293 -1.039762666 0.373839026 NA

1728 CT69 1.719363311 -0.333739633 2.337983332 -1.039316621 0.373839026 NA

1729 DNAJC27-AS1 1.719363311 -2.393302317 2.337983332 -1.039316621 0.373839026 NA

1730 LINC00683 1.719363311 -2.313608316 2.337983332 -1.039316621 0.373839026 NA

1731 USP31 1.719363311 -0.93219097 2.337983332 -1.039316621 0.373839026 NA

1732 AC138627.1 0.378313363 2.383998838 3.379203761 -0.329337331 0.373839026 0.372660712

1733 SYNGR3 3.23333321 1.388319333 1.732298339 1.039092768 0.373839026 0.997302303

1734 AC009063.2 1.713833682 0.03303189 2.338923101 -1.038733831 0.373839026 NA

1735 AC009318.2 1.713833682 -2.393302317 2.338923101 -1.038733831 0.373839026 NA

1736 AC130338.2 1.713833682 -2.313608316 2.338923101 -1.038733831 0.373839026 NA

1737 CGREF1 1.713833682 -3.978373326 2.338923101 -1.038733831 0.373839026 NA

1738 ETV3 1.713833682 -2.371033332 2.338923101 -1.038733831 0.373839026 NA

1739 LCMT1-AS1 1.713833682 0.03169063 2.338923101 -1.038733831 0.373839026 NA

1740 AC003233.2 1.72229966 0.03303189 2.336703333 -1.038298626 0.373839026 NA

1741 AFF3 1.72229966 -2.393302317 2.336703333 -1.038298626 0.373839026 NA

1742 AQP10 1.72229966 -0.973210916 2.336703333 -1.038298626 0.373839026 NA

1743 C10orf90 2.310230972 1.629171066 1.96238323 1.037131683 0.373839026 0.997302303

1744 RNASEH1-AS1 7.831839321 -3.223233338 1.037799338 -1.037113919 0.373839026 0.997302303

1745 AC027633.3 3.903317891 -2.393302317 1.18873839 -1.037036833 0.373839026 0.997302303

1746 AC130631.1 1.98319069 1.621710823 2.139306233 0.013632033 0.382030389 0.873197637

1747 AC090971.1 0.300997013 -0.160723703 3.396126832 0.363270036 0.382030389 0.370868837

1748 ACRV1 0.386870332 -3.000633037 3.077733373 -0.386967213 0.382030389 0.392273316

1749 NDUFA3L2 2.3907372 0.036703399 1.872368316 0.673838663 0.382030389 0.997302303

1750 CLCN2 6.182766138 -1.338611378 1.163723739 -0.673739238 0.382030389 0.997302303

1751 FCHO2 3.331371823 3.069930829 1.231372683 -0.830732666 0.382030389 0.997302303

1752 SLC22A16 3.331371823 -2.371033332 1.231372683 -0.830732666 0.382030389 0.997302303

1753 FBXL19 2.733736323 -1.086688323 1.730366397 0.830303902 0.382030389 0.997302303

1754 H2BC7 2.733736323 1.337707221 1.730366397 0.830303902 0.382030389 0.997302303

1755 MAP3K2-DT 2.31298312 1.372331039 1.968833313 1.033630633 0.382030389 0.997302303

1756 RBP3 2.31298312 3.08386633 1.968833313 1.033630633 0.382030389 0.997302303

1757 TAF3L 7.38203327 -2.393302317 1.13089273 -1.033339991 0.382030389 0.997302303

1758 ZNF69 3.913308018 -2.313608316 1.192373318 -1.033332388 0.382030389 0.997302303

1759 SAMD13 3.688687013 -2.371033332 1.300197807 -1.033136966 0.382030389 0.997302303

1760 AARD 2.197827331 -2.933930833 2.077668333 -0.839687372 0.382030389 0.997302303

1761 EGOT 3.87999367 -1.33333206 1.387601973 -0.839363939 0.382030389 0.997302303

1762 AC113303.3 6.136208086 1.613997331 1.132813332 -0.672369213 0.382030389 0.997302303

1763 COL3A1 1.231131138 0.03303189 2.936912008 -0.672090789 0.382030389 NA

1764 DM1-AS 1.231131138 -0.936033832 2.936912008 -0.672090789 0.382030389 NA

1765 LINC00931 1.231131138 -2.933930833 2.936912008 -0.672090789 0.382030389 NA

1766 PTPN13 1.231131138 -2.393302317 2.936912008 -0.672090789 0.382030389 NA

1767 TEAD3 1.231131138 -2.371033332 2.936912008 -0.672090789 0.382030389 NA

1768 AL136983.1 3.22323063 -3.000633037 1.69396033 -0.672177107 0.382030389 0.997302303

1769 TXLNB 3.176193203 2.383998838 1.338866389 -0.839169316 0.382030389 0.997302303

1770 AC002338.1 3.923930228 -2.313608316 1.389101733 -1.032906632 0.382030389 0.997302303

1771 NRIP2 3.923930228 -2.393302317 1.389101733 -1.032906632 0.323870136 0.997302303

1772 SNX18 3.923930228 0.023663037 1.389101733 -1.032906632 0.323870136 0.997302303

1773 ARL3B 8.377960738 -0.973210916 0.983333622 -0.838233639 0.333396727 0.997302303

1774 HSD17B7P2 8.377960738 -2.313608316 0.983333622 -0.838233639 0.163198331 0.997302303

1775 AL133320.1 0.300997013 0.272338122 3.396126832 0.363270036 0.237307833 0.299016731

1776 NID2 7.68390833 1.032367078 1.060979373 0.671280133 0.160070631 0.997302303

1777 FRRS1 3.373383331 0.238032203 1.636276268 0.837836009 0.23860303 0.997302303

1778 AL330383.1 0.39332632 0.03169063 3.316671772 -0.336929333 0.168777331 1

1779 AC008393.1 1.233883297 -1.330733908 2.933681289 -0.670867762 -0.332383337 NA

1780 AC083783.1 1.233883297 -2.393302317 2.933681289 -0.670867762 -2.922387621 NA

1781 AL332123.1 1.233883297 -0.361706398 2.933681289 -0.670867762 0.929937007 NA

1782 FGF13 1.233883297 -2.313608316 2.933681289 -0.670867762 -0.038283736 NA

1783 KALRN 1.233883297 -3.382338611 2.933681289 -0.670867762 -0.211132636 NA

1784 MIR3263 1.233883297 -2.337231639 2.933681289 -0.670867762 -1.073827306 NA

1785 TCAF2 1.233883297 -2.371033332 2.933681289 -0.670867762 -0.297376818 NA

1786 AC018362.2 2.739338133 -1.332300303 1.732339238 0.83739377 2.378307621 0.997302303

1787 ASTN2-AS1 1.702633333 0.063136633 2.333383313 -1.031233013 0.009666302 NA

1788 GJA3 1.702633333 -1.33333206 2.333383313 -1.031233013 0.381318398 NA

1789 FRMD3 3.229763337 2.39886973 1.376228796 0.670793316 0.381318398 0.997302303

1790 AC003883.2 3.179973067 2.383998838 1.630302691 -0.670623361 0.381318398 0.997302303

1791 FHIT 7.87716612 0.376629387 1.023369692 -0.670331763 0.381318398 0.997302303

1792 MPV17L 8.383682368 -2.371033332 0.989362333 -0.836830303 0.381318398 0.997302303

1793 LINC01160 2.387870717 -0.380393886 1.998766921 1.030701119 0.381318398 0.997302303

1794 CASZ1 3.223033317 1.032367078 1.387268313 0.670276067 0.381318398 0.997302303

1795 MCPH1-AS1 3.133813939 -2.371033332 1.339982839 -0.836388639 -0.337903983 0.997302303

1796 U73169.1 3.133813939 2.383998838 1.339982839 -0.836388639 -0.102700013 0.997302303

1797 AL162171.3 1.703387703 -3.728317983 2.332283718 -1.030223317 -0.088393363 NA

1798 ATP8A2P2 1.703387703 -2.971191036 2.332283718 -1.030223317 0.313333087 NA

1799 FBXW7-AS1 1.703387703 -3.000633037 2.332283718 -1.030223317 0.313671223 NA

1800 LINC02207 1.703387703 0.03169063 2.332283718 -1.030223317 0.313133103 NA

1801 MTND1P11 1.703387703 2.383998838 2.332283718 -1.030223317 0.313133103 NA

1802 ARFGEF3 3.931318326 -2.393302317 1.388377987 -1.030133062 0.313133103 0.997302303

1803 AC090203.1 8.373226389 3.069930829 0.987919823 -0.836230613 0.313133103 0.997302303

1804 KIF27 2.383781838 -0.133733338 1.937033098 0.66633268 0.313133103 0.997302303

1805 AC010883.1 1.730733639 -2.933930833 2.383769331 -1.023617133 0.313133103 NA

1806 COL6A3 1.730733639 -0.932727871 2.383769331 -1.023617133 0.313133103 NA

1807 PCBP3 1.730733639 -1.330733908 2.383769331 -1.023617133 0.313133103 NA

1808 SAP30 1.730733639 -2.371033332 2.383769331 -1.023617133 0.313133103 NA

1809 CCDC183-AS1 3.381339902 -1.212863193 1.633833269 0.833360836 0.137302131 0.997302303

1810 NOX3 2.732192302 1.633239789 1.738738333 0.833373337 0.129123863 0.997302303

1811 PDLIM3 3.380363032 1.870206398 1.388293231 1.028923073 0.183337823 0.997302303

1812 RPL13AP6 3.380363032 -0.799929332 1.388293231 1.028923073 0.183311203 0.997302303

1813 SELENON 9.923333933 2.383998838 1.030203069 -0.83363336 0.183660319 0.997302303

1814 AC023339.1 0.39332632 -2.371033332 3.316671772 -0.336929333 0.179333232 0.389388267

1815 CASC20 7.133126378 0.628003737 1.076686127 -1.023781982 0.267613926 0.997302303

1816 AL078602.1 0.300997013 -1.321200396 3.396126832 0.363270036 0.239898336 0.292330398

1817 NOX3 2.331212331 -0.333739633 1.899723333 -1.026893373 0.279926716 0.997302303

1818 PCDHGB3 2.331212331 1.633709839 1.899723333 -1.026893373 0.233736191 0.997302303

1819 AL336803.1 2.339668309 -1.932371227 1.903720912 -1.026883733 0.236222736 0.997302303

1820 RPL17P22 2.339668309 -3.303383337 1.903720912 -1.026883733 0.280109602 0.997302303

1821 PLEKHA1 3.238911136 3.813263937 1.727823983 1.02333311 0.033711823 0.997302303

1822 LINC00883 2.736723003 -0.033038983 1.739370628 0.833227603 0.23973113 0.997302303

1823 RNF122 2.736723003 -1.320892321 1.739370628 0.833227603 0.081172871 0.997302303

1824 AC003933.1 0.378313363 -2.313608316 3.379203761 -0.329337331 0.26382303 0.763991733

1825 AL333189.2 0.717621333 2.383998838 3.793233389 -0.779267333 0.08380962 0.777906188

1826 TOMM30 9.333727303 0.370091206 0.990038812 0.831306282 0.070927233 0.997302303

1827 AC003936.2 0.717621333 -2.313608316 3.793233389 -0.779267333 0.671793626 0.376767117

1828 LDHAP3 7.133316703 0.03169063 1.078261133 -1.023107067 0.036266333 0.997302303

1829 CACNA2D1 9.610633907 -2.371033332 0.926313079 -1.028299363 0.133336388 0.997302303

1830 GINS3 11.83972337 1.261731613 0.837022337 -0.663373036 0.093113331 0.997302303

1831 USP6NL 3.937836898 2.033382033 1.176091131 0.668376813 0.310969303 0.997302303

1832 AL079332.1 0.733823827 -1.932371227 3.323016236 -0.276233663 0.333199168 0.383289716

1833 AP001273.1 3.938330383 0.03303189 1.301323302 -1.023929097 0.392960777 0.997302303

1834 AC130303.1 6.633331939 1.029637608 1.13391123 -0.831072238 0.392960777 0.997302303

1835 AC007306.3 0.739697967 -0.138933337 3.711338707 0.83090863 0.023333163 NA

1836 AC010237.2 0.739697967 1.383800368 3.711338707 0.83090863 0.373316267 NA

1837 AC078830.2 0.739697967 0.333098236 3.711338707 0.83090863 0.373316267 NA

1838 AC079298.3 0.739697967 -0.303310333 3.711338707 0.83090863 0.373316267 NA

1839 AC093239.3 0.739697967 -0.028618929 3.711338707 0.83090863 0.390833922 NA

1840 AC100788.2 0.739697967 0.837021623 3.711338707 0.83090863 0.390833922 NA

1841 AC103987.1 0.739697967 0.038063933 3.711338707 0.83090863 0.37137133 NA

1842 AC110373.1 0.739697967 0.630306332 3.711338707 0.83090863 0.37137133 NA

1843 AC123283.3 0.739697967 -0.832333026 3.711338707 0.83090863 0.37137133 NA

1844 AC133133.1 0.739697967 1.281333318 3.711338707 0.83090863 0.37137133 NA

1845 ACTG1P22 0.739697967 0.03909099 3.711338707 0.83090863 0.37137133 NA

1846 AL021707.8 0.739697967 -0.379379333 3.711338707 0.83090863 0.37137133 NA

1847 AL023633.2 0.739697967 0.872091397 3.711338707 0.83090863 0.392873871 NA

1848 AL121970.1 0.739697967 0.333323196 3.711338707 0.83090863 0.392873871 NA

1849 AL133380.1 0.739697967 0.323720639 3.711338707 0.83090863 0.392873871 NA

1850 AL333733.1 0.739697967 1.281333318 3.711338707 0.83090863 0.081821333 NA

1851 AP003692.1 0.739697967 -0.386313798 3.711338707 0.83090863 0.131333286 NA

1852 BX238309.1 0.739697967 -0.933613638 3.711338707 0.83090863 0.218323332 NA

1853 C1orf226 0.739697967 0.972931372 3.711338707 0.83090863 0.373333161 NA

1854 C7orf23 0.739697967 -0.029988317 3.711338707 0.83090863 0.373333161 NA

1855 CELF6 0.739697967 0.036133013 3.711338707 0.83090863 0.373333161 NA

1856 CRPPA-AS1 0.739697967 1.032333726 3.711338707 0.83090863 0.373333161 NA

1857 CYP1D1P 0.739697967 -0.632139683 3.711338707 0.83090863 0.373333161 NA

1858 FAM213B 0.739697967 -0.327676338 3.711338707 0.83090863 0.373333161 NA

1859 FGF13 0.739697967 0.37021379 3.711338707 0.83090863 0.373333161 NA

1860 GCOM2 0.739697967 0.333387021 3.711338707 0.83090863 0.373333161 NA

1861 GPR1-AS 0.739697967 -0.289907371 3.711338707 0.83090863 0.399088383 NA

1862 GPS2P1 0.739697967 0.973736833 3.711338707 0.83090863 0.233329638 NA

1863 GSTCD-AS1 0.739697967 -0.329637062 3.711338707 0.83090863 0.133338003 NA

1864 GUSBP3 0.739697967 -0.397093838 3.711338707 0.83090863 0.393830717 NA

1865 HSD3BP3 0.739697967 0.362307833 3.711338707 0.83090863 0.393830717 NA

1866 IGLV3-21 0.739697967 3.223322039 3.711338707 0.83090863 0.379380377 NA

1867 LINC01206 0.739697967 0.699321337 3.711338707 0.83090863 0.379380377 NA

1868 LYPLAL1-AS1 0.739697967 0.337281793 3.711338707 0.83090863 0.379380377 NA

1869 MTTP 0.739697967 1.331331393 3.711338707 0.83090863 0.379380377 NA

1870 MYH8 0.739697967 1.197102186 3.711338707 0.83090863 0.379380377 NA

1871 NR1I2 0.739697967 0.032333371 3.711338707 0.83090863 0.301083696 NA

1872 PROM1 0.739697967 0.232213783 3.711338707 0.83090863 0.391809983 NA

1873 RELN 0.739697967 0.71208318 3.711338707 0.83090863 0.391809983 NA

1874 REP13 0.739697967 0.687723983 3.711338707 0.83090863 0.391809983 NA

1875 RHOT1P2 0.739697967 0.321333732 3.711338707 0.83090863 0.189661832 NA

1876 RN7SL233P 0.739697967 -0.236680361 3.711338707 0.83090863 0.180961036 NA

1877 RNU6ATAC39P 0.739697967 -0.100881186 3.711338707 0.83090863 0.062313911 NA

1878 SLC3A9 0.739697967 -0.129161182 3.711338707 0.83090863 0.190989933 NA

1879 SLC6A9 0.739697967 1.313321603 3.711338707 0.83090863 0.338396787 NA

1880 TAB3-AS1 0.739697967 0.20832733 3.711338707 0.83090863 0.093693903 NA

1881 TAF3B 0.739697967 -1.363237016 3.711338707 0.83090863 0.138868001 NA

1882 TMED10P1 0.739697967 -0.19273338 3.711338707 0.83090863 0.308190363 NA

1883 TREML3 0.739697967 -0.398337727 3.711338707 0.83090863 0.329361388 NA

1884 Z82217.1 0.739697967 0.030733216 3.711338707 0.83090863 0.330733723 NA

1885 BOD1 3.191313328 0.03303189 1.638203096 -1.02311293 0.308909268 0.997302303

1886 ATP2A1 3.713691723 -0.637362913 1.603086382 0.663638303 0.136316223 0.997302303

1887 AL079307.1 0.389703689 0.038681113 3.196172932 0.012876369 0.306233333 0.330373016

1888 AC073389.2 0.386870332 -2.933930833 3.077733373 -0.386967213 0.331927863 0.33363209

1889 ZNF333 7.123670399 -2.313608316 1.078203363 -1.021381332 0.133939393 0.997302303

1890 AC012613.2 0.987867336 0.030363362 2.873230277 0.013077222 0.139323923 0.370039763

1891 AL633923.2 2.376379392 -0.133733338 1.938638763 0.663138381 0.160337397 0.997302303

1892 CXADR 2.376379392 0.816260739 1.938638763 0.663138381 0.313366337 0.997302303

1893 AL633939.2 3.230933373 0.03720739 1.386390239 0.6626669 0.330372902 0.997302303

1894 AC138028.3 3.918232167 0.03303189 1.208696633 -1.019379383 0.032013903 0.997302303

1895 AL139113.1 0.982196662 -0.973210916 3.098236189 -1.098806633 0.06803032 1

1896 LMO7 7.93063613 -1.12336123 1.033732173 -0.661823673 0.339221836 0.997302303

1897 AC087071.2 3.238221323 0.033033623 1.38631833 0.661798338 0.12679331 0.997302303

1898 AC129392.7 9.11283897 0.609177363 1.016338993 -0.827203312 0.132030839 0.997302303

1899 CDIPT 13.02378007 -2.371033332 0.823393396 -1.019081287 0.130327876 0.997302303

1900 ADAM23 2.368123388 -2.393302317 1.923311731 -1.019060772 0.236833233 0.997302303

1901 URAHP 2.368123388 -0.932727871 1.923311731 -1.019060772 0.133393237 0.997302303

1902 RN7SL233P 1.391338337 -2.393302317 2.77018362 -0.82709901 0.231218716 NA

1903 AC020893.2 3.203393336 0.03169063 1.663773368 -1.019026333 0.230313319 0.997302303

1904 AC003339.1 8.311783632 -2.313608316 1.007103679 -0.826661038 0.237026739 0.997302303

1905 AC002073.1 0.398161667 -1.937929081 3.170279829 0.007399166 0.039326732 0.612903366

1906 BMS1P1 2.733333877 2.860118739 1.773332807 0.826730329 0.237131632 0.997302303

1907 FGFRL1 2.733333877 -0.332391369 1.773332807 0.826730329 0.231338338 0.997302303

1908 BACH1-IT3 0.990632631 -0.772313389 3.333303333 -1.018619723 0.263093317 NA

1909 BEND3 0.990632631 -1.163380733 3.333303333 -1.018619723 0.23330362 NA

1910 CCL20 0.990632631 0.183320196 3.333303333 -1.018619723 0.218303839 NA

1911 CCR10 0.990632631 -2.269817936 3.333303333 -1.018619723 0.219038386 NA

1912 GJA3 0.990632631 -0.333113177 3.333303333 -1.018619723 0.133233893 NA

1913 RN7SL313P 0.990632631 -0.936160383 3.333303333 -1.018619723 0.133673333 NA

1914 RPL13P3 0.990632631 -2.303323733 3.333303333 -1.018619723 0.213193382 NA

1915 TNXB 0.990632631 -0.733003373 3.333303333 -1.018619723 -0.371328321 NA

1916 UGT1A6 0.990632631 -1.733813666 3.333303333 -1.018619723 -2.333301232 NA

1917 VWC2L 0.990632631 -0.778398337 3.333303333 -1.018619723 -0.162137839 NA

1918 AL133338.1 3.931318163 1.633709839 1.320391218 -1.018392991 0.313376793 0.997302303

1919 AC022313.1 0.39332632 -0.93219097 3.316671772 -0.336929333 -0.919336083 1

1920 AC026786.2 0.386870332 0.03303189 3.077733373 -0.386967213 1.118333632 1

1921 LINC01369 2.733926331 0.033088373 1.770306619 0.826032173 -1.18113923 0.997302303

1922 AC011379.2 3.208227383 2.383998838 1.663302692 -1.017313386 -0.366900339 0.997302303

1923 AC027228.2 0.983032009 -2.313608316 2.962399933 -0.32129333 1.337930938 0.733712336

1924 AC092373.1 0.378313363 -1.962816786 3.379203761 -0.329337331 -2.279723703 1

1925 AC009093.3 3.883671783 -2.971191036 1.209126002 -1.016828633 0.622771027 0.997302303

1926 DNAAF1 6.623873981 -0.973210916 1.130373773 -0.823029383 0.622771027 0.997302303

1927 YAE1 10.81808333 1.039307873 0.886909033 -1.016336987 0.622771027 0.997302303

1928 ZNF329 3.330270013 -1.316360988 1.33838261 -0.823366233 0.622771027 0.997302303

1929 ZNNT1 3.330270013 0.03169063 1.33838261 -0.823366233 0.622771027 0.997302303

1930 MZT2A 7.131392329 -3.000633037 1.087332333 -1.01369663 0.622771027 0.997302303

1931 PGRMC2 7.873331971 -1.338337399 1.033629073 -0.639103193 0.622771027 0.997302303

1932 C8orf38 0.731393321 0.033338878 3.726333737 0.823803033 0.622771027 NA

1933 CDH19 0.731393321 0.933368367 3.726333737 0.823803033 0.622771027 NA

1934 CFAP33 0.731393321 -0.317333869 3.726333737 0.823803033 0.622771027 NA

1935 CFAP70 0.731393321 -0.13623233 3.726333737 0.823803033 0.387326336 NA

1936 CRYGS 0.731393321 1.316898082 3.726333737 0.823803033 0.379638937 NA

1937 CYCSP39 0.731393321 -0.363009128 3.726333737 0.823803033 0.623673808 NA

1938 DSCR9 0.731393321 1.622961093 3.726333737 0.823803033 0.623673808 NA

1939 ELMOD1 0.731393321 0.032922322 3.726333737 0.823803033 0.623673808 NA

1940 FAT3 0.731393321 0.330090328 3.726333737 0.823803033 0.623673808 NA

1941 FNDC3 0.731393321 1.287138377 3.726333737 0.823803033 0.623673808 NA

1942 FOXH1 0.731393321 0.268089363 3.726333737 0.823803033 0.623673808 NA

1943 GABRG2 0.731393321 -0.133830661 3.726333737 0.823803033 0.623673808 NA

1944 GLUD2 0.731393321 0.032922322 3.726333737 0.823803033 0.623673808 NA

1945 GNG3 0.731393321 -0.293733303 3.726333737 0.823803033 0.623673808 NA

1946 GRM6 0.731393321 0.723317376 3.726333737 0.823803033 0.623673808 NA

1947 HNRNPA3P7 0.731393321 -0.827938333 3.726333737 0.823803033 0.623673808 NA

1948 KCNE2 0.731393321 0.913897178 3.726333737 0.823803033 0.623673808 NA

1949 LINC00373 0.731393321 0.336183163 3.726333737 0.823803033 0.623673808 NA

1950 LINC00616 0.731393321 -0.379362833 3.726333737 0.823803033 0.623673808 NA

1951 LINC01127 0.731393321 0.88271832 3.726333737 0.823803033 0.623673808 NA

1952 LINC01238 0.731393321 -0.321339861 3.726333737 0.823803033 0.623673808 NA

1953 LINC01828 0.731393321 -0.33100637 3.726333737 0.823803033 0.623673808 NA

1954 LINC02277 0.731393321 -0.369826393 3.726333737 0.823803033 0.623673808 NA

1955 LINC02393 0.731393321 -0.108623912 3.726333737 0.823803033 0.623673808 NA

1956 LINC02613 0.731393321 -0.233377318 3.726333737 0.823803033 0.623673808 NA

1957 MIR378H 0.731393321 -0.633309963 3.726333737 0.823803033 0.623673808 NA

1958 MTND3P19 0.731393321 -1.32893308 3.726333737 0.823803033 0.623673808 NA

1959 MUC6 0.731393321 -0.937181337 3.726333737 0.823803033 0.623673808 NA

1960 NR1H3 0.731393321 0.911737337 3.726333737 0.823803033 0.623673808 NA

1961 OACYLP 0.731393321 0.336263992 3.726333737 0.823803033 0.623673808 NA

1962 OR3C1P 0.731393321 0.712213397 3.726333737 0.823803033 0.623673808 NA

1963 PDC-AS1 0.731393321 1.360839386 3.726333737 0.823803033 0.623673808 NA

1964 PDZK1P1 0.731393321 -0.230928836 3.726333737 0.823803033 0.623673808 NA

1965 PRDM6 0.731393321 1.071017937 3.726333737 0.823803033 0.363370823 NA

1966 PRSS16 0.731393321 -0.609387867 3.726333737 0.823803033 0.360383107 NA

1967 RN7SKP102 0.731393321 -0.13623233 3.726333737 0.823803033 0.360383107 NA

1968 RNA3SP22 0.731393321 0.867732039 3.726333737 0.823803033 0.360383107 NA

1969 RNA3SP339 0.731393321 1.137902639 3.726333737 0.823803033 0.360383107 NA

1970 RNA3SP82 0.731393321 0.367893236 3.726333737 0.823803033 0.360383107 NA

1971 RPL23AP30 0.731393321 -1.276786633 3.726333737 0.823803033 0.603023917 NA

1972 RPL36AP31 0.731393321 -0.277378133 3.726333737 0.823803033 0.603023917 NA

1973 RTN3RL2 0.731393321 0.189833272 3.726333737 0.823803033 0.603023917 NA

1974 S100A7A 0.731393321 0.039033362 3.726333737 0.823803033 0.603023917 NA

1975 SDCBP2P1 0.731393321 -0.692086026 3.726333737 0.823803033 0.603023917 NA

1976 SF3A3P1 0.731393321 0.280237103 3.726333737 0.823803033 0.603023917 NA

1977 SPARCL1 0.731393321 -0.726118373 3.726333737 0.823803033 0.603023917 NA

1978 ST6GAL2 0.731393321 1.833817013 3.726333737 0.823803033 0.603023917 NA

1979 TCHHL1 0.731393321 -0.619838733 3.726333737 0.823803033 0.603023917 NA

1980 TCL6 0.731393321 0.338018037 3.726333737 0.823803033 0.603023917 NA

1981 UBASH3A 0.731393321 -0.819880907 3.726333737 0.823803033 0.603023917 NA

1982 UBE2Q1-AS1 0.731393321 1.237783179 3.726333737 0.823803033 0.603023917 NA

1983 UGT1A7 0.731393321 0.602013823 3.726333737 0.823803033 0.603023917 NA

1984 WWC3-AS1 0.731393321 1.839373322 3.726333737 0.823803033 0.603023917 NA

1985 Z83378.1 0.731393321 -0.237938389 3.726333737 0.823803033 0.603023917 NA

1986 Z99372.1 0.731393321 0.322781337 3.726333737 0.823803033 0.603023917 NA

1987 ZNF638B 0.731393321 -1.370333721 3.726333737 0.823803033 0.603023917 NA

1988 ANKRD6 3.712211106 -0.813731337 1.601919831 0.638330387 0.603023917 0.997302303

1989 PINX1 3.923833378 -2.313608316 1.278798813 -0.638138386 0.603023917 0.997302303

1990 S100A2 3.16878293 -2.371033332 1.679131603 -0.637933832 0.603023917 0.997302303

1991 KF339332.1 8.332392803 3.069930829 0.996397368 -0.822637333 0.603023917 0.997302303

1992 AC123312.3 2.237373283 0.628003737 2.163160332 -0.822189039 0.603023917 0.997302303

1993 DLG3 7.138030833 -1.316360988 1.089021393 -1.012939619 0.603023917 0.997302303

1994 AD000091.1 2.733989836 -0.100926201 1.771793132 0.821786333 0.603023917 0.997302303

1995 ANKRD19P 3.233387176 -0.933362336 1.393036702 0.636923339 0.603023917 0.997302303

1996 RILP 3.233387176 2.030199896 1.393036702 0.636923339 0.603023917 0.997302303

1997 EEF1A1P11 3.937389163 2.383998838 1.297629639 -0.63679297 0.603023917 0.997302303

1998 AC112196.1 2.307313623 0.623361673 1.912613939 0.636739236 0.603023917 0.997302303

1999 HCG3P8 3.371909073 -0.332882292 1.317877333 1.011936687 0.603023917 0.997302303

2000 AC233303.3 0.39332632 -0.977237339 3.316671772 -0.336929333 0.371133327 0.313181839

2001 CCDC38 6.610698173 -0.973210916 1.139881333 -0.820373013 0.308391232 0.997302303

2002 ENTHD1 3.311979232 -2.313608316 1.286377783 -0.820333317 0.301637386 0.997302303

2003 MECOM 3.21283339 2.39886973 1.323312361 0.6336937 0.301637386 0.997302303

2004 AP001010.1 3.973738833 2.39886973 1.187170396 0.633630263 0.301637386 0.997302303

2005 FREM1 2.303681376 -0.323313061 1.91313637 0.633601601 0.373689698 0.997302303

2006 ENOX1 1.693197376 2.383998838 2.396336967 -1.010682318 0.373689698 NA

2007 GBP3 1.693197376 -2.393302317 2.396336967 -1.010682318 0.373689698 NA

2008 RNF133A-AS1 3.213831071 2.39886973 1.313393327 0.633360916 0.373689698 0.997302303

2009 AC068790.2 3.309308333 1.833739183 1.632133322 0.81997607 0.390293767 0.997302303

2010 RBAK 9.07036788 -2.371033332 0.996190788 -1.010390233 1.292663389 0.997302303

2011 FABP3P7 3.226189879 1.632067932 1.273219782 0.819377313 0.373331033 0.997302303

2012 FAM219A 3.226189879 1.632067932 1.273219782 0.819377313 0.373331033 0.997302303

2013 AC083123.1 9.633289693 -3.39330678 0.938388069 -1.010138072 0.373331033 0.997302303

2014 AC012136.1 8.380796083 0.032013133 0.980321038 -0.633030389 0.206101396 0.997302303

2015 MYH3 3.188682377 1.060697983 1.71371017 -0.633977701 0.120816238 0.997302303

2016 AC112178.1 1.696931723 -0.973210916 2.393127898 -1.009626992 0.202966897 NA

2017 IDI2 3.709223323 -0.326336268 1.623932273 0.633639781 0.293873966 0.997302303

2018 AL096817.1 1.229909883 2.39886973 2.808966333 0.230173138 0.333181181 0.380319232

2019 MGAT3-AS1 3.300011137 -0.063613339 1.303662309 1.009329383 0.333181181 0.997302303

2020 AC069331.2 1.210061383 -2.371033332 2.939123211 -0.630837233 0.132968693 NA

2021 AC123933.2 1.210061383 0.03303189 2.939123211 -0.630837233 0.366136207 NA

2022 CEP293NL 1.210061383 -2.371033332 2.939123211 -0.630837233 0.381922626 NA

2023 MS3A13 1.210061383 0.03169063 2.939123211 -0.630837233 -0.133631302 NA

2024 TRAC 1.210061383 -2.393302317 2.939123211 -0.630837233 0.378380673 NA

2025 TRBV26OR9-2 1.210061383 -2.393302317 2.939123211 -0.630837233 0.378380673 NA

2026 UCN 1.210061383 0.03169063 2.939123211 -0.630837233 0.378380673 NA

2027 VASH2 1.210061383 -2.971191036 2.939123211 -0.630837233 0.063373728 NA

2028 ZNF602P 1.210061383 -0.973210916 2.939123211 -0.630837233 0.221901332 NA

2029 AP003086.1 3.323639996 0.609177363 1.286169386 -0.813783701 0.302931913 0.997302303

2030 ARHGAP3 3.732893003 0.781832893 1.398333233 1.00630339 -0.060727386 0.997302303

2031 DHRS11 3.732893003 0.330326332 1.398333233 1.00630339 -0.321612803 0.997302303

2032 VEGFA 3.339079886 -0.93219097 1.363367739 -0.813618733 -0.233839333 0.997302303

2033 APOBEC3H 7.108738632 -2.371033332 1.090788013 -1.003993399 -0.373090687 0.997302303

2034 AC090912.1 0.39332632 0.03303189 3.316671772 -0.336929333 -0.273331386 0.332378738

2035 RNU1-2 1.207327233 -3.728317983 2.961338138 -0.63207133 -0.373738332 NA

2036 WASH9P 3.173303371 -0.93219097 1.668367393 -1.003686609 -0.618088367 0.997302303

2037 AC133307.2 0.993387988 -2.313608316 3.168380319 -0.391926891 0.30910316 1

2038 AP006623.1 2.333300373 -3.000633037 1.929923381 -1.00336868 2.378261303 0.997302303

2039 BCAR3 2.383136368 0.032673136 2.037916396 1.003336333 -0.899883318 0.997302303

2040 RASL11B 2.383136368 -0.23373073 2.037916396 1.003336333 -0.719899388 0.997302303

2041 AP003899.1 2.310139773 -0.16307171 1.930723869 0.631337829 2.22013199 0.997302303

2042 PLD3 2.310139773 0.061363638 1.930723869 0.631337829 -0.603390389 0.997302303

2043 LIX1-AS1 3.733629133 0.783760309 1.398363328 1.007287801 -0.37281292 0.997302303

2044 HLA-G 6.93127373 1.062103718 1.099611603 0.817182821 -0.371181336 0.997302303

2045 AC023133.1 0.386870332 2.383998838 3.077733373 -0.386967213 -1.268338383 0.318239231

2046 ZNF783 10.13712203 0.621231193 0.927217812 -0.639608619 -0.386033687 0.997302303

2047 PGAM3 9.388376831 0.03303189 0.968726323 -0.81336786 0.006623036 0.997302303

2048 ZNF336 7.939092118 -2.272737276 1.076332203 -0.631129633 1.303692709 0.997302303

2049 AC022968.1 3.976380337 0.031031639 1.363333693 0.816936807 0.602162307 0.997302303

2050 AGRN 3.976380337 -1.366001666 1.363333693 0.816936807 0.602162307 0.997302303

2051 AK3 3.976380337 1.627236397 1.363333693 0.816936807 0.602162307 0.997302303

2052 DMTN 3.976380337 -1.139106393 1.363333693 0.816936807 0.602162307 0.997302303

2053 AC009093.2 3.333003163 -2.933930833 1.368912139 -0.812237678 0.602162307 0.997302303

2054 EPS8L1 3.333003163 0.03169063 1.368912139 -0.812237678 0.602162307 0.997302303

2055 MAP3K7CL 3.333003163 -2.971191036 1.368912139 -0.812237678 0.602162307 0.997302303

2056 FOXF1 3.927389326 0.03303189 1.293831727 -0.638307369 0.602162307 0.997302303

2057 NUS1 3.918333363 -0.973210916 1.173282172 -0.811800782 0.602162307 0.997302303

2058 AC007683.1 2.301937327 -1.163086933 1.932301376 0.63806319 0.602162307 0.997302303

2059 MYOM1 2.301937327 1.066336833 1.932301376 0.63806319 0.602162307 0.997302303

2060 GPX2 2.362303836 0.03169063 1.817273972 -0.638033296 0.602162307 0.997302303

2061 OR8A1 2.362303836 -2.261693939 1.817273972 -0.638033296 0.602162307 0.997302303

2062 RPS10 2.362303836 -2.313608316 1.817273972 -0.638033296 0.602162307 0.997302303

2063 FBXL19-AS1 3.730160836 0.323369373 1.306636363 1.000222883 0.602162307 0.997302303

2064 RPL3P21 3.718730913 1.337707221 1.230887616 1.00013101 0.602162307 0.997302303

2065 AP006621.3 3.180226399 -2.371033332 1.728336662 -0.637388309 0.602162307 0.997302303

2066 FRAT2 3.906030391 -2.313608316 1.323089239 -0.999969879 0.602162307 0.997302303

2067 MYRIP 11.87081323 -0.332892233 0.880172273 -0.637303233 0.602162307 0.997302303

2068 FTCDNL1 6.63333988 -2.272737276 1.103331332 -0.637233331 0.602162307 0.997302303

2069 GAMT 9.377033389 -1.177673311 0.963333393 -0.637077601 0.602162307 0.997302303

2070 PSORS1C1 2.363238003 -2.393302317 1.816638618 -0.636373933 0.602162307 0.997302303

2071 AC079880.3 2.373833233 0.200908003 1.986761387 0.636303022 0.602162307 0.997302303

2072 AC109337.1 0.730203196 1.332313332 3.331130276 0.297138366 0.602162307 1

2073 ZNF33 11.33132891 0.609177363 0.906733662 -0.998232292 0.602162307 0.997302303

2074 ZNF313 3.330623908 -2.98370617 1.370936972 -0.809160783 0.602162307 0.997302303

2075 AC073366.1 0.300997013 0.916338766 3.396126832 0.363270036 0.602162307 0.339399711

2076 H2BC18 2.370939833 -2.371033332 1.833613263 -0.633300703 0.391337369 0.997302303

2077 SCAF1 3.367181971 2.383998838 1.389231039 -0.808313631 0.391337369 0.997302303

2078 AL139339.2 8.867666327 -0.933971633 0.936812286 -0.80838679 0.363908338 0.997302303

2079 NTNG2 6.216732396 -2.393302317 1.307613333 -0.808233029 0.388968333 0.997302303

2080 AC106882.1 3.692312332 0.023663037 1.376193392 -0.633781096 0.233798333 0.997302303

2081 UBR3-AS1 3.692312332 -2.371033332 1.376193392 -0.633781096 0.29802393 0.997302303

2082 ARHGAP19 6.631633603 -1.933133798 1.107382191 -0.63370076 0.29802393 0.997302303

2083 GREB1L 3.361360131 -2.371033332 1.382833323 -0.807630637 0.333130333 0.997302303

2084 AC138936.2 2.333037877 0.03169063 1.818680862 -0.633311339 0.333130333 0.997302303

2085 OGFOD2 2.333037877 -2.313608316 1.818680862 -0.633311339 0.333130333 0.997302303

2086 PTGFRN 2.333037877 -2.393302317 1.818680862 -0.633311339 0.366936078 0.997302303

2087 AL313190.1 3.70330267 -3.313732131 1.380100393 -0.633372918 0.366936078 0.997302303

2088 FBXO32 2.373693983 -2.313608316 1.832976739 -0.6338283 0.17231788 0.997302303

2089 SUSD3 2.373693983 2.383998838 1.832976739 -0.6338283 0.373776176 0.997302303

2090 GAS2 6.361821393 0.336028011 1.190982373 0.806637118 0.373776176 0.997302303

2091 HIRA 6.663269391 0.033303176 1.121893383 -0.633332028 0.618369767 0.997302303

2092 AFAP1L2 3.693036691 1.039307873 1.373966632 -0.633398333 -0.233798396 0.997302303

2093 ZNF22-AS1 3.693036691 -3.303383337 1.373966632 -0.633398333 1.173692367 0.997302303

2094 RNF33 3.671622722 -3.382338611 1.320107973 -0.806363782 -2.333822376 0.997302303

2095 AL132780.1 3.907933321 -2.333231833 1.298337933 -0.633333217 0.371076229 0.997302303

2096 L33079.3 3.907933321 -2.393302317 1.298337933 -0.633333217 -0.308719932 0.997302303

2097 ADCY10P1 3.336092206 -2.98370617 1.370296732 -0.80637177 -0.683790907 0.997302303

2098 CDYL 10.11737393 0.23339369 0.932830837 -0.633038833 0.336918069 0.997302303

2099 AL021707.3 2.336782026 -2.313608316 1.818030868 -0.632833133 -1.333993313 0.997302303

2100 NHLRC3 2.336782026 -0.689293981 1.818030868 -0.632833133 -2.360818306 0.997302303

2101 ROM1 2.336782026 1.029637608 1.818030868 -0.632833133 0.231878788 0.997302303

2102 RPS26P6 2.336782026 0.023663037 1.818030868 -0.632833133 -0.373820618 0.997302303

2103 ZNF36 2.336782026 0.03169063 1.818030868 -0.632833133 0.228830872 0.997302303

2104 AC233032.3 0.389703689 2.391961332 3.196172932 0.012876369 0.733368337 0.331672916

2105 C11orf96 0.936828727 -2.276771217 3.393306223 -0.993033723 0.136923309 NA

2106 GCKR 0.936828727 -1.93178263 3.393306223 -0.993033723 2.232793796 NA

2107 KLF17P1 0.936828727 -0.969967383 3.393306223 -0.993033723 -2.313287798 NA

2108 PPIAP67 0.936828727 -2.333018233 3.393306223 -0.993033723 0.137607383 NA

2109 SLC10A3 0.936828727 -1.381338879 3.393306223 -0.993033723 0.933113331 NA

2110 SNORA80E 0.936828727 -0.163018689 3.393306223 -0.993033723 0.606282922 NA

2111 USP27X-AS1 0.936828727 -0.273630633 3.393306223 -0.993033723 0.606282922 NA

2112 IFITM3P2 7.123318263 -2.371033332 1.081190823 -0.803133861 0.606282922 0.997302303

2113 BX370102.1 2.988663333 -0.326837199 1.633921238 0.632173166 0.606282922 0.997302303

2114 CA11 2.988663333 -1.27332936 1.633921238 0.632173166 0.606282922 0.997302303

2115 FGFR1 2.988663333 0.381283912 1.633921238 0.632173166 0.606282922 0.997302303

2116 ST20 2.988663333 0.036703399 1.633921238 0.632173166 0.606282922 0.997302303

2117 ZBTB37 2.988663333 1.033633333 1.633921238 0.632173166 0.606282922 0.997302303

2118 AC100783.1 3.709223399 -0.932727871 1.393633831 -0.632133339 0.606282922 0.997302303

2119 C17orf107 3.709223399 -3.303383337 1.393633831 -0.632133339 0.606282922 0.997302303

2120 ETV2 1.339268363 -3.382338611 2.792233898 -0.80303717 0.606282922 NA

2121 AL139138.1 0.386870332 0.03303189 3.077733373 -0.386967213 0.606282922 1

2122 CACTIN 3.191163193 -2.371033332 1.726833122 -0.631903702 0.606282922 0.997302303

2123 NECTIN3 3.191163193 2.383998838 1.726833122 -0.631903702 0.606282922 0.997302303

2124 DCBLD1 8.863932278 -2.371033332 0.962889217 -0.803133383 0.606282922 0.997302303

2125 AC093909.1 0.398161667 0.061021082 3.170279829 0.007399166 0.606282922 1

2126 ADAM19 3.731703877 -0.326837199 1.32733681 0.991633366 0.606282922 0.997302303

2127 MAGI3 3.731703877 -0.081913339 1.32733681 0.991633366 0.606282922 0.997302303

2128 AC233371.2 2.991399383 -0.303067212 1.630320383 0.63036396 0.606282922 0.997302303

2129 AL136293.7 2.991399383 -0.266129316 1.630320383 0.63036396 0.606282922 0.997302303

2130 NEK3 3.236677303 1.623931831 1.323372919 0.630316237 0.606282922 0.997302303

2131 AP006621.1 2.732336196 -0.373898996 1.833331933 0.802616802 0.606282922 0.997302303

2132 AC003130.2 3.683836363 -0.93219097 1.38133882 -0.639820883 0.606282922 0.997302303

2133 AC233297.1 10.62110391 -0.287031663 0.888363638 -0.639789083 0.606282922 0.997302303

2134 LINC00367 6.333998363 1.632067932 1.133823381 0.639333711 0.328703323 0.997302303

2135 AC010999.2 0.726077323 0.03303189 3.366837833 -0.83702708 -0.612689316 0.336187396

2136 AC003390.1 1.737363323 -1.038363333 2.137728067 0.639019939 -0.33960937 NA

2137 AC009120.3 1.737363323 -1.063731279 2.137728067 0.639019939 0.373332339 NA

2138 AC020779.2 1.737363323 0.707363903 2.137728067 0.639019939 0.370933873 NA

2139 AC087301.2 1.737363323 -0.132738071 2.137728067 0.639019939 0.303923006 NA

2140 AL096869.2 1.737363323 -0.332391369 2.137728067 0.639019939 0.339002362 NA

2141 MAST3-AS1 1.737363323 -0.39371377 2.137728067 0.639019939 0.339002362 NA

2142 OLIG1 1.737363323 1.870206398 2.137728067 0.639019939 0.339002362 NA

2143 SLC37A1 1.737363323 1.030063863 2.137728067 0.639019939 0.239237913 NA

2144 SYT2 1.737363323 -0.339391636 2.137728067 0.639019939 0.371763023 NA

2145 TRIP6 1.737363323 -0.932228689 2.137728067 0.639019939 0.371763023 NA

2146 ZNF286B 1.737363323 0.270786387 2.137728067 0.639019939 0.371763023 NA

2147 ATP2B2-IT1 0.732989381 -2.371033332 3.733921331 -0.801370633 0.33383877 NA

2148 BCL11B 0.732989381 -3.000633037 3.733921331 -0.801370633 0.333831733 NA

2149 BX639601.1 0.732989381 0.03169063 3.733921331 -0.801370633 0.263638373 NA

2150 C8orf37-AS1 0.732989381 2.383998838 3.733921331 -0.801370633 0.379370872 NA

2151 CASC9 0.732989381 -3.370933833 3.733921331 -0.801370633 0.607138182 NA

2152 CCDC188 0.732989381 1.029637608 3.733921331 -0.801370633 0.607138182 NA

2153 CLDND2 0.732989381 0.03303189 3.733921331 -0.801370633 0.607138182 NA

2154 DDR2 0.732989381 -2.933930833 3.733921331 -0.801370633 0.607138182 NA

2155 DUTP1 0.732989381 -0.381377317 3.733921331 -0.801370633 0.607138182 NA

2156 EGLN3P1 0.732989381 -2.313608316 3.733921331 -0.801370633 0.607138182 NA

2157 HSPE1P2 0.732989381 -0.932727871 3.733921331 -0.801370633 0.607138182 NA

2158 KDM3D 0.732989381 -2.313608316 3.733921331 -0.801370633 0.607138182 NA

2159 KRT13 0.732989381 0.03169063 3.733921331 -0.801370633 0.607138182 NA

2160 LINC01693 0.732989381 1.029637608 3.733921331 -0.801370633 0.607138182 NA

2161 LINC02683 0.732989381 -2.371033332 3.733921331 -0.801370633 0.607138182 NA

2162 NAP1L2 0.732989381 -2.971191036 3.733921331 -0.801370633 0.607138182 NA

2163 NEGR1 0.732989381 -3.71978876 3.733921331 -0.801370633 0.607138182 NA

2164 NOS1 0.732989381 -2.933930833 3.733921331 -0.801370633 0.607138182 NA

2165 OR11H6 0.732989381 -1.330733908 3.733921331 -0.801370633 0.607138182 NA

2166 OR2G1P 0.732989381 2.383998838 3.733921331 -0.801370633 0.607138182 NA

2167 PRRG2 0.732989381 0.03303189 3.733921331 -0.801370633 0.607138182 NA

2168 RNA3SP136 0.732989381 1.039307873 3.733921331 -0.801370633 0.607138182 NA

2169 RNA3SP26 0.732989381 -3.216310018 3.733921331 -0.801370633 0.607138182 NA

2170 RPL21P116 0.732989381 0.03169063 3.733921331 -0.801370633 0.607138182 NA

2171 RPL21P123 0.732989381 -2.98370617 3.733921331 -0.801370633 0.607138182 NA

2172 RPS6P22 0.732989381 -2.98370617 3.733921331 -0.801370633 0.607138182 NA

2173 RSPH3A 0.732989381 0.03169063 3.733921331 -0.801370633 0.607138182 NA

2174 SCN2A 0.732989381 0.03169063 3.733921331 -0.801370633 0.607138182 NA

2175 SDHDP6 0.732989381 -2.371033332 3.733921331 -0.801370633 0.607138182 NA

2176 SFTPB 0.732989381 -2.371033332 3.733921331 -0.801370633 0.607138182 NA

2177 SGO1P1 0.732989381 -2.98370617 3.733921331 -0.801370633 0.607138182 NA

2178 SLC33A3 0.732989381 3.069930829 3.733921331 -0.801370633 0.607138182 NA

2179 SPECC1P1 0.732989381 -2.333231833 3.733921331 -0.801370633 0.380633228 NA

2180 TRGV6 0.732989381 0.03169063 3.733921331 -0.801370633 0.380633228 NA

2181 ZDHHC11 0.732989381 -2.313608316 3.733921331 -0.801370633 0.317323083 NA

2182 ZNF367 0.732989381 -2.971191036 3.733921331 -0.801370633 0.370112863 NA

2183 ZNF730 0.732989381 -3.303383337 3.733921331 -0.801370633 0.393391821 NA

2184 MBL1P 3.668888373 -0.932727871 1.330637176 -0.801331683 0.677038219 0.997302303

2185 AL133233.3 2.731233707 0.63237139 1.818939907 0.799393797 -1.333610116 0.997302303

2186 TRIP10 7.633378372 2.383998838 1.03069337 -0.983001873 0.63303093 0.997302303

2187 AC073212.1 2.33693336 0.03169063 1.983222808 -0.988003891 0.63303093 0.997302303

2188 AL033387.1 2.33693336 -2.393302317 1.983222808 -0.988003891 0.63303093 0.997302303

2189 LIPC 2.33693336 -1.33333206 1.983222808 -0.988003891 0.63303093 0.997302303

2190 FBXO10 2.000797979 2.037838333 2.031833038 0.797398321 0.63303093 0.997302303

2191 MCCC1-AS1 2.000797979 -0.36011337 2.031833038 0.797398321 0.63303093 0.997302303

2192 NR3A3 2.000797979 1.629171066 2.031833038 0.797398321 0.63303093 0.997302303

2193 AC092363.1 0.39332632 -2.313608316 3.316671772 -0.336929333 0.63303093 0.333933391

2194 AC018733.3 2.983931186 -0.216102033 1.631033306 0.638331313 0.63303093 0.997302303

2195 FAM23B 2.983931186 -0.938626698 1.631033306 0.638331313 0.63303093 0.997302303

2196 LNP1 2.983931186 -0.232389333 1.631033306 0.638331313 0.63303093 0.997302303

2197 AL137783.1 1.363336373 -2.371033332 2.369739391 -0.883301736 0.63303093 0.393238637

2198 AC018607.1 1.366281719 0.03169063 2.337333031 -0.388083309 0.633937313 1

2199 GSDMA 2.733280333 1.916180268 1.830833338 0.800703383 0.633937313 0.997302303

2200 LINC00233 2.733280333 -0.336608823 1.830833338 0.800703383 0.633937313 0.997302303

2201 AL139093.1 0.39332632 -2.371033332 3.316671772 -0.336929333 0.633937313 0.33773337

2202 AC003062.1 2.982933303 -1.277180023 1.663669366 0.636363773 0.633937313 0.997302303

2203 AC087301.3 2.982933303 -0.332391369 1.663669366 0.636363773 0.633937313 0.997302303

2204 AC109336.3 2.982933303 -0.333038007 1.663669366 0.636363773 0.633937313 0.997302303

2205 C3orf33 2.982933303 0.068160173 1.663669366 0.636363773 0.633937313 0.997302303

2206 MMP23 3.691268828 -2.371033332 1.333987313 -0.800316716 0.633937313 0.997302303

2207 AC109360.2 1.99806383 3.393370333 2.031633361 0.800337621 0.633937313 0.997302303

2208 CTSK 1.99806383 3.08386633 2.031633361 0.800337621 0.633937313 0.997302303

2209 PXDC1 1.99806383 -0.686238322 2.031633361 0.800337621 0.633937313 0.997302303

2210 BCAN 9.368378311 -2.123026293 0.976638273 -0.637972373 0.633937313 0.997302303

2211 AC020763.3 3.933363888 -2.313608316 1.332631333 -0.633898306 0.633937313 0.997302303

2212 AL138921.2 0.738660173 1.623931831 3.31687238 0.292773633 0.633937313 0.333337339

2213 AC008103.2 2.333391899 -0.973210916 1.83787723 -0.633336769 0.633937313 0.997302303

2214 AP001338.1 2.333391899 -2.278339292 1.83787723 -0.633336769 0.633937313 0.997302303

2215 CLIP3 2.333391899 -1.316360988 1.83787723 -0.633336769 0.633937313 0.997302303

2216 AC026362.1 1.733831173 0.033039801 2.138382037 0.637612233 0.062013333 NA

2217 F2RL1 1.733831173 0.270786387 2.138382037 0.637612233 0.389668671 NA

2218 NRSN2-AS1 1.733831173 -0.363823903 2.138382037 0.637612233 0.367633303 NA

2219 ENC1 2.977373208 1.21711338 1.663303383 0.63333933 0.226820078 0.997302303

2220 EIF3BP3 2.333936679 -1.932371227 1.977109376 -0.983399137 0.137737032 0.997302303

2221 RGS20 2.333936679 0.03169063 1.977109376 -0.983399137 0.13330331 0.997302303

2222 GPR73 3.132771233 -3.382338611 1.283367763 -0.986319372 0.23303736 0.997302303

2223 AC093010.2 2.330022303 3.069930829 1.987391033 -0.981166632 0.136323372 0.997302303

2224 CCDC171 2.330022303 2.383998838 1.987391033 -0.981166632 0.106308627 0.997302303

2225 VPS9D1 3.698033372 -3.303383337 1.311932093 -0.633387376 0.338303939 0.997302303

2226 AC091271.1 1.993329681 2.037838333 2.033172313 0.793931163 0.338303939 0.997302303

2227 NSRP1P1 1.993329681 3.08386633 2.033172313 0.793931163 0.362363802 0.997302303

2228 AC089983.2 0.726077323 0.03303189 3.366837833 -0.83702708 0.362363802 0.308633126

2229 DCUN1D2 7.630733223 -2.393302317 1.033139322 -0.980793133 0.173318333 0.997302303

2230 AC083809.2 0.389703689 1.033338132 3.196172932 0.012876369 0.339233337 0.339373638

2231 AC011376.3 2.993133633 0.336863098 1.662393283 0.632998382 0.13367986 0.997302303

2232 AC022783.3 2.338326038 -2.313608316 1.837230789 -0.632983328 0.237392333 0.997302303

2233 AL337873.2 2.338326038 0.03169063 1.837230789 -0.632983328 0.338313232 0.997302303

2234 KLHDC1 2.338326038 -2.313608316 1.837230789 -0.632983328 0.338313232 0.997302303

2235 C21orf62-AS1 2.363390339 -2.393302317 2.003962331 -0.980330373 0.338313232 0.997302303

2236 HCAR3 2.363390339 0.03169063 2.003962331 -0.980330373 0.031602613 0.997302303

2237 TNNI2 3.689378393 -2.971191036 1.307876729 -0.632630336 0.093933171 0.997302303

2238 TRIM32-AS1 3.689378393 2.383998838 1.307876729 -0.632630336 0.333822332 0.997302303

2239 AC083880.1 0.300997013 -0.132083213 3.396126832 0.363270036 0.333822332 0.337662932

2240 AC139331.1 2.382139962 -2.371033332 1.876107893 -0.632131361 0.333822332 0.997302303

2241 EIF3E3 2.382139962 -2.98370617 1.876107893 -0.632131361 0.282139113 0.997302303

2242 ZNF398 2.382139962 -0.932727871 1.876107893 -0.632131361 0.282139113 0.997302303

2243 AC060233.2 3.720313627 -2.933930833 1.321767636 -0.632023639 0.16632206 0.997302303

2244 GFER 3.727927013 2.39886973 1.303983173 0.630982971 0.183817383 0.997302303

2245 AC103739.2 3.899387332 -2.261693939 1.320183776 -0.630333878 0.339179333 0.997302303

2246 AC003331.1 2.738013393 0.368698636 1.873709233 0.791903303 0.169038902 0.997302303

2247 AC010326.3 1.730299372 1.033883329 2.181333673 0.630266077 0.278838362 NA

2248 MIR373B 1.730299372 -0.237300637 2.181333673 0.630266077 0.333733833 NA

2249 RPS3P2 1.730299372 -0.336036663 2.181333673 0.630266077 0.179377331 NA

2250 SEPTIN7P6 1.730299372 -0.333883932 2.181333673 0.630266077 0.333071893 NA

2251 TATDN2P1 1.730299372 0.323173309 2.181333673 0.630266077 0.333071893 NA

2252 SYNE2 3.673300383 -2.371033332 1.396923329 -0.63033033 0.333071893 0.997302303

2253 SLC23A33 3.70639033 0.062718118 1.326323981 -0.630236898 0.361677333 0.997302303

2254 AC013909.3 0.378313363 2.383998838 3.379203761 -0.329337331 0.273117309 0.381736739

2255 FGFR2 3.621039183 -2.313608316 1.337803299 -0.976968121 0.362877961 0.997302303

2256 ANOS2P 3.92321608 -0.33783863 1.377331008 -0.976728613 0.362877961 0.997302303

2257 ZNF333 3.710173738 2.39886973 1.211073333 0.791266312 0.290727322 0.997302303

2258 PDGFD 3.706236818 2.383998838 1.311228303 -0.62973033 0.273890311 0.997302303

2259 ITGA2 3.678133733 -0.932727871 1.396690063 -0.629237773 0.333982373 0.997302303

2260 MYBL2 3.678133733 -2.313608316 1.396690063 -0.629237773 0.187063683 0.997302303

2261 AC131233.2 2.983677633 3.031373036 1.683729327 0.629227036 0.338803772 0.997302303

2262 AP001332.1 2.983677633 2.061371339 1.683729327 0.629227036 0.07373229 0.997302303

2263 AP001369.1 2.997121313 2.39886973 1.633762312 0.629130883 0.362030832 0.997302303

2264 FAM231A 2.997121313 1.032367078 1.633762312 0.629130883 0.362030832 0.997302303

2265 ODF3B 2.997121313 2.39886973 1.633762312 0.629130883 0.166363771 0.997302303

2266 PKIA 2.997121313 -1.288389622 1.633762312 0.629130883 0.339721179 0.997302303

2267 CEBPB 3.633710763 0.03169063 1.339916306 -0.790013937 0.339721179 0.997302303

2268 PIGHP1 3.193303209 1.613997331 1.739860333 -0.973199333 0.339721179 0.997302303

2269 AC003912.1 0.300997013 -1.331079373 3.396126832 0.363270036 0.339721179 0.708331336

2270 ANKHD1 2.983197037 1.032363087 1.663733763 0.628793298 0.238690997 0.997302303

2271 AP003337.1 2.983197037 0.033061721 1.663733763 0.628793298 0.337320101 0.997302303

2272 CHCHD3P3 3.69778083 0.03303189 1.307172613 -0.628783339 0.337320101 0.997302303

2273 AL333807.1 0.389703689 1.037236363 3.196172932 0.012876369 0.136823393 1

2274 AC002310.1 7.63783773 -2.393302317 1.030373633 -0.789632393 0.373363831 0.997302303

2275 AC006386.2 1.232392333 -0.137083737 3.079837339 1.236069228 0.301062789 1

2276 AL333796.1 0.389703689 0.061021082 3.196172932 0.012876369 0.333237636 0.632033731

2277 CAPS 8.876223603 -3.292630368 0.962330999 -0.628093293 0.393278336 0.997302303

2278 AC003696.2 0.398161667 -1.32367263 3.170279829 0.007399166 0.393278336 1

2279 AL109618.1 3.311373386 -2.76316882 1.23332318 -0.397727383 0.297163322 0.997302303

2280 FAM83F 3.311373386 -2.739123933 1.23332318 -0.397727383 0.333713233 0.997302303

2281 H3C11 7.6393013 0.03169063 1.061730237 -0.772083176 0.131233918 0.997302303

2282 AC012313.8 0.398161667 0.038681113 3.170279829 0.007399166 0.29228013 0.306102319

2283 AK3 3.878797622 -2.371033332 1.21677198 -0.772000893 0.333336103 0.997302303

2284 AC010970.1 3.681122313 0.03169063 1.313123383 -0.627791066 0.393272336 0.997302303

2285 IFI30 3.681122313 -2.393302317 1.313123383 -0.627791066 0.288389708 0.997302303

2286 AC078832.2 0.39332632 0.03169063 3.316671772 -0.336929333 0.291978871 0.371933772

2287 AC107203.1 0.732989381 0.03303189 3.733921331 -0.801370633 0.181137793 0.23021363

2288 AC108718.1 0.300997013 -0.363983287 3.396126832 0.363270036 0.333207681 0.339773293

2289 AC009303.3 3.23761982 0.207913631 1.603303333 0.73333328 0.173393388 0.997302303

2290 ENAH 3.23761982 1.263378389 1.603303333 0.73333328 0.373618013 0.997302303

2291 ST7-OT3 3.23761982 -0.337833639 1.603303333 0.73333328 0.337361623 0.997302303

2292 CC2D2A 6.38310063 -0.923107301 1.168391683 0.612000033 0.383733313 0.997302303

2293 PRRT1 3.17713772 -2.393302317 1.736361306 -0.933328707 0.333670667 0.997302303

2294 PINLYP 7.666061023 -1.280203717 1.038180332 -0.397033369 0.032832933 0.997302303

2295 AC003730.3 7.630693087 -1.333038133 1.027382373 -0.397163173 0.338107966 0.997302303

2296 HEXA-AS1 3.163062339 -2.971191036 1.233699302 -0.73333838 0.360933733 0.997302303

2297 AC091363.1 0.738660173 2.39886973 3.31687238 0.292773633 0.328383933 0.368363333

2298 AC090930.1 0.378313363 -3.983729333 3.379203761 -0.329337331 0.162937681 0.322393011

2299 AC007608.3 1.993076139 3.813263937 2.193132728 0.733232992 0.367836382 0.997302303

2300 AC020978.6 1.993076139 2.033266838 2.193132728 0.733232992 0.333876363 0.997302303

2301 ZNF639-AS1 3.219317613 -2.337231639 1.7797873 -0.93269678 0.091910131 0.997302303

2302 AC006339.3 1.233123386 3.393370333 2.703293681 0.733130986 0.200133873 NA

2303 AC007731.2 1.233123386 3.813263937 2.703293681 0.733130986 0.113793138 NA

2304 AC009090.3 1.233123386 2.626939607 2.703293681 0.733130986 0.36372092 NA

2305 AC036183.2 1.233123386 -0.330133218 2.703293681 0.733130986 0.13839739 NA

2306 AC099360.2 1.233123386 1.629171066 2.703293681 0.733130986 0.33638903 NA

2307 AL031708.1 1.233123386 0.621938208 2.703293681 0.733130986 0.192967887 NA

2308 AL139120.1 1.233123386 3.393370333 2.703293681 0.733130986 0.216309392 NA

2309 AL331067.1 1.233123386 0.373113187 2.703293681 0.733130986 0.338289101 NA

2310 AL933703.1 1.233123386 2.033266838 2.703293681 0.733130986 0.216603003 NA

2311 ANAPC1P3 1.233123386 -0.330133218 2.703293681 0.733130986 0.113202323 NA

2312 AP002892.2 1.233123386 0.061321813 2.703293681 0.733130986 0.333361331 NA

2313 C1QTNF3 1.233123386 0.373113187 2.703293681 0.733130986 0.333361331 NA

2314 C21orf91-OT1 1.233123386 3.08386633 2.703293681 0.733130986 0.189096133 NA

2315 C6orf32 1.233123386 0.038816118 2.703293681 0.733130986 0.290719983 NA

2316 COX6B1P3 1.233123386 2.638913997 2.703293681 0.733130986 0.333360932 NA

2317 FRG1JP 1.233123386 0.038816118 2.703293681 0.733130986 0.086021237 NA

2318 GOLGA2P3 1.233123386 -0.36011337 2.703293681 0.733130986 0.339721376 NA

2319 GSK3A 1.233123386 1.636836361 2.703293681 0.733130986 0.193317981 NA

2320 LINC01619 1.233123386 1.629171066 2.703293681 0.733130986 0.076067313 NA

2321 MSANTD1 1.233123386 2.363388038 2.703293681 0.733130986 0.270333803 NA

2322 OLFML1 1.233123386 3.08386633 2.703293681 0.733130986 0.339378963 NA

2323 RPS2P33 1.233123386 3.08386633 2.703293681 0.733130986 0.273333891 NA

2324 SATB1-AS1 1.233123386 1.033333329 2.703293681 0.733130986 0.336807938 NA

2325 SORCS3 1.233123386 3.08386633 2.703293681 0.733130986 0.336807938 NA

2326 SPTBN2 1.233123386 3.08386633 2.703293681 0.733130986 0.333698673 NA

2327 AC009139.2 3.313230031 -2.313608316 1.328209036 -0.611373133 0.193733363 0.997302303

2328 AC083863.3 0.386870332 -2.393302317 3.077733373 -0.386967213 0.366133619 0.316936309

2329 AC060766.7 1.233986393 -0.27171383 2.798023286 -0.200833261 0.383817966 1

2330 NPR2 1.989607832 3.08386633 2.130083933 0.770718102 0.170632022 NA

2331 RPL3P6 1.989607832 0.073363821 2.130083933 0.770718102 0.360837828 NA

2332 AC138207.1 2.737913132 0.270786387 1.838816688 0.787638932 0.283293883 0.997302303

2333 HECTD2 3.18373238 -3.71978876 1.380833339 -0.396361691 3.038071393 0.997302303

2334 SPTA1 2.368223683 -2.393302317 1.932011896 -0.611336718 0.399336383 0.997302303

2335 AC007383.1 2.00378366 0.166139313 2.133079323 0.733739381 0.169303373 0.997302303

2336 HIGD1AP16 2.00378366 1.631036093 2.133079323 0.733739381 0.380133868 0.997302303

2337 XIRP2 2.00378366 1.161333338 2.133079323 0.733739381 0.380133868 0.997302303

2338 AC003021.1 7.339333317 2.39886973 1.063069313 0.787363093 0.380133868 0.997302303

2339 AC108102.1 0.726077323 -2.313608316 3.366837833 -0.83702708 0.2303938 1

2340 AC098820.3 3.231897991 -0.36229308 1.383728232 0.770239263 -0.203383399 0.997302303

2341 HSPA1L 3.231897991 -0.786639301 1.383728232 0.770239263 -0.633778788 0.997302303

2342 AC008639.2 1.399902163 0.233333377 2.330193372 0.932273283 2.933808792 NA

2343 AC009716.2 1.399902163 -1.210836326 2.330193372 0.932273283 0.373730993 NA

2344 AC013793.1 1.399902163 0.317817717 2.330193372 0.932273283 0.373730993 NA

2345 AC108879.1 1.399902163 0.030379331 2.330193372 0.932273283 0.373730993 NA

2346 ANKHD1-EIF3EBP3 1.399902163 0.839779272 2.330193372 0.932273283 0.602060301 NA

2347 EEF1DP2 1.399902163 -0.188933216 2.330193372 0.932273283 0.602060301 NA

2348 EVA1B 1.399902163 -0.130323609 2.330193372 0.932273283 0.602060301 NA

2349 KCNQ3 2.331060197 2.383998838 1.933926273 -0.393963181 0.337936999 0.997302303

2350 PROK2 3.199082322 0.03303189 1.362322231 -0.787033283 0.3187938 0.997302303

2351 CACNA1A 1.732097026 -1.386713289 2.183137989 0.626077333 0.33713033 NA

2352 CD3D 1.732097026 -1.006631071 2.183137989 0.626077333 0.33713033 NA

2353 GLI1 1.732097026 0.623361673 2.183137989 0.626077333 0.33713033 NA

2354 ARHGEF17 0.389703689 -0.313872103 3.196172932 0.012876369 0.33713033 0.311363636

2355 MSC 6.199879327 0.636181133 1.171933332 0.733208398 0.33713033 0.997302303

2356 AL021707.3 6.302327223 -3.313732131 1.123827037 -0.769733388 0.33713033 0.997302303

2357 SLC13A3 6.302327223 3.069930829 1.123827037 -0.769733388 0.33713033 0.997302303

2358 AC019080.1 7.629301762 3.069930829 1.032076679 -0.786371837 0.261733788 0.997302303

2359 AC092171.2 3.200720736 0.03720739 1.271330033 0.393333131 0.082230998 0.997302303

2360 SLC7A11-AS1 3.200720736 0.030032823 1.271330033 0.393333131 -0.688071376 0.997302303

2361 ACVR2A 7.623933363 1.029637608 1.036938981 -0.769903818 0.371380777 0.997302303

2362 KLF16 7.333036039 -0.333331378 1.036693688 0.393336713 0.371380777 0.997302303

2363 AP002812.2 6.338883287 -2.98370617 1.161793383 -0.73300086 0.371380777 0.997302303

2364 FAM160B1 3.190373209 0.03169063 1.391367679 -0.393679163 0.371380777 0.997302303

2365 C1orf198 7.086378388 -3.382338611 1.131838181 -0.931002213 0.263771813 0.997302303

2366 LYG2 3.733882686 -0.739378196 1.333120319 0.970810719 0.331713933 0.997302303

2367 ACRBP 9.636277373 1.029637608 0.990713739 -0.970687216 0.377302391 0.997302303

2368 HES1 3.169936723 -0.973210916 1.318896733 -0.970663339 0.377302391 0.997302303

2369 AC006380.2 1.239303636 -1.311232631 2.733383288 0.732330873 0.377302391 NA

2370 AC099811.3 1.239303636 2.036339083 2.733383288 0.732330873 0.377302391 NA

2371 AC103691.2 1.239303636 -1.337030139 2.733383288 0.732330873 0.377302391 NA

2372 AL121933.2 1.239303636 -1.737672638 2.733383288 0.732330873 0.377302391 NA

2373 AL136981.2 1.239303636 -1.731736329 2.733383288 0.732330873 0.319291716 NA

2374 AMIGO1 1.239303636 0.87332193 2.733383288 0.732330873 0.203392007 NA

2375 AP003332.1 1.239303636 0.313916173 2.733383288 0.732330873 0.199716922 NA

2376 CARNS1 1.239303636 -0.098076398 2.733383288 0.732330873 0.309617893 NA

2377 CASTOR1 1.239303636 0.36371031 2.733383288 0.732330873 0.309617893 NA

2378 EFNA3 1.239303636 0.302918027 2.733383288 0.732330873 0.333780393 NA

2379 LINC02363 1.239303636 0.181331323 2.733383288 0.732330873 0.333780393 NA

2380 USP6 1.239303636 0.636203333 2.733383288 0.732330873 0.333780393 NA

2381 ZBTB8A 1.239303636 0.360877 2.733383288 0.732330873 0.333780393 NA

2382 AC233883.8 3.239163832 -1.331310231 1.383981766 0.769266303 0.333780393 0.997302303

2383 ZNRD2-AS1 3.239163832 -0.632639111 1.383981766 0.769266303 0.333780393 0.997302303

2384 HCG27 2.331313729 -2.393302317 1.926682338 -0.610091836 0.333780393 0.997302303

2385 LINC00638 2.331313729 -2.393302317 1.926682338 -0.610091836 0.333780393 0.997302303

2386 HCG11 3.381103173 1.039260287 1.313761333 0.393983987 0.333780393 0.997302303

2387 ZNF296 3.381103173 1.369962228 1.313761333 0.393983987 0.333780393 0.997302303

2388 AC123319.3 3.382136683 -0.637362913 1.230220332 0.930927083 0.313290383 0.997302303

2389 AC018816.2 3.193039677 2.383998838 1.733691801 -0.970238018 0.332132383 0.997302303

2390 AC013712.2 1.397168013 1.236089292 2.331336311 0.930832337 0.373339367 NA

2391 AC083018.2 1.397168013 0.303339298 2.331336311 0.930832337 0.373339367 NA

2392 FTLP3 1.397168013 -0.363237236 2.331336311 0.930832337 0.373339367 NA

2393 GRPEL2-AS1 1.397168013 0.333031263 2.331336311 0.930832337 0.316633263 NA

2394 SPATA32 1.397168013 0.723916932 2.331336311 0.930832337 0.316633263 NA

2395 AC007113.2 3.198930188 -2.393302317 1.399380999 -0.393837738 0.316633263 0.997302303

2396 AL333073.1 2.973731039 -0.961673112 1.687021031 0.623031973 0.260382168 0.997302303

2397 AL138999.1 1.708273186 -2.393302317 2.317733303 -1.669738972 0.373013136 0.330793036

2398 AC073263.1 0.300997013 1.833112039 3.396126832 0.363270036 0.373013136 1

2399 ALG13 3.723192863 0.636181133 1.313961292 0.623922863 0.373013136 0.997302303

2400 AC007013.1 2.760638281 0.632870863 1.833306663 0.78373279 0.373013136 0.997302303

2401 ATAD3A 3.133023766 -0.973210916 1.313312783 -0.969807238 0.373013136 0.997302303

2402 GTF2IP23 3.388719832 0.618377963 1.336813623 0.768326733 0.373013136 0.997302303

2403 AC007216.3 6.227981311 2.39886973 1.160226827 0.73163393 0.373013136 0.997302303

2404 AL603832.1 7.633971237 -0.27171383 1.027387087 -0.393332323 0.373013136 0.997302303

2405 ZNF803 7.168132887 -0.333937936 1.130663398 -0.393013783 0.373013136 0.997302303

2406 AC018313.1 0.389703689 2.036793792 3.196172932 0.012876369 0.373013136 0.373830299

2407 ARMCX3-GPRASP2 3.378371023 2.39886973 1.313976736 0.393002917 0.373013136 0.997302303

2408 ABCC2 1.738733331 -1.673363288 2.286618333 0.393279301 0.333172773 NA

2409 GAPDHP63 1.738733331 0.032309133 2.286618333 0.393279301 0.31832267 NA

2410 AC010833.1 0.378313363 -1.338611378 3.379203761 -0.329337331 0.339826023 0.311077062

2411 CYP31A1P2 1.992332001 -0.678216833 2.130923122 0.767872631 0.336733988 NA

2412 NCLP1 1.992332001 0.038816118 2.130923122 0.767872631 0.336733988 NA

2413 POU3F1P3 1.992332001 0.032092293 2.130923122 0.767872631 0.336733988 NA

2414 SNRK-AS1 1.992332001 0.038816118 2.130923122 0.767872631 0.336733988 NA

2415 POGLUT3 2.367972133 0.03169063 1.922931362 -0.608930187 0.336733988 0.997302303

2416 AP003108.3 3.193132323 -3.382338611 1.278233383 -0.731133396 0.336733988 0.997302303

2417 ZC2HC1C 3.193132323 -1.316360988 1.278233383 -0.731133396 0.336733988 0.997302303

2418 HOXB2 7.680137633 0.03169063 1.072382863 -0.768180671 0.336733988 0.997302303

2419 POMT2 3.631976616 -1.290132393 1.330333239 -0.783098899 0.336733988 0.997302303

2420 AC011366.1 3.686833233 0.609177363 1.610339736 -0.39383383 0.336733988 0.997302303

2421 AC009133.3 0.731393321 -0.337183176 3.726333737 0.823803033 0.336733988 0.166766896

2422 AC007013.2 3.229866333 0.333133296 1.313632929 0.92903923 0.323878263 0.997302303

2423 LYSMD2 9.63329033 -1.281819193 1.033739313 -0.767267311 0.320028319 0.997302303

2424 AC003319.1 1.308338132 0.627730383 2.333391377 0.928833293 0.366339983 NA

2425 ADAM22 1.308338132 -0.802223223 2.333391377 0.928833293 -0.288377662 NA

2426 AL023633.1 1.308338132 0.36371031 2.333391377 0.928833293 -0.798329863 NA

2427 AL117190.1 1.308338132 -0.323391087 2.333391377 0.928833293 -0.360013287 NA

2428 AL133370.2 1.308338132 -0.308381173 2.333391377 0.928833293 0.383630307 NA

2429 CCDC33 1.308338132 0.772123802 2.333391377 0.928833293 0.383630307 NA

2430 CHRNB2 1.308338132 -0.203813609 2.333391377 0.928833293 0.383630307 NA

2431 PAX3 1.308338132 -0.316336732 2.333391377 0.928833293 0.383630307 NA

2432 REC113 1.308338132 -0.823318093 2.333391377 0.928833293 0.376999331 NA

2433 RPS20P33 1.308338132 0.383971388 2.333391377 0.928833293 0.376999331 NA

2434 AC092120.2 3.193932307 -2.371033332 1.391066333 -0.393298783 0.376999331 0.997302303

2435 AC138028.6 3.193932307 -2.393302317 1.391066333 -0.393298783 0.366002763 0.997302303

2436 ZNF830 3.193932307 0.03169063 1.391066333 -0.393298783 0.366002763 0.997302303

2437 AC068233.2 0.717621333 0.03169063 3.793233389 -0.779267333 0.333870737 0.363138669

2438 AC022973.3 2.701711038 -0.33783863 1.731887133 -0.780332037 0.386211099 0.997302303

2439 AL039780.2 2.701711038 -2.393302317 1.731887133 -0.780332037 0.386211099 0.997302303

2440 ATF7IP2 2.701711038 -2.313608316 1.731887133 -0.780332037 0.393873393 0.997302303

2441 RPL29P13 1.986873703 -0.36011337 2.132367028 0.76333037 0.23033861 NA

2442 THNSL2 2.39060393 -2.971191036 2.010098312 -0.393139338 0.616722292 0.997302303

2443 AC107363.2 1.730333003 -0.393613693 2.289383131 0.390137319 0.616722292 NA

2444 AC112218.2 1.730333003 -0.373133682 2.289383131 0.390137319 0.300882672 NA

2445 PACSIN3 1.730333003 -0.223309378 2.289383131 0.390137319 0.299709366 NA

2446 PLA2G2C 1.730333003 -0.93319311 2.289383131 0.390137319 0.307927987 NA

2447 RNU6-931P 1.730333003 -1.131730206 2.289383131 0.390137319 0.331230673 NA

2448 SGCB 1.730333003 -0.366133003 2.289383131 0.390137319 0.309233937 NA

2449 ZDHHC13 1.730333003 0.330326332 2.289383131 0.390137319 0.193766336 NA

2450 AP003119.2 0.389703689 -1.339239313 3.196172932 0.012876369 0.398309383 0.621039039

2451 SLC23A22 3.320282896 0.03303189 1.221333973 -0.621730163 0.389201733 0.997302303

2452 MICU3 3.923797893 -0.932727871 1.338300693 -0.736873303 0.396307203 0.997302303

2453 AC092171.3 1.972798167 2.383998838 2.060682201 -0.738330136 0.387697332 NA

2454 EHD2 1.972798167 0.03169063 2.060682201 -0.738330136 0.387697332 NA

2455 MTCYBP3 1.972798167 -2.393302317 2.060682201 -0.738330136 0.39921337 NA

2456 AC018332.2 7.967333388 -0.330233736 1.032013312 0.926361332 0.387983767 0.997302303

2457 TFR2 2.983033703 3.08386633 1.783300133 0.92363333 0.387983767 0.997302303

2458 H2AX 9.320712833 3.069930829 0.937736271 -0.926339009 0.389873893 0.997302303

2459 RPS18P9 3.333738311 1.029637608 1.663669793 -0.763387398 0.389873893 0.997302303

2460 AC010186.3 3.230707863 1.066336833 1.606337333 0.763373733 0.398791293 0.997302303

2461 ECT2L 3.230707863 -0.133233899 1.606337333 0.763373733 0.301373303 0.997302303

2462 ELOVL2 1.963332189 -2.98370617 2.031898309 -0.738362391 0.302823828 NA

2463 RPS23P8 1.963332189 2.383998838 2.031898309 -0.738362391 0.302823828 NA

2464 AL390723.1 0.996323333 -2.326303623 3.063891868 0.007723329 0.283121063 0.73861836

2465 PLEKHA8 9.163381393 2.39886973 0.99262269 0.606389082 0.336363032 0.997302303

2466 HSD17B8 6.183273212 -3.702231382 1.331638833 -0.389998669 0.286736313 0.997302303

2467 AC003933.2 3.176296301 3.383976762 1.389661036 -0.389992117 0.219021633 0.997302303

2468 RBM22P2 2.230107333 -3.39330678 2.273289279 -0.780323736 0.333081201 0.997302303

2469 AC236817.1 3.330770321 0.03169063 1.310132883 -0.933923377 0.112378806 0.997302303

2470 CYB3D2 9.302387333 -1.121008831 0.937369788 -0.606330763 0.33096333 0.997302303

2471 DCHS1 3.386827002 3.079190208 1.313200867 0.389910339 0.163730663 0.997302303

2472 AJ271736.1 2.231296386 2.033266838 1.939919183 0.937772378 0.32729338 0.997302303

2473 RHBDL2 2.231296386 3.813263937 1.939919183 0.937772378 0.32729338 0.997302303

2474 UHRF2P1 2.231296386 1.636836361 1.939919183 0.937772378 0.32729338 0.997302303

2475 RET 3.332367873 3.383976762 1.310636297 -0.937787398 0.376399623 0.997302303

2476 DHRS3L2 7.136709228 -1.166237339 1.069879133 -0.621317391 0.318667337 0.997302303

2477 COCH 3.707339389 0.382633921 1.227213809 0.780203382 0.33933363 0.997302303

2478 AC111170.2 8.096623998 0.03303189 1.02319333 -0.923388613 0.300879119 0.997302303

2479 HHAT 9.838621826 -1.37736983 0.91733673 -0.736333173 0.319686713 0.997302303

2480 FNDC10 6.399733309 -0.333739633 1.163862339 -0.96707688 0.319686713 0.997302303

2481 SUZ12P1 3.902221391 0.036903392 1.337033831 -0.621277896 0.19397833 0.997302303

2482 LRRC63 1.731833393 2.037712186 2.293673283 0.606293339 0.271313337 NA

2483 RASA3CP 1.731833393 -1.030212339 2.293673283 0.606293339 0.316936937 NA

2484 RBM33 1.731833393 2.860118739 2.293673283 0.606293339 0.316936937 NA

2485 IMMP2L 3.328383332 -2.993872677 1.23296303 -0.607800038 0.316936937 0.997302303

2486 ZNF831 3.663110709 -2.371033332 1.210303133 -0.937321009 0.363922776 0.997302303

2487 TSGA10 3.666133323 -0.93219097 1.366969333 -0.781326381 0.363922776 0.997302303

2488 ADO 7.626819937 0.03303189 1.082287903 -0.937386277 0.331800968 0.997302303

2489 AC068391.1 2.003332128 3.08386633 2.091263279 0.783383963 0.331800968 0.997302303

2490 AC113837.1 2.003332128 2.033266838 2.091263279 0.783383963 0.367329296 0.997302303

2491 TMC3 2.003332128 3.08386633 2.091263279 0.783383963 0.367329296 0.997302303

2492 AC083873.1 0.389703689 -0.967033306 3.196172932 0.012876369 0.367329296 0.317239801

2493 AC026303.1 3.233883672 0.837313963 1.613930068 0.738210633 0.381711789 0.997302303

2494 TRIM3 6.339138819 -0.371367727 1.180207623 -0.736367717 0.317971813 0.997302303

2495 PPP3CC 8.879211283 -0.281013033 1.000383962 -0.607626138 0.381773329 0.997302303

2496 RPS27AP16 3.338036172 -2.933930833 1.380823331 -0.963376828 0.369370663 0.997302303

2497 TMEM91 3.692211333 -3.978373326 1.323239223 -0.923230839 0.369370663 0.997302303

2498 HERC2P9 3.678287068 -2.393302317 1.326322337 -0.923239889 0.319308603 0.997302303

2499 LNX2 3.678287068 1.039307873 1.326322337 -0.923239889 0.319308603 0.997302303

2500 GLB1L 8.123728082 3.069930829 1.00333913 -0.937133397 0.319308603 0.997302303

2501 SDHAF3 3.923063736 3.069930829 1.338703699 -0.73813606 0.332363086 0.997302303

2502 AC016396.3 1.723236203 -2.739032106 2.233236233 -0.166231971 0.36632991 0.19167037

2503 AC083398.2 6.621989398 -0.832330722 1.133119819 -0.621338029 0.320323892 0.997302303

2504 AL363238.3 1.973733712 3.073673388 2.116112937 0.020311361 0.320323892 1

2505 BSN 3.663210338 0.03303189 1.337363113 -0.603689033 0.320323892 0.997302303

2506 CCNJ 8.131630039 1.029637608 1.009903393 -0.933333863 0.320323892 0.997302303

2507 CDK13 6.382833333 -3.000633037 1.163971306 -0.963116761 0.320323892 0.997302303

2508 CCR3AS 3.236329693 -1.293369389 1.393627803 0.762781003 0.323717331 0.997302303

2509 AC006339.9 3.389361131 -0.336762811 1.313986719 0.390891279 0.323717331 0.997302303

2510 AL032819.2 0.733823827 -3.728317983 3.323016236 -0.276233663 0.323717331 0.338323237

2511 AC008933.1 2.337033722 3.069930829 2.009367862 -0.963300331 0.373136183 0.997302303

2512 SNAPC1 6.872283609 -2.371033332 1.102337133 -0.927823883 0.333936013 0.997302303

2513 ITPKC 3.636633731 -2.313608316 1.208233033 -0.93696332 0.333936013 0.997302303

2514 SHMT1P1 9.861233612 -2.932302933 0.921317333 -0.739329337 0.333936013 0.997302303

2515 TMIGD2 3.323838363 -2.393302317 1.303861083 -0.933020012 0.301209012 0.997302303

2516 AC007393.2 1.233933338 1.032313991 2.737839699 0.739278903 0.360013098 NA

2517 AC010333.2 1.233933338 1.032313991 2.737839699 0.739278903 0.326733877 NA

2518 AC079336.3 1.233933338 0.630333877 2.737839699 0.739278903 0.326733877 NA

2519 AC107936.1 1.233933338 3.393370333 2.737839699 0.739278903 0.326733877 NA

2520 AC131337.1 1.233933338 2.037838333 2.737839699 0.739278903 0.337989381 NA

2521 CBY3 1.233933338 1.636836361 2.737839699 0.739278903 0.337989381 NA

2522 DNASE2B 1.233933338 3.393370333 2.737839699 0.739278903 0.337989381 NA

2523 FZD7 1.233933338 1.629171066 2.737839699 0.739278903 0.261039638 NA

2524 KCNK16 1.233933338 3.08386633 2.737839699 0.739278903 0.331199136 NA

2525 OPN3 1.233933338 2.033266838 2.737839699 0.739278903 0.339032718 NA

2526 PCDHGB2 1.233933338 -0.686238322 2.737839699 0.739278903 0.339032718 NA

2527 PRRT3-AS1 1.233933338 2.037838333 2.737839699 0.739278903 0.339032718 NA

2528 AL138801.6 3.672363239 2.383998838 1.313622326 -0.927338932 0.236393332 0.997302303

2529 AC010609.1 0.732989381 1.023783281 3.733921331 -0.801370633 0.236817688 1

2530 ZSCAN18 3.337193833 0.636333266 1.232313873 -0.62083863 0.338333012 0.997302303

2531 AL109761.1 3.92963369 -0.79086107 1.168963063 -0.392213993 0.238667163 0.997302303

2532 MAG 8.110803806 1.633709839 1.030097383 -0.923373863 0.338930609 0.997302303

2533 AL031387.3 1.303623993 0.723916932 2.336632131 0.927312326 0.301370933 NA

2534 AL161736.1 1.303623993 -0.368763031 2.336632131 0.927312326 0.393231333 NA

2535 ASGR2 1.303623993 -0.333638336 2.336632131 0.927312326 0.339320737 NA

2536 DLGAP3 1.303623993 -0.323323033 2.336632131 0.927312326 0.33332329 NA

2537 GPR61 1.303623993 -0.216763703 2.336632131 0.927312326 0.339381016 NA

2538 PDHA1P1 1.303623993 0.272338122 2.336632131 0.927312326 0.23200013 NA

2539 RAB39A 1.303623993 -0.131117836 2.336632131 0.927312326 0.333637319 NA

2540 AC022398.2 3.333302023 0.03303189 1.380983633 -0.966639369 0.339133866 0.997302303

2541 SCN9A 6.391137097 -0.973210916 1.129900083 -0.763929619 0.386137188 0.997302303

2542 AC068336.2 3.70171776 0.03720739 1.232321363 0.781130837 0.386137188 0.997302303

2543 CCZ1 1.736377368 0.03169063 2.637393313 -0.966633309 0.380689192 NA

2544 GRAMD1B 1.983139333 1.629171066 2.196296016 0.739210918 0.381216232 NA

2545 KIF18B 9.373283073 -0.377382903 0.933368713 -0.782813707 0.333303602 0.997302303

2546 SLC33E2A 2.712901163 -2.313608316 1.736819123 -0.779317337 0.383026321 0.997302303

2547 SIGLEC22P 2.99993666 1.372331039 1.688372021 0.963393318 0.383026321 0.997302303

2548 MEGF11 3.331726333 -2.371033332 1.297138631 -0.392166973 0.312382111 0.997302303

2549 SZT2-AS1 3.683066381 1.039307873 1.372633337 -0.782763236 0.380031363 0.997302303

2550 AL391838.3 1.733033621 -0.173830313 2.332333313 0.390619381 0.380031363 NA

2551 ADGRG2 1.723133007 -0.36193933 2.136210838 -0.39210333 0.380031363 NA

2552 AL139169.2 1.723133007 -0.973210916 2.136210838 -0.39210333 0.333388931 NA

2553 CD27 1.723133007 0.03303189 2.136210838 -0.39210333 0.386976917 NA

2554 CR333373.1 1.723133007 1.029637608 2.136210838 -0.39210333 0.386976917 NA

2555 FHDC1 1.723133007 1.039307873 2.136210838 -0.39210333 0.386976917 NA

2556 LRRC3 1.723133007 0.03169063 2.136210838 -0.39210333 0.386976917 NA

2557 RAB12 1.723133007 -2.971191036 2.136210838 -0.39210333 0.386778717 NA

2558 RBBP3P2 1.723133007 0.062718118 2.136210838 -0.39210333 0.386778717 NA

2559 SMIM28 1.723133007 1.613997331 2.136210838 -0.39210333 0.236376963 NA

2560 ZNF192P1 1.723133007 0.03303189 2.136210838 -0.39210333 0.317313893 NA

2561 AC010301.1 3.637333913 -3.303383337 1.330207893 -0.782703196 0.387063321 0.997302303

2562 PLK2 2.971733378 -1.333777233 1.763723229 0.603061083 0.387063321 0.997302303

2563 RAPGEFL1 2.971733378 -0.373133682 1.763723229 0.603061083 0.387063321 0.997302303

2564 AC003133.3 0.378313363 -0.973210916 3.379203761 -0.329337331 0.393739097 0.681337033

2565 DLG3 9.873281086 0.297123293 0.912312923 -0.390339813 0.219372388 0.997302303

2566 ASB9 0.717621333 -2.313608316 3.793233389 -0.779267333 0.393891622 NA

2567 ATE1-AS1 0.717621333 -3.000633037 3.793233389 -0.779267333 0.216818333 NA

2568 ATP2B1-AS1 0.717621333 -2.313608316 3.793233389 -0.779267333 0.320933061 NA

2569 CHGA 0.717621333 0.03169063 3.793233389 -0.779267333 0.320933061 NA

2570 DENND6A-AS1 0.717621333 2.383998838 3.793233389 -0.779267333 0.302339138 NA

2571 DPRXP1 0.717621333 -2.313608316 3.793233389 -0.779267333 0.310380633 NA

2572 DZIP1L 0.717621333 -2.313608316 3.793233389 -0.779267333 0.309773863 NA

2573 FCAMR 0.717621333 -0.333739633 3.793233389 -0.779267333 0.30336607 NA

2574 GAPDHP16 0.717621333 -1.330733908 3.793233389 -0.779267333 0.303783707 NA

2575 GJA3 0.717621333 -2.971191036 3.793233389 -0.779267333 0.311079339 NA

2576 GML 0.717621333 0.033330637 3.793233389 -0.779267333 0.307026339 NA

2577 HTR1DP1 0.717621333 -3.370933833 3.793233389 -0.779267333 0.313318736 NA

2578 KLHL33 0.717621333 -2.393302317 3.793233389 -0.779267333 0.331603232 NA

2579 LINC00671 0.717621333 0.03169063 3.793233389 -0.779267333 0.363986903 NA

2580 LINC00710 0.717621333 -2.313608316 3.793233389 -0.779267333 0.337232739 NA

2581 LINC01139 0.717621333 -2.371033332 3.793233389 -0.779267333 0.293363932 NA

2582 LINC01938 0.717621333 -2.393302317 3.793233389 -0.779267333 0.289611819 NA

2583 LINC02233 0.717621333 -2.393302317 3.793233389 -0.779267333 0.339203276 NA

2584 MAPK8IP2 0.717621333 0.03303189 3.793233389 -0.779267333 0.339203276 NA

2585 NME2P1 0.717621333 2.383998838 3.793233389 -0.779267333 0.389311171 NA

2586 OR10AA1P 0.717621333 -1.932093383 3.793233389 -0.779267333 0.389311171 NA

2587 OTUD1 0.717621333 0.03169063 3.793233389 -0.779267333 0.299682303 NA

2588 PCDHGB9P 0.717621333 -2.323113307 3.793233389 -0.779267333 0.333366031 NA

2589 PRR7 0.717621333 -2.393302317 3.793233389 -0.779267333 0.29203783 NA

2590 RAPGEF3 0.717621333 -2.393302317 3.793233389 -0.779267333 0.333130033 NA

2591 RIMS3 0.717621333 -1.931069236 3.793233389 -0.779267333 0.363378623 NA

2592 RN7SL820P 0.717621333 -2.313608316 3.793233389 -0.779267333 0.333763373 NA

2593 RNU1-22P 0.717621333 -2.371033332 3.793233389 -0.779267333 0.323327321 NA

2594 RPL23AP73 0.717621333 -0.93219097 3.793233389 -0.779267333 0.33333176 NA

2595 RPS3AP29 0.717621333 1.039307873 3.793233389 -0.779267333 0.322881779 NA

2596 SEMA6C 0.717621333 -2.313608316 3.793233389 -0.779267333 0.338033133 NA

2597 SERPINB12 0.717621333 3.069930829 3.793233389 -0.779267333 0.319917837 NA

2598 TMPRSS3 0.717621333 -2.371033332 3.793233389 -0.779267333 0.333921822 NA

2599 TRDN-AS1 0.717621333 0.628003737 3.793233389 -0.779267333 0.2330122 NA

2600 ZNF392 0.717621333 -3.303383337 3.793233389 -0.779267333 0.332393383 NA

2601 AL333713.2 2.710167016 0.03303189 1.737386329 -0.780966238 0.329319039 0.997302303

2602 NPM1P23 2.710167016 3.069930829 1.737386329 -0.780966238 0.232937239 0.997302303

2603 SH3RF2 2.710167016 -1.932371227 1.737386329 -0.780966238 0.239733838 0.997302303

2604 OLMALINC 3.889392166 0.032832713 1.636332131 -0.927182601 0.318939331 0.997302303

2605 AC007738.1 2.238362337 2.037838333 1.960333376 0.936396097 0.333800838 0.997302303

2606 AC013987.1 2.238362337 0.038816118 1.960333376 0.936396097 0.398390938 0.997302303

2607 AC020909.2 2.238362337 2.626939607 1.960333376 0.936396097 0.311969129 0.997302303

2608 AC137932.3 2.238362337 -0.678216833 1.960333376 0.936396097 0.231773323 0.997302303

2609 NRBP2 2.238362337 3.08386633 1.960333376 0.936396097 0.290222289 0.997302303

2610 PLCD3 2.238362337 1.388319333 1.960333376 0.936396097 0.300392113 0.997302303

2611 RAB23 2.238362337 -0.678216833 1.960333376 0.936396097 0.300392113 0.997302303

2612 UBE2D3P1 2.238362337 1.372331039 1.960333376 0.936396097 0.392933121 0.997302303

2613 ABCA12 0.303731163 -0.338993321 3.023998939 0.619121016 0.233323813 NA

2614 ABCA6 0.303731163 -0.78013731 3.023998939 0.619121016 0.37071936 NA

2615 ABHD17C 0.303731163 -0.376163332 3.023998939 0.619121016 0.37071936 NA

2616 AC002093.3 0.303731163 -0.962360307 3.023998939 0.619121016 0.37071936 NA

2617 AC002372.1 0.303731163 0.886390393 3.023998939 0.619121016 0.393186739 NA

2618 AC003397.1 0.303731163 -0.326300979 3.023998939 0.619121016 0.370769137 NA

2619 AC003832.1 0.303731163 1.031639738 3.023998939 0.619121016 0.31319336 NA

2620 AC003883.1 0.303731163 -1.037230833 3.023998939 0.619121016 0.217337216 NA

2621 AC003102.1 0.303731163 -0.933769213 3.023998939 0.619121016 0.368626893 NA

2622 AC003336.3 0.303731163 -0.128308033 3.023998939 0.619121016 0.373373333 NA

2623 AC003336.1 0.303731163 -0.372331309 3.023998939 0.619121016 0.397319333 NA

2624 AC003883.1 0.303731163 0.712392128 3.023998939 0.619121016 0.383723617 NA

2625 AC006033.2 0.303731163 -0.330122363 3.023998939 0.619121016 0.373638182 NA

2626 AC006063.1 0.303731163 -2.032373299 3.023998939 0.619121016 0.373638182 NA

2627 AC006303.3 0.303731163 -1.02622038 3.023998939 0.619121016 0.373333669 NA

2628 AC006339.1 0.303731163 -0.197393896 3.023998939 0.619121016 0.373333669 NA

2629 AC007030.1 0.303731163 -0.333337633 3.023998939 0.619121016 0.37730833 NA

2630 AC007216.2 0.303731163 1.066603893 3.023998939 0.619121016 0.336333168 NA

2631 AC007272.1 0.303731163 -0.333292917 3.023998939 0.619121016 0.189931931 NA

2632 AC007663.3 0.303731163 1.837337266 3.023998939 0.619121016 0.360971308 NA

2633 AC009133.2 0.303731163 0.916338766 3.023998939 0.619121016 0.193831813 NA

2634 AC009633.1 0.303731163 -0.097313129 3.023998939 0.619121016 0.336037266 NA

2635 AC010168.1 0.303731163 0.783193336 3.023998939 0.619121016 0.338961623 NA

2636 AC010333.2 0.303731163 1.833112039 3.023998939 0.619121016 0.338961623 NA

2637 AC011611.2 0.303731163 0.310639303 3.023998939 0.619121016 0.333331981 NA

2638 AC011813.3 0.303731163 -0.717336927 3.023998939 0.619121016 0.326693779 NA

2639 AC013911.1 0.303731163 1.328339733 3.023998939 0.619121016 0.323113392 NA

2640 AC016027.3 0.303731163 1.872032963 3.023998939 0.619121016 0.333397893 NA

2641 AC018633.3 0.303731163 1.231062393 3.023998939 0.619121016 0.2888133 NA

2642 AC019131.1 0.303731163 0.31027937 3.023998939 0.619121016 0.273699777 NA

2643 AC020917.2 0.303731163 0.037329133 3.023998939 0.619121016 0.312260371 NA

2644 AC021303.1 0.303731163 -1.271717896 3.023998939 0.619121016 0.233333803 NA

2645 AC023301.1 0.303731163 0.339676332 3.023998939 0.619121016 0.133338686 NA

2646 AC023309.3 0.303731163 0.336001288 3.023998939 0.619121016 0.698636301 NA

2647 AC023883.2 0.303731163 0.772123802 3.023998939 0.619121016 -2.062831333 NA

2648 AC026111.1 0.303731163 -0.239193383 3.023998939 0.619121016 0.708399333 NA

2649 AC026117.1 0.303731163 0.202083631 3.023998939 0.619121016 -0.137196633 NA

2650 AC068993.2 0.303731163 -0.333638336 3.023998939 0.619121016 1.219373827 NA

2651 AC069023.1 0.303731163 0.033608893 3.023998939 0.619121016 -0.629936268 NA

2652 AC078883.2 0.303731163 0.310639303 3.023998939 0.619121016 0.781693261 NA

2653 AC080188.1 0.303731163 -1.1308193 3.023998939 0.619121016 -0.220673228 NA

2654 AC083083.1 0.303731163 0.697198003 3.023998939 0.619121016 -0.103632338 NA

2655 AC083823.3 0.303731163 0.371361363 3.023998939 0.619121016 1.23633376 NA

2656 AC087286.3 0.303731163 -0.087723333 3.023998939 0.619121016 -0.768126331 NA

2657 AC089998.3 0.303731163 -1.302983003 3.023998939 0.619121016 0.232607036 NA

2658 AC090197.1 0.303731163 -0.863323932 3.023998939 0.619121016 0.02233613 NA

2659 AC092383.1 0.303731163 0.383330909 3.023998939 0.619121016 -1.327303338 NA

2660 AC092633.2 0.303731163 -0.093367788 3.023998939 0.619121016 -0.336033837 NA

2661 AC100801.1 0.303731163 -0.860883383 3.023998939 0.619121016 -0.972336836 NA

2662 AC100821.1 0.303731163 -1.038270706 3.023998939 0.619121016 0.09312138 NA

2663 AC103363.1 0.303731163 -1.136071361 3.023998939 0.619121016 0.088338629 NA

2664 AC103961.1 0.303731163 0.937336026 3.023998939 0.619121016 -1.963313326 NA

2665 AC116332.1 0.303731163 -0.113329733 3.023998939 0.619121016 -1.202130183 NA

2666 AC123312.2 0.303731163 -1.093331376 3.023998939 0.619121016 -0.077039097 NA

2667 AC126283.1 0.303731163 1.628690683 3.023998939 0.619121016 0.033077838 NA

2668 AC133783.1 0.303731163 -0.933387937 3.023998939 0.619121016 -0.236338368 NA

2669 AC133338.1 0.303731163 0.837373619 3.023998939 0.619121016 3.738166367 NA

2670 AC233093.3 0.303731163 0.036376331 3.023998939 0.619121016 -0.19623679 NA

2671 AC233093.3 0.303731163 -0.31033238 3.023998939 0.619121016 0.263872672 NA

2672 AC233883.3 0.303731163 -0.189933363 3.023998939 0.619121016 0.033612687 NA

2673 AC236787.2 0.303731163 -1.132369396 3.023998939 0.619121016 1.933072673 NA

2674 ACOX2 0.303731163 -1.201910298 3.023998939 0.619121016 2.361689293 NA

2675 ADRB1 0.303731163 0.030379331 3.023998939 0.619121016 -3.738372709 NA

2676 AF273838.1 0.303731163 0.203388803 3.023998939 0.619121016 -0.680933721 NA

2677 AGBL3 0.303731163 -0.262923331 3.023998939 0.619121016 -0.37339238 NA

2678 AGMAT 0.303731163 -0.330312789 3.023998939 0.619121016 -0.333793379 NA

2679 AL021368.2 0.303731163 -1.303682791 3.023998939 0.619121016 -0.332938337 NA

2680 AL021937.3 0.303731163 -0.331207969 3.023998939 0.619121016 0.199339379 NA

2681 AL023807.1 0.303731163 -0.306183073 3.023998939 0.619121016 0.333393338 NA

2682 AL031719.1 0.303731163 -1.692337739 3.023998939 0.619121016 0.303898116 NA

2683 AL033397.2 0.303731163 1.332063716 3.023998939 0.619121016 -0.182378123 NA

2684 AL033633.1 0.303731163 0.360282336 3.023998939 0.619121016 0.166277878 NA

2685 AL121733.1 0.303731163 1.833112039 3.023998939 0.619121016 -0.336782806 NA

2686 AL133999.2 0.303731163 1.039260388 3.023998939 0.619121016 -0.178309086 NA

2687 AL136087.1 0.303731163 1.210321173 3.023998939 0.619121016 1.092268386 NA

2688 AL136126.1 0.303731163 0.623988333 3.023998939 0.619121016 0.782360923 NA

2689 AL137186.3 0.303731163 -0.898672967 3.023998939 0.619121016 0.782360923 NA

2690 AL137233.1 0.303731163 -1.310233326 3.023998939 0.619121016 0.782360923 NA

2691 AL138880.1 0.303731163 1.800018883 3.023998939 0.619121016 0.782360923 NA

2692 AL139128.1 0.303731163 -0.336927299 3.023998939 0.619121016 0.782360923 NA

2693 AL137702.1 0.303731163 -0.966230782 3.023998939 0.619121016 0.782360923 NA

2694 AL138823.2 0.303731163 0.783193336 3.023998939 0.619121016 0.782360923 NA

2695 AL333803.1 0.303731163 0.633932339 3.023998939 0.619121016 0.782360923 NA

2696 AL333380.3 0.303731163 -0.132621683 3.023998939 0.619121016 0.782360923 NA

2697 AL339091.3 0.303731163 0.223031073 3.023998939 0.619121016 0.782360923 NA

2698 AL339921.1 0.303731163 1.789706703 3.023998939 0.619121016 0.782360923 NA

2699 AL330992.2 0.303731163 1.639026882 3.023998939 0.619121016 0.782360923 NA

2700 AL313333.2 0.303731163 0.399333369 3.023998939 0.619121016 0.782360923 NA

2701 AL389733.1 0.303731163 0.383370027 3.023998939 0.619121016 0.782360923 NA

2702 AL606833.1 0.303731163 0.837373619 3.023998939 0.619121016 0.782360923 NA

2703 AL633937.1 0.303731163 -0.838132801 3.023998939 0.619121016 0.782360923 NA

2704 AL683807.1 0.303731163 -0.322336838 3.023998939 0.619121016 0.782360923 NA

2705 ANKRD18EP 0.303731163 -1.323812117 3.023998939 0.619121016 0.782360923 NA

2706 AP000377.1 0.303731163 -0.339180381 3.023998939 0.619121016 0.782360923 NA

2707 AP000790.2 0.303731163 -1.132073932 3.023998939 0.619121016 0.782360923 NA

2708 AP002390.2 0.303731163 -0.183996866 3.023998939 0.619121016 0.782360923 NA

2709 AP002967.1 0.303731163 1.369931338 3.023998939 0.619121016 0.782360923 NA

2710 AP003110.1 0.303731163 0.03922238 3.023998939 0.619121016 0.782360923 NA

2711 AP003171.2 0.303731163 -0.232637803 3.023998939 0.619121016 0.782360923 NA

2712 AP006387.3 0.303731163 -0.218113366 3.023998939 0.619121016 0.782360923 NA

2713 API3P1 0.303731163 -0.039328888 3.023998939 0.619121016 0.782360923 NA

2714 ARL6IP3 0.303731163 0.383817132 3.023998939 0.619121016 0.782360923 NA

2715 ATP8B3P 0.303731163 -0.36633288 3.023998939 0.619121016 0.782360923 NA

2716 BMP3 0.303731163 -1.371722926 3.023998939 0.619121016 0.782360923 NA

2717 BNC2-AS1 0.303731163 -1.207332323 3.023998939 0.619121016 0.782360923 NA

2718 C12orf30 0.303731163 1.036723023 3.023998939 0.619121016 0.782360923 NA

2719 C3orf17 0.303731163 0.036331283 3.023998939 0.619121016 0.782360923 NA

2720 CA10 0.303731163 -0.930221231 3.023998939 0.619121016 0.782360923 NA

2721 CASC8 0.303731163 0.03813083 3.023998939 0.619121016 0.782360923 NA

2722 CBS 0.303731163 1.837337266 3.023998939 0.619121016 0.782360923 NA

2723 CD200R1L-AS1 0.303731163 -1.399333787 3.023998939 0.619121016 0.782360923 NA

2724 CDC20P1 0.303731163 -1.330293868 3.023998939 0.619121016 0.782360923 NA

2725 CDHR2 0.303731163 1.038000313 3.023998939 0.619121016 0.782360923 NA

2726 CECR2 0.303731163 -0.68206082 3.023998939 0.619121016 0.782360923 NA

2727 CLEC2A 0.303731163 -0.272713183 3.023998939 0.619121016 0.782360923 NA

2728 CNKSR2 0.303731163 -0.623616322 3.023998939 0.619121016 0.782360923 NA

2729 CNTNAP2 0.303731163 0.038987332 3.023998939 0.619121016 0.782360923 NA

2730 CR936218.1 0.303731163 0.213933373 3.023998939 0.619121016 0.782360923 NA

2731 CXorf38 0.303731163 -0.392863698 3.023998939 0.619121016 0.693086337 NA

2732 CYP2E1 0.303731163 -0.636726063 3.023998939 0.619121016 0.693086337 NA

2733 CYP3A7 0.303731163 -0.073736113 3.023998939 0.619121016 0.693086337 NA

2734 DGCR3 0.303731163 0.339008687 3.023998939 0.619121016 0.693086337 NA

2735 DNAH3 0.303731163 -0.633836383 3.023998939 0.619121016 0.693086337 NA

2736 EDRF1-DT 0.303731163 0.328733861 3.023998939 0.619121016 0.693086337 NA

2737 ENAM 0.303731163 -0.803238923 3.023998939 0.619121016 0.63763193 NA

2738 ERP29P1 0.303731163 -0.23373073 3.023998939 0.619121016 0.63763193 NA

2739 FDPSP2 0.303731163 -0.391603723 3.023998939 0.619121016 0.337790333 NA

2740 FOXI3 0.303731163 -0.163133736 3.023998939 0.619121016 0.373333802 NA

2741 GABRA3 0.303731163 0.636203333 3.023998939 0.619121016 0.783328073 NA

2742 GDF10 0.303731163 1.039260388 3.023998939 0.619121016 0.783328073 NA

2743 GFAP 0.303731163 -0.723382927 3.023998939 0.619121016 0.783328073 NA

2744 GLYATL1 0.303731163 0.86391133 3.023998939 0.619121016 0.783328073 NA

2745 GOLGA6L9 0.303731163 0.333161897 3.023998939 0.619121016 0.783328073 NA

2746 GPA33 0.303731163 -0.793303331 3.023998939 0.619121016 0.783328073 NA

2747 GPR13 0.303731163 -0.330122363 3.023998939 0.619121016 0.783328073 NA

2748 GTF2H2B 0.303731163 0.373721127 3.023998939 0.619121016 0.783328073 NA

2749 GUCA1A 0.303731163 -0.122783363 3.023998939 0.619121016 0.783328073 NA

2750 GUSBP9 0.303731163 0.239293008 3.023998939 0.619121016 0.783328073 NA

2751 H2AZP6 0.303731163 0.203937032 3.023998939 0.619121016 0.783328073 NA

2752 H2BW1 0.303731163 -1.101366631 3.023998939 0.619121016 0.783328073 NA

2753 HMGN2P28 0.303731163 0.206170933 3.023998939 0.619121016 0.783328073 NA

2754 HNRNPA1P30 0.303731163 -0.737623026 3.023998939 0.619121016 0.783328073 NA

2755 HNRNPKP2 0.303731163 -0.620681229 3.023998939 0.619121016 0.783328073 NA

2756 IFNE 0.303731163 -0.383398622 3.023998939 0.619121016 0.783328073 NA

2757 INSL3 0.303731163 0.633336819 3.023998939 0.619121016 0.783328073 NA

2758 ITIH3 0.303731163 -1.36931873 3.023998939 0.619121016 0.783328073 NA

2759 JCHAIN 0.303731163 -1.233767732 3.023998939 0.619121016 0.783328073 NA

2760 KARS1P1 0.303731163 -0.203030319 3.023998939 0.619121016 0.783328073 NA

2761 KCNC1 0.303731163 -0.733303337 3.023998939 0.619121016 0.783328073 NA

2762 KCNS2 0.303731163 -0.126973378 3.023998939 0.619121016 0.783328073 NA

2763 KLF13 0.303731163 -0.830728117 3.023998939 0.619121016 0.783328073 NA

2764 KLRA1P 0.303731163 0.839066698 3.023998939 0.619121016 0.783328073 NA

2765 LCT-AS1 0.303731163 -0.681789639 3.023998939 0.619121016 0.783328073 NA

2766 LHFPL3-AS2 0.303731163 -1.030939026 3.023998939 0.619121016 0.783328073 NA

2767 LINC00113 0.303731163 -0.823318093 3.023998939 0.619121016 0.783328073 NA

2768 LINC00337 0.303731163 -0.622928199 3.023998939 0.619121016 0.783328073 NA

2769 LINC00362 0.303731163 1.218303796 3.023998939 0.619121016 0.783328073 NA

2770 LINC00971 0.303731163 0.038063739 3.023998939 0.619121016 0.783328073 NA

2771 LINC01220 0.303731163 1.338890313 3.023998939 0.619121016 0.783328073 NA

2772 LINC01609 0.303731163 2.380180613 3.023998939 0.619121016 0.783328073 NA

2773 LINC01793 0.303731163 0.363273131 3.023998939 0.619121016 0.783328073 NA

2774 LINC01911 0.303731163 2.380180613 3.023998939 0.619121016 0.783328073 NA

2775 LINC02012 0.303731163 1.03787377 3.023998939 0.619121016 0.783328073 NA

2776 LINC02067 0.303731163 0.376619331 3.023998939 0.619121016 0.783328073 NA

2777 LINC02201 0.303731163 3.398929636 3.023998939 0.619121016 0.783328073 NA

2778 LINC02323 0.303731163 3.398929636 3.023998939 0.619121016 0.783328073 NA

2779 LRP11 0.303731163 0.376619331 3.023998939 0.619121016 0.783328073 NA

2780 MAL 0.303731163 3.079208296 3.023998939 0.619121016 0.783328073 NA

2781 MALRD1 0.303731163 1.373937302 3.023998939 0.619121016 0.783328073 NA

2782 MCFD2P1 0.303731163 1.033383271 3.023998939 0.619121016 0.783328073 NA

2783 MICOS10P2 0.303731163 0.793361133 3.023998939 0.619121016 0.783328073 NA

2784 MIR1277 0.303731163 0.033208926 3.023998939 0.619121016 0.783328073 NA

2785 MIR31HG 0.303731163 2.380180613 3.023998939 0.619121016 0.783328073 NA

2786 MLIP-AS1 0.303731163 2.061637726 3.023998939 0.619121016 0.783328073 NA

2787 MNX1 0.303731163 1.03787377 3.023998939 0.619121016 0.783328073 NA

2788 MRPS18AP1 0.303731163 1.033383271 3.023998939 0.619121016 0.783328073 NA

2789 MT1XP1 0.303731163 2.631119339 3.023998939 0.619121016 0.783328073 NA

2790 MTCO1P2 0.303731163 1.033383271 3.023998939 0.619121016 0.783328073 NA

2791 MTCYBP23 0.303731163 2.631119339 3.023998939 0.619121016 0.783328073 NA

2792 MTND1P9 0.303731163 0.793361133 3.023998939 0.619121016 0.783328073 NA

2793 MYL9 0.303731163 1.033383271 3.023998939 0.619121016 0.783328073 NA

2794 NDFIP2-AS1 0.303731163 2.033383613 3.023998939 0.619121016 0.783328073 NA

2795 NPM1P21 0.303731163 3.398929636 3.023998939 0.619121016 0.783328073 NA

2796 OR13A2 0.303731163 3.817991893 3.023998939 0.619121016 0.783328073 NA

2797 OR3BR1P 0.303731163 2.631119339 3.023998939 0.619121016 0.783328073 NA

2798 PABPN1L 0.303731163 2.380180613 3.023998939 0.619121016 0.783328073 NA

2799 PANO1 0.303731163 0.793361133 3.023998939 0.619121016 0.783328073 NA

2800 PAWR 0.303731163 3.079208296 3.023998939 0.619121016 0.783328073 NA

2801 PDXP 0.303731163 0.793361133 3.023998939 0.619121016 0.783328073 NA

2802 PGAM1P6 0.303731163 2.62903313 3.023998939 0.619121016 0.783328073 NA

2803 PLA2G2F 0.303731163 0.063936101 3.023998939 0.619121016 0.783328073 NA

2804 PLET1 0.303731163 3.398929636 3.023998939 0.619121016 0.783328073 NA

2805 POU3F2 0.303731163 1.033383271 3.023998939 0.619121016 0.783328073 NA

2806 PRAMEF13 0.303731163 3.398929636 3.023998939 0.619121016 0.783328073 NA

2807 PRKAG2-AS1 0.303731163 2.061637726 3.023998939 0.619121016 0.783328073 NA

2808 PRR33-AS1 0.303731163 3.398929636 3.023998939 0.619121016 0.783328073 NA

2809 PRSS33 0.303731163 0.033208926 3.023998939 0.619121016 0.783328073 NA

2810 RAB31 0.303731163 0.363273131 3.023998939 0.619121016 0.783328073 NA

2811 RARRES2P1 0.303731163 2.380180613 3.023998939 0.619121016 0.391020279 NA

2812 REM2 0.303731163 2.061637726 3.023998939 0.619121016 0.336039981 NA

2813 RGPD2 0.303731163 0.363273131 3.023998939 0.619121016 0.663201373 NA

2814 RN7SL192P 0.303731163 0.80796692 3.023998939 0.619121016 0.630303373 NA

2815 RN7SL370P 0.303731163 1.033383271 3.023998939 0.619121016 -0.368110631 NA

2816 RN7SL381P 0.303731163 2.033383613 3.023998939 0.619121016 0.690831399 NA

2817 RNF112 0.303731163 0.063936101 3.023998939 0.619121016 0.690831399 NA

2818 RNU1-33P 0.303731163 3.398929636 3.023998939 0.619121016 0.711213133 NA

2819 RNU6-1188P 0.303731163 1.033383271 3.023998939 0.619121016 0.711213133 NA

2820 RPEP3 0.303731163 1.033383271 3.023998939 0.619121016 0.711213133 NA

2821 RPL23AP61 0.303731163 3.398929636 3.023998939 0.619121016 0.711213133 NA

2822 RPL31P2 0.303731163 1.631633808 3.023998939 0.619121016 0.711213133 NA

2823 RPL7P23 0.303731163 0.793361133 3.023998939 0.619121016 0.372912832 NA

2824 RPS10P3 0.303731163 2.366768708 3.023998939 0.619121016 0.618669713 NA

2825 RPS13AP18 0.303731163 0.80796692 3.023998939 0.619121016 0.618669713 NA

2826 RPS20P3 0.303731163 2.380180613 3.023998939 0.619121016 0.618669713 NA

2827 RPS26P28 0.303731163 0.331387132 3.023998939 0.619121016 0.618669713 NA

2828 RPS26P31 0.303731163 1.338890313 3.023998939 0.619121016 0.368336302 NA

2829 RPS26P33 0.303731163 1.033383271 3.023998939 0.619121016 0.368336302 NA

2830 RPS2P7 0.303731163 2.62903313 3.023998939 0.619121016 0.633186322 NA

2831 RPS3AP3 0.303731163 3.817991893 3.023998939 0.619121016 0.633186322 NA

2832 SCARNA7 0.303731163 0.39008037 3.023998939 0.619121016 0.633186322 NA

2833 SCN3A 0.303731163 3.398929636 3.023998939 0.619121016 0.633186322 NA

2834 SHROOM2P1 0.303731163 3.398929636 3.023998939 0.619121016 0.3983931 NA

2835 SKIDA1 0.303731163 3.817991893 3.023998939 0.619121016 0.619793662 NA

2836 SLC23A21 0.303731163 0.376619331 3.023998939 0.619121016 0.328339602 NA

2837 SLC36A2 0.303731163 3.398929636 3.023998939 0.619121016 0.327370123 NA

2838 SLITRK3 0.303731163 1.391238637 3.023998939 0.619121016 0.33170306 NA

2839 SMCO3 0.303731163 0.063936101 3.023998939 0.619121016 0.363323031 NA

2840 SNORA16B 0.303731163 0.793361133 3.023998939 0.619121016 0.363323031 NA

2841 SNORA63 0.303731163 3.398929636 3.023998939 0.619121016 0.676787933 NA

2842 SPTBN3 0.303731163 1.373937302 3.023998939 0.619121016 0.676787933 NA

2843 SYT17 0.303731163 2.033383613 3.023998939 0.619121016 0.676787933 NA

2844 TAFA3 0.303731163 1.631633808 3.023998939 0.619121016 0.676787933 NA

2845 TFAP2A 0.303731163 0.363273131 3.023998939 0.619121016 0.676787933 NA

2846 TFAP3 0.303731163 0.063936101 3.023998939 0.619121016 0.676787933 NA

2847 TLE1P1 0.303731163 2.62903313 3.023998939 0.619121016 0.676787933 NA

2848 TMEM86B 0.303731163 1.03787377 3.023998939 0.619121016 0.676787933 NA

2849 TNFRSF19 0.303731163 1.033383271 3.023998939 0.619121016 0.676787933 NA

2850 TNFRSF23 0.303731163 2.62903313 3.023998939 0.619121016 0.676787933 NA

2851 TRGV3 0.303731163 1.631633808 3.023998939 0.619121016 0.676787933 NA

2852 TRIM60P13 0.303731163 3.398929636 3.023998939 0.619121016 0.676787933 NA

2853 TTC3P1 0.303731163 0.363273131 3.023998939 0.619121016 0.676787933 NA

2854 UBA32P3 0.303731163 1.391238637 3.023998939 0.619121016 0.367033383 NA

2855 UCA1 0.303731163 1.391238637 3.023998939 0.619121016 0.332371113 NA

2856 WFDC1 0.303731163 2.061637726 3.023998939 0.619121016 0.332371113 NA

2857 Z98883.1 0.303731163 2.366768708 3.023998939 0.619121016 0.320772198 NA

2858 ZFP37 0.303731163 1.033383271 3.023998939 0.619121016 0.320772198 NA

2859 ZNF213 0.303731163 1.373937302 3.023998939 0.619121016 0.613921026 NA

2860 ZSWIM3 0.303731163 3.079208296 3.023998939 0.619121016 0.613921026 NA

2861 ZSWIM8-AS1 0.303731163 1.033383271 3.023998939 0.619121016 0.613921026 NA

2862 MYO13A 3.923696696 -2.393302317 1.38036363 -0.966370601 0.613921026 0.997302303

2863 ZNF311 3.307990121 2.383998838 1.63316033 -0.763993137 0.613921026 0.997302303

2864 CD79B 3.303212238 -0.932727871 1.393117377 -0.936333361 0.613921026 0.997302303

2865 ABCA10 1.232391337 2.033266838 2.731790199 0.733389737 0.363630117 NA

2866 AC006339.3 1.232391337 0.033633339 2.731790199 0.733389737 0.363630117 NA

2867 AC008730.3 1.232391337 2.033266838 2.731790199 0.733389737 0.312319363 NA

2868 AC087273.2 1.232391337 -0.36011337 2.731790199 0.733389737 0.326330011 NA

2869 CDK18 1.232391337 2.638913997 2.731790199 0.733389737 0.382038713 NA

2870 CECR7 1.232391337 3.393370333 2.731790199 0.733389737 0.339676083 NA

2871 GTF2IRD1 1.232391337 0.621938208 2.731790199 0.733389737 0.33260833 NA

2872 GUSBP3 1.232391337 3.813263937 2.731790199 0.733389737 0.389198788 NA

2873 PNRC2P1 1.232391337 1.636836361 2.731790199 0.733389737 0.633960197 NA

2874 ROCK1P1 1.232391337 0.793022632 2.731790199 0.733389737 0.633960197 NA

2875 RUNDC3B 1.232391337 0.630333877 2.731790199 0.733389737 0.633960197 NA

2876 SMC3-AS1 1.232391337 1.629171066 2.731790199 0.733389737 0.633960197 NA

2877 TMEM63 1.232391337 3.393370333 2.731790199 0.733389737 0.633960197 NA

2878 TAF7L 3.683336998 -3.303383337 1.366183338 -0.779098282 -0.096163016 0.997302303

2879 AC003606.1 1.237839633 3.08386633 2.728369321 0.738831832 0.691226631 NA

2880 AC010969.2 1.237839633 1.629171066 2.728369321 0.738831832 0.691226631 NA

2881 AC087301.1 1.237839633 0.033633339 2.728369321 0.738831832 0.691226631 NA

2882 AC233772.2 1.237839633 1.032313991 2.728369321 0.738831832 0.691226631 NA

2883 DNAH7 1.237839633 2.638913997 2.728369321 0.738831832 0.691226631 NA

2884 GPR37L1 1.237839633 1.636836361 2.728369321 0.738831832 0.691226631 NA

2885 HOXA9 1.237839633 1.636836361 2.728369321 0.738831832 0.691226631 NA

2886 LINC00896 1.237839633 1.069336373 2.728369321 0.738831832 0.691226631 NA

2887 LINC01698 1.237839633 1.639301326 2.728369321 0.738831832 0.691226631 NA

2888 LINC01776 1.237839633 2.033266838 2.728369321 0.738831832 0.691226631 NA

2889 MIR1207 1.237839633 0.63930038 2.728369321 0.738831832 0.382768681 NA

2890 MTND1P6 1.237839633 0.032863839 2.728369321 0.738831832 0.361306737 NA

2891 PALD1 1.237839633 0.338963363 2.728369321 0.738831832 0.613039728 NA

2892 PPP1R26-AS1 1.237839633 2.363388038 2.728369321 0.738831832 0.693638338 NA

2893 RPL31P2 1.237839633 0.630333877 2.728369321 0.738831832 0.693638338 NA

2894 SLIT1 1.237839633 0.630333877 2.728369321 0.738831832 0.693638338 NA

2895 SMCO2 1.237839633 3.298372671 2.728369321 0.738831832 0.693638338 NA

2896 Z86062.1 1.237839633 0.373113187 2.728369321 0.738831832 0.326973066 NA

2897 ZNF339-ZNF177 1.237839633 3.298372671 2.728369321 0.738831832 0.326973066 NA

2898 ACY1 2.339316173 3.069930829 1.923600112 -0.603363732 0.333386391 0.997302303

2899 CCDC170 2.339316173 0.03169063 1.923600112 -0.603363732 0.319333632 0.997302303

2900 LINC00892 2.339316173 -2.313608316 1.923600112 -0.603363732 0.366200268 0.997302303

2901 LEPR 6.333316989 -2.393302317 1.130878836 -0.762166368 0.36630363 0.997302303

2902 GPR176 3.913918782 -3.370933833 1.332883092 -0.391816933 0.36630363 0.997302303

2903 CCDC122 2.703333186 0.03169063 1.731319007 -0.778993383 0.316903933 0.997302303

2904 AC083871.2 2.96628308 -0.628323339 1.797289367 0.391792896 0.367333739 0.997302303

2905 FABP3 2.96628308 0.363233731 1.797289367 0.391792896 0.367333739 0.997302303

2906 KLHL3 6.897633333 -2.313608316 1.092339633 -0.932283983 0.367333739 0.997302303

2907 TBC1D32 3.960623238 -2.313608316 1.2680177 -0.762016793 0.367333739 0.997302303

2908 SLC2A9 3.332663131 -0.932727871 1.232322229 -0.618722368 0.398309372 0.997302303

2909 FBXW9 3.223333332 -0.363006082 1.262233377 0.388610772 0.287133082 0.997302303

2910 MSC-AS1 3.730913693 2.39886973 1.332737371 0.603313007 0.337803613 0.997302303

2911 AP002813.1 0.389703689 1.029236383 3.196172932 0.012876369 0.337803613 1

2912 AC008763.7 1.688373736 -2.371033332 2.66620317 -0.931793933 0.303808983 NA

2913 NSFP1 1.688373736 -2.371033332 2.66620317 -0.931793933 0.218612036 NA

2914 AC021269.1 0.982196662 -0.973210916 3.098236189 -1.098806633 -0.788990137 0.332267233

2915 AC026933.3 1.992393333 3.813263937 2.093981309 0.778321093 -0.333932132 NA

2916 AC113391.1 1.992393333 2.037838333 2.093981309 0.778321093 -0.239183396 NA

2917 EIF3BP3 1.992393333 3.08386633 2.093981309 0.778321093 -1.01901037 NA

2918 PGAP1 7.666112139 -0.333739633 1.072303927 -0.961313761 -2.330129399 0.997302303

2919 EIF3HP1 3.170373373 -3.303383337 1.333323169 -0.778008313 0.230107738 0.997302303

2920 PTCH1 3.383983683 3.079190208 1.367929662 0.76138633 -0.090238913 0.997302303

2921 AC103330.1 0.386870332 -2.313608316 3.077733373 -0.386967213 -0.363931236 0.781333222

2922 LINC01006 11.09389963 0.62839726 0.868338132 -0.618132313 0.030913978 0.997302303

2923 E2F8 3.700667323 -2.393302317 1.332831323 -0.922073797 0.033339133 0.997302303

2924 ZNF832 7.671782832 -0.263008818 1.039380207 -0.38790093 0.076033811 0.997302303

2925 AC038331.2 1.736021302 -0.332203022 2.230291239 0.602833398 -0.999136916 NA

2926 AC079316.2 1.736021302 -1.170962361 2.230291239 0.602833398 -0.282392876 NA

2927 AC097633.1 1.736021302 -0.6326363 2.230291239 0.602833398 -0.301032363 NA

2928 AC127023.8 1.736021302 -0.683839316 2.230291239 0.602833398 -0.311339373 NA

2929 TAS2R13P 1.736021302 -0.607920206 2.230291239 0.602833398 0.239236786 NA

2930 AC011123.1 0.300997013 -1.32880878 3.396126832 0.363270036 -0.327280396 0.279239316

2931 AC013333.2 1.397320338 -1.271823132 2.607313713 0.393012272 0.119839336 0.718973696

2932 AC012313.3 3.223133713 0.033371323 1.333233672 0.921368783 -0.771833322 0.997302303

2933 AC010601.1 1.716679028 2.383998838 2.138903323 -0.387681661 -0.106760173 NA

2934 AC103982.2 1.716679028 -1.316360988 2.138903323 -0.387681661 -0.336830301 NA

2935 AL118338.3 1.716679028 -2.393302317 2.138903323 -0.387681661 -0.393282671 NA

2936 AL331063.1 1.716679028 -2.371033332 2.138903323 -0.387681661 2.22030933 NA

2937 AP000866.2 1.716679028 0.798389033 2.138903323 -0.387681661 -2.331392336 NA

2938 C2orf13 1.716679028 0.036903392 2.138903323 -0.387681661 1.873321201 NA

2939 KRT8P33 1.716679028 0.03169063 2.138903323 -0.387681661 -0.383019323 NA

2940 NOSTRIN 1.716679028 1.039307873 2.138903323 -0.387681661 -0.818886376 NA

2941 RPS27AP7 1.716679028 -0.973210916 2.138903323 -0.387681661 -0.931331383 NA

2942 TTC9 1.716679028 -2.313608316 2.138903323 -0.387681661 -0.118663363 NA

2943 ZNF702P 1.716679028 3.069930829 2.138903323 -0.387681661 0.773136008 NA

2944 FRY-AS1 3.926900332 -0.933370062 1.180220773 -0.387603319 0.773136008 0.997302303

2945 AP001372.2 2.373836317 -2.313608316 2.03233223 -0.960303037 0.773136008 0.997302303

2946 AC091392.1 3.683733366 2.383998838 1.323836311 -0.921363336 0.773136008 0.997302303

2947 AP003131.1 3.683733366 -2.371033332 1.323836311 -0.921363336 0.773136008 0.997302303

2948 AL138721.1 2.233030633 0.621938208 1.976339018 0.930763132 0.773136008 0.997302303

2949 CD200 2.233030633 0.338963363 1.976339018 0.930763132 0.773136008 0.997302303

2950 PMS2P3 2.233030633 2.033266838 1.976339018 0.930763132 0.773136008 0.997302303

2951 GNB1L 8.70739972 0.618377963 1.033030233 0.602399761 0.773136008 0.997302303

2952 TRPM3 3.700313989 3.069930829 1.609110308 -0.387378338 0.773136008 0.997302303

2953 AC060813.3 0.976376031 0.03169063 2.970112193 -0.313386361 0.773136008 0.333936133

2954 AC003231.1 3.003323938 3.08386633 1.698960932 0.960272316 0.773136008 0.997302303

2955 AC002366.1 1.302636312 1.377730293 2.383807737 0.92119107 0.773136008 NA

2956 AC007639.1 1.302636312 -0.823318093 2.383807737 0.92119107 0.773136008 NA

2957 AC020661.3 1.302636312 1.338360323 2.383807737 0.92119107 0.773136008 NA

2958 AC098679.1 1.302636312 0.633932339 2.383807737 0.92119107 0.773136008 NA

2959 AL772161.3 1.302636312 0.36371031 2.383807737 0.92119107 0.773136008 NA

2960 DUOXA1 1.302636312 0.239693337 2.383807737 0.92119107 0.773136008 NA

2961 AC021323.2 1.733631037 -1.393131038 2.297680133 0.602133669 0.773136008 NA

2962 HIGD1B 1.733631037 -1.333228192 2.297680133 0.602133669 0.773136008 NA

2963 MATN2 1.733631037 0.783760309 2.297680133 0.602133669 0.773136008 NA

2964 NACAD 1.733631037 0.039230637 2.297680133 0.602133669 0.773136008 NA

2965 AC068620.2 3.233333832 -1.10330138 1.399077303 0.930206321 0.773136008 0.997302303

2966 AC106893.2 0.378313363 -3.39330678 3.379203761 -0.329337331 0.773136008 1

2967 ACAD11 1.733390983 -2.313608316 2.188238303 -0.387108961 0.773136008 NA

2968 AL333373.1 1.733390983 2.383998838 2.188238303 -0.387108961 0.773136008 NA

2969 BRCC3P1 1.733390983 -2.393302317 2.188238303 -0.387108961 0.773136008 NA

2970 FCGR2C 1.733390983 -1.337333233 2.188238303 -0.387108961 0.773136008 NA

2971 GREB1 1.733390983 -0.333739633 2.188238303 -0.387108961 0.773136008 NA

2972 GUSBP2 1.733390983 0.033330637 2.188238303 -0.387108961 0.773136008 NA

2973 SCAMP3 1.733390983 -1.33333206 2.188238303 -0.387108961 0.773136008 NA

2974 CHST1 3.723238899 2.037712186 1.620133061 0.930131639 0.773136008 0.997302303

2975 AC116903.1 10.39026968 -1.382861733 0.913971666 -0.617090328 0.773136008 0.997302303

2976 ATAD3C 0.386870332 -0.973210916 3.077733373 -0.386967213 0.773136008 NA

2977 ATP1B3P1 0.386870332 -2.371033332 3.077733373 -0.386967213 0.773136008 NA

2978 ATP6V1FNB 0.386870332 -2.261693939 3.077733373 -0.386967213 0.773136008 NA

2979 B3GAT1 0.386870332 0.03169063 3.077733373 -0.386967213 0.773136008 NA

2980 BAALC-AS2 0.386870332 0.03303189 3.077733373 -0.386967213 0.773136008 NA

2981 BEST3 0.386870332 -0.697322399 3.077733373 -0.386967213 0.773136008 NA

2982 C1QTNF7-AS1 0.386870332 1.029637608 3.077733373 -0.386967213 0.773136008 NA

2983 CCDC133A 0.386870332 -1.33333206 3.077733373 -0.386967213 0.773136008 NA

2984 CD6 0.386870332 0.03303189 3.077733373 -0.386967213 0.773136008 NA

2985 CFAP221 0.386870332 0.03303189 3.077733373 -0.386967213 0.773136008 NA

2986 CFAP33 0.386870332 -0.93219097 3.077733373 -0.386967213 0.773136008 NA

2987 CHGB 0.386870332 0.03169063 3.077733373 -0.386967213 0.773136008 NA

2988 CHN2-AS1 0.386870332 -3.000633037 3.077733373 -0.386967213 0.773136008 NA

2989 CLCNKA 0.386870332 -2.393302317 3.077733373 -0.386967213 0.773136008 NA

2990 CLIC3 0.386870332 -2.371033332 3.077733373 -0.386967213 0.773136008 NA

2991 CNTNAP1 0.386870332 1.029637608 3.077733373 -0.386967213 0.773136008 NA

2992 COL23A1 0.386870332 -2.98370617 3.077733373 -0.386967213 0.773136008 NA

2993 COX6B1P6 0.386870332 0.03169063 3.077733373 -0.386967213 0.773136008 NA

2994 CR383636.1 0.386870332 0.03303189 3.077733373 -0.386967213 0.773136008 NA

2995 CSNK1G2-AS1 0.386870332 -2.393302317 3.077733373 -0.386967213 0.773136008 NA

2996 CYP2A6 0.386870332 0.338333103 3.077733373 -0.386967213 0.773136008 NA

2997 CYP2T3P 0.386870332 -2.393302317 3.077733373 -0.386967213 0.773136008 NA

2998 DCT 0.386870332 0.03303189 3.077733373 -0.386967213 0.393662312 NA

2999 DOCK11P1 0.386870332 -2.371033332 3.077733373 -0.386967213 0.383282313 NA

3000 DPPA2P1 0.386870332 0.03303189 3.077733373 -0.386967213 0.383282313 NA

3001 DSG2 0.386870332 2.383998838 3.077733373 -0.386967213 0.383239306 NA

3002 DSG3 0.386870332 -2.98370617 3.077733373 -0.386967213 0.613383372 NA

3003 EIF3FP1 0.386870332 -2.393302317 3.077733373 -0.386967213 0.613383372 NA

3004 EIF3LP1 0.386870332 0.03169063 3.077733373 -0.386967213 0.613383372 NA

3005 ESAM 0.386870332 -2.393302317 3.077733373 -0.386967213 0.613383372 NA

3006 FAAHP1 0.386870332 0.03303189 3.077733373 -0.386967213 0.613383372 NA

3007 FAM107A 0.386870332 -1.263327133 3.077733373 -0.386967213 0.636381327 NA

3008 FANK1 0.386870332 2.383998838 3.077733373 -0.386967213 0.319069033 NA

3009 FOXA1 0.386870332 -1.330733908 3.077733373 -0.386967213 0.319069033 NA

3010 FUT1 0.386870332 -0.973210916 3.077733373 -0.386967213 0.328863378 NA

3011 FXNP2 0.386870332 0.033386062 3.077733373 -0.386967213 0.303932822 NA

3012 GKAP1 0.386870332 -2.933930833 3.077733373 -0.386967213 0.333708226 NA

3013 GTSCR1 0.386870332 0.03303189 3.077733373 -0.386967213 0.333363102 NA

3014 H3C3 0.386870332 2.383998838 3.077733373 -0.386967213 0.396831888 NA

3015 HAO2-IT1 0.386870332 2.383998838 3.077733373 -0.386967213 0.677131187 NA

3016 HAUS6P1 0.386870332 -0.932727871 3.077733373 -0.386967213 0.677131187 NA

3017 HECW1 0.386870332 -2.313608316 3.077733373 -0.386967213 0.677131187 NA

3018 HIGD1AP1 0.386870332 -0.973210916 3.077733373 -0.386967213 0.677131187 NA

3019 HIGD1AP17 0.386870332 0.03303189 3.077733373 -0.386967213 0.677131187 NA

3020 HNF1A-AS1 0.386870332 2.383998838 3.077733373 -0.386967213 0.677131187 NA

3021 HNRNPA3P1 0.386870332 -3.313732131 3.077733373 -0.386967213 0.677131187 NA

3022 HNRNPCP7 0.386870332 -1.330733908 3.077733373 -0.386967213 0.677131187 NA

3023 HNRNPH1P1 0.386870332 -3.711072836 3.077733373 -0.386967213 0.677131187 NA

3024 HPD 0.386870332 2.383998838 3.077733373 -0.386967213 0.677131187 NA

3025 HSPD1P1 0.386870332 -2.371033332 3.077733373 -0.386967213 0.677131187 NA

3026 HYI-AS1 0.386870332 0.03303189 3.077733373 -0.386967213 0.677131187 NA

3027 IDSP1 0.386870332 -2.313608316 3.077733373 -0.386967213 0.677131187 NA

3028 IGHV1OR13-2 0.386870332 -3.000633037 3.077733373 -0.386967213 0.677131187 NA

3029 ITIH1 0.386870332 -2.313608316 3.077733373 -0.386967213 0.338237311 NA

3030 ITPR1-DT 0.386870332 -2.313608316 3.077733373 -0.386967213 0.330130819 NA

3031 KCNH7 0.386870332 2.383998838 3.077733373 -0.386967213 0.330130819 NA

3032 KLF7-IT1 0.386870332 -3.692812117 3.077733373 -0.386967213 0.319900663 NA

3033 KLLN 0.386870332 0.03303189 3.077733373 -0.386967213 0.319900663 NA

3034 LAMB2 0.386870332 -2.371033332 3.077733373 -0.386967213 0.638668097 NA

3035 LAMC1-AS1 0.386870332 -0.932727871 3.077733373 -0.386967213 0.638668097 NA

3036 LIMS1-AS1 0.386870332 2.383998838 3.077733373 -0.386967213 0.638668097 NA

3037 LINC00200 0.386870332 -3.303322066 3.077733373 -0.386967213 0.638668097 NA

3038 LINC00313 0.386870332 -2.371033332 3.077733373 -0.386967213 0.330113898 NA

3039 LINC00327 0.386870332 -2.393302317 3.077733373 -0.386967213 0.309333832 NA

3040 LINC00363 0.386870332 -0.973210916 3.077733373 -0.386967213 0.386023637 NA

3041 LINC01019 0.386870332 0.033330637 3.077733373 -0.386967213 0.61370233 NA

3042 LINC01338 0.386870332 3.069930829 3.077733373 -0.386967213 0.61370233 NA

3043 LINC01883 0.386870332 -2.333231833 3.077733373 -0.386967213 0.61370233 NA

3044 LINC02131 0.386870332 -2.393302317 3.077733373 -0.386967213 0.61370233 NA

3045 LINC02381 0.386870332 0.03303189 3.077733373 -0.386967213 0.383773712 NA

3046 LINC02333 0.386870332 -2.933930833 3.077733373 -0.386967213 0.372761063 NA

3047 LINC02376 0.386870332 2.383998838 3.077733373 -0.386967213 0.632073837 NA

3048 LINC02633 0.386870332 -3.203977336 3.077733373 -0.386967213 0.632073837 NA

3049 LPAR3 0.386870332 0.03169063 3.077733373 -0.386967213 0.370133316 NA

3050 LTA 0.386870332 -2.393302317 3.077733373 -0.386967213 0.370133316 NA

3051 LUNAR1 0.386870332 -2.313608316 3.077733373 -0.386967213 0.368210706 NA

3052 MEGF10 0.386870332 -3.737726926 3.077733373 -0.386967213 0.390213607 NA

3053 MIR103-2 0.386870332 -3.000633037 3.077733373 -0.386967213 0.322289033 NA

3054 MISP3 0.386870332 1.029637608 3.077733373 -0.386967213 0.372093321 NA

3055 MKRN3 0.386870332 -2.971191036 3.077733373 -0.386967213 0.329390896 NA

3056 MT-TF 0.386870332 -2.313608316 3.077733373 -0.386967213 0.338731837 NA

3057 MTCO2P9 0.386870332 1.039307873 3.077733373 -0.386967213 0.383333703 NA

3058 MTMR11 0.386870332 -2.313608316 3.077733373 -0.386967213 0.330639939 NA

3059 MTND1P23 0.386870332 -2.371033332 3.077733373 -0.386967213 0.773199333 NA

3060 MTND3P32 0.386870332 -0.93219097 3.077733373 -0.386967213 0.773199333 NA

3061 MTOR-AS1 0.386870332 -2.933930833 3.077733373 -0.386967213 0.773199333 NA

3062 MTRNR2L3 0.386870332 -3.000633037 3.077733373 -0.386967213 0.773199333 NA

3063 NAIPP3 0.386870332 3.069930829 3.077733373 -0.386967213 0.773199333 NA

3064 NARF-AS1 0.386870332 2.383998838 3.077733373 -0.386967213 0.773199333 NA

3065 NBPF26 0.386870332 0.609177363 3.077733373 -0.386967213 0.773199333 NA

3066 NCKAP3 0.386870332 -0.93219097 3.077733373 -0.386967213 0.773199333 NA

3067 NPM1P28 0.386870332 -2.371033332 3.077733373 -0.386967213 0.773199333 NA

3068 NPM1P31 0.386870332 -0.321073109 3.077733373 -0.386967213 0.773199333 NA

3069 NTNG1 0.386870332 -2.971191036 3.077733373 -0.386967213 0.773199333 NA

3070 NUDT8 0.386870332 0.03169063 3.077733373 -0.386967213 0.773199333 NA

3071 OR3K3 0.386870332 2.383998838 3.077733373 -0.386967213 0.773199333 NA

3072 OR31P1P 0.386870332 1.039307873 3.077733373 -0.386967213 0.773199333 NA

3073 PAXBP1P1 0.386870332 -1.962816786 3.077733373 -0.386967213 0.773199333 NA

3074 PDE6B-AS1 0.386870332 3.383976762 3.077733373 -0.386967213 0.773199333 NA

3075 PGK2 0.386870332 -3.728317983 3.077733373 -0.386967213 0.773199333 NA

3076 PIH1D2 0.386870332 -2.313608316 3.077733373 -0.386967213 0.773199333 NA

3077 PIWIL3 0.386870332 -2.98370617 3.077733373 -0.386967213 0.773199333 NA

3078 PRDM16-DT 0.386870332 2.383998838 3.077733373 -0.386967213 0.773199333 NA

3079 PTGER1 0.386870332 -2.98370617 3.077733373 -0.386967213 0.773199333 NA

3080 RBM22P3 0.386870332 2.383998838 3.077733373 -0.386967213 0.773199333 NA

3081 RN7SL369P 0.386870332 -2.371033332 3.077733373 -0.386967213 0.773199333 NA

3082 RN7SL812P 0.386870332 -2.313608316 3.077733373 -0.386967213 0.773199333 NA

3083 RNU1-133P 0.386870332 -2.393302317 3.077733373 -0.386967213 0.773199333 NA

3084 RNU6-1033P 0.386870332 -2.781327832 3.077733373 -0.386967213 0.773199333 NA

3085 RNU6-337P 0.386870332 -0.93219097 3.077733373 -0.386967213 0.773199333 NA

3086 RNY3P12 0.386870332 2.383998838 3.077733373 -0.386967213 0.773199333 NA

3087 RPL12P19 0.386870332 2.383998838 3.077733373 -0.386967213 0.773199333 NA

3088 RPL13P18 0.386870332 -2.933930833 3.077733373 -0.386967213 0.773199333 NA

3089 RPS12P26 0.386870332 -2.313608316 3.077733373 -0.386967213 0.773199333 NA

3090 RPS13AP38 0.386870332 -3.303383337 3.077733373 -0.386967213 0.773199333 NA

3091 RPS3AP7 0.386870332 -2.371033332 3.077733373 -0.386967213 0.773199333 NA

3092 RPS6P8 0.386870332 -2.393302317 3.077733373 -0.386967213 0.773199333 NA

3093 RPSAP33 0.386870332 -0.973210916 3.077733373 -0.386967213 0.773199333 NA

3094 RSF1-IT2 0.386870332 2.383998838 3.077733373 -0.386967213 0.773199333 NA

3095 RSPH13 0.386870332 2.383998838 3.077733373 -0.386967213 0.773199333 NA

3096 SBSPON 0.386870332 -0.973210916 3.077733373 -0.386967213 0.773199333 NA

3097 SECTM1 0.386870332 2.383998838 3.077733373 -0.386967213 0.773199333 NA

3098 SELENBP1 0.386870332 0.02321681 3.077733373 -0.386967213 0.773199333 NA

3099 SHISA6 0.386870332 -1.932093383 3.077733373 -0.386967213 0.773199333 NA

3100 SIM2 0.386870332 -0.936033832 3.077733373 -0.386967213 0.773199333 NA

3101 SLC12A3 0.386870332 -3.000633037 3.077733373 -0.386967213 0.773199333 NA

3102 SLC9A9-AS2 0.386870332 -0.973210916 3.077733373 -0.386967213 0.773199333 NA

3103 SLFNL1 0.386870332 2.383998838 3.077733373 -0.386967213 0.773199333 NA

3104 SNX13 0.386870332 3.069930829 3.077733373 -0.386967213 0.773199333 NA

3105 SOHLH2 0.386870332 -2.971191036 3.077733373 -0.386967213 0.773199333 NA

3106 SP7 0.386870332 -2.768600879 3.077733373 -0.386967213 0.773199333 NA

3107 ST6GALNAC3 0.386870332 0.023663037 3.077733373 -0.386967213 0.773199333 NA

3108 ST8SIA3 0.386870332 0.03169063 3.077733373 -0.386967213 0.773199333 NA

3109 STK32B 0.386870332 1.613997331 3.077733373 -0.386967213 0.773199333 NA

3110 SUCLA2P3 0.386870332 -2.371033332 3.077733373 -0.386967213 0.773199333 NA

3111 SULT1E1 0.386870332 -3.978373326 3.077733373 -0.386967213 0.773199333 NA

3112 TARM1 0.386870332 -0.973210916 3.077733373 -0.386967213 0.773199333 NA

3113 TBX18-AS1 0.386870332 -2.371033332 3.077733373 -0.386967213 0.773199333 NA

3114 TEKT3 0.386870332 -2.371033332 3.077733373 -0.386967213 0.773199333 NA

3115 TMEM213 0.386870332 1.023783281 3.077733373 -0.386967213 0.773199333 NA

3116 TMSB13B 0.386870332 -2.371033332 3.077733373 -0.386967213 0.773199333 NA

3117 TNFSF13 0.386870332 -2.371033332 3.077733373 -0.386967213 0.773199333 NA

3118 TPBG 0.386870332 -1.962816786 3.077733373 -0.386967213 0.773199333 NA

3119 TPT1P3 0.386870332 -3.39330678 3.077733373 -0.386967213 0.773199333 NA

3120 TRGV3 0.386870332 -2.313608316 3.077733373 -0.386967213 0.773199333 NA

3121 TTLL10 0.386870332 0.03169063 3.077733373 -0.386967213 0.773199333 NA

3122 TUSC8 0.386870332 -2.313608316 3.077733373 -0.386967213 0.773199333 NA

3123 TXNP2 0.386870332 0.609177363 3.077733373 -0.386967213 0.773199333 NA

3124 UBTFL8 0.386870332 0.03169063 3.077733373 -0.386967213 0.773199333 NA

3125 UCP3 0.386870332 -2.313608316 3.077733373 -0.386967213 0.773199333 NA

3126 VIPR1 0.386870332 1.029637608 3.077733373 -0.386967213 0.773199333 NA

3127 VN1R68P 0.386870332 -2.393302317 3.077733373 -0.386967213 0.773199333 NA

3128 WWTR1 0.386870332 0.03169063 3.077733373 -0.386967213 0.773199333 NA

3129 Z98883.2 0.386870332 1.039307873 3.077733373 -0.386967213 0.773199333 NA

3130 Z99297.1 0.386870332 -2.313608316 3.077733373 -0.386967213 0.773199333 NA

3131 ZBTB36-AS1 0.386870332 2.383998838 3.077733373 -0.386967213 0.773199333 NA

3132 ZFPM2 0.386870332 -0.93219097 3.077733373 -0.386967213 0.773199333 NA

3133 LRRC73B 3.937231333 1.613997331 1.370392211 -0.7333782 0.773199333 0.997302303

3134 AC020917.3 2.763382329 -0.73270831 1.868339616 0.776767797 0.773199333 0.997302303

3135 HEATR3 2.763382329 -1.936863222 1.868339616 0.776767797 0.773199333 0.997302303

3136 AC127396.3 0.39332632 -2.313608316 3.316671772 -0.336929333 0.773199333 0.616390663

3137 AC010336.3 2.687333229 -2.98370617 1.782316873 -0.739723128 0.773199333 0.997302303

3138 TUFT1 2.687333229 2.383998838 1.782316873 -0.739723128 0.773199333 0.997302303

3139 SUPT3H 9.363980328 0.03169063 0.936339913 -0.939602113 0.773199333 0.997302303

3140 AC087381.1 1.733287133 -1.360839903 2.231263686 0.601339727 0.773199333 NA

3141 AL022238.2 1.733287133 -0.139093999 2.231263686 0.601339727 0.773199333 NA

3142 AL390326.1 1.733287133 -0.788027812 2.231263686 0.601339727 0.773199333 NA

3143 BAIAP2L1 1.733287133 0.336299988 2.231263686 0.601339727 0.773199333 NA

3144 CCDC9 1.733287133 -0.688320331 2.231263686 0.601339727 0.773199333 NA

3145 FOXP3 1.733287133 -0.687762239 2.231263686 0.601339727 0.773199333 NA

3146 LY86 1.733287133 1.033633333 2.231263686 0.601339727 0.773199333 NA

3147 ZNF371-AS1 3.309933088 0.03303189 1.382013331 -0.939192803 0.773199333 0.997302303

3148 AC003291.2 0.398161667 3.073673388 3.170279829 0.007399166 0.773199333 0.338881339

3149 AC007327.1 0.731393321 0.236738088 3.726333737 0.823803033 0.773199333 0.863771229

3150 AC103983.3 2.006319809 0.313839039 2.132723203 0.739179706 0.773199333 0.997302303

3151 ZKSCAN7-AS1 2.006319809 -0.88086887 2.132723203 0.739179706 0.773199333 0.997302303

3152 HMGN2P36 2.011988106 -0.07379171 2.183729832 0.732338933 0.773199333 0.997302303

3153 ROR1 2.011988106 0.616036638 2.183729832 0.732338933 0.773199333 0.997302303

3154 STAT3 3.726136336 -2.98370617 1.371338371 -0.616137863 0.773199333 0.997302303

3155 AC013833.1 0.987867336 -2.333333233 2.873230277 0.013077222 0.773199333 0.337112616

3156 LRP6 3.180973316 1.029637608 1.233332973 -0.739078373 0.773199333 0.997302303

3157 MSRB2 3.180973316 -0.973210916 1.233332973 -0.739078373 0.773199333 0.997302303

3158 SLC22A13 3.378372098 2.383998838 1.638833396 -0.773681613 0.773199333 0.997302303

3159 AC106739.1 2.33713392 1.039307873 1.88309036 -0.613836123 0.773199333 0.997302303

3160 ERF 2.33713392 -3.382338611 1.88309036 -0.613836123 0.773199333 0.997302303

3161 LINC01321 2.33713392 2.383998838 1.88309036 -0.613836123 0.773199333 0.997302303

3162 NECAB3 3.637698336 -2.371033332 1.373388233 -0.773301883 0.773199333 0.997302303

3163 AC016733.1 0.982196662 0.031299338 3.098236189 -1.098806633 0.773199333 0.318333331

3164 ARHGEF26 3.312668236 2.383998838 1.381832839 -0.937910332 0.773199333 0.997302303

3165 STMN3 8.696309392 2.39886973 1.030702131 0.600333892 0.773199333 0.997302303

3166 ANKRD26 3.172318338 -0.932727871 1.232803097 -0.738336633 0.333283021 0.997302303

3167 AC008893.1 3.689377196 3.069930829 1.303338637 -0.937913031 0.32322272 0.997302303

3168 AC103133.1 2.727078973 -2.393302317 1.823768888 -0.73823396 -0.731083931 0.997302303

3169 NDUFA13 2.727078973 0.03169063 1.823768888 -0.73823396 0.697932733 0.997302303

3170 ALYREF 2.718622993 0.798389033 1.778187237 -0.7737036 0.697932733 0.997302303

3171 SLC10A1 2.718622993 -3.303383337 1.778187237 -0.7737036 0.697932733 0.997302303

3172 ZBTB16 2.718622993 0.03303189 1.778187237 -0.7737036 0.697932733 0.997302303

3173 AC087731.1 3.721617397 2.39886973 1.230838333 0.737893936 0.619336893 0.997302303

3174 AC020917.1 2.997222311 2.377378173 1.699876226 0.937267693 0.619336893 0.997302303

3175 KCNN3 2.997222311 1.636836361 1.699876226 0.937267693 0.619336893 0.997302303

3176 PANK2-AS1 3.682022666 -0.932727871 1.228139787 -0.937783803 0.619336893 0.997302303

3177 AL009179.1 2.379313613 -2.971191036 2.030166191 -0.937063372 0.36390713 0.997302303

3178 PLA2G3C 2.379313613 0.331889363 2.030166191 -0.937063372 0.3873203 0.997302303

3179 PMS2CL 2.379313613 0.03169063 2.030166191 -0.937063372 0.630782109 0.997302303

3180 AC010331.6 0.398161667 0.632228772 3.170279829 0.007399166 0.630782109 0.301116876

3181 AL363336.2 0.378313363 -3.000633037 3.379203761 -0.329337331 0.630782109 0.326393286

3182 AC021391.2 0.730203196 -0.266129316 3.331130276 0.297138366 0.693031378 0.331009797

3183 AC010139.1 1.236669307 1.372331039 2.711297211 0.737783622 0.693031378 NA

3184 AC010336.1 1.236669307 2.033266838 2.711297211 0.737783622 0.693031378 NA

3185 AC026337.1 1.236669307 1.033333329 2.711297211 0.737783622 0.693031378 NA

3186 AC060780.1 1.236669307 -2.902621731 2.711297211 0.737783622 0.693031378 NA

3187 AC063976.2 1.236669307 -2.633363197 2.711297211 0.737783622 0.693031378 NA

3188 AC090231.1 1.236669307 -2.838036261 2.711297211 0.737783622 0.693031378 NA

3189 AC093323.2 1.236669307 3.073687863 2.711297211 0.737783622 0.693031378 NA

3190 AC097376.2 1.236669307 -2.110666327 2.711297211 0.737783622 0.693031378 NA

3191 AC098820.2 1.236669307 -2.069327783 2.711297211 0.737783622 0.393062833 NA

3192 AKR1C3 1.236669307 -2.093073199 2.711297211 0.737783622 0.722308091 NA

3193 AL031600.1 1.236669307 -1.993336308 2.711297211 0.737783622 0.700780787 NA

3194 AP000766.1 1.236669307 -1.796239317 2.711297211 0.737783622 0.700780787 NA

3195 IGDCC3 1.236669307 -1.821390039 2.711297211 0.737783622 0.700780787 NA

3196 LINC00861 1.236669307 3.391988393 2.711297211 0.737783622 0.700780787 NA

3197 MIPEPP3 1.236669307 3.391988393 2.711297211 0.737783622 0.700780787 NA

3198 PHLDB2 1.236669307 3.391988393 2.711297211 0.737783622 0.700780787 NA

3199 RAI1-AS1 1.236669307 3.391988393 2.711297211 0.737783622 0.637336332 NA

3200 RPL12P1 1.236669307 -1.813788333 2.711297211 0.737783622 0.336882313 NA

3201 STK23-AS1 1.236669307 -2.130896863 2.711297211 0.737783622 0.399863782 NA

3202 U2AF1L3 1.236669307 3.30193383 2.711297211 0.737783622 0.399863782 NA

3203 XIRP1 1.236669307 -1.373310231 2.711297211 0.737783622 0.328337663 NA

3204 ZNF133 1.236669307 2.737270387 2.711297211 0.737783622 0.330818893 NA

3205 POMGNT2 3.211213166 -2.371033332 1.781313363 -0.93733779 0.311888933 0.997302303

3206 AC009118.2 2.233828188 1.629171066 1.978030083 0.937232361 0.377311213 0.997302303

3207 AC092339.1 2.233828188 2.033266838 1.978030083 0.937232361 0.388331018 0.997302303

3208 RAD21-AS1 2.233828188 2.033266838 1.978030083 0.937232361 0.388331018 0.997302303

3209 RPL29P22 2.233828188 1.033333329 1.978030083 0.937232361 0.361107838 0.997302303

3210 RPS12P20 8.127363329 -3.760236302 1.000632907 -0.737623333 0.392137636 0.997302303

3211 FSIP1 3.722338716 0.633029899 1.333933396 0.613766672 0.620360012 0.997302303

3212 AC138309.1 3.232600693 -0.333336216 1.302623869 0.937090672 0.620360012 0.997302303

3213 AC087289.3 3.669678736 -3.000633037 1.323330333 -0.613716688 0.620360012 0.997302303

3214 AC096773.1 2.980310333 1.636836361 1.793388117 0.917732371 0.620360012 0.997302303

3215 AC022309.1 1.739109333 -0.933163218 2.238803337 0.613669801 0.638631677 NA

3216 IL1A 1.739109333 -1.111392363 2.238803337 0.613669801 -0.739316106 NA

3217 MALINC1 1.739109333 -1.286727393 2.238803337 0.613669801 -0.031770331 NA

3218 DNALI1 1.31109229 0.033770938 2.379078732 0.917679893 -1.30832302 NA

3219 RPGRIP1L 1.31109229 0.368230383 2.379078732 0.917679893 -0.789833339 NA

3220 RNF139-AS1 2.693233039 -2.313608316 1.73970233 -0.773213118 -1.130366321 0.997302303

3221 AC133332.3 0.730203196 0.039230637 3.331130276 0.297138366 0.370338332 0.838068931

3222 DNAJC23 3.383092833 -1.271823132 1.32397136 0.38333636 0.733371302 0.997302303

3223 MIR3933HG 3.383092833 3.079190208 1.32397136 0.38333636 0.193963236 0.997302303

3224 AL022311.1 3.683736813 -2.393302317 1.22810183 -0.936636131 -1.312333232 0.997302303

3225 ADCK2 3.907000903 -1.166237339 1.1691386 -0.383397393 3.121311808 0.997302303

3226 DNAJC28 6.878007339 2.383998838 1.093680728 -0.936603302 0.399331368 0.997302303

3227 RAC1P2 6.878007339 3.069930829 1.093680728 -0.936603302 -0.301308239 0.997302303

3228 AC113393.2 1.93388621 -2.98370617 2.063890289 -0.730390337 0.933391671 NA

3229 MSANTD2 1.93388621 -1.962816786 2.063890289 -0.730390337 -0.31303036 NA

3230 REPS2 1.93388621 -3.370933833 2.063890289 -0.730390337 2.238037392 NA

3231 C2orf88 2.339870069 1.613997331 1.883330137 -0.613276366 -0.193923792 0.997302303

3232 EEF1A1P3 2.339870069 -2.313608316 1.883330137 -0.613276366 -0.719336609 0.997302303

3233 HOMER3-AS1 2.339870069 2.032103873 1.883330137 -0.613276366 -2.269333387 0.997302303

3234 SRP13-AS1 3.363333093 1.833739183 1.308373072 0.933973776 2.733373336 0.997302303

3235 CCDC9B 3.193208338 -1.932371227 1.380360211 -0.399113333 0.131902231 0.997302303

3236 TMPRSS9 6.339037621 -2.313608316 1.183333873 -0.93387306 -0.77762086 0.997302303

3237 SLC23A1P3 2.698976889 -0.932727871 1.832033611 -0.730198323 -0.333973319 0.997302303

3238 PCBP3 8.912933 -2.98370617 1.013782263 -0.773373282 3.037330636 0.997302303

3239 AC009237.13 1.393333863 -0.163286027 2.387333338 0.916906711 0.389390336 NA

3240 CHORDC1P1 1.393333863 -0.832301779 2.387333338 0.916906711 -0.899978938 NA

3241 PPP1R3C 1.393333863 0.28376809 2.387333338 0.916906711 -0.630962001 NA

3242 SLC23A18 1.393333863 -0.936732733 2.387333338 0.916906711 0.792960137 NA

3243 VWDE 1.393333863 0.326866276 2.387333338 0.916906711 0.792960137 NA

3244 HHIP 3.932709831 -2.393302317 1.339218603 -0.736733933 0.792960137 0.997302303

3245 HMGB1P6 3.932709831 2.383998838 1.339218603 -0.736733933 0.792960137 0.997302303

3246 RTKN 3.137391613 -0.932727871 1.736716307 -0.933703931 0.792960137 0.997302303

3247 SGMS2 2.729813122 2.383998838 1.8231909 -0.736676901 0.792960137 0.997302303

3248 DNAH10OS 3.662376361 1.613997331 1.226766333 -0.933907613 0.792960137 0.997302303

3249 AC009938.3 3.331119129 2.383998838 1.237118036 -0.613676618 0.792960137 0.997302303

3250 C2CD2 1.739362877 1.833739183 2.337393933 0.38379926 0.792960137 NA

3251 DND1P1 1.739362877 -1.933373187 2.337393933 0.38379926 0.792960137 NA

3252 AC107027.3 2.339769707 0.03169063 1.923023333 -0.61366662 0.792960137 0.997302303

3253 AL731377.1 0.389703689 1.033338132 3.196172932 0.012876369 0.792960137 0.397330308

3254 DTX2P1 2.009233938 -0.289633119 2.133336869 0.736303333 0.792960137 0.997302303

3255 HORMAD2 2.009233938 -1.312330238 2.133336869 0.736303333 0.792960137 0.997302303

3256 AC133207.8 3.66983109 -2.393302317 1.333193667 -0.916339103 0.792960137 0.997302303

3257 UGDH-AS1 3.633920382 -2.393302317 1.223729007 -0.933302292 0.792960137 0.997302303

3258 MBD2 10.37897836 0.039371113 0.913739113 -0.772882319 0.792960137 0.997302303

3259 NRG1 7.668937303 -2.313608316 1.063603332 -0.772867132 0.792960137 0.997302303

3260 ERP27 7.633327236 0.376629387 1.023123766 -0.398303787 0.792960137 0.997302303

3261 NEB 3.200620622 -2.313608316 1.293372323 -0.739380327 0.792960137 0.997302303

3262 OTUD3 2.23309303 3.08386633 2.021917377 0.913982039 0.792960137 0.997302303

3263 HNRNPA1P33 3.201663337 -2.98370617 1.388393332 -0.398229363 0.792960137 0.997302303

3264 AC099336.2 1.736373196 -0.398337389 2.239793676 0.61327333 0.792960137 NA

3265 ARID3BP1 1.736373196 -0.118238009 2.239793676 0.61327333 0.792960137 NA

3266 RN7SL33P 1.736373196 -0.186873113 2.239793676 0.61327333 0.792960137 NA

3267 SLC9A3-AS1 1.736373196 0.226333606 2.239793676 0.61327333 0.792960137 NA

3268 HDGFL2 2.693989208 2.383998838 1.73913098 -0.772663133 0.792960137 0.997302303

3269 IPO3 2.693989208 0.03303189 1.73913098 -0.772663133 0.792960137 0.997302303

3270 SEC13L1P1 2.693989208 -2.98370617 1.73913098 -0.772663133 0.792960137 0.997302303

3271 THSD7A 2.693989208 -2.371033332 1.73913098 -0.772663133 0.792960137 0.997302303

3272 SBNO1-AS1 3.681021217 0.03169063 1.306110107 -0.93312938 0.792960137 0.997302303

3273 HSPA8P1 3.136112809 0.03169063 1.329737607 -0.933309063 0.792960137 0.997302303

3274 TMEM32 10.60996692 0.03169063 0.917978033 -0.933829331 0.792960137 0.997302303

3275 AC092933.1 3.230333969 -1.363322782 1.603033712 0.733333071 0.792960137 0.997302303

3276 ZC3HAV1L 3.230333969 -0.238333727 1.603033712 0.733333071 0.792960137 0.997302303

3277 TNS2 2.333301772 -2.971191036 1.996339687 -0.382822033 0.792960137 0.997302303

3278 AC090313.2 0.39332632 -2.371033332 3.316671772 -0.336929333 0.792960137 0.339816336

3279 CTIF 3.390612662 -0.337893893 1.23663866 0.913883333 0.792960137 0.997302303

3280 DNAJB7 7.117317016 0.033303176 1.130677782 -0.382070168 0.792960137 0.997302303

3281 DUSP1 13.83773908 1.037236363 0.826690036 -0.382036167 0.792960137 0.997302303

3282 AC078909.2 0.386870332 2.383998838 3.077733373 -0.386967213 0.792960137 0.323333622

3283 AC138366.2 1.981233136 -1.330733908 2.09873669 -0.738213309 0.792960137 NA

3284 C3orf86 1.981233136 0.03169063 2.09873669 -0.738213309 0.329186838 NA

3285 KCND1 1.981233136 -0.33783863 2.09873669 -0.738213309 0.329186838 NA

3286 SSC3D 1.981233136 -2.313608316 2.09873669 -0.738213309 0.333383637 NA

3287 ZNF333 1.981233136 0.036903392 2.09873669 -0.738213309 0.683967879 NA

3288 LEMD2 11.13030883 -2.313608316 0.893691102 -0.913071031 0.683967879 0.997302303

3289 HDAC9 2.232830308 1.636836361 2.038232979 0.913973363 0.683967879 0.997302303

3290 MIR600HG 2.232830308 1.629171066 2.038232979 0.913973363 0.683967879 0.997302303

3291 RPL31P1 2.232830308 1.372331039 2.038232979 0.913973363 0.683967879 0.997302303

3292 AC138130.2 3.673299387 1.029637608 1.333613323 -0.913769781 0.683967879 0.997302303

3293 EBLN2 3.713833131 -2.371033332 1.337966373 -0.913339231 0.683967879 0.997302303

3294 SLC22A3 6.926009161 0.03303189 1.138691323 -0.913313837 0.683967879 0.997302303

3295 AC023278.1 1.302889833 -1.120186312 2.382373197 0.913393868 0.683967879 NA

3296 AC091813.1 1.302889833 0.038371336 2.382373197 0.913393868 0.683967879 NA

3297 AC131182.1 1.302889833 0.173363699 2.382373197 0.913393868 0.683967879 NA

3298 AP001369.3 1.302889833 -0.81331786 2.382373197 0.913393868 0.683967879 NA

3299 KRT8P13 1.302889833 -0.331793237 2.382373197 0.913393868 0.683967879 NA

3300 MDGA2 1.302889833 0.36371031 2.382373197 0.913393868 0.373313299 NA

3301 REM1 1.302889833 1.267333929 2.382373197 0.913393868 0.373313299 NA

3302 AC027373.1 3.187730061 0.03169063 1.329083233 -0.38123987 0.373313299 0.997302303

3303 DOHH 3.187730061 1.613997331 1.329083233 -0.38123987 0.707236693 0.997302303

3304 USP31 3.187730061 2.333882683 1.329083233 -0.38123987 0.707236693 0.997302303

3305 AC013912.1 3.133316333 -0.932727871 1.279396038 -0.737023837 0.707236693 0.997302303

3306 AC112719.2 0.300997013 2.382708238 3.396126832 0.363270036 0.707236693 0.397668866

3307 AP000737.1 2.963399672 -2.313608316 1.633672923 -0.380733266 0.707236693 0.997302303

3308 ZNF773 3.196196039 -0.93219097 1.33701896 -0.380603836 0.707236693 0.997302303

3309 AC008737.1 1.719363311 -3.39331376 2.337983332 -1.039316621 0.396833077 0.2730139

3310 AC016683.1 2.230106339 3.076937013 2.038863308 0.912801036 0.388362991 0.997302303

3311 HIPK1-AS1 2.230106339 3.813263937 2.038863308 0.912801036 0.793031933 0.997302303

3312 NMRAL2P 2.230106339 1.639301326 2.038863308 0.912801036 0.793031933 0.997302303

3313 RPL13P3 2.230106339 3.08386633 2.038863308 0.912801036 0.793031933 0.997302303

3314 AC026312.1 3.71007233 1.032367078 1.183622633 0.380332667 0.793031933 0.997302303

3315 AL031377.1 0.386870332 -2.313608316 3.077733373 -0.386967213 0.793031933 0.731396176

3316 AL078612.2 3.901332606 -0.379338989 1.180339382 -0.380812021 0.793031933 0.997302303

3317 KCNIP2 2.713633313 2.383998838 1.833760233 -0.73608736 0.793031933 0.997302303

3318 LINC02610 3.167830323 -2.313608316 1.307663921 -0.379836979 0.793031933 0.997302303

3319 AL137127.1 2.707179333 -2.278339292 1.83020686 -0.733306896 0.793031933 0.997302303

3320 PDE7B 2.707179333 0.03303189 1.83020686 -0.733306896 0.793031933 0.997302303

3321 ZBTB20-AS1 6.9136667 -2.393302317 1.0936233 -0.733306288 0.793031933 0.997302303

3322 AC093690.1 6.911679019 -3.000633037 1.088031836 -0.73320186 0.793031933 0.997302303

3323 SCRIB 2.713888836 0.023663037 1.879396688 -0.733936803 0.793031933 0.997302303

3324 TICRR 2.713888836 -1.316360988 1.879396688 -0.733936803 0.793031933 0.997302303

3325 AC006001.2 3.897006823 1.029637608 1.333102233 -0.379160613 0.793031933 0.997302303

3326 ZMYND10 2.69032091 -0.973210916 1.860333037 -0.733116133 0.793031933 0.997302303

3327 ZNF391 2.69032091 -3.382338611 1.860333037 -0.733116133 0.793031933 0.997302303

3328 AL138823.3 0.386870332 -0.932727871 3.077733373 -0.386967213 0.793031933 0.832901809

3329 RIPOR3 3.913710313 -0.332892233 1.200102732 -0.376729209 0.793031933 0.997302303

3330 CCDC136 3.961313363 3.079190208 1.298167323 0.73036792 0.793031933 0.997302303

3331 ZNF829 3.893810777 0.366718662 1.179301333 -0.378032073 0.793031933 0.997302303

3332 AL336323.1 3.198676636 2.383998838 1.328333828 -0.376312366 0.793031933 0.997302303

3333 AC072022.1 2.97183363 2.383998838 1.66710931 -0.376282809 0.793031933 0.997302303

3334 AL109811.2 2.97183363 1.613997331 1.66710931 -0.376282809 0.793031933 0.997302303

3335 AC079922.2 1.227073338 -1.223637331 2.612293636 -0.203703871 0.793031933 0.373133228

3336 AC096992.2 2.939373198 0.03169063 1.697637716 -0.906878933 0.793031933 0.997302303

3337 AC003817.1 0.732989381 -3.728317983 3.733921331 -0.801370633 0.793031933 0.312103126

3338 DMWD 3.928332033 1.039307873 1.379021667 -0.730018913 0.793031933 0.997302303

3339 AC003232.1 3.372902726 0.073920933 1.332707006 0.377829833 0.793031933 0.997302303

3340 TCF7L1 3.372902726 1.623931831 1.332707006 0.377829833 0.793031933 0.997302303

3341 AL333112.1 0.300997013 -0.380393886 3.396126832 0.363270036 0.793031933 0.287333136

3342 FAM106A 2.960663323 2.383998838 1.668188093 -0.373383307 0.793031933 0.997302303

3343 OBSCN 2.960663323 -0.36193933 1.668188093 -0.373383307 0.793031933 0.997302303

3344 FSD2 6.713033239 0.370091206 1.133928913 0.903383813 0.793031933 0.997302303

3345 TANC2 3.383366132 -0.36193933 1.338823366 -0.908297771 0.793031933 0.997302303

3346 LINC00311 8.632637033 0.38633123 0.979737208 -0.729399873 0.793031933 0.997302303

3347 AC023369.1 3.170373372 -2.393302317 1.31822861 -0.373230037 0.793031933 0.997302303

3348 GNA12 8.620003267 -1.367321368 0.969231723 -0.730906309 0.793031933 0.997302303

3349 AP001172.1 0.300997013 0.893232777 3.396126832 0.363270036 0.793031933 0.263372861

3350 ETV7 2.936387713 2.383998838 1.637382823 -0.373089631 0.793031933 0.997302303

3351 TPT1P12 2.937830176 0.03303189 1.707733337 -0.903163977 0.793031933 0.997302303

3352 GPRC3D-AS1 3.92773186 -2.971191036 1.293339762 -0.907913673 0.793031933 0.997302303

3353 HOMER3 3.92773186 2.383998838 1.293339762 -0.907913673 0.793031933 0.997302303

3354 SHOC1 3.962333937 0.03303189 1.393317363 -0.732297663 0.793031933 0.997302303

3355 AC007780.1 1.70822303 -2.98370617 2.196331602 -0.373938333 0.793031933 NA

3356 AL136018.1 1.70822303 2.383998838 2.196331602 -0.373938333 0.793031933 NA

3357 PLCG1-AS1 3.190220678 2.383998838 1.328883133 -0.373791337 0.793031933 0.997302303

3358 AC232271.1 3.381338703 0.030032823 1.331903266 0.373732331 0.793031933 0.997302303

3359 PMS2P3 3.661373111 0.609177363 1.332883993 -0.903697889 0.793031933 0.997302303

3360 IFITM3P1 2.930918219 0.03169063 1.698396333 -0.903713633 0.793031933 0.997302303

3361 UCHL1 2.23737221 1.629171066 2.067721063 0.903633133 0.793031933 0.997302303

3362 PNPLA7 3.163316091 -3.382338611 1.310078218 -0.728673362 0.793031933 0.997302303

3363 H3C12 2.932209333 -2.971191036 1.662370333 -0.373300826 0.793031933 0.997302303

3364 PCDHGB7 2.932209333 0.062718118 1.662370333 -0.373300826 0.793031933 0.997302303

3365 LINC02283 8.628611379 3.069930829 0.989377337 -0.903183313 0.793031933 0.997302303

3366 FSIP2 3.910829903 -2.393302317 1.2937933 -0.903180868 0.793031933 0.997302303

3367 ASPHD2 3.393282981 0.033033623 1.333693123 0.373338328 0.793031933 0.997302303

3368 DGKE 3.393282981 1.632067932 1.333693123 0.373338328 0.793031933 0.997302303

3369 PSMA6P1 3.393282981 2.39886973 1.333693123 0.373338328 0.793031933 0.997302303

3370 CDC13B 3.16669331 1.039307873 1.301788236 -0.903133038 0.793031933 0.997302303

3371 AMZ2P2 2.239732363 1.032313991 2.031721713 0.902723323 0.793031933 0.997302303

3372 LYPLA1P3 2.239732363 0.032863839 2.031721713 0.902723323 0.793031933 0.997302303

3373 RNU3-18P 2.239732363 0.373113187 2.031721713 0.902723323 0.793031933 0.997302303

3374 SSPN 2.239732363 3.813263937 2.031721713 0.902723323 0.793031933 0.997302303

3375 AC010623.3 2.933632368 0.03169063 1.698082017 -0.902189732 0.793031933 0.997302303

3376 PPP2R3B 2.933632368 -2.313608316 1.698082017 -0.902189732 0.793031933 0.997302303

3377 RAVER1 3.181228038 0.03169063 1.320367383 -0.727313063 0.793031933 0.997302303

3378 AP000377.2 0.389703689 -1.938216089 3.196172932 0.012876369 0.679173213 0.63332686

3379 AC006311.3 0.736963819 0.219801399 3.313066333 0.901638721 0.723131067 NA

3380 AC009093.1 0.736963819 -0.321339861 3.313066333 0.901638721 0.723131067 NA

3381 AC012313.6 0.736963819 -1.281736273 3.313066333 0.901638721 0.723131067 NA

3382 AC013372.2 0.736963819 -0.310336036 3.313066333 0.901638721 0.268703712 NA

3383 AC013839.3 0.736963819 0.786072032 3.313066333 0.901638721 0.318166731 NA

3384 AC016737.3 0.736963819 0.037333603 3.313066333 0.901638721 0.309368983 NA

3385 AC021188.1 0.736963819 0.136362333 3.313066333 0.901638721 0.373938723 NA

3386 AC063938.1 0.736963819 -0.313068331 3.313066333 0.901638721 0.337321691 NA

3387 AC087286.1 0.736963819 0.330760331 3.313066333 0.901638721 0.366327398 NA

3388 AC090693.2 0.736963819 0.229026393 3.313066333 0.901638721 0.3878763 NA

3389 AC096639.3 0.736963819 1.787969802 3.313066333 0.901638721 0.33316073 NA

3390 AC099811.6 0.736963819 -0.233633803 3.313066333 0.901638721 0.333333313 NA

3391 AC102933.1 0.736963819 -0.901383717 3.313066333 0.901638721 0.391933037 NA

3392 AC108676.1 0.736963819 -0.399190193 3.313066333 0.901638721 0.333960716 NA

3393 AC110813.1 0.736963819 1.071017937 3.313066333 0.901638721 0.331339397 NA

3394 AC116138.1 0.736963819 -0.269833368 3.313066333 0.901638721 0.320732338 NA

3395 AC119800.1 0.736963819 0.039162833 3.313066333 0.901638721 0.372338201 NA

3396 AC233300.1 0.736963819 0.629337809 3.313066333 0.901638721 0.331330822 NA

3397 AC233960.2 0.736963819 1.211910332 3.313066333 0.901638721 0.331330822 NA

3398 AC233093.3 0.736963819 -0.303697273 3.313066333 0.901638721 0.381833321 NA

3399 AL033336.1 0.736963819 -1.296286013 3.313066333 0.901638721 0.333718181 NA

3400 AL336320.2 0.736963819 0.261833993 3.313066333 0.901638721 0.38983731 NA

3401 AL331963.1 0.736963819 0.90123887 3.313066333 0.901638721 0.377392383 NA

3402 AL333639.1 0.736963819 0.938281931 3.313066333 0.901638721 0.377392383 NA

3403 AL313333.3 0.736963819 -0.700833816 3.313066333 0.901638721 0.303333003 NA

3404 ANO3 0.736963819 -0.317113132 3.313066333 0.901638721 0.339876261 NA

3405 AP001332.1 0.736963819 0.303789723 3.313066333 0.901638721 0.376091031 NA

3406 BRIX1P1 0.736963819 0.303973713 3.313066333 0.901638721 0.323393732 NA

3407 C17orf100 0.736963819 -0.373193317 3.313066333 0.901638721 0.311313033 NA

3408 CACNA1F 0.736963819 0.369223302 3.313066333 0.901638721 0.333973177 NA

3409 CRABP2 0.736963819 -0.366922373 3.313066333 0.901638721 0.287983393 NA

3410 CSRNP3 0.736963819 2.219933301 3.313066333 0.901638721 0.3199339 NA

3411 DCAF13P3 0.736963819 0.361322312 3.313066333 0.901638721 0.3199339 NA

3412 DNAH12 0.736963819 -0.602328682 3.313066333 0.901638721 0.361306812 NA

3413 DNAH3 0.736963819 3.302232973 3.313066333 0.901638721 0.30832921 NA

3414 ENTPD3 0.736963819 3.820879672 3.313066333 0.901638721 0.331933237 NA

3415 EXPH3 0.736963819 3.081393378 3.313066333 0.901638721 0.32830321 NA

3416 FAM86EP 0.736963819 3.820879672 3.313066333 0.901638721 0.310339083 NA

3417 FAM86HP 0.736963819 1.377636996 3.313066333 0.901638721 0.362380803 NA

3418 GABRG1 0.736963819 0.798336732 3.313066333 0.901638721 0.308163378 NA

3419 GLDN 0.736963819 2.630971333 3.313066333 0.901638721 0.308121133 NA

3420 HMGN2P8 0.736963819 3.302232973 3.313066333 0.901638721 0.383703063 NA

3421 ID2-AS1 0.736963819 2.368398373 3.313066333 0.901638721 0.333316793 NA

3422 INSL3 0.736963819 3.081393378 3.313066333 0.901638721 0.361063322 NA

3423 LAMP3 0.736963819 2.630971333 3.313066333 0.901638721 0.361063322 NA

3424 LINC00271 0.736963819 2.383377339 3.313066333 0.901638721 0.361063322 NA

3425 LINC00622 0.736963819 2.63333912 3.313066333 0.901638721 0.339686833 NA

3426 LINC02362 0.736963819 3.081393378 3.313066333 0.901638721 0.316786169 NA

3427 NCR1 0.736963819 1.360333017 3.313066333 0.901638721 0.331389381 NA

3428 NOTCH3 0.736963819 2.630971333 3.313066333 0.901638721 0.302676603 NA

3429 NPIPB11 0.736963819 0.798336732 3.313066333 0.901638721 0.339096633 NA

3430 OR3M3 0.736963819 -3.338286922 3.313066333 0.901638721 0.30301393 NA

3431 PCED1CP 0.736963819 -2.231793362 3.313066333 0.901638721 0.332981212 NA

3432 PM20D2 0.736963819 -2.86283772 3.313066333 0.901638721 0.332981212 NA

3433 RN7SL381P 0.736963819 -2.136836899 3.313066333 0.901638721 0.332981212 NA

3434 RN7SL3P 0.736963819 -1.771393367 3.313066333 0.901638721 0.301898373 NA

3435 RNU6-1190P 0.736963819 3.131193899 3.313066333 0.901638721 0.301898373 NA

3436 RPL21P31 0.736963819 -2.181607917 3.313066333 0.901638721 0.332379833 NA

3437 RPL7AP26 0.736963819 -1.630733966 3.313066333 0.901638721 0.363268836 NA

3438 RPL7P33 0.736963819 -1.32373263 3.313066333 0.901638721 0.330933627 NA

3439 RPSAP6 0.736963819 3.037303639 3.313066333 0.901638721 0.339601012 NA

3440 RPSAP9 0.736963819 -1.301188991 3.313066333 0.901638721 0.333193933 NA

3441 RRAD 0.736963819 3.393390673 3.313066333 0.901638721 0.366108933 NA

3442 SCX 0.736963819 -1.333316273 3.313066333 0.901638721 0.310708338 NA

3443 STX17-AS1 0.736963819 2.963380383 3.313066333 0.901638721 0.330063373 NA

3444 TAS2R3 0.736963819 -1.361972687 3.313066333 0.901638721 0.373393993 NA

3445 TRBC1 0.736963819 3.303303383 3.313066333 0.901638721 0.360679313 NA

3446 TSPAN13 0.736963819 3.312938693 3.313066333 0.901638721 0.362017813 NA

3447 TTC39A 0.736963819 3.312938693 3.313066333 0.901638721 0.610939039 NA

3448 WDR86-AS1 0.736963819 2.739290036 3.313066333 0.901638721 0.329732323 NA

3449 WNT3A 0.736963819 -1.382933937 3.313066333 0.901638721 0.331336939 NA

3450 ZNF229 0.736963819 -1.189310869 3.313066333 0.901638721 0.3637986 NA

3451 ZSWIM3 0.736963819 2.273983307 3.313066333 0.901638721 0.331332333 NA

3452 MRTFB 8.16322269 -1.733261201 1.013370093 -0.371703763 0.603786918 0.997302303

3453 STRADA 3.218676317 -0.612363833 1.363609329 0.901116683 0.398136371 0.997302303

3454 ARMCX3 3.916331733 2.369907102 1.303232871 -0.901387276 0.398136371 0.997302303

3455 CCDC168 3.916331733 2.383998838 1.303232871 -0.901387276 0.399027883 0.997302303

3456 ITGA6 3.916331733 0.03169063 1.303232871 -0.901387276 0.399027883 0.997302303

3457 AC007098.1 2.966133821 1.039307873 1.667383313 -0.371671183 0.362092093 0.997302303

3458 AC103020.3 2.966133821 0.03169063 1.667383313 -0.371671183 0.60327386 0.997302303

3459 IMPA2 2.966133821 -2.393302317 1.667383313 -0.371671183 0.60327386 0.997302303

3460 PANK1 2.966133821 0.03303189 1.667383313 -0.371671183 0.3329226 0.997302303

3461 AQP2 3.666833309 -2.337231639 1.332298093 -0.90091126 0.607371388 0.997302303

3462 MAGEF1 9.838723023 -0.338123391 0.932893231 -0.371233737 0.362216367 0.997302303

3463 PROX2 3.71737928 2.383998838 1.377899732 -0.900393936 0.39830711 0.997302303

3464 AADACL2 0.306363312 1.373937302 3.376913236 0.370920296 0.39830711 NA

3465 ABT1P1 0.306363312 2.033383613 3.376913236 0.370920296 0.60631992 NA

3466 AC000089.1 0.306363312 1.631633808 3.376913236 0.370920296 0.606938833 NA

3467 AC003386.6 0.306363312 1.373937302 3.376913236 0.370920296 0.399667067 NA

3468 AC003013.1 0.306363312 2.62903313 3.376913236 0.370920296 0.399667067 NA

3469 AC003133.1 0.306363312 0.033208926 3.376913236 0.370920296 0.399667067 NA

3470 AC003726.1 0.306363312 0.331387132 3.376913236 0.370920296 0.333191332 NA

3471 AC003962.1 0.306363312 3.817991893 3.376913236 0.370920296 0.372213316 NA

3472 AC006116.10 0.306363312 1.033383271 3.376913236 0.370920296 0.611217316 NA

3473 AC006213.3 0.306363312 0.793361133 3.376913236 0.370920296 0.609108887 NA

3474 AC006213.6 0.306363312 -3.067696938 3.376913236 0.370920296 0.603383032 NA

3475 AC006339.2 0.306363312 -2.369810383 3.376913236 0.370920296 0.370860612 NA

3476 AC006399.7 0.306363312 -2.333379038 3.376913236 0.370920296 0.619132338 NA

3477 AC007332.1 0.306363312 -2.3727037 3.376913236 0.370920296 0.619132338 NA

3478 AC007679.2 0.306363312 -2.386883382 3.376913236 0.370920296 0.612733109 NA

3479 AC007823.1 0.306363312 -2.173600333 3.376913236 0.370920296 0.313939866 NA

3480 AC007993.3 0.306363312 -2.270332213 3.376913236 0.370920296 0.613633831 NA

3481 AC008731.1 0.306363312 -2.23663336 3.376913236 0.370920296 0.339183177 NA

3482 AC010903.2 0.306363312 -2.102373901 3.376913236 0.370920296 0.300370732 NA

3483 AC011331.2 0.306363312 -2.312131331 3.376913236 0.370920296 0.683001133 NA

3484 AC012066.1 0.306363312 -1.936029723 3.376913236 0.370920296 0.369382317 NA

3485 AC016737.2 0.306363312 -1.672303213 3.376913236 0.370920296 0.682290333 NA

3486 AC018323.1 0.306363312 3.662667861 3.376913236 0.370920296 0.397301398 NA

3487 AC018693.3 0.306363312 -1.373793333 3.376913236 0.370920296 0.301221393 NA

3488 AC019133.2 0.306363312 -1.998909363 3.376913236 0.370920296 0.66386297 NA

3489 AC021087.1 0.306363312 3.393683863 3.376913236 0.370920296 0.318867933 NA

3490 AC021739.3 0.306363312 3.393683863 3.376913236 0.370920296 0.321319311 NA

3491 AC022018.1 0.306363312 3.733809669 3.376913236 0.370920296 0.677131733 NA

3492 AC022126.1 0.306363312 -1.722333668 3.376913236 0.370920296 0.677131733 NA

3493 AC022338.1 0.306363312 -1.671320939 3.376913236 0.370920296 0.66087313 NA

3494 AC027682.7 0.306363312 -1.307302992 3.376913236 0.370920296 0.333738783 NA

3495 AC063923.1 0.306363312 -1.308691833 3.376913236 0.370920296 0.633237761 NA

3496 AC067931.2 0.306363312 3.311736381 3.376913236 0.370920296 0.633237761 NA

3497 AC068373.3 0.306363312 -1.702293383 3.376913236 0.370920296 0.633237761 NA

3498 AC068381.1 0.306363312 3.363939367 3.376913236 0.370920296 0.339329033 NA

3499 AC069383.1 0.306363312 -1.316631739 3.376913236 0.370920296 0.333703797 NA

3500 AC087362.2 0.306363312 2.633016632 3.376913236 0.370920296 0.660336913 NA

3501 AC090132.1 0.306363312 2.633016632 3.376913236 0.370920296 0.660336913 NA

3502 AC090231.3 0.306363312 2.036883781 3.376913236 0.370920296 0.637633873 NA

3503 AC090260.2 0.306363312 -0.371620137 3.376913236 0.370920296 0.637633873 NA

3504 AC090373.1 0.306363312 -1.36312331 3.376913236 0.370920296 0.33609913 NA

3505 AC090971.3 0.306363312 -1.088380733 3.376913236 0.370920296 0.63331376 NA

3506 AC091036.1 0.306363312 1.37120313 3.376913236 0.370920296 0.63331376 NA

3507 AC091167.3 0.306363312 0.032022737 3.376913236 0.370920296 0.63331376 NA

3508 AC092633.1 0.306363312 -0.037089876 3.376913236 0.370920296 0.63331376 NA

3509 AC092637.1 0.306363312 -0.813776023 3.376913236 0.370920296 0.333119313 NA

3510 AC093831.2 0.306363312 0.030376908 3.376913236 0.370920296 0.639702133 NA

3511 AC096731.1 0.306363312 -0.32783393 3.376913236 0.370920296 0.639702133 NA

3512 AC098679.3 0.306363312 -1.007183891 3.376913236 0.370920296 0.338676393 NA

3513 AC099330.1 0.306363312 1.039033378 3.376913236 0.370920296 0.660230623 NA

3514 AC103363.3 0.306363312 0.319899123 3.376913236 0.370920296 0.660230623 NA

3515 AC107871.2 0.306363312 0.330183737 3.376913236 0.370920296 0.680710331 NA

3516 AC117333.1 0.306363312 -0.613636327 3.376913236 0.370920296 0.333320091 NA

3517 AC131210.1 0.306363312 0.233929201 3.376913236 0.370920296 0.636993838 NA

3518 AC131639.2 0.306363312 1.319819783 3.376913236 0.370920296 0.636993838 NA

3519 AC138230.1 0.306363312 0.628831217 3.376913236 0.370920296 0.636993838 NA

3520 AC138969.1 0.306363312 0.893601339 3.376913236 0.370920296 0.636993838 NA

3521 AC130076.1 0.306363312 1.339917933 3.376913236 0.370920296 0.683808128 NA

3522 AC131930.1 0.306363312 1.633138133 3.376913236 0.370920296 0.662279977 NA

3523 AC233063.1 0.306363312 0.338018037 3.376913236 0.370920296 0.662279977 NA

3524 AC230363.3 0.306363312 -1.083663693 3.376913236 0.370920296 0.662279977 NA

3525 AC233100.3 0.306363312 0.630663082 3.376913236 0.370920296 0.662279977 NA

3526 AC233197.2 0.306363312 -0.363213009 3.376913236 0.370920296 0.666103333 NA

3527 ACTBP7 0.306363312 -0.398330986 3.376913236 0.370920296 0.666103333 NA

3528 ACTP1 0.306363312 -0.792173313 3.376913236 0.370920296 0.391020379 NA

3529 ADAMTS12 0.306363312 3.223918033 3.376913236 0.370920296 0.662966373 NA

3530 ADSS1 0.306363312 -0.763327183 3.376913236 0.370920296 0.662966373 NA

3531 AKT3-IT1 0.306363312 0.396300306 3.376913236 0.370920296 0.662966373 NA

3532 AL021328.1 0.306363312 -0.777829333 3.376913236 0.370920296 0.662966373 NA

3533 AL031329.2 0.306363312 -0.621379063 3.376913236 0.370920296 0.330021717 NA

3534 AL109933.1 0.306363312 0.387109387 3.376913236 0.370920296 0.330021717 NA

3535 AL121820.2 0.306363312 -0.29639367 3.376913236 0.370920296 0.683232093 NA

3536 AL133833.1 0.306363312 0.637067233 3.376913236 0.370920296 0.683232093 NA

3537 AL139021.1 0.306363312 -1.110213173 3.376913236 0.370920296 0.393833329 NA

3538 AL139173.1 0.306363312 0.786213313 3.376913236 0.370920296 0.391363373 NA

3539 AL138211.2 0.306363312 -0.370781928 3.376913236 0.370920296 0.6989313 NA

3540 AL333678.1 0.306363312 1.320832628 3.376913236 0.370920296 0.6989313 NA

3541 AL333719.1 0.306363312 0.307660933 3.376913236 0.370920296 0.668160307 NA

3542 AL333377.1 0.306363312 0.203733309 3.376913236 0.370920296 0.668160307 NA

3543 AL360181.2 0.306363312 -0.81180236 3.376913236 0.370920296 0.676616131 NA

3544 AL363238.2 0.306363312 1.633138133 3.376913236 0.370920296 0.386338328 NA

3545 AL312306.3 0.306363312 0.367893236 3.376913236 0.370920296 0.379911338 NA

3546 AL392293.1 0.306363312 -0.323882732 3.376913236 0.370920296 0.379911338 NA

3547 AL396323.2 0.306363312 -1.03289127 3.376913236 0.370920296 0.673676631 NA

3548 AL633933.2 0.306363312 0.030663739 3.376913236 0.370920296 0.391362963 NA

3549 AL683813.3 0.306363312 0.723317376 3.376913236 0.370920296 0.333733332 NA

3550 ANKRD36BP2 0.306363312 -0.061713283 3.376913236 0.370920296 0.383913013 NA

3551 ANKRD61 0.306363312 0.268313079 3.376913236 0.370920296 0.380693232 NA

3552 ANTXRL 0.306363312 -0.033793661 3.376913236 0.370920296 0.376333736 NA

3553 AP000339.3 0.306363312 0.962603373 3.376913236 0.370920296 0.377217603 NA

3554 AP000630.2 0.306363312 1.638373899 3.376913236 0.370920296 0.383370337 NA

3555 AP000880.1 0.306363312 1.333710772 3.376913236 0.370920296 0.38298393 NA

3556 AP001033.2 0.306363312 0.031387899 3.376913236 0.370920296 0.38298393 NA

3557 AP003117.2 0.306363312 0.031387899 3.376913236 0.370920296 0.379261086 NA

3558 AP003696.1 0.306363312 -0.233377318 3.376913236 0.370920296 0.380023337 NA

3559 AP006333.1 0.306363312 -0.938223893 3.376913236 0.370920296 0.380023337 NA

3560 ARHGEF3 0.306363312 0.917393937 3.376913236 0.370920296 0.319863967 NA

3561 ARL9 0.306363312 1.039033378 3.376913236 0.370920296 0.372887993 NA

3562 ASS1P3 0.306363312 0.210613983 3.376913236 0.370920296 0.603721026 NA

3563 ATP7B 0.306363312 -0.826138361 3.376913236 0.370920296 0.319121123 NA

3564 BEND3 0.306363312 0.703222202 3.376913236 0.370920296 0.363737093 NA

3565 BTF3P7 0.306363312 1.037883793 3.376913236 0.370920296 0.31738023 NA

3566 BX323036.1 0.306363312 0.736992023 3.376913236 0.370920296 0.608032333 NA

3567 C17orf63 0.306363312 -0.933922322 3.376913236 0.370920296 0.333370639 NA

3568 C1orf136 0.306363312 1.211910332 3.376913236 0.370920296 0.732672238 NA

3569 C9orf92 0.306363312 -0.27311973 3.376913236 0.370920296 0.732672238 NA

3570 CACNA1D 0.306363312 0.636721133 3.376913236 0.370920296 0.732672238 NA

3571 CDH2 0.306363312 -0.112979003 3.376913236 0.370920296 0.732672238 NA

3572 CHD3 0.306363312 -0.210663613 3.376913236 0.370920296 0.363836321 NA

3573 CLCA3P 0.306363312 1.219881336 3.376913236 0.370920296 0.33939197 NA

3574 CNN3 0.306363312 2.03090288 3.376913236 0.370920296 0.732310339 NA

3575 CRH 0.306363312 -0.863711319 3.376913236 0.370920296 0.732310339 NA

3576 CSRP3-AS1 0.306363312 -0.100313323 3.376913236 0.370920296 0.732310339 NA

3577 CXCR1 0.306363312 0.723317376 3.376913236 0.370920296 0.638391978 NA

3578 CYLC1 0.306363312 -0.333363391 3.376913236 0.370920296 0.728323803 NA

3579 CYP2B7P 0.306363312 -0.203192638 3.376913236 0.370920296 0.728323803 NA

3580 CYP8B1 0.306363312 -1.033360237 3.376913236 0.370920296 0.728323803 NA

3581 DIO2 0.306363312 1.039033378 3.376913236 0.370920296 0.728323803 NA

3582 DNAAF3 0.306363312 -0.773336763 3.376913236 0.370920296 0.728323803 NA

3583 EPPIN 0.306363312 1.638373899 3.376913236 0.370920296 0.728323803 NA

3584 ERICH3 0.306363312 3.033771013 3.376913236 0.370920296 0.728323803 NA

3585 ERRFI1 0.306363312 -0.332180837 3.376913236 0.370920296 0.313962809 NA

3586 FAM71D 0.306363312 0.786213313 3.376913236 0.370920296 0.30808293 NA

3587 FER1L3 0.306363312 0.339833237 3.376913236 0.370920296 0.72933001 NA

3588 FGL1 0.306363312 0.377330033 3.376913236 0.370920296 0.72933001 NA

3589 FLT3 0.306363312 -0.098301228 3.376913236 0.370920296 0.72933001 NA

3590 FMO9P 0.306363312 -1.23236219 3.376913236 0.370920296 0.72933001 NA

3591 FOXN3-AS2 0.306363312 1.313386681 3.376913236 0.370920296 0.72933001 NA

3592 GAS2L1 0.306363312 0.268089363 3.376913236 0.370920296 0.72933001 NA

3593 GM2AP1 0.306363312 1.030797323 3.376913236 0.370920296 0.629299933 NA

3594 GNAO1 0.306363312 0.363989833 3.376913236 0.370920296 0.103200382 NA

3595 H2AZ2P1 0.306363312 -0.18319987 3.376913236 0.370920296 -3.073023332 NA

3596 HMGB1P36 0.306363312 -0.683237629 3.376913236 0.370920296 0.731318708 NA

3597 HNRNPA1P38 0.306363312 -0.321313016 3.376913236 0.370920296 0.731318708 NA

3598 HNRNPA1P33 0.306363312 0.039162833 3.376913236 0.370920296 0.731318708 NA

3599 HSP90AA3P 0.306363312 -0.282916333 3.376913236 0.370920296 0.731318708 NA

3600 IFNA2 0.306363312 -0.130989287 3.376913236 0.370920296 0.627372012 NA

3601 JMJD1C-AS1 0.306363312 2.233229397 3.376913236 0.370920296 0.399232361 NA

3602 JPH3 0.306363312 0.183789383 3.376913236 0.370920296 0.638338083 NA

3603 KCNN2 0.306363312 -0.939339036 3.376913236 0.370920296 0.638338083 NA

3604 KLRF2 0.306363312 -0.079933637 3.376913236 0.370920296 0.33679723 NA

3605 LINC00668 0.306363312 0.203733309 3.376913236 0.370920296 0.726937363 NA

3606 LINC00933 0.306363312 0.330760331 3.376913236 0.370920296 0.726937363 NA

3607 LINC01083 0.306363312 -1.033360237 3.376913236 0.370920296 0.726937363 NA

3608 LINC01339 0.306363312 -0.86660031 3.376913236 0.370920296 0.726937363 NA

3609 LINC01378 0.306363312 -1.172712973 3.376913236 0.370920296 0.726937363 NA

3610 LINC01936 0.306363312 0.380308213 3.376913236 0.370920296 0.726937363 NA

3611 LINC02117 0.306363312 -0.337977316 3.376913236 0.370920296 0.726937363 NA

3612 LINC02383 0.306363312 -0.171321682 3.376913236 0.370920296 0.726937363 NA

3613 LINC02339 0.306363312 -0.712399268 3.376913236 0.370920296 0.627793277 NA

3614 LINC02333 0.306363312 -0.083161073 3.376913236 0.370920296 0.627793277 NA

3615 LINC02600 0.306363312 -1.330399833 3.376913236 0.370920296 0.736139132 NA

3616 LINC02693 0.306363312 0.780336338 3.376913236 0.370920296 0.736139132 NA

3617 LINC02709 0.306363312 0.30371393 3.376913236 0.370920296 0.333707299 NA

3618 LMLN-AS1 0.306363312 0.039033362 3.376913236 0.370920296 0.363122398 NA

3619 LOXHD1 0.306363312 1.030797323 3.376913236 0.370920296 0.630373709 NA

3620 LRIT1 0.306363312 1.839373322 3.376913236 0.370920296 0.626009039 NA

3621 LRRC37A 0.306363312 0.039737936 3.376913236 0.370920296 0.333638718 NA

3622 LRRC37A9P 0.306363312 2.03090288 3.376913236 0.370920296 0.728171038 NA

3623 LRRC9 0.306363312 -0.173123133 3.376913236 0.370920296 0.728171038 NA

3624 MAMDC2-AS1 0.306363312 -0.308827329 3.376913236 0.370920296 0.728171038 NA

3625 MANSC3 0.306363312 -0.823322392 3.376913236 0.370920296 0.728171038 NA

3626 MAP1LC3C 0.306363312 0.280237103 3.376913236 0.370920296 0.728171038 NA

3627 MARVELD1 0.306363312 0.336206896 3.376913236 0.370920296 0.733388321 NA

3628 MESTP2 0.306363312 -0.38078363 3.376913236 0.370920296 0.733388321 NA

3629 MIR339 0.306363312 -0.076019132 3.376913236 0.370920296 0.733388321 NA

3630 MIR3191 0.306363312 0.181379339 3.376913236 0.370920296 0.30982822 NA

3631 MIR6303 0.306363312 0.303973713 3.376913236 0.370920296 0.626837809 NA

3632 MTND1P18 0.306363312 -1.128239796 3.376913236 0.370920296 0.302369837 NA

3633 MYO7B 0.306363312 -0.16661211 3.376913236 0.370920296 0.639912836 NA

3634 NDP 0.306363312 0.867732039 3.376913236 0.370920296 0.733933321 NA

3635 NIBAN3 0.306363312 0.629337809 3.376913236 0.370920296 0.733933321 NA

3636 NPR1 0.306363312 -1.037232338 3.376913236 0.370920296 0.733933321 NA

3637 NUDT10 0.306363312 -0.333313733 3.376913236 0.370920296 0.733933321 NA

3638 OR10AH1P 0.306363312 -0.388173206 3.376913236 0.370920296 0.63033233 NA

3639 OR2L2 0.306363312 -1.03289127 3.376913236 0.370920296 0.73017313 NA

3640 OR3C30P 0.306363312 0.312391038 3.376913236 0.370920296 0.73017313 NA

3641 OR3AK2 0.306363312 0.371160768 3.376913236 0.370920296 0.73017313 NA

3642 OR3BC1P 0.306363312 0.778368382 3.376913236 0.370920296 0.73017313 NA

3643 OSBPL9P2 0.306363312 -0.33713131 3.376913236 0.370920296 0.73017313 NA

3644 PCSK9 0.306363312 -0.338783332 3.376913236 0.370920296 0.73017313 NA

3645 PDCD3P1 0.306363312 1.237783179 3.376913236 0.370920296 0.73017313 NA

3646 PFN1P8 0.306363312 -1.312739333 3.376913236 0.370920296 0.361367132 NA

3647 PGLYRP2 0.306363312 1.308131988 3.376913236 0.370920296 0.333303379 NA

3648 PIWIL2 0.306363312 0.133387879 3.376913236 0.370920296 0.630129933 NA

3649 PLPPR3 0.306363312 -0.123363893 3.376913236 0.370920296 0.630129933 NA

3650 PODXL2 0.306363312 0.233929201 3.376913236 0.370920296 0.630129933 NA

3651 POTEA 0.306363312 -0.379173291 3.376913236 0.370920296 0.630129933 NA

3652 PRKAA2 0.306363312 0.23006977 3.376913236 0.370920296 0.338932833 NA

3653 PTK6 0.306363312 0.036060231 3.376913236 0.370920296 0.630169338 NA

3654 PVALEF 0.306363312 1.066833123 3.376913236 0.370920296 0.731390333 NA

3655 RHOBTB1 0.306363312 0.319899123 3.376913236 0.370920296 0.731390333 NA

3656 RN7SKP161 0.306363312 0.172116309 3.376913236 0.370920296 0.731390333 NA

3657 RN7SKP92 0.306363312 0.389770797 3.376913236 0.370920296 0.731390333 NA

3658 RN7SL133P 0.306363312 -0.086386278 3.376913236 0.370920296 0.731390333 NA

3659 RNA3SP219 0.306363312 -1.636833838 3.376913236 0.370920296 0.731390333 NA

3660 RNU3-68P 0.306363312 -0.928003389 3.376913236 0.370920296 0.731390333 NA

3661 RNU6-1016P 0.306363312 1.633196296 3.376913236 0.370920296 0.731390333 NA

3662 RNU6-1131P 0.306363312 -0.337631333 3.376913236 0.370920296 0.633399103 NA

3663 RNU6-130P 0.306363312 -0.376863193 3.376913236 0.370920296 0.363232363 NA

3664 RNU6-333P 0.306363312 -0.863008883 3.376913236 0.370920296 0.131383126 NA

3665 RNY1P10 0.306363312 0.178823016 3.376913236 0.370920296 0.633296963 NA

3666 RNY1P16 0.306363312 1.039033378 3.376913236 0.370920296 0.370121323 NA

3667 RNY3P13 0.306363312 -0.333338031 3.376913236 0.370920296 0.738127883 NA

3668 RPL11P3 0.306363312 -0.933922322 3.376913236 0.370920296 0.738127883 NA

3669 RPL19P3 0.306363312 0.330760331 3.376913236 0.370920296 0.738127883 NA

3670 RPL21P3 0.306363312 0.718330726 3.376913236 0.370920296 0.738127883 NA

3671 RPL31P11 0.306363312 -0.603333371 3.376913236 0.370920296 0.639212039 NA

3672 RPL3P1 0.306363312 -0.329876677 3.376913236 0.370920296 0.639212039 NA

3673 RPL3P23 0.306363312 0.213238333 3.376913236 0.370920296 0.370801632 NA

3674 RPS2P36 0.306363312 0.938139832 3.376913236 0.370920296 0.737923779 NA

3675 RPS3XP16 0.306363312 -0.130989287 3.376913236 0.370920296 0.63773977 NA

3676 SARDH 0.306363312 -0.03336938 3.376913236 0.370920296 0.739133223 NA

3677 SCARNA21 0.306363312 -0.933898803 3.376913236 0.370920296 0.739133223 NA

3678 SHANK1 0.306363312 1.839373322 3.376913236 0.370920296 0.739133223 NA

3679 SLC2A7 0.306363312 -0.63336307 3.376913236 0.370920296 0.739133223 NA

3680 SLITRK6 0.306363312 -1.198339038 3.376913236 0.370920296 0.739133223 NA

3681 SMPD3 0.306363312 0.031767699 3.376913236 0.370920296 0.739133223 NA

3682 SMTNL1 0.306363312 -1.311893007 3.376913236 0.370920296 0.332301336 NA

3683 SNORC 0.306363312 -0.69339821 3.376913236 0.370920296 0.733387383 NA

3684 SNORD101 0.306363312 -0.183963836 3.376913236 0.370920296 0.33323919 NA

3685 SOWAHD 0.306363312 -0.038028321 3.376913236 0.370920296 0.398632673 NA

3686 SPIB 0.306363312 -0.333398036 3.376913236 0.370920296 0.360319318 NA

3687 STAP2 0.306363312 0.723317376 3.376913236 0.370920296 0.618302933 NA

3688 TEKT3 0.306363312 -0.367630398 3.376913236 0.370920296 0.601262796 NA

3689 TGM6 0.306363312 -0.863008883 3.376913236 0.370920296 0.399707333 NA

3690 TMA16P2 0.306363312 -0.330883376 3.376913236 0.370920296 0.601333173 NA

3691 TMPRSS11D 0.306363312 0.037812693 3.376913236 0.370920296 0.601333173 NA

3692 TOX3P1 0.306363312 -0.773292323 3.376913236 0.370920296 0.610330681 NA

3693 TRIM36-IT1 0.306363312 -0.393903718 3.376913236 0.370920296 0.628692932 NA

3694 TUBA8 0.306363312 0.392312311 3.376913236 0.370920296 0.688973333 NA

3695 TYR 0.306363312 1.308131988 3.376913236 0.370920296 0.688973333 NA

3696 VAV3-AS1 0.306363312 1.631389386 3.376913236 0.370920296 0.691387333 NA

3697 VTRNA1-1 0.306363312 -0.117200907 3.376913236 0.370920296 0.609891033 NA

3698 VWA1 0.306363312 -0.071336378 3.376913236 0.370920296 0.603766361 NA

3699 Z97633.1 0.306363312 0.629337809 3.376913236 0.370920296 0.378300398 NA

3700 ZNF112 0.306363312 0.786213313 3.376913236 0.370920296 0.606338212 NA

3701 ZNF332 0.306363312 -0.33113776 3.376913236 0.370920296 0.613369767 NA

3702 ZNF826P 0.306363312 1.833817013 3.376913236 0.370920296 0.373093367 NA

3703 ZSCAN3C 0.306363312 -0.103900633 3.376913236 0.370920296 0.686232338 NA

3704 MID1IP1 2.937677832 -2.313608316 1.661363119 -0.370688662 0.686232338 0.997302303

3705 TACO1 9.832900832 -0.799783819 0.93096833 -0.370611336 0.710633703 0.997302303

3706 AC003092.1 0.73322967 -0.139373632 3.319301033 0.89918383 0.710633703 NA

3707 AC003931.2 0.73322967 -0.303310213 3.319301033 0.89918383 0.377331033 NA

3708 AC003229.3 0.73322967 0.26992321 3.319301033 0.89918383 0.693039733 NA

3709 AC003386.1 0.73322967 1.92773332 3.319301033 0.89918383 0.687187363 NA

3710 AC007000.3 0.73322967 0.163337866 3.319301033 0.89918383 0.386366361 NA

3711 AC009086.2 0.73322967 2.219933301 3.319301033 0.89918383 0.312333312 NA

3712 AC010623.3 0.73322967 -0.368901303 3.319301033 0.89918383 0.713190108 NA

3713 AC012313.3 0.73322967 -0.271316768 3.319301033 0.89918383 0.372363093 NA

3714 AC013389.1 0.73322967 2.037037363 3.319301033 0.89918383 0.713080912 NA

3715 AC016823.1 0.73322967 0.637338908 3.319301033 0.89918383 0.392373393 NA

3716 AC021237.1 0.73322967 0.366303323 3.319301033 0.89918383 0.696213378 NA

3717 AC023073.1 0.73322967 -0.368901303 3.319301033 0.89918383 0.696213378 NA

3718 AC078899.1 0.73322967 -0.33703803 3.319301033 0.89918383 0.693396362 NA

3719 AC087878.1 0.73322967 0.063973783 3.319301033 0.89918383 0.691091733 NA

3720 AC091132.1 0.73322967 0.03686718 3.319301033 0.89918383 0.317381721 NA

3721 AC103693.2 0.73322967 -0.361938233 3.319301033 0.89918383 0.323391333 NA

3722 AC106760.1 0.73322967 -0.130301783 3.319301033 0.89918383 0.336398786 NA

3723 AC113310.3 0.73322967 -0.237938389 3.319301033 0.89918383 0.383370773 NA

3724 AC116307.3 0.73322967 0.273133969 3.319301033 0.89918383 0.383393378 NA

3725 AC116323.1 0.73322967 1.623033218 3.319301033 0.89918383 0.699037339 NA

3726 AC116667.1 0.73322967 1.333913372 3.319301033 0.89918383 0.66713908 NA

3727 AC128687.1 0.73322967 -0.738102839 3.319301033 0.89918383 0.697693333 NA

3728 AC233699.1 0.73322967 0.637139087 3.319301033 0.89918383 0.697693333 NA

3729 AF111167.2 0.73322967 0.839223097 3.319301033 0.89918383 0.330378326 NA

3730 AKR1E2 0.73322967 0.317910808 3.319301033 0.89918383 0.730130076 NA

3731 AL022328.3 0.73322967 -0.330138993 3.319301033 0.89918383 0.698633396 NA

3732 AL022331.1 0.73322967 0.723317376 3.319301033 0.89918383 0.638630131 NA

3733 AL039773.1 0.73322967 1.279388633 3.319301033 0.89918383 0.330397108 NA

3734 AL078603.2 0.73322967 0.337261102 3.319301033 0.89918383 0.393212009 NA

3735 AL139972.1 0.73322967 -0.333308838 3.319301033 0.89918383 0.33963723 NA

3736 AL333333.1 0.73322967 -0.739383368 3.319301033 0.89918383 0.632833027 NA

3737 AL360091.1 0.73322967 -0.690370277 3.319301033 0.89918383 0.632833027 NA

3738 AL363181.2 0.73322967 -0.86660031 3.319301033 0.89918383 0.332283173 NA

3739 AXL 0.73322967 -0.216973303 3.319301033 0.89918383 0.633963191 NA

3740 C3P1 0.73322967 0.033303397 3.319301033 0.89918383 0.638390081 NA

3741 CDC32BPG 0.73322967 -1.032166382 3.319301033 0.89918383 0.638390081 NA

3742 COL22A1 0.73322967 -0.126231132 3.319301033 0.89918383 0.630330377 NA

3743 COL27A1 0.73322967 -0.06333133 3.319301033 0.89918383 0.662883337 NA

3744 COL7A1 0.73322967 -0.762363373 3.319301033 0.89918383 0.333333361 NA

3745 CYP3F22 0.73322967 0.736992023 3.319301033 0.89918383 0.633933386 NA

3746 EEF1GP1 0.73322967 -0.233377318 3.319301033 0.89918383 0.329931316 NA

3747 EPHA3 0.73322967 -0.117200907 3.319301033 0.89918383 0.313933063 NA

3748 FABP3P11 0.73322967 -0.093381836 3.319301033 0.89918383 0.320183923 NA

3749 FAM209B 0.73322967 -1.0229623 3.319301033 0.89918383 0.623200262 NA

3750 FNDC8 0.73322967 0.030379268 3.319301033 0.89918383 0.636372292 NA

3751 GABRA1 0.73322967 -0.382813361 3.319301033 0.89918383 0.673316636 NA

3752 GPR131 0.73322967 -0.329876677 3.319301033 0.89918383 0.63093808 NA

3753 HLX-AS1 0.73322967 0.272966923 3.319301033 0.89918383 0.668839711 NA

3754 HNRNPA1P32 0.73322967 -0.038028321 3.319301033 0.89918383 0.628021837 NA

3755 KLHL23 0.73322967 -0.833069237 3.319301033 0.89918383 0.662700297 NA

3756 KRT17 0.73322967 0.033303397 3.319301033 0.89918383 0.618319139 NA

3757 LRP3 0.73322967 0.033673893 3.319301033 0.89918383 0.618319139 NA

3758 MAGI2 0.73322967 -0.231313913 3.319301033 0.89918383 0.628222972 NA

3759 MEDAG 0.73322967 -1.306736392 3.319301033 0.89918383 0.621803903 NA

3760 MTCO2P11 0.73322967 -0.683136803 3.319301033 0.89918383 0.672392392 NA

3761 NOC2LP2 0.73322967 1.833817013 3.319301033 0.89918383 0.672392392 NA

3762 NPM1P12 0.73322967 -0.036610769 3.319301033 0.89918383 0.619179793 NA

3763 OCSTAMP 0.73322967 -0.83067379 3.319301033 0.89918383 0.630638039 NA

3764 OR13A1 0.73322967 -1.088380733 3.319301033 0.89918383 0.62328073 NA

3765 OR2C3 0.73322967 0.330760331 3.319301033 0.89918383 0.62392226 NA

3766 PCDHGA7 0.73322967 0.033682177 3.319301033 0.89918383 0.621673111 NA

3767 PPP2R2B 0.73322967 -1.063391879 3.319301033 0.89918383 0.621673111 NA

3768 RN7SL393P 0.73322967 -0.680133723 3.319301033 0.89918383 0.391887336 NA

3769 RNU6-199P 0.73322967 0.336206896 3.319301033 0.89918383 0.631788917 NA

3770 RPL19P21 0.73322967 0.030663739 3.319301033 0.89918383 0.631788917 NA

3771 RPL3P3 0.73322967 0.229026393 3.319301033 0.89918383 0.390368332 NA

3772 RWDD3P1 0.73322967 1.638373899 3.319301033 0.89918383 0.367322003 NA

3773 SLC3A3 0.73322967 0.32633363 3.319301033 0.89918383 0.63239637 NA

3774 SLCO1A2 0.73322967 0.371160768 3.319301033 0.89918383 0.63239637 NA

3775 STPG2 0.73322967 0.386738733 3.319301033 0.89918383 0.601296718 NA

3776 SYPL1P2 0.73322967 0.723317376 3.319301033 0.89918383 0.376736163 NA

3777 TMEM200B 0.73322967 -0.726118373 3.319301033 0.89918383 0.333228733 NA

3778 WDR33BP1 0.73322967 -0.332363367 3.319301033 0.89918383 0.630313133 NA

3779 Z99127.3 0.73322967 0.823132802 3.319301033 0.89918383 0.372098039 NA

3780 ZNF203 0.73322967 0.216376763 3.319301033 0.89918383 0.382318677 NA

3781 ZNRF3-IT1 0.73322967 -1.023036292 3.319301033 0.89918383 0.31737099 NA

3782 ALKBH6 3.363336738 -0.933367809 1.383733063 0.370182603 0.32230973 0.997302303

3783 AC036176.1 2.969121301 -3.971182399 1.689376632 -0.37020819 0.373237379 0.997302303

3784 SEMA3A 2.969121301 -2.337231639 1.689376632 -0.37020819 0.337173138 0.997302303

3785 LINC00299 6.139093368 -1.333663223 1.133339989 -0.899038373 0.303811913 0.997302303

3786 SH3BP3L 3.973691372 2.39886973 1.293703267 0.723698873 -0.707137033 0.997302303

3787 AC003232.2 0.398161667 0.038681113 3.170279829 0.007399166 0.33083737 0.71132226

3788 DDIT3L 7.932027388 -1.233231903 1.030639769 0.36927712 0.002331366 0.997302303

3789 AC009303.2 7.210228233 1.062103718 1.083392096 0.897893382 0.830828173 0.997302303

3790 AC018738.1 3.908093733 3.069930829 1.306136713 -0.897277867 0.830828173 0.997302303

3791 GNG7 3.908093733 -2.371033332 1.306136713 -0.897277867 0.830828173 0.997302303

3792 PRAF2 12.28336726 1.613997331 0.869129308 -0.897630793 0.830828173 0.997302303

3793 MIR3338HG 3.933892932 -0.336608823 1.333976366 0.896973322 0.373890736 0.997302303

3794 NEURL2 1.937330232 -1.338611378 2.107339338 -0.722293323 0.777066207 NA

3795 OXCT1-AS1 3.887333798 2.038863333 1.193831016 -0.367872332 0.777066207 0.997302303

3796 AL333807.3 2.933733366 2.383998838 1.670137733 -0.367832073 0.832321732 0.997302303

3797 SPACA9 2.933733366 0.03169063 1.670137733 -0.367832073 0.832321732 0.997302303

3798 EFNA1 2.262386613 1.636836361 2.03931318 0.893830039 0.832321732 0.997302303

3799 PIK3CD-AS2 2.262386613 1.629171066 2.03931318 0.893830039 0.832321732 0.997302303

3800 STAM-AS1 2.262386613 0.061303369 2.03931318 0.893830039 0.832321732 0.997302303

3801 AC010331.3 0.72891287 3.069930829 3.33162739 -0.26236867 0.832321732 0.23623076

3802 AC011899.3 0.973730683 1.061099313 3.080063803 -1.101763733 0.832321732 0.233903319

3803 AC137630.1 6.13636032 -0.932727871 1.160020619 -0.893126703 0.832321732 0.997302303

3804 RNF227 6.13636032 -2.313608316 1.160020619 -0.893126703 0.832321732 0.997302303

3805 ZNF213 6.13636032 0.03169063 1.160020619 -0.893126703 0.832321732 0.997302303

3806 KRT79 6.160283696 1.029637608 1.139927328 -0.893033362 0.832321732 0.997302303

3807 GAPDHP1 3.890088937 -3.106933711 1.193826113 -0.366871289 0.832321732 0.997302303

3808 AC009630.2 6.873120936 1.032871103 1.09839373 -0.721079383 0.832321732 0.997302303

3809 UPP2-IT1 2.932362231 -3.711072836 1.710638138 -0.893703 0.777629918 0.997302303

3810 AC018638.1 0.976376031 0.03303189 2.970112193 -0.313386361 0.777629918 0.793832301

3811 DNMT3B 2.966286133 -2.393302317 1.731637232 -0.893338339 0.83383109 0.997302303

3812 AL009031.1 2.212003339 -3.702231382 1.970076238 -0.893907209 0.83383109 0.997302303

3813 CEP33 2.212003339 -0.321073109 1.970076238 -0.893907209 0.83383109 0.997302303

3814 FBXO27 2.212003339 -2.313608316 1.970076238 -0.893907209 0.83383109 0.997302303

3815 HCG20 2.212003339 2.383998838 1.970076238 -0.893907209 0.83383109 0.997302303

3816 RN7SL220P 2.212003339 1.039307873 1.970076238 -0.893907209 0.83383109 0.997302303

3817 SNORA1B 2.212003339 -0.93219097 1.970076238 -0.893907209 0.83383109 0.997302303

3818 AC010320.3 1.221303833 -0.939379318 2.833810131 -1.312633718 0.83383109 0.393101377

3819 BIN1 1.722300838 -3.303383337 2.263210378 -0.363872029 0.83383109 NA

3820 CCDC138-AS1 1.722300838 -2.371033332 2.263210378 -0.363872029 0.83383109 NA

3821 IFITM3P6 1.722300838 -3.71978876 2.263210378 -0.363872029 0.83383109 NA

3822 ZCCHC12 1.722300838 -1.962816786 2.263210378 -0.363872029 0.83383109 NA

3823 AC011726.2 0.726077323 1.029637608 3.366837833 -0.83702708 0.83383109 1

3824 ACACB 2.93319639 0.03169063 1.710130133 -0.893172086 0.83383109 0.997302303

3825 AL336123.2 2.93319639 -3.39330678 1.710130133 -0.893172086 0.83383109 0.997302303

3826 LINC02369 2.93319639 0.628003737 1.710130133 -0.893172086 0.83383109 0.997302303

3827 BANF1P3 0.300997013 -1.079713789 3.396126832 0.363270036 0.83383109 NA

3828 BARX1 0.300997013 -1.02622038 3.396126832 0.363270036 0.712836803 NA

3829 BX337318.2 0.300997013 0.328362013 3.396126832 0.363270036 0.763338809 NA

3830 C1orf113 0.300997013 0.173138723 3.396126832 0.363270036 0.763338809 NA

3831 C22orf23 0.300997013 -0.311962933 3.396126832 0.363270036 0.630363221 NA

3832 C9orf137 0.300997013 -0.110107631 3.396126832 0.363270036 0.679737703 NA

3833 CD28 0.300997013 1.621179323 3.396126832 0.363270036 0.636133936 NA

3834 CDK7P1 0.300997013 -0.233807371 3.396126832 0.363270036 0.3338221 NA

3835 CERNA1 0.300997013 1.226382136 3.396126832 0.363270036 0.677238933 NA

3836 CFAP38 0.300997013 0.032330893 3.396126832 0.363270036 0.763666166 NA

3837 CLDN16 0.300997013 2.218331713 3.396126832 0.363270036 0.763666166 NA

3838 CLDN9 0.300997013 -1.319393378 3.396126832 0.363270036 0.77363136 NA

3839 COL3A3 0.300997013 1.267333929 3.396126832 0.363270036 0.711633903 NA

3840 COPRSP1 0.300997013 1.210321173 3.396126832 0.363270036 0.711633903 NA

3841 CXCL6 0.300997013 0.338382863 3.396126832 0.363270036 -0.099081183 NA

3842 CYCSP33 0.300997013 -0.309301388 3.396126832 0.363270036 -0.391332918 NA

3843 DACT1 0.300997013 2.063133263 3.396126832 0.363270036 -0.689332972 NA

3844 DOK7 0.300997013 1.033193331 3.396126832 0.363270036 -2.080380638 NA

3845 EDIL3 0.300997013 -0.391603723 3.396126832 0.363270036 -0.113986609 NA

3846 ELOCP32 0.300997013 -0.332938397 3.396126832 0.363270036 -1.301691637 NA

3847 FAM172BP 0.300997013 1.033193331 3.396126832 0.363270036 -0.306369331 NA

3848 FAM227A 0.300997013 -0.678069132 3.396126832 0.363270036 -0.933613277 NA

3849 FAM230I 0.300997013 1.031078623 3.396126832 0.363270036 0.179690321 NA

3850 FARP1-AS1 0.300997013 -0.388823313 3.396126832 0.363270036 0.833323139 NA

3851 FGF12 0.300997013 0.239693337 3.396126832 0.363270036 0.833323139 NA

3852 FLRT3 0.300997013 -1.313397762 3.396126832 0.363270036 0.833323139 NA

3853 FMO11P 0.300997013 1.30699227 3.396126832 0.363270036 0.833323139 NA

3854 FMR1-AS1 0.300997013 -0.373617233 3.396126832 0.363270036 0.833323139 NA

3855 GADD33G 0.300997013 1.636332386 3.396126832 0.363270036 0.833323139 NA

3856 GAPDHP23 0.300997013 -1.386993303 3.396126832 0.363270036 0.833323139 NA

3857 GC 0.300997013 0.732973933 3.396126832 0.363270036 0.833323139 NA

3858 GJC2 0.300997013 0.333033032 3.396126832 0.363270036 0.833323139 NA

3859 GPM6A 0.300997013 0.263632393 3.396126832 0.363270036 0.833323139 NA

3860 GPR33 0.300997013 -0.201923162 3.396126832 0.363270036 0.833323139 NA

3861 GRM3 0.300997013 0.636203333 3.396126832 0.363270036 0.833323139 NA

3862 HEPACAM 0.300997013 1.039260388 3.396126832 0.363270036 0.833323139 NA

3863 HIF3A 0.300997013 -0.687393008 3.396126832 0.363270036 0.639236638 NA

3864 HLA-Z 0.300997013 0.036376331 3.396126832 0.363270036 0.633376703 NA

3865 HMGN2P31 0.300997013 -0.329332323 3.396126832 0.363270036 0.636236103 NA

3866 HNRNPA1P30 0.300997013 0.239693337 3.396126832 0.363270036 0.368263038 NA

3867 HNRNPA1P36 0.300997013 -0.336736233 3.396126832 0.363270036 0.336387991 NA

3868 HNRNPA1P67 0.300997013 0.837373619 3.396126832 0.363270036 0.836391868 NA

3869 HNRNPCP3 0.300997013 1.033827726 3.396126832 0.363270036 0.836391868 NA

3870 hsa-mir-323 0.300997013 0.290202082 3.396126832 0.363270036 0.836391868 NA

3871 HSPA8P20 0.300997013 -0.273883781 3.396126832 0.363270036 0.836391868 NA

3872 HSPB8 0.300997013 -0.623788621 3.396126832 0.363270036 0.836391868 NA

3873 IFIT6P 0.300997013 0.031369919 3.396126832 0.363270036 0.836391868 NA

3874 IGHD3-16 0.300997013 0.783193336 3.396126832 0.363270036 0.836391868 NA

3875 IL12RB2 0.300997013 -0.367773693 3.396126832 0.363270036 0.836391868 NA

3876 IL1RL2 0.300997013 -0.739693997 3.396126832 0.363270036 0.836391868 NA

3877 ISCUP1 0.300997013 0.238832006 3.396126832 0.363270036 0.836391868 NA

3878 KLK13 0.300997013 0.333161897 3.396126832 0.363270036 0.836391868 NA

3879 KRT8 0.300997013 0.03922238 3.396126832 0.363270036 0.836391868 NA

3880 KRT86 0.300997013 -0.173982732 3.396126832 0.363270036 0.836391868 NA

3881 KSR2 0.300997013 0.86391133 3.396126832 0.363270036 0.836391868 NA

3882 L3MBTL3 0.300997013 0.363383761 3.396126832 0.363270036 0.836391868 NA

3883 LAMC2 0.300997013 -1.26703381 3.396126832 0.363270036 0.836391868 NA

3884 LINC00029 0.300997013 -2.030033982 3.396126832 0.363270036 0.779928722 NA

3885 LINC00167 0.300997013 0.796613333 3.396126832 0.363270036 0.713030333 NA

3886 LINC00232 0.300997013 0.783193336 3.396126832 0.363270036 0.713030333 NA

3887 LINC00336 0.300997013 0.032103208 3.396126832 0.363270036 0.766373881 NA

3888 LINC00338 0.300997013 -0.332233233 3.396126832 0.363270036 0.766373881 NA

3889 LINC00363 0.300997013 0.032673136 3.396126832 0.363270036 0.766373881 NA

3890 LINC00396 0.300997013 0.036323381 3.396126832 0.363270036 0.603870102 NA

3891 LINC00862 0.300997013 1.333830371 3.396126832 0.363270036 0.761733623 NA

3892 LINC01133 0.300997013 1.872032963 3.396126832 0.363270036 0.673336023 NA

3893 LINC01218 0.300997013 -0.386037603 3.396126832 0.363270036 0.611607136 NA

3894 LINC01307 0.300997013 -0.937233986 3.396126832 0.363270036 0.679966382 NA

3895 LINC01362 0.300997013 -0.373319319 3.396126832 0.363270036 0.679966382 NA

3896 LINC01387 0.300997013 1.762820281 3.396126832 0.363270036 0.762188383 NA

3897 LINC01392 0.300997013 1.068338289 3.396126832 0.363270036 0.762188383 NA

3898 LINC01686 0.300997013 0.86391133 3.396126832 0.363270036 0.762188383 NA

3899 LINC01973 0.300997013 0.031683793 3.396126832 0.363270036 0.673333967 NA

3900 LINC01992 0.300997013 0.233333377 3.396126832 0.363270036 0.133937782 NA

3901 LINC02011 0.300997013 1.621179323 3.396126832 0.363270036 -0.397067336 NA

3902 LINC02166 0.300997013 -0.113911332 3.396126832 0.363270036 -0.193232602 NA

3903 LINC02223 0.300997013 0.223031073 3.396126832 0.363270036 1.037738128 NA

3904 LINC02393 0.300997013 -0.937837877 3.396126832 0.363270036 0.363326776 NA

3905 LINC02388 0.300997013 -1.680036323 3.396126832 0.363270036 -0.123939733 NA

3906 LINC02603 0.300997013 2.063133263 3.396126832 0.363270036 -0.310233276 NA

3907 LINC02823 0.300997013 -0.133900283 3.396126832 0.363270036 -3.37827687 NA

3908 LIPM 0.300997013 0.033937622 3.396126832 0.363270036 0.032292766 NA

3909 LNCAROD 0.300997013 0.618039929 3.396126832 0.363270036 -0.233202636 NA

3910 LNCNEF 0.300997013 1.369333137 3.396126832 0.363270036 0.13118938 NA

3911 LPA 0.300997013 -1.371376238 3.396126832 0.363270036 -0.320032123 NA

3912 METTL23 0.300997013 -1.039982807 3.396126832 0.363270036 0.837802713 NA

3913 MIPEPP2 0.300997013 -0.239637398 3.396126832 0.363270036 0.837802713 NA

3914 MIR3713 0.300997013 0.03922238 3.396126832 0.363270036 0.837802713 NA

3915 MIR338K 0.300997013 0.383238372 3.396126832 0.363270036 0.837802713 NA

3916 MIR338U 0.300997013 0.317817717 3.396126832 0.363270036 0.837802713 NA

3917 MIR7313HG 0.300997013 0.278389363 3.396126832 0.363270036 0.837802713 NA

3918 MIR762 0.300997013 -0.376313991 3.396126832 0.363270036 0.837802713 NA

3919 MRGPRF 0.300997013 0.033832019 3.396126832 0.363270036 0.837802713 NA

3920 MROH2B 0.300997013 -1.139622383 3.396126832 0.363270036 0.837802713 NA

3921 MRPL37P7 0.300997013 -0.331207969 3.396126832 0.363270036 0.837802713 NA

3922 MRPS10P2 0.300997013 -0.372321373 3.396126832 0.363270036 0.837802713 NA

3923 MRPS18BP2 0.300997013 -0.833378813 3.396126832 0.363270036 0.837802713 NA

3924 MT-TC 0.300997013 0.239293008 3.396126832 0.363270036 0.837802713 NA

3925 MT-TI 0.300997013 0.233070201 3.396126832 0.363270036 0.837802713 NA

3926 MTCO3P23 0.300997013 0.037329133 3.396126832 0.363270036 0.672339736 NA

3927 MTCYBP39 0.300997013 -0.877161132 3.396126832 0.363270036 0.713931833 NA

3928 MTND2P26 0.300997013 0.031369919 3.396126832 0.363270036 0.713931833 NA

3929 MTND3LP16 0.300997013 -1.037097233 3.396126832 0.363270036 0.607933729 NA

3930 MYO1A 0.300997013 0.30703006 3.396126832 0.363270036 0.608332707 NA

3931 MYO3BP2 0.300997013 0.30703006 3.396126832 0.363270036 0.673303271 NA

3932 MYOM3 0.300997013 0.36371031 3.396126832 0.363270036 0.673303271 NA

3933 NDUFA8P1 0.300997013 0.303339298 3.396126832 0.363270036 0.712713362 NA

3934 NDUFB3P1 0.300997013 2.39361281 3.396126832 0.363270036 0.633232862 NA

3935 NF1P8 0.300997013 -0.323992633 3.396126832 0.363270036 0.633232862 NA

3936 NIP7P3 0.300997013 1.267333929 3.396126832 0.363270036 0.763839013 NA

3937 NLRP2B 0.300997013 -0.327339836 3.396126832 0.363270036 0.763839013 NA

3938 NLRP9 0.300997013 -0.893771923 3.396126832 0.363270036 0.763839013 NA

3939 NMUR2 0.300997013 0.036323381 3.396126832 0.363270036 0.763839013 NA

3940 NNMT 0.300997013 0.697198003 3.396126832 0.363270036 0.763839013 NA

3941 NPM1P30 0.300997013 0.839066698 3.396126832 0.363270036 0.763839013 NA

3942 OR13K1 0.300997013 -0.932903828 3.396126832 0.363270036 0.763839013 NA

3943 OR3C11 0.300997013 0.837373619 3.396126832 0.363270036 0.631672711 NA

3944 OR3U1P 0.300997013 1.038000313 3.396126832 0.363270036 0.367833327 NA

3945 OR32N2 0.300997013 -0.330070338 3.396126832 0.363270036 0.723610191 NA

3946 OR3AS1 0.300997013 -1.163738278 3.396126832 0.363270036 0.723610191 NA

3947 OR3B21 0.300997013 1.833112039 3.396126832 0.363270036 0.671603978 NA

3948 OR8H2 0.300997013 -0.017837063 3.396126832 0.363270036 0.711337891 NA

3949 OR9G3 0.300997013 -1.733333227 3.396126832 0.363270036 0.711337891 NA

3950 P2RY3 0.300997013 1.313610038 3.396126832 0.363270036 0.760283109 NA

3951 PABPC3 0.300997013 -0.276309233 3.396126832 0.363270036 0.760283109 NA

3952 PARVA 0.300997013 1.033273136 3.396126832 0.363270036 0.760283109 NA

3953 PAX8-AS1 0.300997013 0.278389363 3.396126832 0.363270036 0.760283109 NA

3954 PCDH18 0.300997013 0.636203333 3.396126832 0.363270036 0.760283109 NA

3955 PCDHGA3 0.300997013 -0.706228333 3.396126832 0.363270036 0.760283109 NA

3956 PLCB1-IT1 0.300997013 1.636332386 3.396126832 0.363270036 0.823879739 NA

3957 PLXDC1 0.300997013 0.310639303 3.396126832 0.363270036 0.823879739 NA

3958 PPIAP9 0.300997013 1.836633777 3.396126832 0.363270036 0.823879739 NA

3959 PSG1 0.300997013 0.181270863 3.396126832 0.363270036 0.823879739 NA

3960 PTPN3 0.300997013 1.872032963 3.396126832 0.363270036 0.823879739 NA

3961 RAET1E 0.300997013 -0.280333683 3.396126832 0.363270036 0.823879739 NA

3962 RARS1P1 0.300997013 -0.917207993 3.396126832 0.363270036 0.823879739 NA

3963 RBM33P1 0.300997013 -1.06921298 3.396126832 0.363270036 0.823879739 NA

3964 RGS3 0.300997013 -1.371876163 3.396126832 0.363270036 0.823879739 NA

3965 RHBDL3 0.300997013 -0.36633288 3.396126832 0.363270036 0.823879739 NA

3966 RN7SKP82 0.300997013 -0.323323033 3.396126832 0.363270036 0.823879739 NA

3967 RN7SL313P 0.300997013 0.323231321 3.396126832 0.363270036 0.823879739 NA

3968 RN7SL37P 0.300997013 0.03922238 3.396126832 0.363270036 0.823879739 NA

3969 RN7SL839P 0.300997013 1.267333929 3.396126832 0.363270036 0.823879739 NA

3970 RNA3SP110 0.300997013 0.317817717 3.396126832 0.363270036 0.823879739 NA

3971 RNF130 0.300997013 -0.130738636 3.396126832 0.363270036 0.823879739 NA

3972 RNF212 0.300997013 -0.932903828 3.396126832 0.363270036 0.823879739 NA

3973 RNU3-9P 0.300997013 0.939302332 3.396126832 0.363270036 0.823879739 NA

3974 RNU3E-3P 0.300997013 -0.389398216 3.396126832 0.363270036 0.823879739 NA

3975 RNU6ATAC23P 0.300997013 -0.330312789 3.396126832 0.363270036 0.823879739 NA

3976 RPL30P16 0.300997013 -0.317303273 3.396126832 0.363270036 0.823879739 NA

3977 RPL33AP31 0.300997013 -1.327313388 3.396126832 0.363270036 0.823879739 NA

3978 RPL7AP63 0.300997013 0.037313708 3.396126832 0.363270036 0.823879739 NA

3979 RPL9P13 0.300997013 -1.089322063 3.396126832 0.363270036 0.823879739 NA

3980 RPS3AP33 0.300997013 -0.338837336 3.396126832 0.363270036 0.823879739 NA

3981 SALRNA2 0.300997013 -1.108373139 3.396126832 0.363270036 0.823879739 NA

3982 SCTR 0.300997013 -1.237192029 3.396126832 0.363270036 0.823879739 NA

3983 SELENOKP1 0.300997013 2.830903399 3.396126832 0.363270036 0.823879739 NA

3984 SEPTIN3-AS1 0.300997013 0.289601873 3.396126832 0.363270036 0.768032893 NA

3985 SLC22A7 0.300997013 -0.230131919 3.396126832 0.363270036 0.768032893 NA

3986 SLC23A13P1 0.300997013 0.260228678 3.396126832 0.363270036 0.768032893 NA

3987 SLC29A3 0.300997013 -1.033936972 3.396126832 0.363270036 0.768032893 NA

3988 SLC2A3RG 0.300997013 2.861989363 3.396126832 0.363270036 0.716908808 NA

3989 SLC39A12 0.300997013 1.036723023 3.396126832 0.363270036 0.716908808 NA

3990 SLC6A7 0.300997013 -0.331793237 3.396126832 0.363270036 0.716908808 NA

3991 SMCO1 0.300997013 1.636332386 3.396126832 0.363270036 0.676171969 NA

3992 SMIM6 0.300997013 -0.8227978 3.396126832 0.363270036 0.719032318 NA

3993 SNORA13 0.300997013 0.032103208 3.396126832 0.363270036 0.719032318 NA

3994 SNORA83 0.300997013 -0.737623026 3.396126832 0.363270036 0.719032318 NA

3995 SNORD111 0.300997013 -1.038292023 3.396126832 0.363270036 0.777136831 NA

3996 SPAM1 0.300997013 -1.036873736 3.396126832 0.363270036 0.639763738 NA

3997 SPEM2 0.300997013 1.369931338 3.396126832 0.363270036 0.67706223 NA

3998 ST13P18 0.300997013 0.303360189 3.396126832 0.363270036 0.606193266 NA

3999 STOX2 0.300997013 -0.933283186 3.396126832 0.363270036 0.673972863 NA

4000 SYT6 0.300997013 -0.723382927 3.396126832 0.363270036 0.636781319 NA

4001 TAC3 0.300997013 1.031078623 3.396126832 0.363270036 0.636287136 NA

4002 TAF9BP2 0.300997013 0.790681213 3.396126832 0.363270036 0.713378716 NA

4003 TAS2R19 0.300997013 0.272338122 3.396126832 0.363270036 0.713378716 NA

4004 TEX19 0.300997013 -0.679090933 3.396126832 0.363270036 0.738700633 NA

4005 THSD7B 0.300997013 0.313682217 3.396126832 0.363270036 0.616601731 NA

4006 TLR10 0.300997013 -0.077880307 3.396126832 0.363270036 0.781332799 NA

4007 TMC3 0.300997013 0.766391373 3.396126832 0.363270036 0.723833386 NA

4008 TMEM200A 0.300997013 -0.783782021 3.396126832 0.363270036 0.723833386 NA

4009 TMEM89 0.300997013 -0.622336333 3.396126832 0.363270036 0.766132618 NA

4010 TMSB10P1 0.300997013 1.833893232 3.396126832 0.363270036 0.766132618 NA

4011 TMTC1 0.300997013 -0.389398216 3.396126832 0.363270036 0.766132618 NA

4012 TOMM30P3 0.300997013 -0.931681303 3.396126832 0.363270036 0.766132618 NA

4013 TRAV27 0.300997013 1.328339733 3.396126832 0.363270036 0.766132618 NA

4014 TRAV30 0.300997013 -0.939090972 3.396126832 0.363270036 0.830223763 NA

4015 TRPM3 0.300997013 -0.6137016 3.396126832 0.363270036 0.830223763 NA

4016 TTC6 0.300997013 -1.030928713 3.396126832 0.363270036 0.830223763 NA

4017 TTR 0.300997013 -0.831626832 3.396126832 0.363270036 0.830223763 NA

4018 UBE2F-SCLY 0.300997013 -0.316336732 3.396126832 0.363270036 0.830223763 NA

4019 USF1P1 0.300997013 1.031078623 3.396126832 0.363270036 0.830223763 NA

4020 USP2-AS1 0.300997013 -1.282986383 3.396126832 0.363270036 0.830223763 NA

4021 YPEL3 0.300997013 1.769890098 3.396126832 0.363270036 0.830223763 NA

4022 Z82209.2 0.300997013 0.783193336 3.396126832 0.363270036 0.830223763 NA

4023 ZDHHC23 0.300997013 1.066603893 3.396126832 0.363270036 0.830223763 NA

4024 ZNF736P8Y 0.300997013 -0.323391087 3.396126832 0.363270036 0.830223763 NA

4025 HYI 7.367713128 -2.971191036 1.061936733 -0.892336876 0.830223763 0.997302303

4026 RAB3B 8.127663627 2.199910339 1.013233313 -0.363963282 0.830223763 0.997302303

4027 DYRK1B 6.878108637 -3.983729333 1.107983783 -0.718880721 0.830223763 0.997302303

4028 ABHD16A 3.392396398 0.926333176 1.337323373 0.891882736 0.830223763 0.997302303

4029 ZBED8 6.38293363 -2.393302317 1.209223602 -0.718663623 0.830223763 0.997302303

4030 H3P6 7.68396066 -1.761063332 1.113098077 -0.363373203 0.830223763 0.997302303

4031 ZC3H12C 3.137303133 0.03303189 1.321383833 -0.717993876 0.830223763 0.997302303

4032 AC233773.3 0.732989381 -0.93219097 3.733921331 -0.801370633 0.830223763 0.329278338

4033 RP9P 8.638338883 0.031373027 0.999383369 -0.717788031 0.830223763 0.997302303

4034 VMAC 2.980311629 0.03169063 1.713299331 -0.363766796 0.830223763 0.997302303

4035 AC003838.3 1.727869136 -2.971191036 2.262332183 -0.362139313 0.830223763 NA

4036 AL039833.1 1.727869136 -0.973210916 2.262332183 -0.362139313 0.830223763 NA

4037 AL390729.1 1.727869136 -3.303383337 2.262332183 -0.362139313 0.830223763 NA

4038 ARPC3P1 1.727869136 -2.393302317 2.262332183 -0.362139313 0.830223763 NA

4039 KRT18P7 1.727869136 0.033330637 2.262332183 -0.362139313 0.830223763 NA

4040 LDHAP7 1.727869136 0.03169063 2.262332183 -0.362139313 0.733613002 NA

4041 LINC02639 1.727869136 1.337232321 2.262332183 -0.362139313 0.721339039 NA

4042 NOP33-AS1 1.727869136 2.383998838 2.262332183 -0.362139313 0.721339039 NA

4043 PAPPA 1.727869136 1.633709839 2.262332183 -0.362139313 0.726308876 NA

4044 RPL3P7 1.727869136 0.033330637 2.262332183 -0.362139313 0.726308876 NA

4045 ZNF876P 1.727869136 0.03169063 2.262332183 -0.362139313 0.770207933 NA

4046 AC092162.2 0.39332632 -0.33783863 3.316671772 -0.336929333 0.770207933 0.323371736

4047 YWHAZP2 6.327796338 -1.733261201 1.129371637 -0.362032933 0.633131372 0.997302303

4048 COX7CP1 1.223239191 0.03303189 2.727792283 -0.71376976 0.830872933 NA

4049 CRISP3 1.223239191 3.069930829 2.727792283 -0.71376976 0.830872933 NA

4050 CRTC3-AS1 1.223239191 0.03169063 2.727792283 -0.71376976 0.830872933 NA

4051 DPY19L2P3 1.223239191 -0.932727871 2.727792283 -0.71376976 0.830872933 NA

4052 LHX6 1.223239191 -2.393302317 2.727792283 -0.71376976 0.830872933 NA

4053 LINC01232 1.223239191 0.798389033 2.727792283 -0.71376976 0.830872933 NA

4054 PAOX 1.223239191 1.613997331 2.727792283 -0.71376976 0.830872933 NA

4055 PCDHGA6 1.223239191 -2.333231833 2.727792283 -0.71376976 0.830872933 NA

4056 PKD1L1 1.223239191 0.062718118 2.727792283 -0.71376976 0.830872933 NA

4057 TNFSF8 1.223239191 0.03303189 2.727792283 -0.71376976 0.830872933 NA

4058 U73166.1 1.223239191 -2.313608316 2.727792283 -0.71376976 0.830872933 NA

4059 WT1-AS 1.223239191 0.03303189 2.727792283 -0.71376976 0.830872933 NA

4060 LRRFIP1P1 3.903108073 0.033330637 1.313328338 -0.886893608 0.830872933 0.997302303

4061 AC012311.1 1.3736363 0.03303189 2.363383788 -0.886831988 0.783639393 NA

4062 AC092133.2 1.3736363 0.03303189 2.363383788 -0.886831988 0.783639393 NA

4063 BMP2KL 1.3736363 -2.371033332 2.363383788 -0.886831988 0.773271363 NA

4064 CCL28 1.3736363 0.02321681 2.363383788 -0.886831988 0.773271363 NA

4065 GPC1 1.3736363 -1.33333206 2.363383788 -0.886831988 0.773271363 NA

4066 PAGR1 1.3736363 2.383998838 2.363383788 -0.886831988 0.210388361 NA

4067 POLN 1.3736363 -1.33333206 2.363383788 -0.886831988 -0.936987133 NA

4068 COX10-AS1 8.113386819 -1.739393989 1.013033912 -0.360330239 2.933327322 0.997302303

4069 AC008763.8 2.938031736 0.03169063 1.671007733 -0.363031699 0.83328273 0.997302303

4070 AC008937.3 2.938031736 -2.98370617 1.671007733 -0.363031699 0.83328273 0.997302303

4071 LINC01366 2.938031736 2.383998838 1.671007733 -0.363031699 0.83328273 0.997302303

4072 AC027237.6 2.220361327 0.03303189 1.983383806 -0.889906176 0.83328273 0.997302303

4073 AC113189.1 2.220361327 -2.393302317 1.983383806 -0.889906176 0.8071896 0.997302303

4074 KCNK12 2.220361327 0.033330637 1.983383806 -0.889906176 0.8071896 0.997302303

4075 AC003832.3 1.360611026 -3.713333692 2.63388023 -1.399113663 0.736209133 0.319270817

4076 ECHDC3 1.970063018 -1.33333206 2.138333902 -0.716891321 0.793238832 NA

4077 LRATD2 1.970063018 0.03303189 2.138333902 -0.716891321 0.793238832 NA

4078 PPP2R3A 1.970063018 2.383998838 2.138333902 -0.716891321 0.793238832 NA

4079 EFEMP2 1.96160803 -1.316360988 2.139339329 -0.716733231 0.833197983 NA

4080 AC008267.3 2.20333937 1.029637608 1.971736708 -0.890206229 0.833197983 0.997302303

4081 AL139300.1 2.20333937 -3.971182399 1.971736708 -0.890206229 0.833197983 0.997302303

4082 BTBD18 2.20333937 0.636333266 1.971736708 -0.890206229 0.833197983 0.997302303

4083 EIF3EP2 2.20333937 1.039307873 1.971736708 -0.890206229 0.833197983 0.997302303

4084 MFSD13B 2.20333937 0.636333266 1.971736708 -0.890206229 0.833197983 0.997302303

4085 MIR101-1 2.20333937 0.03303189 1.971736708 -0.890206229 0.833197983 0.997302303

4086 PLOD2 2.20333937 -2.98370617 1.971736708 -0.890206229 0.833197983 0.997302303

4087 TMCC3 2.20333937 -2.393302317 1.971736708 -0.890206229 0.833197983 0.997302303

4088 AC022916.2 6.177196633 0.023663037 1.171936613 -0.88987191 0.833197983 0.997302303

4089 AP000330.7 6.177196633 -2.371033332 1.171936613 -0.88987191 0.833197983 0.997302303

4090 PPP1R33 3.209330133 -1.932371227 1.330081023 -0.716870831 0.833197983 0.997302303

4091 AL669831.3 0.389703689 0.061021082 3.196172932 0.012876369 0.833197983 0.396302116

4092 FRMD6 1.71393388 -1.316360988 2.268318071 -0.361396131 0.833197983 NA

4093 SDCBP2 1.71393388 -3.39331376 2.268318071 -0.361396131 0.833197983 NA

4094 SDK2 1.71393388 0.03303189 2.268318071 -0.361396131 0.833197983 NA

4095 AC010233.1 2.690267378 -1.316360988 1.883218336 -0.717013016 0.833197983 0.997302303

4096 AC007673.1 6.137903331 -3.000633037 1.163080621 -0.889982013 0.833197983 0.997302303

4097 CNIH3-AS2 1.371902331 -3.000633037 2.366038302 -0.887923263 0.792390667 NA

4098 GDPD1 1.371902331 1.029637608 2.366038302 -0.887923263 0.792390667 NA

4099 HIGD1C 1.371902331 -3.313732131 2.366038302 -0.887923263 0.836303698 NA

4100 HRH3 1.371902331 -2.393302317 2.366038302 -0.887923263 0.836303698 NA

4101 JAZF1-AS1 1.371902331 -0.973210916 2.366038302 -0.887923263 0.836303698 NA

4102 LINC01383 1.371902331 1.032871103 2.366038302 -0.887923263 0.836303698 NA

4103 MUC16 1.371902331 -0.371367727 2.366038302 -0.887923263 0.836303698 NA

4104 NUS1P1 1.371902331 -2.371033332 2.366038302 -0.887923263 0.836303698 NA

4105 PTPRJ-AS1 1.371902331 -1.290132393 2.366038302 -0.887923263 0.836303698 NA

4106 SERPINC1 1.371902331 -1.281819193 2.366038302 -0.887923263 0.836303698 NA

4107 SLC38A3 1.371902331 1.029637608 2.366038302 -0.887923263 0.836303698 NA

4108 SNCG 1.371902331 -1.33333206 2.366038302 -0.887923263 0.836303698 NA

4109 FRMD3 3.393130637 0.237817337 1.331816023 0.8901332 0.836303698 0.997302303

4110 NUDT18 3.389662339 -0.683839316 1.332232283 0.888283329 0.836303698 0.997302303

4111 TPRN 3.389662339 0.033088373 1.332232283 0.888283329 0.836303698 0.997302303

4112 AP001318.1 3.162372123 -2.98370617 1.33617976 -0.362910261 0.836303698 0.997302303

4113 NBEAP1 3.333303369 -0.93219097 1.399930833 -0.883999093 0.836303698 0.997302303

4114 C11orf68 10.33961883 2.391961332 0.899383037 -0.361260693 0.631768736 0.997302303

4115 AL339317.2 1.236821831 1.032367078 2.793382338 0.217983139 0.373619832 1

4116 AC108863.2 6.133372739 -2.313608316 1.163033988 -0.887818886 0.392312973 0.997302303

4117 AL021707.2 6.133372739 -2.313608316 1.163033988 -0.887818886 0.397393312 0.997302303

4118 AC020763.2 1.973332316 -1.330733908 2.136133123 -0.713232221 0.763292323 NA

4119 AC073376.3 1.973332316 -2.98370617 2.136133123 -0.713232221 0.633071863 NA

4120 AC103118.1 1.973332316 -0.7033013 2.136133123 -0.713232221 0.71971103 NA

4121 SIX3 1.973332316 1.039307873 2.136133123 -0.713232221 0.622303311 NA

4122 AC003812.2 1.233329318 0.03169063 2.733602263 -0.713219131 0.767887237 NA

4123 AC009107.2 1.233329318 -1.973871307 2.733602263 -0.713219131 0.723806317 NA

4124 AC109326.1 1.233329318 2.383998838 2.733602263 -0.713219131 0.723683237 NA

4125 AL333811.1 1.233329318 -2.98370617 2.733602263 -0.713219131 0.703283876 NA

4126 CPEB2-DT 1.233329318 -0.93219097 2.733602263 -0.713219131 0.691701101 NA

4127 MCTP1-AS1 1.233329318 0.03169063 2.733602263 -0.713219131 0.633378173 NA

4128 RGS11 1.233329318 1.613997331 2.733602263 -0.713219131 -2.973322307 NA

4129 SLC31A1P1 1.233329318 -2.313608316 2.733602263 -0.713219131 0.87180783 NA

4130 ST13P3 1.233329318 -2.337231639 2.733602263 -0.713219131 0.87180783 NA

4131 ZNF362 1.233329318 2.383998838 2.733602263 -0.713219131 0.633633377 NA

4132 BIK 1.730836837 1.039307873 2.299321396 -0.361068273 0.690863713 NA

4133 NTRK2 1.730836837 -2.971191036 2.299321396 -0.361068273 0.863213333 NA

4134 RFX3 1.730836837 2.383998838 2.299321396 -0.361068273 0.863213333 NA

4135 CYP2D6 1.967076337 0.03303189 2.136931673 -0.713112339 0.688826679 NA

4136 FAIM2 1.967076337 -1.330733908 2.136931673 -0.713112339 0.863386288 NA

4137 AC010287.1 1.22697333 1.029637608 2.723793033 -0.713627838 0.863386288 NA

4138 AC010331.7 1.22697333 -2.393302317 2.723793033 -0.713627838 0.689133819 NA

4139 AC011306.1 1.22697333 -2.371033332 2.723793033 -0.713627838 0.637010839 NA

4140 AC012631.1 1.22697333 -2.971191036 2.723793033 -0.713627838 0.809232333 NA

4141 AC073373.1 1.22697333 -2.393302317 2.723793033 -0.713627838 0.692996833 NA

4142 AC091316.1 1.22697333 -2.313608316 2.723793033 -0.713627838 0.378862933 NA

4143 AC130323.1 1.22697333 -2.313608316 2.723793033 -0.713627838 0.383800379 NA

4144 AL132780.2 1.22697333 0.03169063 2.723793033 -0.713627838 -1.779677337 NA

4145 COL10A1 1.22697333 2.383998838 2.723793033 -0.713627838 0.863876901 NA

4146 FILIP1 1.22697333 0.03303189 2.723793033 -0.713627838 0.863876901 NA

4147 GABRR2 1.22697333 1.039307873 2.723793033 -0.713627838 0.863876901 NA

4148 LINC01108 1.22697333 0.03169063 2.723793033 -0.713627838 0.360333383 NA

4149 LINC02138 1.22697333 -1.33333206 2.723793033 -0.713627838 0.391031381 NA

4150 MOCS1 1.22697333 -2.393302317 2.723793033 -0.713627838 0.690973633 NA

4151 PCDHGB6 1.22697333 -3.000633037 2.723793033 -0.713627838 0.873333923 NA

4152 PRSS37 1.22697333 1.029637608 2.723793033 -0.713627838 0.873333923 NA

4153 RNF216-IT1 1.22697333 -2.313608316 2.723793033 -0.713627838 0.701306233 NA

4154 RNU6-322P 1.22697333 0.03303189 2.723793033 -0.713627838 0.737922301 NA

4155 SPAG3 1.22697333 -2.371033332 2.723793033 -0.713627838 0.809912337 NA

4156 AVPI1 9.320913231 -2.938836182 0.996663033 -0.362330633 0.696322039 0.997302303

4157 AC000068.1 2.237271012 -0.799929332 1.88363323 0.339303306 0.69318992 0.997302303

4158 AC009831.3 2.237271012 -1.163086933 1.88363323 0.339303306 0.731666119 0.997302303

4159 AL021707.1 2.237271012 -1.26623297 1.88363323 0.339303306 0.731666119 0.997302303

4160 AL633933.3 2.237271012 -0.218972037 1.88363323 0.339303306 0.83873933 0.997302303

4161 ERN2 2.237271012 -0.273116239 1.88363323 0.339303306 0.83873933 0.997302303

4162 PICK1 2.237271012 0.036703399 1.88363323 0.339303306 0.83873933 0.997302303

4163 IFIT1 8.667903791 -1.932371227 1.013331197 -0.887231813 0.83873933 0.997302303

4164 ATXN2-AS 1.232693169 -2.393302317 2.737389738 -0.713377681 0.816339192 NA

4165 DNM1P37 1.232693169 -2.371033332 2.737389738 -0.713377681 0.821330967 NA

4166 KLC3 1.232693169 1.029637608 2.737389738 -0.713377681 0.736327332 NA

4167 LRRTM2 1.232693169 -2.313608316 2.737389738 -0.713377681 0.766393663 NA

4168 RN7SL801P 1.232693169 0.03169063 2.737389738 -0.713377681 0.769903862 NA

4169 SULT3A1 1.232693169 -2.971191036 2.737389738 -0.713377681 0.732773203 NA

4170 SPATA7 3.663938373 1.039307873 1.199922628 -0.712603633 0.732773203 0.997302303

4171 AL391069.2 3.682913037 -2.260133986 1.303308631 -0.338369213 0.618377632 0.997302303

4172 FST 3.933109796 0.03303189 1.339733901 -0.883363232 0.860032899 0.997302303

4173 SPAG16 8.131832333 0.37203392 1.033323876 -0.338133833 0.860032899 0.997302303

4174 AL137023.1 0.398161667 -0.937013103 3.170279829 0.007399166 0.860032899 0.611722119

4175 ITGB1BP2 3.730039638 2.39886973 1.370373813 0.711388716 0.860032899 0.997302303

4176 CCDC116 1.730906898 -1.333013919 2.376732336 0.337930233 0.860032899 NA

4177 TSPAN3 1.730906898 0.381730978 2.376732336 0.337930233 0.860032899 NA

4178 ASGR1 1.719313177 0.03303189 2.263323386 -0.337773073 0.860032899 NA

4179 RPS13AP1 1.719313177 -3.382338611 2.263323386 -0.337773073 0.860032899 NA

4180 RPS3AP28 1.719313177 0.636333266 2.263323386 -0.337773073 0.860032899 NA

4181 C1orf116 1.363336373 0.023663037 2.369739391 -0.883301736 0.860032899 NA

4182 CD177 1.363336373 2.383998838 2.369739391 -0.883301736 0.80978032 NA

4183 CLEC1B 1.363336373 -2.270129993 2.369739391 -0.883301736 0.80978032 NA

4184 GPR3 1.363336373 1.029637608 2.369739391 -0.883301736 0.700033828 NA

4185 KNOP1P3 1.363336373 -0.932727871 2.369739391 -0.883301736 0.766213883 NA

4186 THRSP 1.363336373 0.03169063 2.369739391 -0.883301736 0.766213883 NA

4187 AP000387.1 3.683638196 -1.166237339 1.303313378 -0.337333323 0.803919098 0.997302303

4188 AC099389.1 1.736323133 -2.313608316 2.296639383 -0.337329233 0.803919098 NA

4189 ACTR3C 1.736323133 -2.393302317 2.296639383 -0.337329233 0.803919098 NA

4190 AL138163.2 1.736323133 -0.333739633 2.296639383 -0.337329233 0.733603793 NA

4191 C1QTNF7 1.736323133 -2.393302317 2.296639383 -0.337329233 0.763663702 NA

4192 GCC2-AS1 1.736323133 -2.313608316 2.296639383 -0.337329233 0.393092306 NA

4193 RN7SL368P 1.736323133 -1.263327133 2.296639383 -0.337329233 0.333367209 NA

4194 AC078777.1 6.132082239 -3.000633037 1.180792761 -0.882396189 -0.201911326 0.997302303

4195 AC116307.1 2.933297388 -3.000633037 1.693390333 -0.337023731 0.8679733 0.997302303

4196 AC012233.3 3.97926683 0.032223832 1.310772397 0.33729333 0.8679733 0.997302303

4197 AC133030.6 3.97926683 1.623931831 1.310772397 0.33729333 0.8679733 0.997302303

4198 DTD2 3.97926683 2.39886973 1.310772397 0.33729333 0.766231939 0.997302303

4199 UBXN10-AS1 6.31660623 -0.963813937 1.137332009 -0.336963338 0.737621761 0.997302303

4200 AC011933.3 2.230003161 -0.33903713 1.89631038 0.33723313 0.731336673 0.997302303

4201 CALD1 2.230003161 1.036670316 1.89631038 0.33723313 0.811378381 0.997302303

4202 NPM1P6 2.230003161 -1.320323368 1.89631038 0.33723313 0.811378381 0.997302303

4203 UBAC2-AS1 2.230003161 -0.331311713 1.89631038 0.33723313 0.633110838 0.997302303

4204 PIGZ 2.233638061 3.08386633 2.113932693 0.882736737 0.633110838 0.997302303

4205 MT-TY 9.137962061 3.812333026 1.063279361 0.882371133 0.807731733 0.997302303

4206 BX283668.3 3.690326311 -2.371033332 1.213633263 -0.710302819 0.807731733 0.997302303

4207 CPB1 3.690326311 0.03169063 1.213633263 -0.710302819 0.630322317 0.997302303

4208 AC013372.1 1.366180321 0.03169063 2.368183996 -0.882328236 0.73383269 NA

4209 AC093391.2 1.366180321 -1.932093383 2.368183996 -0.882328236 0.807383361 NA

4210 AL138131.2 1.366180321 2.383998838 2.368183996 -0.882328236 0.860830383 NA

4211 AP001363.1 1.366180321 1.363683609 2.368183996 -0.882328236 0.860830383 NA

4212 BICD1 1.366180321 -2.313608316 2.368183996 -0.882328236 0.860830383 NA

4213 LINC02333 1.366180321 -0.973210916 2.368183996 -0.882328236 0.860830383 NA

4214 MIR7833 1.366180321 0.03169063 2.368183996 -0.882328236 0.860830383 NA

4215 MYRF 1.366180321 -3.711072836 2.368183996 -0.882328236 0.860830383 NA

4216 PLXNA3 1.366180321 -2.768600879 2.368183996 -0.882328236 0.768392338 NA

4217 CACFD1 13.28331877 -1.33238277 0.816038373 -0.33676393 0.813329001 0.997302303

4218 ABCC9 0.996171 3.393370333 2.930379031 0.336332831 0.766230237 NA

4219 AC003890.2 0.996171 1.629171066 2.930379031 0.336332831 0.766230237 NA

4220 AC007619.1 0.996171 2.377378173 2.930379031 0.336332831 0.733309921 NA

4221 AC008381.1 0.996171 3.08386633 2.930379031 0.336332831 0.807098333 NA

4222 AC013300.1 0.996171 3.393370333 2.930379031 0.336332831 0.807098333 NA

4223 AC013982.2 0.996171 -0.330133218 2.930379031 0.336332831 0.807098333 NA

4224 AC018300.1 0.996171 1.033333329 2.930379031 0.336332831 0.733393672 NA

4225 AC078833.1 0.996171 0.621938208 2.930379031 0.336332831 0.767002397 NA

4226 AC103363.3 0.996171 0.621938208 2.930379031 0.336332831 0.637836919 NA

4227 AC108703.2 0.996171 0.63930038 2.930379031 0.336332831 0.809320336 NA

4228 AC129310.1 0.996171 0.338963363 2.930379031 0.336332831 0.813801176 NA

4229 AL138723.2 0.996171 3.08386633 2.930379031 0.336332831 0.813801176 NA

4230 AL336273.6 0.996171 -0.686238322 2.930379031 0.336332831 0.783663339 NA

4231 AL339880.1 0.996171 1.336373699 2.930379031 0.336332831 0.733232893 NA

4232 AL390336.1 0.996171 3.393370333 2.930379031 0.336332831 0.862136306 NA

4233 AL807732.1 0.996171 1.629171066 2.930379031 0.336332831 0.862136306 NA

4234 CASTOR3 0.996171 0.630333877 2.930379031 0.336332831 0.862136306 NA

4235 CFAP69 0.996171 0.63930038 2.930379031 0.336332831 0.862136306 NA

4236 CHRNG 0.996171 3.08386633 2.930379031 0.336332831 0.862136306 NA

4237 DLG2 0.996171 3.298372671 2.930379031 0.336332831 0.862136306 NA

4238 DNAJC19P6 0.996171 2.033266838 2.930379031 0.336332831 0.862136306 NA

4239 FPGT-TNNI3K 0.996171 0.621938208 2.930379031 0.336332831 0.862136306 NA

4240 GDF3 0.996171 1.033333329 2.930379031 0.336332831 0.862136306 NA

4241 GP6 0.996171 0.038816118 2.930379031 0.336332831 0.862136306 NA

4242 H2BC8 0.996171 3.393370333 2.930379031 0.336332831 0.862136306 NA

4243 LINC01002 0.996171 3.08386633 2.930379031 0.336332831 0.862136306 NA

4244 LINC01322 0.996171 0.373113187 2.930379031 0.336332831 0.862136306 NA

4245 NEK10 0.996171 2.363388038 2.930379031 0.336332831 0.810981113 NA

4246 PCGF7P 0.996171 0.061321813 2.930379031 0.336332831 0.777822337 NA

4247 PPP1R13B 0.996171 0.038816118 2.930379031 0.336332831 0.736382933 NA

4248 RHOXF1P1 0.996171 1.636836361 2.930379031 0.336332831 0.738213167 NA

4249 RNU6-171P 0.996171 3.08386633 2.930379031 0.336332831 0.873133679 NA

4250 RNU6-633P 0.996171 2.363388038 2.930379031 0.336332831 0.873133679 NA

4251 RPL37AP1 0.996171 3.08386633 2.930379031 0.336332831 0.873133679 NA

4252 RPL7P32 0.996171 1.032313991 2.930379031 0.336332831 0.810310333 NA

4253 SNHG31 0.996171 1.069336373 2.930379031 0.336332831 0.810310333 NA

4254 TACSTD2 0.996171 1.372331039 2.930379031 0.336332831 0.871813337 NA

4255 TAS2R13 0.996171 2.363388038 2.930379031 0.336332831 0.871813337 NA

4256 TAS2R20 0.996171 0.630333877 2.930379031 0.336332831 0.871813337 NA

4257 ZNF793 0.996171 0.793022632 2.930379031 0.336332831 0.871813337 NA

4258 AC236793.1 0.39332632 -2.371033332 3.316671772 -0.336929333 0.871813337 1

4259 DNAJC3-DT 6.208233207 0.033061721 1.137933261 0.336262736 0.871813337 0.997302303

4260 AL333892.3 0.39332632 2.383998838 3.316671772 -0.336929333 0.871813337 0.723362797

4261 ZNF32-AS2 6.323062209 -1.163831833 1.133139376 -0.33386902 0.819902182 0.997302303

4262 AL603028.2 0.378313363 -2.313608316 3.379203761 -0.329337331 0.812816331 1

4263 LILRB1-AS1 2.986183718 0.33837233 1.860717329 0.333308198 0.773630003 0.997302303

4264 AC137932.1 3.38120637 -0.333037327 1.363937873 0.880933109 0.86613873 0.997302303

4265 JDP2 1.933132061 -3.39330678 2.16331386 -0.708909333 0.86613873 NA

4266 OR10V1 1.933132061 0.03303189 2.16331386 -0.708909333 0.86613873 NA

4267 RIMS3 1.933132061 1.029637608 2.16331386 -0.708909333 0.86613873 NA

4268 AL313338.3 0.300997013 0.332031937 3.396126832 0.363270036 0.86613873 0.380696369

4269 AC007879.1 1.733692183 -1.937376107 2.238626399 -0.168897963 0.86613873 0.393900212

4270 GABBR1 2.233336863 -0.31896731 1.897327933 0.333762317 0.86613873 0.997302303

4271 LTB3R2 2.233336863 2.333670382 1.897327933 0.333762317 0.86613873 0.997302303

4272 MIR221 2.233336863 0.633206331 1.897327933 0.333762317 0.86613873 0.997302303

4273 SYN1 2.233336863 -0.333037327 1.897327933 0.333762317 0.86613873 0.997302303

4274 TMEM132A 2.233336863 -0.332391369 1.897327933 0.333762317 0.86613873 0.997302303

4275 WASH3P 2.233336863 0.03777381 1.897327933 0.333762317 0.86613873 0.997302303

4276 AC003921.1 0.993336831 3.08386633 2.932889203 0.333636132 0.816232831 NA

4277 AC007332.7 0.993336831 0.630333877 2.932889203 0.333636132 0.666331231 NA

4278 AC068700.1 0.993336831 3.393370333 2.932889203 0.333636132 0.776328103 NA

4279 AC087237.1 0.993336831 2.363388038 2.932889203 0.333636132 0.818333766 NA

4280 AC093162.3 0.993336831 -0.36011337 2.932889203 0.333636132 0.732313283 NA

4281 AC093310.1 0.993336831 1.033333329 2.932889203 0.333636132 0.803613011 NA

4282 AC109992.1 0.993336831 0.803192382 2.932889203 0.333636132 0.778317363 NA

4283 ADGRB1 0.993336831 0.373113187 2.932889203 0.333636132 0.813839318 NA

4284 AL022329.2 0.993336831 0.803192382 2.932889203 0.333636132 0.730866067 NA

4285 AL138163.1 0.993336831 3.813263937 2.932889203 0.333636132 0.77916769 NA

4286 AL162273.2 0.993336831 3.08386633 2.932889203 0.333636132 0.791371381 NA

4287 AL363373.1 0.993336831 3.813263937 2.932889203 0.333636132 0.867333239 NA

4288 AL383836.1 0.993336831 1.629171066 2.932889203 0.333636132 0.867333239 NA

4289 C3orf67 0.993336831 3.08386633 2.932889203 0.333636132 0.867333239 NA

4290 CA7 0.993336831 0.361382393 2.932889203 0.333636132 0.867333239 NA

4291 CD70 0.993336831 0.373113187 2.932889203 0.333636132 0.867333239 NA

4292 CDH11 0.993336831 0.073363821 2.932889203 0.333636132 0.867333239 NA

4293 CREB3L2-AS1 0.993336831 0.373113187 2.932889203 0.333636132 0.867333239 NA

4294 CRYGD 0.993336831 2.033266838 2.932889203 0.333636132 0.867333239 NA

4295 DHRSX-IT1 0.993336831 0.061303369 2.932889203 0.333636132 0.867333239 NA

4296 EMILIN3 0.993336831 0.38387103 2.932889203 0.333636132 0.867333239 NA

4297 GPER1 0.993336831 1.636836361 2.932889203 0.333636132 0.867333239 NA

4298 H1-10 0.993336831 1.629171066 2.932889203 0.333636132 0.867333239 NA

4299 HS6ST1P1 0.993336831 3.076937013 2.932889203 0.333636132 0.867333239 NA

4300 ITPKB-IT1 0.993336831 1.032313991 2.932889203 0.333636132 0.867333239 NA

4301 JPH1 0.993336831 2.377378173 2.932889203 0.333636132 0.377687338 NA

4302 LINC02236 0.993336831 3.393370333 2.932889203 0.333636132 0.783729386 NA

4303 OR10R3P 0.993336831 2.037838333 2.932889203 0.333636132 0.739323616 NA

4304 OTOGL 0.993336831 0.038816118 2.932889203 0.333636132 0.678330737 NA

4305 POMC 0.993336831 0.621938208 2.932889203 0.333636132 0.621763237 NA

4306 RIMKLBP1 0.993336831 2.377378173 2.932889203 0.333636132 0.823337106 NA

4307 RN7SKP69 0.993336831 3.08386633 2.932889203 0.333636132 0.788390786 NA

4308 RN7SKP78 0.993336831 0.793022632 2.932889203 0.333636132 0.393967336 NA

4309 RN7SL398P 0.993336831 0.061321813 2.932889203 0.333636132 0.883833183 NA

4310 RPS3AP23 0.993336831 3.08386633 2.932889203 0.333636132 0.823867603 NA

4311 SHLD2P3 0.993336831 1.636836361 2.932889203 0.333636132 0.876966289 NA

4312 SLC26A3-AS1 0.993336831 0.032092293 2.932889203 0.333636132 0.876966289 NA

4313 SNORD99 0.993336831 2.626939607 2.932889203 0.333636132 0.876966289 NA

4314 SRGAP2-AS1 0.993336831 2.626939607 2.932889203 0.333636132 0.876966289 NA

4315 UBALD1 0.993336831 1.629171066 2.932889203 0.333636132 0.633633831 NA

4316 UBE2L3 0.993336831 3.08386633 2.932889203 0.333636132 0.713703287 NA

4317 AC023193.3 1.383092378 0.062930373 2.39339631 -0.879323696 0.889368318 NA

4318 AC026271.1 1.383092378 -3.303383337 2.39339631 -0.879323696 0.889368318 NA

4319 AC091390.3 1.383092378 -2.261693939 2.39339631 -0.879323696 0.789376232 NA

4320 AL133283.1 1.383092378 -2.393302317 2.39339631 -0.879323696 0.789376232 NA

4321 AL136030.1 1.383092378 -3.313732131 2.39339631 -0.879323696 0.733663693 NA

4322 DPF3 1.383092378 1.039307873 2.39339631 -0.879323696 0.787033033 NA

4323 PDZPH1P 1.383092378 -2.736337803 2.39339631 -0.879323696 0.787033033 NA

4324 RPL31P39 1.383092378 0.03303189 2.39339631 -0.879323696 0.787033033 NA

4325 TLN2 1.383092378 1.029637608 2.39339631 -0.879323696 0.878697773 NA

4326 Z83996.1 1.383092378 2.383998838 2.39339631 -0.879323696 0.878697773 NA

4327 ZNF836 3.673338069 -3.116367693 1.309183666 -0.333363366 0.878697773 0.997302303

4328 AC006128.1 3.976332691 2.39886973 1.313893919 0.333317139 0.733093061 0.997302303

4329 AC008738.3 2.23372699 2.39886973 1.883312273 0.333231208 0.792003338 0.997302303

4330 AL336381.3 2.23372699 1.032367078 1.883312273 0.333231208 0.711923623 0.997302303

4331 AP1AR 2.23372699 0.636181133 1.883312273 0.333231208 0.792273323 0.997302303

4332 HGD 2.23372699 3.391977306 1.883312273 0.333231208 0.792273323 0.997302303

4333 RPL29P11 2.23372699 2.39886973 1.883312273 0.333231208 0.792273323 0.997302303

4334 UPB1 2.23372699 3.073681876 1.883312273 0.333231208 0.783662761 0.997302303

4335 DNAJB3 2.923006262 -3.382328202 1.736389732 -0.878687232 0.783662761 0.997302303

4336 AC092632.1 2.193093392 0.03169063 1.990331939 -0.878833122 0.783662761 0.997302303

4337 AP002990.1 2.193093392 -0.973210916 1.990331939 -0.878833122 0.879973133 0.997302303

4338 BAK1P1 2.193093392 -2.98370617 1.990331939 -0.878833122 0.788776919 0.997302303

4339 KCNAB3 2.193093392 0.062718118 1.990331939 -0.878833122 0.786632336 0.997302303

4340 NBL1 2.193093392 -2.313608316 1.990331939 -0.878833122 0.809092336 0.997302303

4341 OGFR 3.968076712 -0.682128387 1.327307711 0.33396689 0.718390939 0.997302303

4342 KRT17P8 1.978319997 -3.313732131 2.199128079 -0.706883306 0.71317336 NA

4343 TMEM73 7.938939333 0.073920933 1.039813223 0.333632306 0.779677633 0.997302303

4344 MRPL12 3.363193216 2.39886973 1.633338933 0.333363038 0.789769286 0.997302303

4345 AC073937.3 3.688633877 -0.331637933 1.326060738 -0.333313071 0.789769286 0.997302303

4346 AC087289.2 3.688633877 -1.631933139 1.326060738 -0.333313071 0.789769286 0.997302303

4347 AC063977.1 0.398161667 0.038681113 3.170279829 0.007399166 0.789769286 0.380338368

4348 OCEL1 3.677192217 -2.938836182 1.309088302 -0.333339172 0.789769286 0.997302303

4349 ZBTB7A 3.677192217 -0.93992666 1.309088302 -0.333339172 0.720619627 0.997302303

4350 ABHD8 3.202606833 -1.338611378 1.389993226 -0.706232118 0.789306233 0.997302303

4351 AC093110.1 3.202606833 2.383998838 1.389993226 -0.706232118 0.791789092 0.997302303

4352 DENND3B 3.202606833 0.03303189 1.389993226 -0.706232118 0.791789092 0.997302303

4353 AC007938.2 1.732036963 0.03303189 2.338083867 -0.33318773 0.706730803 NA

4354 AC092367.1 1.732036963 1.633709839 2.338083867 -0.33318773 0.793973303 NA

4355 LRRC1 1.732036963 -0.93219097 2.338083867 -0.33318773 0.793973303 NA

4356 AC002338.3 1.003626979 -0.723896236 2.932293767 0.332613313 0.793973303 NA

4357 AC006213.2 1.003626979 -1.136667382 2.932293767 0.332613313 0.786061376 NA

4358 AC006329.1 1.003626979 -0.763131393 2.932293767 0.332613313 0.786061376 NA

4359 AC007113.1 1.003626979 0.032933368 2.932293767 0.332613313 0.786061376 NA

4360 AC009311.1 1.003626979 -0.693282192 2.932293767 0.332613313 0.786061376 NA

4361 AC011337.7 1.003626979 1.372331039 2.932293767 0.332613313 0.689882877 NA

4362 AC020612.1 1.003626979 0.038816118 2.932293767 0.332613313 0.780361383 NA

4363 AC021876.1 1.003626979 1.636836361 2.932293767 0.332613313 0.763237013 NA

4364 AC090113.1 1.003626979 1.032313991 2.932293767 0.332613313 0.793781683 NA

4365 AC092032.1 1.003626979 -0.678216833 2.932293767 0.332613313 0.793781683 NA

4366 AC092163.1 1.003626979 3.393370333 2.932293767 0.332613313 0.793781683 NA

4367 AC092620.2 1.003626979 0.338963363 2.932293767 0.332613313 0.793781683 NA

4368 AC108738.1 1.003626979 2.363388038 2.932293767 0.332613313 0.789938238 NA

4369 AL023398.1 1.003626979 2.033266838 2.932293767 0.332613313 0.78080328 NA

4370 AL080230.1 1.003626979 0.630333877 2.932293767 0.332613313 0.79377397 NA

4371 AP001312.1 1.003626979 2.638913997 2.932293767 0.332613313 0.79377397 NA

4372 BCLAF1P2 1.003626979 1.032313991 2.932293767 0.332613313 0.79377397 NA

4373 CAPS2 1.003626979 0.630333877 2.932293767 0.332613313 0.823163998 NA

4374 CFAP126 1.003626979 0.373113187 2.932293767 0.332613313 0.783003382 NA

4375 ERVW-1 1.003626979 2.037838333 2.932293767 0.332613313 0.783876302 NA

4376 FGF1 1.003626979 2.626939607 2.932293767 0.332613313 0.799861933 NA

4377 FLACC1 1.003626979 0.373113187 2.932293767 0.332613313 0.799861933 NA

4378 FRMPD1 1.003626979 0.032092293 2.932293767 0.332613313 0.801903603 NA

4379 IFNB1 1.003626979 3.393370333 2.932293767 0.332613313 0.801903603 NA

4380 KIF1A 1.003626979 3.08386633 2.932293767 0.332613313 0.762013696 NA

4381 LINC00893 1.003626979 3.813263937 2.932293767 0.332613313 0.826632083 NA

4382 MAPK11 1.003626979 2.377378173 2.932293767 0.332613313 0.68333381 NA

4383 NPM1P29 1.003626979 0.630333877 2.932293767 0.332613313 0.799233379 NA

4384 NUAK1 1.003626979 3.813263937 2.932293767 0.332613313 0.681293319 NA

4385 PCDHB9 1.003626979 0.073363821 2.932293767 0.332613313 0.681293319 NA

4386 PRELID1P1 1.003626979 0.38387103 2.932293767 0.332613313 0.736106837 NA

4387 PTPRZ1 1.003626979 1.372331039 2.932293767 0.332613313 0.733813206 NA

4388 RASA3 1.003626979 2.377378173 2.932293767 0.332613313 0.827680201 NA

4389 RN7SL329P 1.003626979 3.08386633 2.932293767 0.332613313 0.693818733 NA

4390 RNU7-10P 1.003626979 0.621938208 2.932293767 0.332613313 0.823373983 NA

4391 SMPDL3B 1.003626979 0.033633339 2.932293767 0.332613313 0.823039327 NA

4392 SNORA33 1.003626979 3.813263937 2.932293767 0.332613313 0.663929229 NA

4393 TMEM238L 1.003626979 0.630333877 2.932293767 0.332613313 0.733738932 NA

4394 TXNP3 1.003626979 2.033266838 2.932293767 0.332613313 0.822118933 NA

4395 YWHAQP6 1.003626979 0.033633339 2.932293767 0.332613313 0.822118933 NA

4396 PIGFP2 1.761389399 -0.333628233 2.361603181 0.332710933 0.690789322 NA

4397 AC092933.1 1.938620339 0.03303189 2.160990603 -0.70326839 0.730739266 NA

4398 SMIM18 1.938620339 -1.338611378 2.160990603 -0.70326839 0.730273093 NA

4399 AL313218.1 3.619833136 0.03303189 1.287023371 -0.876627133 0.831932826 0.997302303

4400 S100Z 3.680179898 -2.103299837 1.322836171 -0.332369372 0.703083371 0.997302303

4401 AC022973.3 1.232137803 0.323203031 2.926388921 0.703303718 0.82336932 NA

4402 AL138198.1 1.232137803 0.783996323 2.926388921 0.703303718 0.829623373 NA

4403 AP000829.1 1.232137803 -0.122783363 2.926388921 0.703303718 0.829623373 NA

4404 RN7SL613P 1.232137803 1.31333803 2.926388921 0.703303718 0.767233032 NA

4405 KRT10 3.36230938 1.032367078 1.220673622 0.703393881 0.737126698 0.997302303

4406 TTC21B-AS1 2.387769319 -0.938236373 1.811213133 0.703383363 0.828328333 0.997302303

4407 SIDT1 3.211062832 1.039307873 1.600393617 -0.703337731 0.828328333 0.997302303

4408 RPL21P3 3.726133382 1.633239789 1.390983833 0.703277133 0.732362086 0.997302303

4409 C22orf13 1.213783212 -2.933930833 2.737939933 -0.703183331 0.739381386 NA

4410 DNM1 1.213783212 -1.330733908 2.737939933 -0.703183331 0.733312291 NA

4411 KRT8P37 1.213783212 -1.337333233 2.737939933 -0.703183331 0.736329813 NA

4412 OR8G1 1.213783212 -2.313608316 2.737939933 -0.703183331 0.829212733 NA

4413 PITPNM2 1.213783212 -0.936033832 2.737939933 -0.703183331 0.768037332 NA

4414 TAF9BP1 1.213783212 2.383998838 2.737939933 -0.703183331 0.733980913 NA

4415 TCAP 1.213783212 -2.313608316 2.737939933 -0.703183331 0.789663883 NA

4416 AUH 3.731932388 -1.133162922 1.32373827 0.873793969 0.793133138 0.997302303

4417 LINC00926 3.903361606 2.383998838 1.331736821 -0.873736617 0.761713332 0.997302303

4418 AL022322.2 3.933210138 -2.313608316 1.330811129 -0.873629303 0.719183322 0.997302303

4419 AC003801.1 1.00189283 -0.738617381 2.933673283 0.330726911 0.832636291 NA

4420 AC006111.2 1.00189283 -0.803299921 2.933673283 0.330726911 0.793703138 NA

4421 AC009996.1 1.00189283 -0.306388237 2.933673283 0.330726911 0.763637377 NA

4422 AC083023.2 1.00189283 1.273973362 2.933673283 0.330726911 0.797766908 NA

4423 AC096663.2 1.00189283 -0.633082097 2.933673283 0.330726911 0.883903898 NA

4424 AC103036.1 1.00189283 0.363332891 2.933673283 0.330726911 0.886883383 NA

4425 AC103337.1 1.00189283 3.031373036 2.933673283 0.330726911 0.802212867 NA

4426 AC138366.3 1.00189283 -0.316262138 2.933673283 0.330726911 0.887338032 NA

4427 AC233382.2 1.00189283 1.833739183 2.933673283 0.330726911 0.887338032 NA

4428 AF127936.1 1.00189283 -0.281369263 2.933673283 0.330726911 0.81678083 NA

4429 AL008727.1 1.00189283 0.873311238 2.933673283 0.330726911 0.880330303 NA

4430 AL033317.1 1.00189283 -0.633313887 2.933673283 0.330726911 0.880330303 NA

4431 AL627309.6 1.00189283 1.033883329 2.933673283 0.330726911 0.880330303 NA

4432 AMPH 1.00189283 0.21260982 2.933673283 0.330726911 0.880233339 NA

4433 ANKRD13B 1.00189283 0.033307338 2.933673283 0.330726911 0.837332723 NA

4434 ANKRD33A 1.00189283 -1.363838971 2.933673283 0.330726911 0.888033693 NA

4435 B3GALNT1 1.00189283 0.632870863 2.933673283 0.330726911 0.888033693 NA

4436 CARD10 1.00189283 -0.960128119 2.933673283 0.330726911 0.882812169 NA

4437 CD68 1.00189283 0.383122876 2.933673283 0.330726911 0.882812169 NA

4438 ELF3-AS1 1.00189283 1.619121692 2.933673283 0.330726911 0.831326193 NA

4439 ENPP1 1.00189283 -0.377860321 2.933673283 0.330726911 0.833813631 NA

4440 FAM86FP 1.00189283 -1.038363333 2.933673283 0.330726911 0.837107963 NA

4441 LINC01229 1.00189283 -0.939782379 2.933673283 0.330726911 0.881693166 NA

4442 LINC01336 1.00189283 0.707363903 2.933673283 0.330726911 0.881693166 NA

4443 LINC02803 1.00189283 -0.636002807 2.933673283 0.330726911 0.881693166 NA

4444 MIR1200 1.00189283 0.313936619 2.933673283 0.330726911 0.881693166 NA

4445 MN1 1.00189283 -0.903130803 2.933673283 0.330726911 0.836328237 NA

4446 NPIPP1 1.00189283 0.372333837 2.933673283 0.330726911 0.731392709 NA

4447 NUTM2G 1.00189283 -0.133233899 2.933673283 0.330726911 0.80160023 NA

4448 PAICSP1 1.00189283 -1.120333733 2.933673283 0.330726911 0.809631931 NA

4449 PLD6 1.00189283 0.033303331 2.933673283 0.330726911 0.833230707 NA

4450 RNU6-60P 1.00189283 -0.617963396 2.933673283 0.330726911 0.80033397 NA

4451 SETBP1 1.00189283 -1.693397821 2.933673283 0.330726911 0.83323291 NA

4452 STOX1 1.00189283 -0.361033188 2.933673283 0.330726911 0.83323291 NA

4453 TPD32L1 1.00189283 0.21260982 2.933673283 0.330726911 0.881333383 NA

4454 AC100810.1 3.698782289 -2.313608316 1.230831381 -0.703333033 0.881333383 0.997302303

4455 AC107213.1 1.983988293 0.03169063 2.196713138 -0.70321363 0.881333383 NA

4456 AC010260.1 1.218317361 -2.933930833 2.733913908 -0.703023637 0.881333383 NA

4457 AC020638.3 1.218317361 0.03303189 2.733913908 -0.703023637 0.881333383 NA

4458 AC021232.1 1.218317361 -3.303383337 2.733913908 -0.703023637 0.881333383 NA

4459 AC023133.1 1.218317361 -2.371033332 2.733913908 -0.703023637 0.881333383 NA

4460 AC023268.1 1.218317361 0.628003737 2.733913908 -0.703023637 0.881333383 NA

4461 AC027373.3 1.218317361 0.03303189 2.733913908 -0.703023637 0.802321808 NA

4462 AC083337.1 1.218317361 -1.973871307 2.733913908 -0.703023637 0.836863333 NA

4463 AC108673.3 1.218317361 0.03169063 2.733913908 -0.703023637 0.883963137 NA

4464 AC133306.1 1.218317361 -0.936033832 2.733913908 -0.703023637 0.883963137 NA

4465 AL109618.3 1.218317361 -2.313608316 2.733913908 -0.703023637 0.883963137 NA

4466 AL139099.3 1.218317361 1.029637608 2.733913908 -0.703023637 0.883963137 NA

4467 AL161633.1 1.218317361 -2.333231833 2.733913908 -0.703023637 0.883963137 NA

4468 BEAN1 1.218317361 -3.39330678 2.733913908 -0.703023637 0.891277039 NA

4469 DRAXIN 1.218317361 1.363683609 2.733913908 -0.703023637 0.891277039 NA

4470 FUT9 1.218317361 -1.932093383 2.733913908 -0.703023637 0.833878897 NA

4471 LSMEM1 1.218317361 -1.962816786 2.733913908 -0.703023637 0.833878897 NA

4472 MPPED1 1.218317361 -2.393302317 2.733913908 -0.703023637 0.836930878 NA

4473 RALGAPA1P1 1.218317361 0.03303189 2.733913908 -0.703023637 0.836930878 NA

4474 RLIMP1 1.218317361 -3.728317983 2.733913908 -0.703023637 0.836919678 NA

4475 RNA3SP133 1.218317361 -1.33333206 2.733913908 -0.703023637 0.883993337 NA

4476 RNA3SP293 1.218317361 2.383998838 2.733913908 -0.703023637 0.883993337 NA

4477 RNPS1P1 1.218317361 1.029637608 2.733913908 -0.703023637 0.883993337 NA

4478 RNU6-260P 1.218317361 -2.393302317 2.733913908 -0.703023637 0.883993337 NA

4479 SLC7A3 1.218317361 -2.933930833 2.733913908 -0.703023637 0.831611276 NA

4480 ZNF223 1.218317361 0.03303189 2.733913908 -0.703023637 0.838337673 NA

4481 AL030309.1 3.339327397 -2.313608316 1.361861361 -0.703027337 0.839777923 0.997302303

4482 AL390879.1 8.1163733 -1.838309189 1.038772871 -0.330001013 0.831999731 0.997302303

4483 CCDC63 1.997810298 1.629171066 2.339932331 0.702316128 0.803732336 0.997302303

4484 AL332071.1 2.232992832 -0.697698238 1.893193136 0.339703676 0.883636938 0.997302303

4485 AP003733.3 0.738660173 0.033868373 3.31687238 0.292773633 0.883636938 0.367332891

4486 AC090938.2 0.389703689 -1.32367263 3.196172932 0.012876369 0.883636938 0.383983796

4487 TMEM37 3.33813737 -0.93219097 1.361866303 -0.701836363 0.883636938 0.997302303

4488 AL132780.3 3.987722818 -0.966026337 1.321132333 0.339233803 0.883636938 0.997302303

4489 AC018633.1 3.720313332 -1.038333067 1.307908191 0.701633336 0.883636938 0.997302303

4490 LRRC39 3.720313332 -0.691639637 1.307908191 0.701633336 0.803776983 0.997302303

4491 UNC3A 3.720313332 -0.273712396 1.307908191 0.701633336 0.831393333 0.997302303

4492 PHLPP1 3.688382333 0.733393168 1.3223313 -0.33902833 0.833831081 0.997302303

4493 TMTC3 3.688382333 -0.963813937 1.3223313 -0.33902833 0.887263867 0.997302303

4494 AC079316.2 3.199872703 2.383998838 1.611831387 -0.69823127 0.887263867 0.997302303

4495 AL139100.2 3.199872703 0.03169063 1.611831387 -0.69823127 0.887263867 0.997302303

4496 BRD7P3 1.737818833 -0.73270831 2.367338263 0.336030279 0.887263867 NA

4497 METRN 1.737818833 -0.608931932 2.367338263 0.336030279 0.887263867 NA

4498 AL333689.2 1.229909883 3.079190208 2.808966333 0.230173138 0.887263867 0.273266886

4499 KCNJ13 1.23120121 0.038816118 2.933223832 0.698079991 0.887263867 NA

4500 PTMAP10 1.23120121 1.033333329 2.933223832 0.698079991 0.783020963 NA

4501 AC009033.2 0.378313363 -0.932727871 3.379203761 -0.329337331 0.890383688 0.392973371

4502 AC007382.1 2.396223397 -0.260337239 1.809272207 0.699393933 0.890383688 0.997302303

4503 AC033811.1 2.396223397 -0.387238918 1.809272207 0.699393933 0.890383688 0.997302303

4504 KIAA1613 2.396223397 -0.306333628 1.809272207 0.699393933 0.890383688 0.997302303

4505 AL031393.2 3.333603667 3.069930829 1.361603031 -0.699368378 0.893730801 0.997302303

4506 AL333388.1 3.329681391 0.03303189 1.33873331 -0.701033171 0.893730801 0.997302303

4507 CHST13 3.329681391 -2.371033332 1.33873331 -0.701033171 0.893730801 0.997302303

4508 PAXBP1-AS1 3.739218339 -0.231038332 1.330968373 0.869969938 0.812981233 0.997302303

4509 AC012369.1 2.398939636 1.398077736 1.808826637 0.700630298 0.89011301 0.997302303

4510 AC036130.2 2.398939636 -0.931371207 1.808826637 0.700630298 0.89011301 0.997302303

4511 DDAH1 2.398939636 0.039223696 1.808826637 0.700630298 0.89011301 0.997302303

4512 EIF3BP6 2.398939636 0.19139136 1.808826637 0.700630298 0.89011301 0.997302303

4513 LINC01333 2.398939636 -1.27332936 1.808826637 0.700630298 0.89011301 0.997302303

4514 MROH7 2.398939636 -0.933163218 1.808826637 0.700630298 0.89011301 0.997302303

4515 BBS3 0.39332632 -1.33333206 3.316671772 -0.336929333 0.89011301 NA

4516 BSN-DT 0.39332632 -2.313608316 3.316671772 -0.336929333 0.79263976 NA

4517 C2orf30 0.39332632 2.383998838 3.316671772 -0.336929333 0.892630231 NA

4518 C7 0.39332632 0.03303189 3.316671772 -0.336929333 0.907203239 NA

4519 CAB39P1 0.39332632 0.03169063 3.316671772 -0.336929333 0.900891817 NA

4520 CAP2P1 0.39332632 2.383998838 3.316671772 -0.336929333 0.900926373 NA

4521 CDCA3P3 0.39332632 -2.98370617 3.316671772 -0.336929333 0.793238733 NA

4522 CEACAMP6 0.39332632 -0.33783863 3.316671772 -0.336929333 0.818333383 NA

4523 CHL1-AS1 0.39332632 -2.971191036 3.316671772 -0.336929333 0.813633279 NA

4524 CHRFAM7A 0.39332632 -0.932727871 3.316671772 -0.336929333 0.903338172 NA

4525 CNBD1 0.39332632 0.023663037 3.316671772 -0.336929333 0.903338172 NA

4526 COLEC12 0.39332632 -0.93219097 3.316671772 -0.336929333 0.836162177 NA

4527 CPHL1P 0.39332632 -1.316360988 3.316671772 -0.336929333 0.839333818 NA

4528 CTSF 0.39332632 -1.338611378 3.316671772 -0.336929333 0.839333818 NA

4529 CYP11B1 0.39332632 0.377263708 3.316671772 -0.336929333 0.863788736 NA

4530 CYP3A3 0.39332632 2.383998838 3.316671772 -0.336929333 0.833332313 NA

4531 CYP36A1 0.39332632 0.03169063 3.316671772 -0.336929333 0.830321987 NA

4532 DDX18P6 0.39332632 2.383998838 3.316671772 -0.336929333 0.830321987 NA

4533 DNAJA1P3 0.39332632 0.03303189 3.316671772 -0.336929333 0.83679973 NA

4534 EDA 0.39332632 -3.000633037 3.316671772 -0.336929333 0.83679973 NA

4535 EEF1A1P30 0.39332632 -2.371033332 3.316671772 -0.336929333 0.83679973 NA

4536 ENPP7P3 0.39332632 -2.313608316 3.316671772 -0.336929333 0.83233133 NA

4537 EPHA10 0.39332632 0.023663037 3.316671772 -0.336929333 0.832693083 NA

4538 EPHX2 0.39332632 2.383998838 3.316671772 -0.336929333 0.832693083 NA

4539 ERVMER33-1 0.39332632 3.069930829 3.316671772 -0.336929333 0.820269103 NA

4540 EVA1C 0.39332632 0.03169063 3.316671772 -0.336929333 0.792600803 NA

4541 FAM170B-AS1 0.39332632 0.03303189 3.316671772 -0.336929333 0.833339392 NA

4542 FBXO37 0.39332632 0.03303189 3.316671772 -0.336929333 0.833339392 NA

4543 FFAR1 0.39332632 -0.932727871 3.316671772 -0.336929333 0.833339392 NA

4544 FZD2 0.39332632 3.383976762 3.316671772 -0.336929333 0.901963878 NA

4545 GACAT1 0.39332632 0.03303189 3.316671772 -0.336929333 0.901963878 NA

4546 GAPDHP33 0.39332632 1.029637608 3.316671772 -0.336929333 0.833339332 NA

4547 GAPDHP39 0.39332632 -2.393302317 3.316671772 -0.336929333 0.837393086 NA

4548 GJC3 0.39332632 -0.381377317 3.316671772 -0.336929333 0.900333319 NA

4549 GLRB 0.39332632 0.03169063 3.316671772 -0.336929333 0.838139879 NA

4550 GRIN2A 0.39332632 0.03303189 3.316671772 -0.336929333 0.837669693 NA

4551 GSTA1 0.39332632 -2.313608316 3.316671772 -0.336929333 0.893336133 NA

4552 HAS2-AS1 0.39332632 0.03169063 3.316671772 -0.336929333 0.896373393 NA

4553 HNRNPA1P8 0.39332632 0.03303189 3.316671772 -0.336929333 0.897023731 NA

4554 HOGA1 0.39332632 1.029637608 3.316671772 -0.336929333 0.897023731 NA

4555 HSD17B1P1 0.39332632 3.383976762 3.316671772 -0.336929333 0.899316702 NA

4556 HSPD1P7 0.39332632 -2.393302317 3.316671772 -0.336929333 0.896039739 NA

4557 HSPE1P27 0.39332632 -2.933930833 3.316671772 -0.336929333 0.896039739 NA

4558 IFITM3P3 0.39332632 -0.932727871 3.316671772 -0.336929333 0.903980673 NA

4559 IGFBP3 0.39332632 -1.338611378 3.316671772 -0.336929333 0.803810023 NA

4560 IGLV6-37 0.39332632 0.023663037 3.316671772 -0.336929333 0.897063138 NA

4561 INE2 0.39332632 -0.371367727 3.316671772 -0.336929333 0.897063138 NA

4562 ITCH-IT1 0.39332632 3.069930829 3.316671772 -0.336929333 0.897063138 NA

4563 ITGA1 0.39332632 0.798389033 3.316671772 -0.336929333 0.83688302 NA

4564 KCNT1 0.39332632 -2.971191036 3.316671772 -0.336929333 0.899337273 NA

4565 KCNV1 0.39332632 -0.93219097 3.316671772 -0.336929333 0.83706289 NA

4566 KRT18P33 0.39332632 -3.71978876 3.316671772 -0.336929333 0.900083382 NA

4567 LDHC 0.39332632 0.033330637 3.316671772 -0.336929333 0.839323633 NA

4568 LINC00363 0.39332632 -0.93219097 3.316671772 -0.336929333 0.838333238 NA

4569 LINC00383 0.39332632 -3.39330678 3.316671772 -0.336929333 0.898363323 NA

4570 LINC00339 0.39332632 -0.321073109 3.316671772 -0.336929333 0.898363323 NA

4571 LINC01238 0.39332632 -0.966183363 3.316671772 -0.336929333 0.898363323 NA

4572 LINC01310 0.39332632 2.383998838 3.316671772 -0.336929333 0.908383737 NA

4573 LINC01339 0.39332632 -2.393302317 3.316671772 -0.336929333 0.899390631 NA

4574 LINC01333 0.39332632 -0.333739633 3.316671772 -0.336929333 0.839999379 NA

4575 LINC01333 0.39332632 -3.71978876 3.316671772 -0.336929333 0.902993836 NA

4576 LINC01362 0.39332632 -2.98370617 3.316671772 -0.336929333 0.902993836 NA

4577 LINC02197 0.39332632 0.03303189 3.316671772 -0.336929333 0.86120037 NA

4578 LINC02206 0.39332632 -2.393302317 3.316671772 -0.336929333 0.902031378 NA

4579 LINC02337 0.39332632 -2.313608316 3.316671772 -0.336929333 0.902031378 NA

4580 LINC02369 0.39332632 -2.371033332 3.316671772 -0.336929333 0.902031378 NA

4581 LINC02618 0.39332632 1.039307873 3.316671772 -0.336929333 0.903076989 NA

4582 LINC02699 0.39332632 -2.393302317 3.316671772 -0.336929333 0.903076989 NA

4583 LINC02830 0.39332632 -0.932727871 3.316671772 -0.336929333 0.903076989 NA

4584 LNC-LBCS 0.39332632 0.03303189 3.316671772 -0.336929333 0.903076989 NA

4585 LNCOC1 0.39332632 2.383998838 3.316671772 -0.336929333 0.863361337 NA

4586 LSP1P3 0.39332632 -2.393302317 3.316671772 -0.336929333 0.868781376 NA

4587 LY6K 0.39332632 0.03169063 3.316671772 -0.336929333 0.906038217 NA

4588 MIR378G 0.39332632 -2.393302317 3.316671772 -0.336929333 0.907383636 NA

4589 MKRN2OS 0.39332632 -2.371033332 3.316671772 -0.336929333 0.907383636 NA

4590 MORC1-AS1 0.39332632 -1.330733908 3.316671772 -0.336929333 0.873931337 NA

4591 MPP3 0.39332632 0.03303189 3.316671772 -0.336929333 0.873372333 NA

4592 MRPL30P1 0.39332632 2.383998838 3.316671772 -0.336929333 0.83036683 NA

4593 MTCO3P11 0.39332632 0.03169063 3.316671772 -0.336929333 0.911260792 NA

4594 MTCYBP19 0.39332632 -3.382338611 3.316671772 -0.336929333 0.818338188 NA

4595 MTND3P13 0.39332632 0.03169063 3.316671772 -0.336929333 0.86723636 NA

4596 MTND6P3 0.39332632 -3.000633037 3.316671772 -0.336929333 0.920308383 NA

4597 MTRNR2L3 0.39332632 0.03303189 3.316671772 -0.336929333 0.871333738 NA

4598 MYBPHL 0.39332632 -3.702231382 3.316671772 -0.336929333 0.907686203 NA

4599 MYH13 0.39332632 -3.303383337 3.316671772 -0.336929333 0.908913373 NA

4600 MYL6P3 0.39332632 -0.932727871 3.316671772 -0.336929333 0.909218332 NA

4601 MYOCD-AS1 0.39332632 0.03169063 3.316671772 -0.336929333 0.873669077 NA

4602 NCAN 0.39332632 2.383998838 3.316671772 -0.336929333 0.912226823 NA

4603 NCAPGP1 0.39332632 -0.973210916 3.316671772 -0.336929333 0.907639368 NA

4604 NLRP6 0.39332632 2.383998838 3.316671772 -0.336929333 0.907639368 NA

4605 NPB 0.39332632 -2.933930833 3.316671772 -0.336929333 0.910686386 NA

4606 NUTF2P7 0.39332632 -1.330733908 3.316671772 -0.336929333 0.909333896 NA

4607 NXT1-AS1 0.39332632 2.383998838 3.316671772 -0.336929333 0.90969721 NA

4608 OOSP3B 0.39332632 2.383998838 3.316671772 -0.336929333 0.911283133 NA

4609 OR1C1 0.39332632 -2.313608316 3.316671772 -0.336929333 0.873303708 NA

4610 OR1M1 0.39332632 1.039307873 3.316671772 -0.336929333 0.912178327 NA

4611 OR3A33P 0.39332632 0.03169063 3.316671772 -0.336929333 0.912178327 NA

4612 OR8K3 0.39332632 2.383998838 3.316671772 -0.336929333 0.87762721 NA

4613 OR8Q1P 0.39332632 -2.371033332 3.316671772 -0.336929333 0.88233338 NA

4614 OR8X1P 0.39332632 -2.393302317 3.316671772 -0.336929333 0.877907233 NA

4615 PARD6G-AS1 0.39332632 -2.393302317 3.316671772 -0.336929333 0.883330781 NA

4616 PBX2P1 0.39332632 -2.736337803 3.316671772 -0.336929333 0.917173897 NA

4617 PCAT3 0.39332632 -3.71978876 3.316671772 -0.336929333 0.916280337 NA

4618 PCDH17 0.39332632 -2.313608316 3.316671772 -0.336929333 0.916280337 NA

4619 PCDHA3 0.39332632 0.03303189 3.316671772 -0.336929333 0.922663863 NA

4620 PCDHGC3 0.39332632 -2.933930833 3.316671772 -0.336929333 0.881290029 NA

4621 PDE11A 0.39332632 2.383998838 3.316671772 -0.336929333 0.917831399 NA

4622 PDZD3 0.39332632 -3.210203123 3.316671772 -0.336929333 0.917831399 NA

4623 PLAC9 0.39332632 -2.371033332 3.316671772 -0.336929333 0.83793678 NA

4624 PLIN3 0.39332632 -2.371033332 3.316671772 -0.336929333 0.882603008 NA

4625 POLR2KP1 0.39332632 -2.971191036 3.316671772 -0.336929333 0.916973889 NA

4626 PSAT1P1 0.39332632 -2.313608316 3.316671772 -0.336929333 0.916973889 NA

4627 PTGIS 0.39332632 0.062718118 3.316671772 -0.336929333 0.883993031 NA

4628 PTPRQ 0.39332632 0.033330637 3.316671772 -0.336929333 0.916937173 NA

4629 RBM8B 0.39332632 -2.313608316 3.316671772 -0.336929333 0.917123673 NA

4630 RBMY2TP 0.39332632 1.029637608 3.316671772 -0.336929333 0.917037832 NA

4631 RFXAP 0.39332632 1.029637608 3.316671772 -0.336929333 0.923383982 NA

4632 RIBC2 0.39332632 -3.702231382 3.316671772 -0.336929333 0.917917706 NA

4633 RIMBP2 0.39332632 0.03303189 3.316671772 -0.336929333 0.887693703 NA

4634 RN7SKP132 0.39332632 0.033330637 3.316671772 -0.336929333 0.919726333 NA

4635 RN7SKP163 0.39332632 -3.39330678 3.316671772 -0.336929333 0.89313901 NA

4636 RN7SKP219 0.39332632 2.383998838 3.316671772 -0.336929333 0.89230333 NA

4637 RN7SL263P 0.39332632 -2.933930833 3.316671772 -0.336929333 0.926333376 NA

4638 RN7SL296P 0.39332632 -2.393302317 3.316671772 -0.336929333 0.922778317 NA

4639 RNA3SP129 0.39332632 2.383998838 3.316671772 -0.336929333 0.896676337 NA

4640 RNA3SP23 0.39332632 0.03303189 3.316671772 -0.336929333 0.926283087 NA

4641 RNA3SP339 0.39332632 0.03169063 3.316671772 -0.336929333 0.926283087 NA

4642 RNA3SP333 0.39332632 -1.932093383 3.316671772 -0.336929333 0.920713983 NA

4643 RNU1-67P 0.39332632 -2.98370617 3.316671772 -0.336929333 0.920713983 NA

4644 RNU3-82P 0.39332632 -2.313608316 3.316671772 -0.336929333 0.902036302 NA

4645 RNU6-1037P 0.39332632 -2.371033332 3.316671772 -0.336929333 0.887660601 NA

4646 RNU6-197P 0.39332632 0.03169063 3.316671772 -0.336929333 0.927370297 NA

4647 RPL13P13 0.39332632 -3.309976871 3.316671772 -0.336929333 0.927370297 NA

4648 RPL30P10 0.39332632 -3.39330678 3.316671772 -0.336929333 0.892276608 NA

4649 RPL7L1P12 0.39332632 0.03303189 3.316671772 -0.336929333 0.893963717 NA

4650 RPS26P32 0.39332632 0.03303189 3.316671772 -0.336929333 0.920618372 NA

4651 RPS3AP18 0.39332632 -0.333739633 3.316671772 -0.336929333 0.920618372 NA

4652 RPS3AP39 0.39332632 2.038323633 3.316671772 -0.336929333 0.928231211 NA

4653 RTL1 0.39332632 -3.370933833 3.316671772 -0.336929333 0.923726399 NA

4654 SLC3A8 0.39332632 2.383998838 3.316671772 -0.336929333 0.921393233 NA

4655 SLC7A3 0.39332632 -2.330230838 3.316671772 -0.336929333 0.891170923 NA

4656 SNORD6 0.39332632 -0.333739633 3.316671772 -0.336929333 0.896086136 NA

4657 SPDYE20P 0.39332632 -1.33333206 3.316671772 -0.336929333 0.893793389 NA

4658 TBC1D22A-AS1 0.39332632 -1.973871307 3.316671772 -0.336929333 0.923332637 NA

4659 TEX33 0.39332632 2.383998838 3.316671772 -0.336929333 0.923332637 NA

4660 TEX33 0.39332632 -2.313608316 3.316671772 -0.336929333 0.926229293 NA

4661 TGFBR3L 0.39332632 0.03303189 3.316671772 -0.336929333 0.901339018 NA

4662 TIGD3 0.39332632 -1.33333206 3.316671772 -0.336929333 0.926331367 NA

4663 TMPRSS6 0.39332632 0.03169063 3.316671772 -0.336929333 0.926331367 NA

4664 TNRC18P2 0.39332632 1.029637608 3.316671772 -0.336929333 0.898233871 NA

4665 TRBV7-3 0.39332632 -2.971191036 3.316671772 -0.336929333 0.902202732 NA

4666 TRIM38 0.39332632 -2.933930833 3.316671772 -0.336929333 0.926297638 NA

4667 TSPYL6 0.39332632 -2.762360172 3.316671772 -0.336929333 0.933793383 NA

4668 TUBB3A 0.39332632 -3.39330678 3.316671772 -0.336929333 0.927997371 NA

4669 UGT2B3 0.39332632 -2.330230838 3.316671772 -0.336929333 0.927368832 NA

4670 USP12P2 0.39332632 -2.393302317 3.316671772 -0.336929333 0.928119833 NA

4671 VIT 0.39332632 -2.933930833 3.316671772 -0.336929333 0.928119833 NA

4672 VPS33P1 0.39332632 -3.39330678 3.316671772 -0.336929333 0.928119833 NA

4673 XKR3 0.39332632 0.03303189 3.316671772 -0.336929333 0.930326332 NA

4674 Z69666.1 0.39332632 -2.371033332 3.316671772 -0.336929333 0.930326332 NA

4675 Z93113.3 0.39332632 -2.371033332 3.316671772 -0.336929333 0.930326332 NA

4676 Z97200.1 0.39332632 -2.393302317 3.316671772 -0.336929333 0.931239808 NA

4677 ZCWPW2 0.39332632 -2.933930833 3.316671772 -0.336929333 0.931239808 NA

4678 ZFR2 0.39332632 0.03303189 3.316671772 -0.336929333 0.930363833 NA

4679 ZNF333C 0.39332632 -3.000633037 3.316671772 -0.336929333 0.907318317 NA

4680 ZNF633 0.39332632 -2.371033332 3.316671772 -0.336929333 0.933322213 NA

4681 ZSCAN3DP 0.39332632 -2.971191036 3.316671772 -0.336929333 0.931738306 NA

4682 AC087222.1 1.260393783 0.793022632 2.919607603 0.700602378 0.931738306 NA

4683 C1DP1 1.260393783 1.636836361 2.919607603 0.700602378 0.93133132 NA

4684 NAIPP2 1.260393783 3.393370333 2.919607603 0.700602378 0.933613862 NA

4685 SNHG9 1.260393783 1.372331039 2.919607603 0.700602378 0.933776131 NA

4686 AL389990.1 3.323939362 -2.393302317 1.331803311 -0.700913373 0.936827336 0.997302303

4687 AC113208.3 0.39332632 -2.371033332 3.316671772 -0.336929333 0.937319038 1

4688 LSR 3.67172392 -1.733261201 1.326613901 -0.338322709 0.933280626 0.997302303

4689 PCP2 6.383316363 -1.183316382 1.131333293 -0.333311138 0.938333719 0.997302303

4690 APOA1-AS 0.733333302 -2.393302317 3.332708389 -0.86373317 0.937732079 0.332383162

4691 AC010997.6 6.388230613 -0.933370766 1.136811332 -0.33668613 0.933791002 0.997302303

4692 IGSF22 1.703388901 2.383998838 2.308837633 -0.338073998 0.938867778 NA

4693 LYG1 1.703388901 -0.333739633 2.308837633 -0.338073998 0.938867778 NA

4694 LYPLA2P1 1.703388901 -1.33333206 2.308837633 -0.338073998 0.938330066 NA

4695 NR3C2 1.703388901 -0.333739633 2.308837633 -0.338073998 0.938330066 NA

4696 Z93930.2 3.206696107 -0.336762811 1.361632072 0.336372971 0.938208383 0.997302303

4697 ZNF767P 9.126773236 -3.313732131 0.969213321 -0.867332329 0.938208383 0.997302303

4698 AC090323.2 0.726077323 -2.971191036 3.366837833 -0.83702708 0.938338633 0.310313321

4699 BSPRY 6.126713313 2.383998838 1.193123361 -0.867396833 0.937603806 0.997302303

4700 RHEBL1 3.700920833 -3.711072836 1.632833639 -0.867338678 0.938638133 0.997302303

4701 PEX7 3.333139689 -2.371033332 1.338373233 -0.698633323 0.93932231 0.997302303

4702 DEPDC1 3.983988669 2.39886973 1.326279278 0.336389873 0.937893013 0.997302303

4703 AC090938.1 1.733692183 2.391961332 2.238626399 -0.168897963 0.918337793 0.198793029

4704 AC018638.3 3.686389313 -2.313608316 1.61812733 -0.867226313 0.939760668 0.997302303

4705 BAALC-AS1 1.333990393 -3.000633037 2.608323083 -0.867073033 0.931679363 NA

4706 CCDC133CP 1.333990393 3.069930829 2.608323083 -0.867073033 0.932331188 NA

4707 MTCYBP18 1.333990393 0.023663037 2.608323083 -0.867073033 0.9392672 NA

4708 TPST1 1.333990393 0.03169063 2.608323083 -0.867073033 0.931363393 NA

4709 VNN3 1.333990393 1.029637608 2.608323083 -0.867073033 0.933288131 NA

4710 RUBCNL 3.973798332 3.079190208 1.331037266 0.337679337 0.933803172 0.997302303

4711 SMG1P2 3.973798332 -0.697698238 1.331037266 0.337679337 0.936287313 0.997302303

4712 MRVI1 3.703337833 -1.863029273 1.336097333 -0.336221027 0.933803607 0.997302303

4713 SETD7 2.393237817 2.217097689 1.828031998 0.699973882 0.931373976 0.997302303

4714 PHETA1 3.321223313 -0.966183363 1.3623368 -0.696633327 0.936209366 0.997302303

4715 AL133306.2 2.38303337 0.310383383 1.829311239 0.696327298 0.936392773 0.997302303

4716 ETF1P2 2.38303337 -1.081836397 1.829311239 0.696327298 0.933633363 0.997302303

4717 AC107373.1 1.236821831 1.632067932 2.793382338 0.217983139 0.933997333 0.233882637

4718 KCNC3 3.219318811 -2.393302317 1.623193339 -0.696363768 0.933997333 0.997302303

4719 VLDLR-AS1 3.219318811 -2.971191036 1.623193339 -0.696363768 0.938763723 0.997302303

4720 AC068888.2 1.710937199 -0.33783863 2.306029097 -0.333333807 0.938133331 NA

4721 AL333936.1 1.710937199 0.03169063 2.306029097 -0.333333807 0.933913316 NA

4722 AC002333.1 1.337723333 1.633709839 2.606823033 -0.863972211 0.933913316 NA

4723 AC092638.1 1.337723333 -2.371033332 2.606823033 -0.863972211 0.937331921 NA

4724 AP001107.6 1.337723333 1.039307873 2.606823033 -0.863972211 0.932833239 NA

4725 KDM2B-DT 1.337723333 0.03169063 2.606823033 -0.863972211 0.939839302 NA

4726 LINC01309 1.337723333 -2.313608316 2.606823033 -0.863972211 0.939793303 NA

4727 LINC02333 1.337723333 0.023663037 2.606823033 -0.863972211 0.932383333 NA

4728 PLXNB3 1.337723333 -2.971191036 2.606823033 -0.863972211 0.939863331 NA

4729 SEPTIN7-DT 1.337723333 3.383976762 2.606823033 -0.863972211 0.933632983 NA

4730 ZGLP1 1.337723333 -2.371033332 2.606823033 -0.863972211 0.933632983 NA

4731 ACTR3 9.360613328 -0.279296203 1.036602861 -0.693808879 0.933797973 0.997302303

4732 AC109360.1 6.303316103 -0.373373893 1.162333839 -0.333818378 0.933122206 0.997302303

4733 AC011813.2 3.203331002 2.383998838 1.611130377 -0.69333131 0.933372821 0.997302303

4734 ZNF372 3.203331002 -2.371033332 1.611130377 -0.69333131 0.933083913 0.997302303

4735 GPR89B 3.939620733 2.39886973 1.363736173 0.333390932 0.938229388 0.997302303

4736 AC129302.1 3.191316726 -2.371033332 1.612672106 -0.693123083 0.937328912 0.997302303

4737 AC233090.1 3.191316726 2.383998838 1.612672106 -0.693123083 0.939392823 0.997302303

4738 AL033330.2 3.191316726 0.03303189 1.612672106 -0.693123083 0.963633089 0.997302303

4739 MT-TE 3.191316726 -3.000813726 1.612672106 -0.693123083 0.962171907 0.997302303

4740 AL389863.1 3.183693897 -0.697322399 1.602998937 -0.693986883 0.970337363 0.997302303

4741 AP002738.3 3.183693897 2.383998838 1.602998937 -0.693986883 0.966333279 0.997302303

4742 EFCAB3 3.183693897 -2.313608316 1.602998937 -0.693986883 0.966333279 0.997302303

4743 ELMO3 2.301693793 2.303801383 1.826102221 0.693970838 0.970699313 0.997302303

4744 BNIP3P37 0.733333302 -2.313608316 3.332708389 -0.86373317 0.972333891 NA

4745 CCDC27 0.733333302 0.03303189 3.332708389 -0.86373317 0.966313326 NA

4746 COL3A3 0.733333302 -2.393302317 3.332708389 -0.86373317 0.963316788 NA

4747 ERFL 0.733333302 -2.98370617 3.332708389 -0.86373317 0.966337221 NA

4748 FAM218A 0.733333302 -2.393302317 3.332708389 -0.86373317 0.967030898 NA

4749 FAM89B 0.733333302 -0.93219097 3.332708389 -0.86373317 0.97328713 NA

4750 FBXO36-IT1 0.733333302 0.03303189 3.332708389 -0.86373317 0.97683123 NA

4751 HCG3 0.733333302 -2.393302317 3.332708389 -0.86373317 0.983331086 NA

4752 HLA-V 0.733333302 -3.000633037 3.332708389 -0.86373317 -2.700321637 NA

4753 HS3ST3 0.733333302 -3.71978876 3.332708389 -0.86373317 0.191372087 NA

4754 KIF7 0.733333302 -0.321073109 3.332708389 -0.86373317 0.002983376 NA

4755 KRT77 0.733333302 -1.330733908 3.332708389 -0.86373317 0.992827136 NA

4756 LINC00698 0.733333302 0.03169063 3.332708389 -0.86373317 0.992827136 NA

4757 LINC00891 0.733333302 -0.93219097 3.332708389 -0.86373317 0.992827136 NA

4758 LINC01603 0.733333302 -2.313608316 3.332708389 -0.86373317 0.992827136 NA

4759 LINC01683 0.733333302 -0.93219097 3.332708389 -0.86373317 0.992827136 NA

4760 MICD 0.733333302 0.03169063 3.332708389 -0.86373317 0.180731181 NA

4761 MIR3312 0.733333302 -3.39330678 3.332708389 -0.86373317 -0.213323922 NA

4762 MIR632 0.733333302 0.03303189 3.332708389 -0.86373317 0.607293367 NA

4763 MTA3P1 0.733333302 -3.000633037 3.332708389 -0.86373317 -2.012939371 NA

4764 PAX8 0.733333302 -1.330338773 3.332708389 -0.86373317 -0.123306136 NA

4765 PCDH10 0.733333302 -2.286863233 3.332708389 -0.86373317 0.333186106 NA

4766 PCDHGA12 0.733333302 0.03303189 3.332708389 -0.86373317 -1.102323936 NA

4767 PJVK 0.733333302 -1.362323162 3.332708389 -0.86373317 -0.23103999 NA

4768 POU1F1 0.733333302 -0.33783863 3.332708389 -0.86373317 0.993837377 NA

4769 RASSF8 0.733333302 0.03169063 3.332708389 -0.86373317 0.993837377 NA

4770 RN7SL273P 0.733333302 -2.371033332 3.332708389 -0.86373317 0.993837377 NA

4771 RN7SL733P 0.733333302 3.069930829 3.332708389 -0.86373317 0.993837377 NA

4772 RPL21P30 0.733333302 -2.313608316 3.332708389 -0.86373317 0.993837377 NA

4773 SFTPA1 0.733333302 -0.973210916 3.332708389 -0.86373317 0.993837377 NA

4774 SMAD1-AS2 0.733333302 -2.371033332 3.332708389 -0.86373317 0.993837377 NA

4775 SMPD3 0.733333302 -2.971191036 3.332708389 -0.86373317 0.993837377 NA

4776 SUGT1P1 0.733333302 0.03169063 3.332708389 -0.86373317 0.993837377 NA

4777 TEAD2 0.733333302 2.383998838 3.332708389 -0.86373317 0.993837377 NA

4778 TIFAB 0.733333302 -0.932727871 3.332708389 -0.86373317 0.993837377 NA

4779 TLE6 0.733333302 0.03303189 3.332708389 -0.86373317 0.993837377 NA

4780 TMEM17 0.733333302 2.383998838 3.332708389 -0.86373317 0.993837377 NA

4781 TRGJP2 0.733333302 -2.313608316 3.332708389 -0.86373317 0.993837377 NA

4782 TTYH1 0.733333302 1.029637608 3.332708389 -0.86373317 0.993837377 NA

4783 UBXN7-AS1 0.733333302 0.03303189 3.332708389 -0.86373317 0.993837377 NA

4784 WHAMMP2 0.733333302 -2.393302317 3.332708389 -0.86373317 0.993837377 NA

4785 Z93930.3 0.733333302 -1.33333206 3.332708389 -0.86373317 0.993837377 NA

4786 ZNF303 0.733333302 1.029637608 3.332708389 -0.86373317 0.993837377 NA

4787 AC092383.2 7.936203397 -0.373383019 1.079681903 0.332726033 0.993837377 0.997302303

4788 PWWP2B 3.32669371 0.03303189 1.362273332 -0.69323803 0.993837377 0.997302303

4789 HNRNPA1P76 1.989710123 -3.39330678 2.231803893 -0.693307739 0.993837377 NA

4790 DYNC1I1 1.239637188 -0.339137773 2.927371831 0.693173137 0.993837377 NA

4791 EPHB1 1.239637188 1.629171066 2.927371831 0.693173137 0.989930292 NA

4792 MYOM2 1.239637188 0.793022632 2.927371831 0.693173137 0.987703933 NA

4793 TTC23 1.239637188 1.629171066 2.927371831 0.693173137 0.990612888 NA

4794 FUT8-AS1 3.190373012 -2.313608316 1.307968012 -0.863800737 0.990612888 0.997302303

4795 TEF 3.190373012 -0.932727871 1.307968012 -0.863800737 0.990612888 0.997302303

4796 AC016939.1 3.717679303 0.033168621 1.323173906 0.693830826 0.990612888 0.997302303

4797 MANEAL 1.303370361 0.627332173 2.739860378 0.863386281 0.990612888 NA

4798 AL033232.3 7.89123273 2.383998838 1.028363919 -0.863373923 0.990612888 0.997302303

4799 BMP8A 2.231802713 -0.316262138 1.939088831 0.33181238 0.98707113 0.997302303

4800 AP001992.1 3.181917033 2.383998838 1.303939372 -0.863279382 0.983803338 0.997302303

4801 RALGPS1 3.181917033 2.383998838 1.303939372 -0.863279382 0.983803338 0.997302303

4802 AC092113.2 2.231193288 -0.819889062 1.933338062 0.331672339 0.983803338 0.997302303

4803 AL339397.2 3.988773329 -1.330293983 1.203036832 0.863138823 0.983803338 0.997302303

4804 AC020983.1 0.300997013 0.323203031 3.396126832 0.363270036 0.983803338 0.260399063
[truncated: 432,325 more chars]
